# Discovery of Highly Functionalized 5-hydroxy-2*H*-pyrrol-2-ones That Exhibit Antiestrogenic Effects in Breast and Endometrial Cancer Cells and Potentiate the Antitumoral Effect of Tamoxifen

**DOI:** 10.3390/cancers14215174

**Published:** 2022-10-22

**Authors:** Miguel Guerra-Rodríguez, Priscila López-Rojas, Ángel Amesty, Haidée Aranda-Tavío, Yeray Brito-Casillas, Ana Estévez-Braun, Leandro Fernández-Pérez, Borja Guerra, Carlota Recio

**Affiliations:** 1 Farmacología Molecular y Traslacional (BIOPharm), Instituto Universitario de Investigaciones Biomédicas y Sanitarias (IUIBS), Universidad de Las Palmas de Gran Canaria (ULPGC), Paseo Blas Cabrera Felipe Físico s/n, 35016 Las Palmas de Gran Canaria, Spain; 2 Departamento de Química Orgánica, Instituto Universitario de Bio-Orgánica Antonio González (IUBO AG), Universidad de La Laguna (ULL), Avda. Astrofísico Fco. Sánchez 2, 38206 La Laguna, Spain

**Keywords:** 5-hydroxy-2*H*-pyrrol-2-ones, ER, antiestrogen, breast cancer, endometrial cancer, synergism

## Abstract

**Simple Summary:**

About 70% of the breast tumors diagnosed are estrogen receptor (ER)-positive and depend on estrogens and the interactions with their ER to grow and survive; their therapeutic treatment has a good clinical prognosis and effectiveness, but antitumoral treatment resistances and undesirable side effects (ovarian cysts, endometrial cancer, or blood clots) remain clinically challenging. This justifies the development of new drugs that modulate ER activity since it is considered a clinically validated therapeutic target. The goal of this study was the identification and the preclinical pharmacological evaluation of new structures with antitumoral and/or antiestrogenic properties with alternative or complementary mechanisms of action to the endocrine therapy used in the gold-standard treatment of ER-positive breast cancer. Thus, we identified two leading compounds (highly-functionalized 5-hydroxy-2*H*-pyrrol-2-ones) with potential antitumoral effects and scarce estrogenic activity, which offers a pharmacological opportunity to progress in the study of ER-positive breast cancer treatment.

**Abstract:**

Tamoxifen improves the overall survival rate in hormone receptor-positive breast cancer patients. However, despite the fact that it exerts antagonistic effects on the ERα, it can act as a partial agonist, resulting in tumor growth in estrogen-sensitive tissues. In this study, highly functionalized 5-hydroxy-2*H*-pyrrol-2-ones were synthesized and evaluated by using ERα- and phenotype-based screening assays. Compounds **32** and **35** inhibited 17β-estradiol (E2)-stimulated ERα-mediated transcription of the luciferase reporter gene in breast cancer cells without inhibition of the transcriptional activity mediated by androgen or glucocorticoid receptors. Compound **32** regulated E2-stimulated ERα-mediated transcription by partial antagonism, whereas compound **35** caused rapid and non-competitive inhibition. Monitoring of 2D and 3D cell growth confirmed potent antitumoral effects of both compounds on ER-positive breast cancer cells. Furthermore, compounds **32** and **35** caused apoptosis and blocked the cell cycle of ER-positive breast cancer cells in the sub-G1 and G0/G1 phases. Interestingly, compound **35** suppressed the functional activity of ERα in the uterus, as demonstrated by the inhibition of E2-stimulated transcription of estrogen and progesterone receptors and alkaline phosphatase enzymatic activity. Compound **35** showed a relatively low binding affinity with ERα. However, its antiestrogenic effect was associated with an increased polyubiquitination and a reduced protein expression of ERα. Clinically relevant, a possible combinatory therapy with compound **35** may enhance the antitumoral efficacy of 4-hydroxy-tamoxifen in ER-positive breast cancer cells. In silico ADME predictions indicated that these compounds exhibit good drug-likeness, which, together with their potential antitumoral effects and their lack of estrogenic activity, offers a pharmacological opportunity to deepen the study of ER-positive breast cancer treatment.

## 1. Introduction

Breast cancer (BC) is the leading cause of cancer death worldwide among women [1,2]. Around 70% of BC cases are estrogen receptor-positive (ER+), which means they depend on estrogens and their interaction with ERα for their survival and progression [3]. ERα plays an essential role in the carcinogenesis and progression of ER+ BC, thus standing out as a key therapeutic target [4,5,6,7]. Accordingly, current pharmacological treatment for ER+ BC includes aromatase inhibitors (AIs) [8,9], selective ERα modulators (SERMs) (e.g., tamoxifen (TAM)), which are partial antiestrogens [5], or selective ERα degraders (SERDs) (e.g., fulvestrant), which are pure antiestrogens [10,11]. However, despite the fact that these endocrine therapies have been demonstrated to be highly versatile in the treatment of ER+ BC, they show some key limitations. First, nearly half of patients treated with AIs develop resistance; secondly, the long-term effectiveness of TAM is limited by the development of resistance in nearly all patients with metastatic BC and in 40% of patients with primary BC [12,13]. Furthermore, BC resistance is still dependent on the constitutive activation of ERα, which renders AIs and SERMs ineffective [14,15]. In addition, TAM, and its more active form, 4-hydroxy-tamoxifen (4-OHTAM), can increase the risk of developing blood clots and endometrial cancer, which is linked to its partial estrogenic effects in these tissues [16,17]. In addition, TAM can act through ERα-independent targets that result in both beneficial and undesirable side effects [5,18,19].

Interestingly, fulvestrant binds to ERα promoting receptor ubiquitination and degradation by the proteasome, thus is an alternative therapy in the treatment of ER+ BC patients under endocrine therapy resistance and has been approved for the treatment of metastatic ERα+ BC following antiestrogen therapy [10,11]. However, this SERD can also induce resistance [20,21] and shows poor oral bioavailability, thus limiting its administration to inconvenient intramuscular injections [22].

Considering all mentioned above, deeper exploration needs to be conducted to identify new compounds with ideal antiestrogenic properties on ER+ BC and an improved therapeutic window [23]. 

Pyrrol-2-ones belong to a class of biologically active compounds [24,25,26,27] that possess different types of pharmacological activities; among others, inhibition of plasminogen activator inhibitor-1 (PAI-1) [28], anti-inflammatory [29] or antitumoral effects [24] have been reported on ER− and ER+ BC cells. In this study, we synthesized and characterized a new chemical library of highly functionalized 5-hydroxy-*2H*-pyrrol-2-ones by using an in-silico modeling approach [30,31] followed by phenotype- and ERα-based screening assays in ERα-positive breast and endometrial cancer cells in order to identify new therapeutic strategies that overcome major limitations of current ER+ BC treatments.

## 2. Materials and Methods

### 2.1. General Methods

The reactions under microwave irradiation were carried out using a Biotage Initiator 2.5. Purification on column chromatography was performed on Merck silica gel 60. Silica gel GF plates were used for preparative TLC purification. NMR spectra were acquired using a Bruker Avance instrument. EIMS and HREIMS data were recorded in a VG Micromass ZAB-2F spectrometer. Melting points were taken on a capillary melting point apparatus and were uncorrected. 

### 2.2. General Procedure for the Synthesis of 5-hydroxy-3,5-diaryl-1,5-dihydro-2H-pyrrol-2-ones (***4**–**50***)

A mixture of 50 mg of chalcone and 2 equiv of isocianyde in 1 mL of H_2_O was placed in a microwave-special closed vial and irradiated for 1 h at 150 °C in a single-mode microwave oven. The reaction mixture was then cooled to room temperature. After removing the solvent under reduced pressure, the residue was purified on silica gel by preparative-TLC (hexane/EtOAc or DCM/MeOH) to afford the desired product.

#### 2.2.1. 1-cyclohexyl-5-hydroxy-3,5-diphenyl-1*H*-pyrrol-2(5*H*)-one (**4**)

Following the general procedure, a mixture of trans-chalcone (50 mg, 0.24 mmol) and cyclohexyl isocyanide (60.9 µL, 0.48 mmol) in H_2_O (1 mL) was irradiated at 150 °C for 1 h and after purification on silica gel preparative-TLC (Hex/AcOEt, 4:1), 57.5 mg (72%) of compound **4** were obtained as an amorphous white solid; mp.: 165–166 °C; ^1^H-NMR (600 MHz, CDCl_3_) *δ*: 7.85 (2H, d, *J* = 6.1 Hz), 7.50 (2H, d, *J* = 6.8 Hz), 7.31–7.38 (6H, m), 6.80 (1H, s), 3.17–3.31 (2H, m), 2.16–2.20 (1H, m), 2.05–2.09 (1H, m), 1.65–1.74 (2H, m), 1.63 (1H, d, *J* = 12.7 Hz), 1.52 (1H, d, *J* = 11.9 Hz), 1.41 (1H, d, *J* = 11.9 Hz), 1.08–1.27 (2H, m), 0.92–1.02 (1H, m); ^13^C-NMR (150 MHz, CDCl_3_) *δ*: 168.7 (C=O), 142.1 (CH), 137.4 (C), 135.0 (C), 130.8 (C), 129.1 (2CH), 128.5 (4CH), 127.7 (2CH), 126.3 (2CH), 90.5 (C), 53.1 (CH), 30.8 (CH_2_), 29.5 (CH_2_), 26.3 (2CH_2_), 25.3 (CH_2_); EIMS *m*/*z*: 333 (M^+^, 100), 251 (62), 236 (20), 235 (71), 105 (81), 98 (17), 77 (27); HREIMS: 333.1716 (calcd for C_22_H_23_NO_2_ 333.1729).

#### 2.2.2. 1-benzyl-5-hydroxy-3,5-diphenyl-1*H*-pyrrol-2(5*H*)-one (**5**)

Following the general procedure, a mixture of trans-chalcone (50 mg, 0.24 mmol) and benzyl isocyanide (59.7 µL, 0.48 mmol) in H_2_O (1 mL) was irradiated at 150 °C for 1 h and after purification on silica gel preparative-TLC (AcOEt/Hex, 4:1), 52.4 mg (64%) of compound **5** were obtained as an amorphous white solid; mp.: 149–151 °C; ^1^H-NMR (500 MHz, CDCl_3_) *δ*: 7.89 (2H, dd, *J* = 8.0, 2.0 Hz), 7.29–7.40 (8H, m), 7.22–7.26 (2H, m), 7.15–7.20 (3H, m), 6.97 (1H, s), 4.70 (1H, d, *J* = 15.0 Hz), 4.03 (1H, d, *J* = 15.0 Hz), 2.75 (1H, s); ^13^C-NMR (125 MHz, CDCl_3_) *δ*: 169.4 (C=O), 142.8 (CH), 138.1 (C), 136.8 (C), 134.5 (C), 130.6 (C), 129.4 (CH), 128.9 (2CH), 128.8 (2CH), 128.7 (CH), 128.6 (2CH), 128.4 (2CH), 127.6 (2CH), 127.3 (CH), 126.3 (2CH), 90.5 (C), 43.3 (CH_2_); EIMS *m*/*z*: 341 (M^+^, 100), 207 (22), 179 (25), 106 (20), 105 (21), 91 (77), 77 (19); HREIMS: 341.1416 (calcd for C_23_H_19_NO_2_ 341.1416).

#### 2.2.3. 5-hydroxy-1-(4-methoxyphenyl)-3,5-diphenyl-1*H*-pyrrol-2(5*H*)-one (**6**)

Following the general procedure, a mixture of trans-chalcone (50 mg, 0.24 mmol) and 4-methoxyphenyl isocyanide (65.9 mg, 0.48 mmol) in H_2_O (1 mL) was irradiated at 150 °C for 1 h and after purification on silica gel preparative-TLC (Hex/AcOEt, 4:1), 26.6 mg (31%) of compound **6** were obtained as an amorphous white solid; mp.: 127–129 °C; ^1^H-NMR (600 MHz, CDCl_3_) *δ*: 7.86–7.89 (2H, m), 7.40–7.42 (2H, m), 7.35–7.38 (3H, m), 7.22–7.30 (5H, m), 7.02 (1H, s), 6.70 (2H, d, *J* = 9.1 Hz), 3.70 (3H, s), 3.52 (1H, s); ^13^C-NMR (150 MHz, CDCl_3_) *δ*: 168.9 (C=O), 157.8 (C), 142.9 (CH), 137.3 (C), 134.4 (C), 130.6 (C), 129.4 (CH), 128.8 (2CH), 128.7 (2CH), 128.6 (C), 128.5 (CH), 127.8 (2CH), 127.0 (2CH), 126.3 (2CH), 114.1 (2CH), 91.3 (C), 55.4 (CH_3_); EIMS *m*/*z*: 357 (M^+^, 22), 236 (18), 235 (100), 122 (15), 105 (50), 77 (13); HREIMS: 357.1353 (calcd for C_23_H_19_NO_3_ 357.1365).

#### 2.2.4. 1-cyclohexyl-5-hydroxy-5-(4-methoxyphenyl)-3-phenyl-1*H*-pyrrol-2(5*H*)-one (**7**)

Following the general procedure, a mixture of (E)-4′-methoxychalcone (50 mg, 0.21 mmol) and cyclohexyl isocyanide (53.2 µL, 0.42 mmol) in H_2_O (1 mL) was irradiated at 150 °C for 1 h and after purification on silica gel preparative-TLC (Hex/AcOEt, 4:1), 28.9 mg (38%) of compound **7** were obtained as an amorphous white solid; mp.: 157–159 °C; ^1^H-NMR (600 MHz, CDCl_3_) *δ*: 7.87 (2H, dd, *J* = 8.2, 1.7 Hz), 7.42 (2H, d, *J* = 8.8 Hz), 7.34–7.39 (3H, m), 6.88 (2H, d, *J* = 8.8 Hz), 6.86 (1H, s), 3.82 (3H, s), 3.18–3.22 (1H, m), 2.56 (1H, s), 2.18–2.22 (1H, m), 2.09–2.13 (1H, m), 1.74 (1H, d, J = 11.4Hz), 1.62–1.68 (2H, m), 1.54 (1H, d, *J* = 11.4 Hz), 1.43 (1H, d, *J* = 11.4 Hz), 1.09–1.26 (2H, m), 0.95–1.06 (1H, m); ^13^C-NMR (125 MHz, CDCl_3_) *δ*: 168.6 (C=O), 159.8 (C), 141.9 (CH), 135.1 (C), 130.9 (C), 129.2 (CH), 129.1 (C), 128.6 (2CH), 127.7 (2CH), 127.6 (2CH), 113.9 (2CH), 90.6 (C), 55.4 (CH_3_), 53.1 (CH), 30.9 (CH_2_), 29.6 (CH_2_), 26.4 (CH_2_), 26.3 (CH_2_), 25.4 (CH_2_); EIMS *m*/*z*: 363 (M^+^, 41), 266 (20), 265 (70), 228 (26), 135 (63), 108 (100), 98 (13); HREIMS: 363.1834 (calcd for C_23_H_25_NO_3_ 363.1834).

#### 2.2.5. 1-benzyl-5-hydroxy-5-(4-methoxyphenyl)-3-phenyl-1*H*-pyrrol-2(5*H*)-one (**8**)

Following the general procedure, a mixture of (*E*)-4′-methoxychalcone (50 mg, 0.21 mmol) and benzyl isocyanide (52.1 µL, 0.42 mmol) in H_2_O (1 mL) was irradiated at 150 °C for 1 h and after purification on silica gel preparative-TLC (AcOEt/Hex, 4:1), 42.8 mg (55%) of compound **8** were obtained as an amorphous white solid; mp.: 153–155 °C; ^1^H-NMR (500 MHz, CDCl_3_) *δ*: 7.90–7.93 (2H, m), 7.27–7.42 (7H, m), 7.17–7.26 (3H, m), 7.00 (1H, s), 6.86 (2H, d, *J* = 8.9 Hz), 4.77 (1H, d, *J* = 15.0 Hz), 4.00 (1H, d, *J* = 15.0 Hz), 3.82 (3H, s), 2.35 (1H, s); ^13^C-NMR (125 MHz, CDCl_3_) *δ*: 169.3 (C=O), 160.0 (C), 142.8 (CH), 138.3 (C), 134.4 (C), 130.7 (C), 129.3 (CH), 128.9 (2CH), 128.7 (2CH), 128.6 (C), 128.5 (2CH), 127.7 (4CH), 127.3 (CH), 114.2 (2CH), 90.5 (C), 55.5 (CH_3_), 43.3 (CH_2_); EIMS *m*/*z*: 371 (M^+^, 25), 209 (22), 135 (11), 108 (100), 106 (12), 91 (39); HREIMS: 371.1537 (calcd for C_24_H_21_NO_3_ 371.1521).

#### 2.2.6. 5-hydroxy-1,5-bis(4-methoxyphenyl)-3-phenyl-1*H*-pyrrol-2(5*H*)-one (**9**)

Following the general procedure, a mixture of (*E*)-4′-methoxychalcone (50 mg, 0.21 mmol) and 4-methoxyphenyl isocyanide (57.6 mg, 0.42 mmol) in H_2_O (1 mL) was irradiated at 150 °C for 1 h and after purification on silica gel preparative-TLC (Hex/AcOEt, 4:1), 19.4 mg (24%) of compound **9** were obtained as an amorphous brown solid; mp.: 121–123 °C; ^1^H-NMR (500 MHz, CDCl_3_) *δ*: 7.89–7.91 (2H, m), 7.37–7.41 (3H, m), 7.34 (2H, d, *J* = 8.9 Hz), 7.28 (2H, d, *J* = 9.1 Hz), 7.04 (1H, s), 6.81 (2H, d, *J* = 8.9 Hz), 6.75 (2H, d, *J* = 9.1 Hz), 3.77 (3H, s), 3.73 (3H, s), 3.18 (1H, brs); ^13^C-NMR (125 MHz, CDCl_3_) *δ*: 168.7 (C=O), 159.8 (C), 157.8 (C), 142.9 (CH), 134.3 (C), 130.6 (C), 129.4 (CH), 129.1 (C), 128.7 (C), 128.6 (2CH), 127.8 (2CH), 127.6 (2CH), 127.0 (2CH), 114.1 (4CH), 91.2 (C), 55.4 (CH_3_), 55.3 (CH_3_); EIMS *m*/*z*: 387 (M^+^, 14), 266 (25), 265 (100), 135 (48); HREIMS: 387.1454 (calcd for C_24_H_21_NO_4_ 387.1471).

#### 2.2.7. 1-cyclohexyl-3-(3-fluorophenyl)-5-hydroxy-5-phenyl-1*H*-pyrrol-2(5*H*)-one (**10**)

Following the general procedure, a mixture of (*E*)-3-fluorochalcone (50 mg, 0.22 mmol) and cyclohexyl isocyanide (56.1 µL, 0.44 mmol) in H_2_O (1 mL) was irradiated at 150 °C for 1 h and after purification on silica gel preparative-TLC (Hex/AcOEt, 4:1), 40.3 mg (52%) of compound **10** were obtained as an amorphous white solid; mp.: 182–183 °C; ^1^H-NMR (600 MHz, CDCl_3_) *δ:* 7.86–7.90 (2H, m), 7.51 (2H, dd, *J* = 8.5, 1.9 Hz), 7.33–7.39 (3H, m), 7.05 (2H, t, *J* = 8.8 Hz), 6.84 (1H, s), 3.19–3.23 (1H, m), 2.83 (1H, s), 2.16–2.19 (1H, m), 2.06–2.09 (1H, m), 1.66–1.75 (1H, m), 1.62–1.69 (2H, m), 1.53 (1H, d, *J* = 12.2 Hz), 1.39–1.44 (1H, m), 1.09–1.22 (2H, m), 0.94–1.03 (1H, m); ^13^C-NMR (150 MHz, CDCl_3_) *δ*: 168.6 (C=O), 163.4 (C, *J* = 249.8 Hz), 141.5 (CH), 137.2 (C), 134.3 (C), 129.6 (CH, *J* = 7.6 Hz), 128.7 (CH, *J* = 8.1 Hz), 128.6 (2CH), 126.9 (C, *J* = 3.4 Hz), 126.3 (3CH), 115.6 (2CH, *J* = 21.1 Hz), 90.6 (C), 53.2 (CH), 30.9 (CH_2_), 29.6 (CH_2_), 26.3 (2CH_2_), 25.4 (CH_2_); EIMS *m*/*z*: 351 (M^+^, 100), 270 (14), 269 (68), 254 (18), 253 (64), 105 (80), 77 (23); HREIMS: 351.1651 (calcd for C_22_H_22_NO_2_F 351.1635).

#### 2.2.8. 1-benzyl-3-(3-fluorophenyl)-5-hydroxy-5-phenyl-1*H*-pyrrol-2(5*H*)-one (**11**)

Following the general procedure, a mixture of (*E*)-3-fluorochalcone (50 mg, 0.22 mmol) and benzyl isocyanide (54.9 µL, 0.44 mmol) in H_2_O (1 mL) was irradiated at 150 °C for 1 h and after purification on silica gel preparative-TLC (AcOEt/Hex, 4:1), 38.1 mg (48%) of compound **11** were obtained as an amorphous white solid; mp.: 142–144 °C; ^1^H-NMR (500 MHz, CDCl_3_) *δ*: 7.88–7.91 (2H, m), 7.36–7.40 (2H, m), 7.31–7.34 (3H, m), 7.22–7.27 (2H, m), 7.16–7.21 (3H, m), 7.06 (2H, t, *J* = 8.7 Hz), 6.95 (1H, s), 4.71 (1H, d, *J* = 15.0 Hz), 4.03 (1H, d, *J* = 15.1 Hz), 2.70 (1H, s); ^13^C-NMR (125 MHz, CDCl_3_) *δ*: 169.3 (C=O), 163.4 (C, *J* = 250.6 Hz), 142.3 (CH), 137.9 (C), 136.6 (C), 133.5 (C), 129.6 (CH, *J* = 8.1 Hz), 128.9 (3CH), 128.8 (3CH), 128.5 (2CH), 127.4 (CH), 126.7 (C, *J* = 3.5 Hz), 126.3 (2CH), 115.7 (CH, *J* = 22.0 Hz), 90.5 (C), 43.4 (CH_2_); EIMS *m*/*z*: 359 (M^+^, 84), 281 (10), 225 (17), 197 (22), 106 (18), 91 (100), 77 (14); HREIMS: 359.1313 (calcd for C_23_H_18_NO_2_F 359.1322).

#### 2.2.9. 3-(3-fluorophenyl)-5-hydroxy-1-(4-methoxyphenyl)-5-phenyl-1*H*-pyrrol-2(5*H*)-one (**12**)

Following the general procedure, a mixture of (*E*)-3-fluorochalcone (50 mg, 0.22 mmol) and 4-methoxyphenyl isocyanide (60.7 mg, 0.44 mmol) in H_2_O (1 mL) was irradiated at 150 °C for 1 h and after purification on silica gel preparative-TLC (Hex/AcOEt, 4:1), 23.2 mg (28%) of compound **12** were obtained as an amorphous white solid; mp.: 169–171 °C; ^1^H-NMR (500 MHz, CDCl_3_) *δ*: 7.88–7.92 (2H, m), 7.42 (2H, dd, *J* = 8.4, 1.6 Hz), 7.24–7.32 (5H, m), 7.07 (2H, t, *J* = 8.7 Hz), 7.02 (1H, s), 6.73 (2H, d, *J* = 9.1 Hz), 3.72 (3H, s); ^13^C-NMR (125 MHz, CDCl_3_) *δ*: 168.7 (C=O), 163.5 (C, *J* = 249.4 Hz), 157.9 (C), 142.3 (CH), 137.2 (C), 133.4 (C), 129.7 (CH, *J* = 8.8 Hz), 128.8 (3CH), 128.7 (CH), 128.5 (C), 126.9 (2CH), 126.6 (C, *J* = 3.6 Hz), 126.2 (2CH), 115.7 (2CH, *J* = 21.2 Hz), 114.1 (2CH), 91.3 (C), 55.4 (CH_3_); EIMS *m*/*z*: 375 (M^+^, 37), 254 (18), 253 (100), 122 (27), 105 (50), 77 (13); HREIMS: 375.1254 (calcd for C_23_H_18_NO_3_F 375.1271).

#### 2.2.10. 1-cyclohexyl-3-(4-fluorophenyl)-5-hydroxy-5-phenyl-1*H*-pyrrol-2(5*H*)-one (**13**)

Following the general procedure, a mixture of (*E*)-4-fluorochalcone (50 mg, 0.22 mmol) and cyclohexyl isocyanide (56.1 µL, 0.44 mmol) in H_2_O (1 mL) was irradiated at 150 °C for 1 h and after purification on silica gel preparative-TLC (Hex/AcOEt, 4:1), 33.3 mg (43%) of compound **13** were obtained as a brown oil; ^1^H-NMR (600 MHz, CDCl_3_) *δ*: 7.86–7.90 (2H, m), 7.51 (2H, dd, *J* = 8.4, 1.7 Hz), 7.33–7.39 (3H, m), 7.05 (2H, t, *J* = 8.8 Hz), 6.84 (1H, s), 3.19.-3.22 (1H, m), 2.84 (1H, s), 2.15–2.17 (1H, m), 2.06–2.09 (1H, m), 1.72 (1H, d, *J* = 12.7 Hz), 1.61–1.68 (2H, m), 1.53 (1H, d, *J* = 12.4 Hz), 1.42 (1H, d, *J* = 12.4 Hz), 1.08–1.26 (2H, m), 0.93–1.03 (1H, m); ^13^C-NMR (150 MHz, CDCl_3_) *δ*: 168.5 (C=O), 163.3 (C, *J* = 250.2 Hz), 141.4 (CH), 137.1 (C), 134.2 (C), 129.5 (2CH, *J* = 8.6 Hz), 128.6 (CH), 128.5 (2CH), 126.9 (C, *J* = 3.6 Hz), 126.2 (2CH), 115.5 (2CH, *J* = 21.1 Hz), 90.5 (C), 53.2 (CH), 30.9 (CH_2_), 29.6 (CH_2_), 26.3 (2CH_2_), 25.4 (CH_2_); EIMS *m*/*z*: 351 (M^+^, 100), 269 (96), 254 (22), 253 (79), 191 (25), 105 (99), 77 (41); HREIMS: 351.1624 (calcd for C_22_H_22_NO_2_F 351.1635).

#### 2.2.11. 1-benzyl-3-(4-fluorophenyl)-5-hydroxy-5-phenyl-1*H*-pyrrol-2(5*H*)-one (**14**)

Following the general procedure, a mixture of (*E*)-4-fluorochalcone (50 mg, 0.22 mmol) and benzyl isocyanide (54.9 µL, 0.44 mmol) in H_2_O (1 mL) was irradiated at 150 °C for 1 h and after purification on silica gel preparative-TLC (Hex/AcOEt, 4:1), 42.8 mg (54%) of compound **14** were obtained as a colorless oil; ^1^H-NMR (500 MHz, CDCl_3_) *δ*: 7.90–7.93 (2H, m), 7.38–7.41 (2H, m), 7.33–7.36 (3H, m), 7.26 (2H, d, *J* = 8.1 Hz), 7.18–7.23 (3H, m), 7.07 (2H, t, *J* = 8.8 Hz), 6.96 (1H, s), 4.73 (1H, d, *J* = 15.1 Hz), 4.04 (1H, d, *J* = 15.1 Hz), 2.71 (1H, s); ^13^C-NMR (125 MHz, CDCl_3_) *δ*: 169.3 (C=O), 163.5 (C, *J* = 249.4 Hz), 142.3 (CH), 138.0 (C), 136.7 (C), 133.5 (C), 129.6 (2CH, *J* = 7.9 Hz), 128.9 (4CH), 128.8 (CH), 128.5 (2CH), 127.4 (CH), 126.8 (C, *J* = 3.2 Hz), 126.3 (2CH), 115.7 (CH, *J* = 21.2 Hz), 90.5 (C), 43.4 (CH_2_); EIMS *m*/*z*: 359 (M+, 81), 197 (34), 106 (37), 105 (73), 91 (100), 77 (37); HREIMS: 359.1310 (calcd for C_23_H_18_NO_2_F 359.1322).

#### 2.2.12. 3-(4-fluorophenyl)-5-hydroxy-1-(4-methoxyphenyl)-5-phenyl-1*H*-pyrrol-2(5*H*)-one (**15**)

Following the general procedure, a mixture of (*E*)-4-fluorochalcone (50 mg, 0.21 mmol) and 4-methoxyphenyl isocyanide (60.7 mg, 0.42 mmol) in H_2_O (1 mL) was irradiated at 150 °C for 1 h and after purification on silica gel preparative-TLC (Hex/AcOEt, 4:1), 24.0 mg (29%) of compound **15** were obtained as a brown oil; ^1^H-NMR (600 MHz, CDCl_3_) *δ*: 8.00–8.03 (2H, m), 7.44 (2H, d, *J* = 8.9 Hz), 7.30–7.38 (4H, m), 7.27 (2H, dd, *J* = 8.9, 1.5 Hz), 7.16 (2H, t, *J* = 8.9 Hz), 6.87 (2H, d, *J* = 8.9 Hz), 5.68 (1H, s), 3.80 (3H, s); ^13^C-NMR (150 MHz, CDCl_3_) δ: 169.2 (C=O), 163.3 (C, *J* = 249.8 Hz), 157.1 (C), 140.4 (CH), 135.7 (C), 134.2 (C), 130.6 (C), 129.4 (2CH, *J* = 8.1 Hz), 129.3 (2CH), 128.6 (CH), 127.5 (C, *J* = 3.1 Hz), 127.2 (2CH), 124.1 (2CH), 115.6 (2CH, *J* = 21.2 Hz), 114.3 (2CH), 91.4 (C), 55.5 (CH_3_); EIMS *m*/*z*: 375 (M^+^, 2), 360 (44), 359 (100), 330 (71), 210 (80), 209 (31), 77 (23); HREIMS: 375.1267 (calcd for C_23_H_18_NO_3_F 375.1271).

#### 2.2.13. 1-cyclohexyl-3-(3-fluoro-4-methoxyphenyl)-5-hydroxy-5-phenyl-1*H*-pyrrol-2(5*H*)-one (**16**)

Following the general procedure, a mixture of (*E*)-3-fluoro-4-methoxychalcone (50 mg, 0.19 mmol) and cyclohexyl isocyanide (49.5 µL, 0.39 mmol) in H_2_O (1 mL) was irradiated at 150 °C for 1 h and after purification on silica gel preparative-TLC (Hex/AcOEt, 4:1), 33.4 mg (45%) of compound **16** were obtained as colorless oil; ^1^H-NMR (500 MHz, CDCl_3_) *δ*: 7.71 (1H, dd, *J* = 8.5, 1.2 Hz), 7.68 (1H, dd, *J* = 12.6, 2.1 Hz), 7.50–7.52 (2H, m), 7.32–7.38 (3H, m), 6.94 (1H, t, *J* = 8.5 Hz), 6.81 (1H, s), 3.90 (3H, s), 3.28 (1H, brs), 3.17–3.21 (1H, m), 2.16–2.19 (1H, m), 2.06–2.09 (1H, m), 1.61–1.75 (3H, m), 1.53 (1H, d, *J* = 12.2 Hz), 1.42 (1H, d, *J* = 12.2 Hz), 1.08–1.26 (2H, m), 0.93–1.03 (1H, m); ^13^C-NMR (150 MHz, CDCl_3_) *δ*: 168.6 (C=O), 152.2 (C, *J* = 245.2 Hz), 148.5 (C, *J* = 10.7 Hz), 140.9 (CH), 137.4 (C), 133.6 (C), 128.6 (2CH), 128.5 (2CH), 126.3 (2CH), 123.9 (C, *J* = 3.6 Hz), 115.4 (CH, *J* = 20.4 Hz), 113.2 (CH, *J* = 1.9 Hz), 90.5 (C), 56.4 (CH_3_), 53.2 (CH), 30.9 (CH_2_), 29.6 (CH_2_), 26.4 (2CH_2_), 25.4 (CH_2_); EIMS *m*/*z*: 381 (M^+^, 100), 299 (60), 283 (39), 221 (21), 126 (23), 105 (85), 77 (22); HREIMS: 381.1759 (calcd for C_23_H_24_NO_3_F 381.1740).

#### 2.2.14. 1-benzyl-3-(3-fluoro-4-methoxyphenyl)-5-hydroxy-5-phenyl-1*H*-pyrrol-2(5*H*)-one (**17**)

Following the general procedure, a mixture of (*E*)-3-fluoro-4-methoxychalcone (50 mg, 0.19 mmol) and benzyl isocyanide (48.5 µL, 0.39 mmol) in H_2_O (1 mL) was irradiated at 150 °C for 1 h and after purification on silica gel preparative-TLC (AcOEt/Hex, 4:1), 31.8 mg (42%) of compound **17** were obtained as an amorphous yellow solid; mp.: 187–188 °C; ^1^H-NMR (500 MHz, CDCl_3_) *δ*: 7.75 (1H, d, *J* = 8.4 Hz), 7.70 (1H, dd, *J* = 12.6, 2.0 Hz), 7.38–7.41 (2H, m), 7.33–7.37 (3H, m), 7.27–7.29 (2H, m), 7.19–7.25 (3H, m), 6.97 (1H, t, *J* = 8.6 Hz), 6.93 (1H, s), 4.78 (1H, d, *J* = 15.0 Hz), 3.99 (1H, d, *J* = 15.0 Hz), 3.91 (3H, s), 2.37 (1H, s); ^13^C-NMR (150 MHz, CDCl_3_) *δ*: 169.2 (C=O), 152.4 (C, *J* = 245.0 Hz), 148.7 (C, *J* = 10.9 Hz), 141.4 (CH), 138.1 (C), 136.8 (C), 133.3 (C), 128.9 (2CH), 128.8 (3CH), 128.6 (2CH), 127.4 (CH), 126.3 (2CH), 124.0 (CH, *J* = 3.4 Hz), 123.6 (C, *J* = 7.1 Hz), 115.4 (CH, *J* = 20.0 Hz), 113.2 (CH, *J* = 2.2 Hz), 90.6 (C), 56.4 (CH_3_), 43.4 (CH_2_); EIMS *m*/*z*: 389 (M^+^, 100), 227 (69), 126 (25), 106 (28), 105 (32), 91 (99), 77 (32); HREIMS: 389.1437 (calcd for C_24_H_20_NO_3_F 389.1427).

#### 2.2.15. 3-(3-fluoro-4-methoxyphenyl)-5-hydroxy-1-(4-methoxyphenyl)-5-phenyl-1*H*-pyrrol-2(5*H*)-one (**18**)

Following the general procedure, a mixture of (*E*)-3-fluoro-4-methoxychalcone (50 mg, 0.20 mmol) and 4-methoxyphenyl isocyanide (53.6 mg, 0.39 mmol) in H_2_O (1 mL) was irradiated at 150 °C for 1 h and after purification on silica gel preparative-TLC (Hex/AcOEt, 7:3), 20.5 mg (26%) of compound **18** were obtained as an amorphous brown solid; mp.: 166–168 °C; ^1^H-NMR (500 MHz, CDCl_3_) *δ*: 7.74 (1H, dt, *J* = 8.6, 1.4 Hz), 7.66 (1H, dd, *J* = 12.7, 2.1 Hz), 7.41 (2H, dd, *J* = 8.4, 1.6 Hz), 7.24–7.32 (5H, m), 6.98 (1H, s), 6.95 (1H, t, *J* = 8.6 Hz), 6.72 (2H, d, *J* = 9.0 Hz), 3.92 (3H, s), 3.72 (3H, s); ^13^C-NMR (125 MHz, CDCl_3_) *δ*: 168.7 (C=O), 157.8 (C), 152.2 (C, *J* = 245.2 Hz), 148.7 (C, *J* = 10.8 Hz), 141.5 (CH), 137.2 (C), 132.8 (C), 128.8 (CH), 128.7 (CH, *J* = 11.0 Hz), 128.6 (C), 126.8 (2CH), 126.2 (2CH), 124.0 (CH, *J* = 3.7 Hz), 123.5 (C, *J* = 7.3 Hz), 115.4 (CH, *J* = 20.1 Hz), 114.1 (2CH), 113.2 (2CH), 91.2 (C), 56.4 (CH_3_), 55.4 (CH_3_); EIMS *m*/*z*: 405 (M^+^, 59), 284 (27), 283 (100), 122 (12), 105 (71), 77 (16): HREIMS: 405.1355 (calcd for C_24_H_20_NO_4_F 405.1376).

#### 2.2.16. 3-(4-chlorophenyl)-1-cyclohexyl-5-hydroxy-5-phenyl-1*H*-pyrrol-2(5*H*)-one (**19**)

Following the general procedure, a mixture of (*E*)-4-chlorochalcone (50 mg, 0.21 mmol) and cyclohexyl isocyanide (52.3 µL, 0.41 mmol) in H_2_O (1 mL) was irradiated at 150 °C for 1 h and after purification on silica gel preparative-TLC (Hex/AcOEt, 4:1), 40.1 mg (53%) of compound **19** were obtained as an yellow oil; ^1^H-NMR (600 MHz, CDCl_3_) *δ*: 7.80 (2H, d, *J* = 8.7 Hz), 7.50 (2H, dd, *J* = 8.3, 1.8 Hz), 7.30–7.38 (5H, m), 6.86 (1H, s), 3.19–3.22 (1H, m), 3.05 (1H, s), 2.14–2.17 (1H, m), 2.04–2.08 (1H, m), 1.60–1.74 (3H, m), 1.53 (1H, d, *J* = 12.3 Hz), 1.42 (1H, d, *J* = 12.3 Hz), 1.07–1.26 (2H, m), 0.93–1.03 (1H, m); ^13^C-NMR (150 MHz, CDCl_3_) *δ*: 168.4 (C=O), 142.2 (CH), 137.1 (C), 135.2 (C), 134.1 (C), 129.2 (C), 129.0 (2CH), 128.8 (2CH), 128.7 (CH), 128.6 (2CH), 126.3 (2CH), 90.6 (C), 53.2 (CH), 30.9 (CH_2_), 29.6 (CH_2_), 26.3 (2CH_2_), 25.3 (CH_2_); EIMS *m*/*z*: 367 (M^+^, 83), 287 (23), 285 (68), 270 (19), 268 (55), 105 (100), 77 (28); HREIMS: 367.1334 (calcd for C_22_H_22_NO_2_^35^Cl 367.1339), 369.1303 (calcd for C_22_H_22_NO_2_^37^Cl 369.1310).

#### 2.2.17. 1-benzyl-3-(4-chlorophenyl)-5-hydroxy-5-phenyl-1*H*-pyrrol-2(5*H*)-one (**20**)

Following the general procedure, a mixture of (*E*)-4-chlorochalcone (50 mg, 0.21 mmol) and benzyl isocyanide (51.2 µL, 0.42 mmol) in H_2_O (1 mL) was irradiated at 150 °C for 1 h and after purification on silica gel preparative-TLC (AcOEt/Hex, 4:1), 42.5 mg (55%) of compound **20** were obtained as an amorphous white solid; mp.: 183–185 °C; ^1^H-NMR (500 MHz, CDCl_3_) δ: 7.86 (2H, d, J = 8.6 Hz), 7.32–7.41 (7H, m), 7.16–7.28 (5H, m), 7.00 (1H, s), 4.74 (1H, d, J = 15.0 Hz), 4.01 (1H, d, J = 15.0 Hz), 2.60 (1H, s); ^13^C-NMR (125 MHz, CDCl_3_) δ: 169.1 (C=O), 142.9 (CH), 137.9 (C), 136.5 (C), 135.4 (C), 133.4 (C), 129.1 (C), 129.0 (4CH), 128.9 (3CH), 128.8 (2CH), 128.5 (2CH), 127.4 (CH), 126.3 (2CH), 90.6 (C), 43.4 (CH_2_); EIMS *m*/*z*: 375 (M^+^, 72), 240 (14), 213 (14), 106 (23), 105 (16), 91 (100), 77 (15); HREIMS: 375.1036 (calcd for C_23_H_18_NO_2_^35^Cl 375.1026), 377.1012 (calcd for C_23_H_18_NO_2_^37^Cl 377.0997).

#### 2.2.18. 3-(4-chlorophenyl)-5-hydroxy-1-(4-methoxyphenyl)-5-phenyl-1*H*-pyrrol-2(5*H*)-one (**21**)

Following the general procedure, a mixture of (*E*)-4-chlorochalcone (50 mg, 0.21 mmol) and 4-methoxyphenyl isocyanide (56.6 mg, 0.41 mmol) in H_2_O (1 mL) was irradiated at 150 °C for 1 h and after purification on silica gel preparative-TLC (Hex/AcOEt, 4:1), 23.3 mg (29%) of compound **21** were obtained as an amorphous white solid; mp.: 163–165 °C; ^1^H-NMR (500 MHz, CDCl_3_) *δ*: 7.84 (2H, d, *J* = 8.7 Hz), 7.39–7.42 (2H, m), 7.34 (2H, d, *J* = 8.7 Hz), 7.23–7.32 (5H, m), 7.05 (1H, s), 6.71 (2H, d, *J* = 9.1 Hz), 3.70 (3H, s), 3.49 (1H, s); ^13^C-NMR (125 MHz, CDCl_3_) *δ*: 168.6 (C=O), 157.8 (C), 143.0 (CH), 137.0 (C), 135.5 (C), 133.2 (C), 129.1 (C), 129.0 (2CH), 128.9 (2CH), 128.8 (2CH), 128.7 (CH), 128.5 (C), 126.8 (2CH), 126.2 (2CH), 114.1 (2CH), 91.3 (C), 55.4 (CH_3_); EIMS *m*/*z*: 391 (M^+^, 38), 271 (35), 270 (19), 269 (100), 122 (42), 105 (56), 77 (16); HREIMS: 391.0967 (calcd for C_23_H_18_NO_3_^35^Cl 391.0975), 393.0838 (calcd for C_23_H_18_NO_3_^37^Cl 393.0946).

#### 2.2.19. 3-(4-bromophenyl)-1-cyclohexyl-5-hydroxy-5-phenyl-1*H*-pyrrol-2(5*H*)-one (**22**)

Following the general procedure, a mixture of (*E*)-4-bromochalcone (50 mg, 0.17 mmol) and cyclohexyl isocyanide (44.2 µL, 0.35 mmol) in H_2_O (1 mL) was irradiated at 150 °C for 1 h and after purification on silica gel preparative-TLC (Hex/AcOEt, 7:3), 41.5 mg (58%) of compound **22** were obtained as a colorless oil; ^1^H-NMR (500 MHz, CDCl_3_) *δ*: 7.76 (2H, d, *J* = 8.8 Hz), 7.46–7.52 (4H, m), 7.32–7.39 (3H, m), 6.89 (1H, d, *J* = 3.6 Hz), 3.19–3.22 (1H, m), 2.86 (1H, brs), 2.15–2.19 (1H, m), 2.05–2.09 (1H, m), 1.72 (1H, d, *J* = 12.7 Hz), 1.60–1.68 (2H, m), 1.53 (1H, d, *J* = 12.2 Hz), 1.42 (1H, d, *J* = 12.2 Hz), 1.07–1.26 (2H, m), 0.93–1.04 (1H, m); ^13^C-NMR (125 MHz, CDCl_3_) *δ*: 168.4 (C=O), 142.2 (CH), 137.0 (C), 134.2 (C), 131.8 (2CH), 129.7 (C), 129.2 (2CH), 128.8 (CH), 128.7 (2CH), 126.3 (2CH), 123.6 (C), 90.7 (C), 53.2 (CH), 30.9 (CH_2_), 29.6 (CH_2_), 26.3 (2CH_2_), 25.3 (CH_2_); EIMS *m*/*z*: 411 (M^+^, 55), 330 (33), 328 (36), 314 (35), 312 (36), 105 (100), 77 (29); HREIMS: 413.0811 (calcd for C_22_H_22_NO_2_^81^Br 413.0813), 411.0826 (calcd for C_22_H_22_NO_2_^79^Br 411.0834).

#### 2.2.20. 1-benzyl-3-(4-bromophenyl)-5-hydroxy-5-phenyl-1*H*-pyrrol-2(5*H*)-one (**23**)

Following the general procedure, a mixture of (*E*)-4-bromochalcone (50 mg, 0.17 mmol) and benzyl isocyanide (43.3 µL, 0.35 mmol) in H_2_O (1 mL) was irradiated at 150 °C for 1 h and after purification on silica gel preparative-TLC (AcOEt/Hex, 4:1), 37.2 mg (51%) of compound **23** were obtained as an amorphous yellow solid; mp.: 186–188 °C; ^1^H-NMR (500 MHz, CDCl_3_) *δ*: 7.81 (2H, d, *J* = 8.5 Hz), 7.51 (2H, d, *J* = 8.5 Hz), 7.33–7.41 (5H, m), 7.19–7.28 (5H, m), 7.03 (1H, s), 4.77 (1H, d, *J* = 15.0 Hz), 4.00 (1H, d, *J* = 15.0 Hz), 2.45 (1H, s); ^13^C-NMR (125 MHz, CDCl_3_) δ: 169.0 (C=O), 142.9 (CH), 138.0 (C), 136.5 (C), 133.6 (C), 131.9 (2CH), 129.5 (C), 129.2 (2CH), 129.0 (2CH), 128.9 (CH), 128.8 (2CH), 128.6 (2CH), 127.5 (CH), 126.3 (2CH), 123.8 (C), 90.6 (C), 43.5 (CH_2_); EIMS *m*/*z*: 419 (M^+^, 61), 405 (21), 404 (13), 403 (22), 105 (57), 91 (100), 77 (24); HREIMS: 419.0533 (calcd for C_23_H_18_NO_2_^79^Br 419.0521), 421.0498 (calcd for C_23_H_18_NO_2_^81^Br 421.0500).

#### 2.2.21. 3-(4-bromophenyl)-5-hydroxy-1-(4-methoxyphenyl)-5-phenyl-1*H*-pyrrol-2(5*H*)-one (**24**)

Following the general procedure, a mixture of (*E*)-4-bromochalcone (50 mg, 0.17 mmol) and 4-methoxyphenyl isocyanide (47.8 mg, 0.35 mmol) in H_2_O (1 mL) was irradiated at 150 °C for 1 h and after purification on silica gel preparative-TLC (Hex/AcOEt, 4:1), 21.2 mg (28%) of compound **24** were obtained as an amorphous white solid; mp.: 166–167 °C; ^1^H-NMR (500 MHz, CDCl_3_) *δ:* 7.75 (2H, d, *J* = 8.7 Hz), 7.50 (2H, d, *J* = 8.7 Hz), 7.39–7.42 (2H, m), 7.23–7.32 (5H, m), 7.07 (1H, s), 6.71 (2H, d, *J* = 9.1 Hz), 3.72 (3H, s), 3.49 (1H, s); ^13^C-NMR (125 MHz, CDCl_3_) *δ*: 168.5 (C=O), 157.8 (C), 143.0 (CH), 136.9 (C), 133.3 (C), 131.9 (2CH), 129.3 (C), 129.2 (2CH), 128.8 (2CH), 128.7 (CH), 128.5 (C), 126.7 (2CH), 126.2 (2CH), 123.8 (C), 114.1 (2CH), 91.3 (C), 55.4 (CH_3_); EIMS *m*/*z*: 435 (M^+^, 36), 315 (18), 314 (98), 312 (100), 122 (63), 104 (86), 77 (24); HREIMS: 435.0472 (calcd for C_23_H_18_NO_3_^79^Br 435.0470), 437.0484 (calcd for C_23_H_18_NO_3_^81^Br 437.0450).

#### 2.2.22. 3-(benzo[d][1,3]dioxol-5-yl)-1-cyclohexyl-5-hydroxy-5-phenyl-1*H*-pyrrol-2(5*H*)-one (**25**)

Following the general procedure, a mixture of (*E*)-3-(benzo[d][1,3]dioxol-5-yl)-1-phenylprop-2-en-1-one (50 mg, 0.20 mmol) and cyclohexyl isocyanide (50.3 µL, 0.40 mmol) in H_2_O (1 mL) was irradiated at 150 °C for 1 h and after purification on silica gel preparative-TLC (Hex/AcOEt, 7:3), 21.6 mg (29%) of compound **25** were obtained as a colorless oil; ^1^H-NMR (500 MHz, CDCl_3_) *δ*: 7.49–7.53 (3H, m), 7.30–7.40 (4H, m), 6.81 (1H, d, *J* = 8.2 Hz), 6.77 (1H, s), 5.96 (2H, s), 3.18–3.22 (1H, m), 3.13 (1H, s), 2.16–2.20 (1H, m), 2.07–2.11 (1H, m), 1.72 (1H, d, *J* = 11.7 Hz), 1.61–1.69 (2H, m), 1.53 (1H, d, *J* = 11.7 Hz), 1.41 (1H, d, J = 12.5 Hz), 1.07–1.26 (2H, m), 0.93–1.04 (1H, m); ^13^C-NMR (125 MHz, CDCl_3_) *δ*: 168.7 (C=O), 148.5 (C), 147.9 (C), 140.5 (CH), 137.6 (C), 134.6 (C), 128.6 (3CH), 126.4 (2CH), 124.9 (C), 122.0 (CH), 108.5 (CH), 107.9 (CH), 101.3 (CH_2_), 90.5 (C), 53.2 (CH), 30.8 (CH_2_), 29.6 (CH_2_), 26.4 (2CH_2_), 25.4 (CH_2_); EIMS *m*/*z*: 377 (M^+^, 78), 295 (41), 122 (24), 105 (62), 84 (100), 77 (25), 66 (87); HREIMS: 377.1635 (calcd for C_23_H_23_NO_4_ 377.1627).

#### 2.2.23. 3-(benzo[d][1,3]dioxol-5-yl)-1-benzyl-5-hydroxy-5-phenyl-1*H*-pyrrol-2(5*H*)-one (**26**)

Following the general procedure, a mixture of (*E*)-3-(benzo[*d*][1,3]dioxol-5-yl)-1-phenylprop-2-en-1-one (50 mg, 0.20 mmol) and benzyl isocyanide (49.3 µL, 0.40 mmol) in H_2_O (1 mL) was irradiated at 150 °C for 1 h and after purification on silica gel preparative-TLC (AcOEt/Hex, 4:1), 27.5 mg (36%) of compound **26** were obtained as a colorless oil; ^1^H-NMR (500 MHz, CDCl_3_) *δ*: 7.54 (1H, ddd, *J* = 8.1, 1.5, 0.7 Hz), 7.30–7.40 (6H, m), 7.15–7.27 (5H, m), 6.87 (1H, s), 6.82 (1H, d, *J* = 8.1 Hz), 5.97 (2H, s), 4.72 (1H, d, *J* = 15.0 Hz), 4.01 (1H, d, *J* = 15.0 Hz), 2.59 (1H, s); ^13^C-NMR (125 MHz, CDCl_3_) δ: 169.4 (C=O), 148.6 (C), 147.9 (C), 141.0 (CH), 138.1 (C), 136.9 (C), 134.0 (C), 128.9 (2CH), 128.8 (2CH), 128.7 (CH), 128.5 (2CH), 127.3 (CH), 126.3 (2CH), 124.6 (C), 122.1 (CH), 108.5 (CH), 107.8 (CH), 101.4 (CH_2_), 90.4 (C), 43.4 (CH_2_); EIMS *m*/*z*: 385 (M^+^, 100), 294 (14), 223 (41), 122 (16), 105 (16), 91 (52), 77 (13); HREIMS: 385.1308 (calcd for C_24_H_19_NO_4_ 385.1314).

#### 2.2.24. 3-(benzo[d][1,3]dioxol-5-yl)-5-hydroxy-1-(4-methoxyphenyl)-5-phenyl-1*H*-pyrrol-2(5*H*)-one (**27**)

Following the general procedure, a mixture of (E)-3-(benzo[d][1,3]dioxol-5-yl)-1-phenylprop-2-en-1-one (50 mg, 0.20 mmol) and 4-methoxyphenyl isocyanide (54.4 mg, 0.40 mmol) in H_2_O (1 mL) was irradiated at 150 °C for 1 h and after purification on silica gel preparative-TLC (Hex/AcOEt, 4:1), 15.1 mg (19%) of compound **27** were obtained as an amorphous brown solid; mp.: 157–159 °C; ^1^H-NMR (500 MHz, CDCl_3_) *δ*: 7.53 (1H, dd, *J* = 8.1, 1.7 Hz), 7.39–7.42 (2H, m), 7.35 (1H, d, *J* = 1.7 Hz), 7.23–7.31 (5H, m), 6.92 (1H, s), 6.82 (1H, d, *J* = 8.1 Hz), 6.71 (2H, d, *J* = 9.1 Hz), 5.98 (2H, s), 3.71 (3H, s), 3.44 (1H, brs); ^13^C-NMR (125 MHz, CDCl_3_) *δ*: 168.9 (C=O), 157.7 (C), 148.7 (C), 147.9 (C), 141.2 (CH), 137.4 (C), 133.7 (C), 128.8 (2CH), 128.7 (C), 128.6 (CH), 126.9 (2CH), 126.2 (2CH), 124.5 (C), 122.1 (CH), 114.1 (2CH), 108.5 (CH), 107.9 (CH), 101.4 (CH_2_), 91.2 (C), 55.4 (CH3); EIMS *m*/*z*: 401 (M^+^, 50), 280 (19), 279 (100), 265 (18), 105 (55), 77 (14); HREIMS: 401.1267 (calcd for C_24_H_19_NO_5_ 401.1263).

#### 2.2.25. 1-cyclohexyl-5-hydroxy-3-(4-methoxyphenyl)-5-phenyl-1*H*-pyrrol-2(5*H*)-one (**28**)

Following the general procedure, a mixture of (*E*)-4-methoxychalcone (50 mg, 0.21 mmol) and cyclohexyl isocyanide (53.2 µL, 0.42 mmol) in H_2_O (1 mL) was irradiated at 150 °C for 1 h and after purification on silica gel preparative-TLC (Hex/AcOEt, 4:1), 29.7 mg (39%) of compound **28** were obtained as a colorless oil; ^1^H-NMR (600 MHz, CDCl_3_) *δ*: 7.88 (2H, d, *J* = 8.9 Hz), 7.52 (2H, m), 7.29–7.38 (3H, m), 6.90 (2H, d, *J* = 8.9 Hz), 6.79 (1H, s), 3.82 (3H, s), 3.19–3.21 (1H, m), 2.52 (1H, s), 2.16–2.20 (1H, m), 2.08–2.11 (1H, m), 1.73 (1H, d, *J* = 11.2 Hz), 1.60–1.69 (2H, m), 1.53 (1H, d, *J* = 11.2 Hz), 1.42 (1H, d, *J* = 12.5 Hz), 1.08–1.26 (2H, m), 0.94–1.04 (1H, m); ^13^C-NMR (150 MHz, CDCl_3_) *δ:* 169.0 (C=O), 160.5 (C), 139.6 (CH), 137.5 (C), 134.8 (C), 129.1 (2CH), 128.6 (4CH), 126.4 (CH), 123.4 (C), 114.0 (2CH), 90.7 (C), 55.5 (CH_3_), 53.2 (CH), 31.0 (CH_2_), 29.6 (CH_2_), 26.4 (2CH_2_), 25.4 (CH_2_); EIMS *m*/*z*: 363 (M^+^, 98), 281 (67), 265 (38), 203 (27), 108 (59), 105 (100), 77 (49); HREIMS: 363.1832 (calcd for C_23_H_25_NO_3_ 363.1834).

#### 2.2.26. 1-benzyl-5-hydroxy-3-(4-methoxyphenyl)-5-phenyl-1*H*-pyrrol-2(5*H*)-one (**29**)

Following the general procedure, a mixture of (*E*)-4-methoxychalcone (50 mg, 0.21 mmol) and benzyl isocyanide (52.1 µL, 0.42 mmol) in H_2_O (1 mL) was irradiated at 150 °C for 1 h and after purification on silica gel preparative-TLC (Hex/AcOEt, 4:1), 24.9 mg (32%) of compound **29** were obtained as a brown oil; ^1^H-NMR (600 MHz, CDCl_3_) *δ*: 7.91 (2H, d, *J* = 8.9 Hz), 7.41 (2H, dd, *J* = 7.9, 1.2 Hz), 7.31–7.37 (3H, m), 7.28 (2H, d, *J* = 7.2 Hz), 7.18–7.24 (3H, m), 6.90–6.94 (3H, m), 4.78 (1H, d, J = 15.0 Hz), 3.99 (1H, d, J = 15.0 Hz), 3.83 (3H, s), 2.32 (1H, s); ^13^C-NMR (150 MHz, CDCl_3_) *δ*: 169.6 (C=O), 160.6 (C), 140.5 (CH), 138.3 (C), 137.1 (C), 134.1 (C), 129.1 (2CH), 128.9 (4CH), 128.7 (CH), 128.5 (2CH), 127.4 (CH), 126.3 (2CH), 123.3 (C), 114.2 (2CH), 90.6 (C), 55.5 (CH_3_), 43.4 (CH_2_); EIMS *m*/*z*: 371 (M^+^, 100), 209 (86), 135 (34), 108 (53), 105 (54), 91 (98), 77 (38); HREIMS: 371.1533 (calcd for C_24_H_21_NO_3_ 371.1521).

#### 2.2.27. 5-hydroxy-1,3-bis(4-methoxyphenyl)-5-phenyl-1*H*-pyrrol-2(5*H*)-one (**30**)

Following the general procedure, a mixture of (*E*)-4-methoxychalcone (50 mg, 0.21 mmol) and 4-methoxyphenyl isocyanide (57.6 mg, 0.42 mmol) in H_2_O (1 mL) was irradiated at 150 °C for 1 h and after purification on silica gel preparative-TLC (Hex/AcOEt, 4:1), 17.8 mg (22%) of compound **30** were obtained as a brown oil; ^1^H-NMR (600 MHz, CDCl_3_) *δ*: 7.90 (2H, d, *J* = 8.9 Hz), 7.43 (2H, d, *J* = 7.8 Hz), 7.26–7.32 (5H, m), 6.96 (1H, s), 6.92 (2H, d, *J* = 8.9 Hz), 6.74 (2H, d, *J* = 9.1 Hz), 3.84 (3H, s), 3.72 (3H, s), 3.21 (1H, brs); ^13^C-NMR (150 MHz, CDCl_3_) *δ*: 169.0 (C=O), 160.5 (C), 157.7 (C), 140.4 (CH), 137.5 (C), 133.8 (C), 129.0 (2CH), 128.6 (C), 128.5 (2CH), 128.4 (CH), 126.9 (2CH), 126.1 (2CH), 123.0 (C), 114.0 (2CH), 113.9 (2CH), 91.1 (C), 55.4 (CH_3_), 55.3 (CH_3_); EIMS *m*/*z*: 387 (M^+^, 50), 266 (35), 265 (96), 210 (19), 135 (29), 105 (100), 77 (50); HREIMS: 387.1471 (calcd for C_24_H_21_NO_4_ 387.1471).

#### 2.2.28. 1-cyclohexyl-3-(3,4-dimethylphenyl)-5-hydroxy-5-phenyl-1*H*-pyrrol-2(5*H*)-one (**31**)

Following the general procedure, a mixture of (E)-3,4-dimethylchalcone (50 mg, 0.21 mmol) and cyclohexyl isocyanide (53.7 µL, 0.42 mmol) in H_2_O (1 mL) was irradiated at 150 °C for 1 h and after purification on silica gel preparative-TLC (Hex/AcOEt, 4:1), 24.4 mg (32%) of compound **31** were obtained as a brown oil; ^1^H-NMR (500 MHz, CDCl_3_) *δ*: 7.72 (1H, s), 7.60 (1H, d, *J* = 7.8 Hz), 7.50–7.53 (2H, m), 7.30–7.38 (3H, m), 1.07 (1H, d, *J* = 7.8 Hz), 6.83 (1H, s), 3.17–3.20 (1H, m), 2.57 (1H, s), 2.27 (6H, s), 2.06–2.24 (2H, m), 1.73 (1H, d, *J* = 12.1 Hz), 1.60–1.69 (2H, m), 1.53 (1H, d, *J* = 12.1 Hz), 1.41 (1H, d, *J* = 12.1 Hz), 1.08–1.23 (2H, m), 0.93–1.03 (1H, m); ^13^C-NMR (125 MHz, CDCl_3_) *δ:* 168.8 (C=O), 140.7 (CH), 137.9 (C), 137.3 (C), 136.7 (C), 135.2 (C), 129.7 (CH), 128.7 (CH), 128.4 (3CH), 128.2 (C), 126.2 (2CH), 125.0 (CH), 90.5 (C), 53.0 (CH), 30.8 (CH_2_), 29.5 (CH_2_), 26.3 (2CH_2_), 25.3 (CH_2_), 19.8 (CH_3_), 19.7 (CH_3_); EIMS *m*/*z*: 361 (M^+^, 93), 279 (89), 237 (55), 201 (24), 106 (59), 105 (100), 77 (41); HREIMS: 361.2037 (calcd for C_24_H_27_NO_2_ 361.2042).

#### 2.2.29. 1-benzyl-3-(3,4-dimethylphenyl)-5-hydroxy-5-phenyl-1*H*-pyrrol-2(5*H*)-one (**32**)

Following the general procedure, a mixture of (*E*)-3,4-dimethylchalcone (50 mg, 0.21 mmol) and benzyl isocyanide (52.6 µL, 0.42 mmol) in H_2_O (1 mL) was irradiated at 150 °C for 1 h and after purification on silica gel preparative-TLC (Hex/AcOEt, 4:1), 29.6 mg (38%) of compound **32** were obtained as a yellow oil; ^1^H-NMR (600 MHz, CDCl_3_) *δ*: 7.71 (1H, d, *J* = 1.4 Hz), 7.65 (1H, dd, *J* = 7.8, 1.8 Hz), 7.38–7.41 (2H, m), 7.31–7.36 (3H, m), 7.27 (2H, d, *J* = 8.1 Hz), 7.17–7.23 (3H, m), 7.15 (1H, d, *J* = 7.8 Hz), 6.95 (1H, s), 4.75 (1H, d, *J* = 15.0 Hz), 4.00 (1H, d, *J* = 15.0 Hz), 2.47 (1H, brs), 2.28 (6H, s); ^13^C-NMR (150 MHz, CDCl_3_) *δ:* 169.4 (C=O), 141.6 (CH), 138.2 (C), 138.1 (C), 136.9 (C), 136.7 (C), 134.6 (C), 129.8 (CH), 128.8 (2CH), 128.7 (2CH), 128.6 (2CH), 128.4 (2CH), 128.0 (C), 127.2 (CH), 126.2 (2CH), 125.0 (CH), 90.4 (C), 43.2 (CH_2_), 19.8 (CH_3_), 19.7 (CH_3_); EIMS *m*/*z*: 369 (M^+^, 90), 237 (47), 207 (41), 106 (72), 105 (73), 91 (100), 77 (40); HREIMS: 369.1724 (calcd for C_25_H_23_NO_2_ 369.1729).

#### 2.2.30. 3-(3,4-dimethylphenyl)-5-hydroxy-1-(4-methoxyphenyl)-5-phenyl-1*H*-pyrrol-2(5*H*)-one (**33**)

Following the general procedure, a mixture of (*E*)-3,4-dimethylchalcone (50 mg, 0.21 mmol) and 4-methoxyphenyl isocyanide (58.1 mg, 0.42 mmol) in H_2_O (1 mL) was irradiated at 150 °C for 1 h and after purification on silica gel preparative-TLC (Hex/AcOEt, 7:3), 18.7 mg (23%) of compound **33** were obtained as a brown oil; ^1^H-NMR (500 MHz, CDCl_3_) *δ*: 7.64–7.67 (2H, m), 7.40–7.43 (2H, m), 7.23–7.31 (5H, m), 7.15 (1H, d, *J* = 7.8 Hz), 6.97 (1H, s), 6.71 (2H, d, *J* = 9.1 Hz), 3.71 (3H, s), 3.38 (1H, brs), 2.28 (3H, s), 2.26 (3H, s); ^13^C-NMR (125 MHz, CDCl_3_) *δ*: 168.9 (C=O), 157.6 (C), 141.6 (CH), 138.1 (C), 137.4 (C), 136.7 (C), 134.2 (C), 129.8 (CH), 128.7 (CH), 128.6 (C), 128.5 (2CH), 128.4 (CH), 127.9 (C), 126.8 (2CH), 126.1 (2CH), 125.1 (CH), 113.9 (2CH), 91.1 (C), 55.3 (CH_3_), 19.8 (CH_3_), 19.7 (CH_3_); EIMS *m*/*z*: 385 (M^+^, 70), 369 (44), 264 (48), 263 (100), 210 (27), 105 (90), 77 (29); HREIMS: 385.1689 (calcd for C_25_H_23_NO_3_ 385.1678).

#### 2.2.31. 1-cyclohexyl-5-hydroxy-3-(4-nitrophenyl)-5-phenyl-1*H*-pyrrol-2(5*H*)-one (**34**)

Following the general procedure, a mixture of (*E*)-4-nitrochalcone (50 mg, 0.20 mmol) and cyclohexyl isocyanide (50.1 µL, 0.40 mmol) in H_2_O (1 mL) was irradiated at 150 °C for 1 h after and purification on silica gel preparative-TLC (Hex/AcOEt, 4:1), 27.6 mg (37%) of compound **34** were obtained as an orange oil; ^1^H-NMR (500 MHz, CDCl_3_) *δ*: 8.22 (2H, d, *J* = 8.8 Hz), 8.08 (2H, d, *J* = 8.8 Hz), 7.50–7.53 (2H, m), 7.36–7.42 (3H, m), 7.10 (1H, s), 3.21–3.25 (1H, m), 2.77 (1H, s), 2.17–2.21 (1H, m), 2.07–2.11 (1H, m), 1.74 (1H, d, *J* = 12.1 Hz), 1.61–1.71 (2H, m), 1.55 (1H, d, *J* = 12.1 Hz), 1.45 (1H, d, *J* = 12.7 Hz), 1.09–1.25 (2H, m), 0.95–1.05 (1H, m); ^13^C-NMR (125 MHz, CDCl_3_) *δ*: 167.7 (C=O), 147.9 (C), 144.9 (CH), 137.0 (C), 136.2 (C), 133.3 (C), 128.9 (CH), 128.7 (2CH), 128.4 (2CH), 126.2 (2CH), 123.7 (2CH), 90.6 (C), 53.3 (CH), 30.8 (CH_2_), 29.5 (CH_2_), 26.2 (2CH_2_), 25.2 (CH_2_); EIMS *m*/*z*: 378 (M^+^, 100), 296 (70), 281 (25), 280 (93), 105 (75), 98 (22), 77 (21); HREIMS: 378.1562 (calcd for C_22_H_22_N_2_O_4_ 378.1580).

#### 2.2.32. 1-benzyl-5-hydroxy-3-(4-nitrophenyl)-5-phenyl-1*H*-pyrrol-2(5*H*)-one (**35**)

Following the general procedure, a mixture of (*E*)-4-nitrochalcone (50 mg, 0.20 mmol) and benzyl isocyanide (49.1 µL, 0.40 mmol) in H_2_O (1 mL) was irradiated at 150 °C for 1 h and after purification on silica gel preparative-TLC (Hex/AcOEt, 4:1), 27.4 mg (36%) of compound **35** were obtained as an orange oil; ^1^H-NMR (500 MHz, CDCl_3_) *δ*: 8.25 (2H, d, *J* = 9.0 Hz), 8.12 (2H, d, *J* = 9.0 Hz), 7.36–7.42 (5H, m), 7.20–7.30 (6H, m), 4.83 (1H, d, *J* = 15.0 Hz), 4.00 (1H, d, *J* = 15.0 Hz), 2.43 (1H, s); ^13^C-NMR (125 MHz, CDCl_3_) *δ*: 168.2 (C=O), 145.7 (CH), 137.6 (C), 136.7 (C), 135.8 (C), 132.6 (C), 129.1 (CH), 129.0 (2CH), 128.8 (2CH), 128.5 (2CH), 128.4 (2CH), 127.6 (CH), 126.1 (2CH), 123.8 (2CH), 120.2 (C), 90.6 (C), 43.5 (CH_2_); EIMS *m*/*z*: 386 (M^+^, 31), 308 (12), 150 (14), 106 (41), 105 (39), 91 (100), 77 (24); HREIMS: 386.1261 (calcd for C_23_H_18_N_2_O_4_ 386.1267).

#### 2.2.33. 1-cyclohexyl-5-hydroxy-5-(3-methoxyphenyl)-3-(3-nitrophenyl)-1*H*-pyrrol-2(5*H*)-one (**36**)

Following the general procedure, a mixture of (*E*)-3′methoxy-3-nitrochalcone (50 mg, 0.17 mmol) and cyclohexyl isocyanide (44.8 µL, 0.34 mmol) in H_2_O (1 mL) was irradiated at 150 °C for 1 h and after purification on silica gel preparative-TLC (Hex/AcOEt, 4:1), 30.2 mg (42%) of compound **36** were obtained as a yellow oil; ^1^H-NMR (500 MHz, CDCl_3_) *δ*: 8.67 (1H, s), 8.25 (1H, d, *J* = 7.7 Hz), 8.17 (1H, d, *J* = 8.1 Hz), 7.54 (1H, t, *J* = 8.1 Hz), 7.29 (1H, t, J = 8.1 Hz), 7.10–7.12 (1H, m), 7.03–7.06 (2H, m), 6.89 (1H, dd, *J* = 8.1, 2.5 Hz), 3.82 (3H, s), 3.21–3.26 (1H, m), 3.11 (1H, brs), 2.17–2.22 (1H, m), 2.08–2.12 (1H, m), 1.63–1.76 (3H, m), 1.47–1.57 (2H, m), 1.10–1.22 (2H, m), 0.96–1.07 (1H, m); ^13^C-NMR (125 MHz, CDCl_3_) *δ*: 167.8 (C=O), 159.9 (C), 148.3 (C), 143.8 (CH), 138.2 (C), 133.5 (CH), 133.0 (C), 132.3 (C), 129.7 (CH), 129.5 (CH), 123.6 (CH), 122.5 (CH), 118.4 (CH), 114.1 (CH), 112.1 (CH), 90.6 (C), 55.4 (CH3), 53.3 (CH), 30.8 (CH_2_), 29.5 (CH_2_), 26.2 (CH_2_), 26.1 (CH_2_), 25.2 (CH2); EIMS *m*/*z*: 408 (M^+^, 71), 326 (41), 311 (42), 310 (100), 135 (80), 107 (21), 98 (59); HREIMS: 408.1674 (calcd for C_23_H_24_N_2_O_5_ 408.1685).

#### 2.2.34. 1-benzyl-5-hydroxy-5-(3-methoxyphenyl)-3-(3-nitrophenyl)-1*H*-pyrrol-2(5*H*)-one (**37**)

Following the general procedure, a mixture of (*E*)-3′methoxy-3-nitrochalcone (50 mg, 0.17 mmol) and benzyl isocyanide (43.9 µL, 0.34 mmol) in H_2_O (1 mL) was irradiated at 150 °C for 1 h and after purification on silica gel preparative-TLC (Hex/AcOEt, 4:1), 35.3 mg (48%) of compound **37** were obtained as a yellow oil; ^1^H-NMR (500 MHz, CDCl_3_) *δ*: 8.73 (1H, t, *J* = 1.9 Hz), 8.33 (1H, ddd, *J* = 7.8, 1.6, 1.0 Hz), 8.21 (1H, ddd, *J* = 8.2, 2.6, 1.0 Hz), 7.58 (1H, t, *J* = 8.2 Hz), 7.21–7.38 (6H, m), 7.19 (1H, s), 6.95–6.98 (2H, m), 6.89 (1H, ddd, *J* = 8.2, 2.6, 1.0 Hz), 4.82 (1H, d, *J* = 15.0 Hz), 4.06 (1H, d, *J* = 15.0 Hz), 3.77 (3H, s), 2.51 (1H, s); ^13^C-NMR (125 MHz, CDCl_3_) *δ*: 168.3 (C=O), 160.2 (C), 148.4 (C), 144.6 (CH), 137.6 (C), 137.5 (C), 133.4 (CH), 132.4 (C), 132.1 (C), 130.1 (CH), 129.6 (CH), 128.8 (2CH), 128.5 (2CH), 127.5 (CH), 123.8 (CH), 122.4 (CH), 118.2 (CH), 114.4 (CH), 112.0 (CH), 90.5 (C), 55.4 (CH_3_), 43.5 (CH_2_); EIMS *m*/*z*: 416 (M^+^, 20), 256 (24), 241 (50), 135 (84), 107 (24), 106 (43), 91 (100); HREIMS: 416.1361 (calcd for C_24_H_20_N_2_O_5_ 416.1372).

#### 2.2.35. 5-hydroxy-5-(3-methoxyphenyl)-1-(4-methoxyphenyl)-3-(3-nitrophenyl)-1*H*-pyrrol-2(5*H*)-one (**38**)

Following the general procedure, a mixture of (*E*)-3′-methoxy-3-nitrochalcone (50 mg, 0.17 mmol) and 4-methoxyphenyl isocyanide (48.5 mg, 0.34 mmol) in H_2_O (1 mL) was irradiated at 150 °C for 1 h and after purification on silica gel preparative-TLC (Hex/AcOEt, 4:1), 19.2 mg (25%) of compound **38** were obtained as a brown oil; ^1^H-NMR (500 MHz, CDCl_3_) *δ*: 8.66 (1H, s), 8.26 (1H, d, *J* = 7.6 Hz), 8.17 (1H, d, *J* = 8.2 Hz), 7.54 (1H, t, *J* = 8.2 Hz), 7.30 (2H, d, *J* = 8.8 Hz), 7.22 (2H, t, *J* = 7.6 Hz), 7.00 (1H, s), 6.96 (1H, d, *J* = 7.6 Hz), 6.81 (1H, d, *J* = 8.2 Hz), 6.72 (2H, d, *J* = 8.8 Hz), 3.87 (1H, brs), 3.74 (3H, s), 3.72 (3H, s); ^13^C-NMR (125 MHz, CDCl_3_) *δ*: 167.9 (C=O), 160.0 (C), 157.7 (C), 148.3 (C), 144.8 (CH), 138.1 (C), 133.4 (CH), 132.0 (C), 131.9 (C), 129.9 (CH), 129.6 (CH), 128.2 (C), 126.3 (2CH), 123.7 (CH), 122.4 (CH), 118.3 (CH), 114.1 (CH), 114.0 (2CH), 112.1 (CH), 91.2 (C), 55.4 (CH_3_), 55.3 (CH_3_); EIMS *m*/*z*: 432 (M^+^, 28), 416 (33), 310 (45), 240 (17), 135 (33), 123 (13), 122 (100); HREIMS: 432.1314 (calcd for C_24_H_20_N_2_O_6_ 432.1321).

#### 2.2.36. 1-cyclohexyl-5-hydroxy-3-(4-methoxyphenyl)-5-(3-nitrophenyl)-1*H*-pyrrol-2(5*H*)-one (**39**)

Following the general procedure, a mixture of (E)-3′-nitro-4-methoxychalcone (50 mg, 0.17 mmol) and cyclohexyl isocyanide (44.8 µL, 0.34 mmol) in H_2_O (1 mL) was irradiated at 150 °C for 1 h and after purification on silica gel preparative-TLC (Hex/AcOEt, 4:1), 33.8 mg (47%) of compound **39** were obtained as an orange oil; ^1^H-NMR (500 MHz, CDCl_3_) *δ*: 8.48 (1H, s), 8.18 (1H, d, *J* = 8.0 Hz), 7.83 (2H, d, *J* = 8.8 Hz), 7.73 (1H, d, *J* = 8.0 Hz), 7.51 (1H, t, *J* = 8.0 Hz), 6.88 (2H, d, *J* = 8.8 Hz), 6.69 (1H, s), 3.81 (3H, s), 3.46 (1H, brs), 3.15–3.19 (1H, m), 2.16–2.21 (1H, m), 2.01–2.06 (1H, m), 1.69–1.75 (1H, m), 1.64 (2H, d, *J* = 12.2 Hz), 1.53 (1H, d, *J* = 12.2 Hz), 1.38 (1H, d, *J* = 12.2 Hz), 1.06–1.22 (2H, m), 0.93–1.03 (1H, m); ^13^C-NMR (125 MHz, CDCl_3_) *δ*: 168.5 (C=O), 160.6 (C), 148.5 (C), 140.6 (C), 138.6 (CH), 135.3 (C), 132.5 (CH), 129.4 (CH), 129.1 (2CH), 123.6 (CH), 122.8 (C), 121.7 (CH), 114.0 (2CH), 89.7 (C), 55.4 (CH_3_), 53.2 (CH), 30.7 (CH_2_), 29.7 (CH_2_), 26.2 (CH_2_), 26.1 (CH_2_), 25.2 (CH_2_); EIMS *m*/*z*: 408 (M^+^, 99), 326 (54), 310 (64), 309 (56), 238 (33), 150 (100), 108 (30); HREIMS: 408.1668 (calcd for C_23_H_24_N_2_O_5_ 408.1685).

#### 2.2.37. 1-benzyl-5-hydroxy-3-(4-methoxyphenyl)-5-(3-nitrophenyl)-1*H*-pyrrol-2(5*H*)-one (**40**)

Following the general procedure, a mixture of (*E*)-3′-nitro-4-methoxychalcone (50 mg, 0.17 mmol) and benzyl isocyanide (43.9 µL, 0.34 mmol) in H_2_O (1 mL) was irradiated at 150 °C for 1 h and after purification on silica gel preparative-TLC (Hex/AcOEt, 4:1), 34.5 mg (47%) of compound **40** were obtained as an amorphous orange solid; mp.: 181–182 °C; ^1^H-NMR (500 MHz, CDCl_3_) *δ*: 8.18 (1H, t, *J* = 1.9 Hz), 8.03 (1H, ddd, *J* = 8.2, 2.3, 1.0 Hz), 7.88 (2H, d, *J* = 8.9 Hz), 7.56 (1H, dd, *J* = 7.8, 1.0 Hz), 7.33 (1H, t, *J* = 8.0 Hz), 7.05–7.12 (5H, m), 6.90 (2H, d, *J* = 8.9 Hz), 6.85 (1H, s), 4.43 (1H, d, *J* = 15.0 Hz), 4.39 1H, d, *J* = 15.0 Hz), 3.83 (3H, s), 3.47 (1H, s); ^13^C-NMR (125 MHz, CDCl_3_) *δ*: 169.5 (C=O), 160.7 (C), 148.3 (C), 139.5 (C), 139.1 (CH), 137.3 (C), 134.6 (C), 132.3 (CH), 129.3 (CH), 129.0 (2CH), 128.7 (2CH), 128.3 (2CH), 127.2 (CH), 123.3 (CH), 122.5 (C), 122.0 (CH), 114.0 (2CH), 89.2 (C), 55.4 (CH_3_), 42.8 (CH_2_); EIMS *m*/*z*: 416 (M^+^, 99), 400 (91), 325 (43), 309 (73), 254 (90), 133 (53), 91 (100); HREIMS: 416.1361 (calcd for C_24_H_20_N_2_O_5_ 416.1372).

#### 2.2.38. 5-hydroxy-1,3-bis(4-methoxyphenyl)-5-(3-nitrophenyl)-1*H*-pyrrol-2(5*H*)-one (**41**)

Following the general procedure, a mixture of (*E*)-3′-nitro-4-methoxychalcone (50 mg, 0.17 mmol) and 4-methoxyphenyl isocyanide (48.5 mg, 0.34 mmol) in H_2_O (1 mL) was irradiated at 150 °C for 1 h and after purification on silica gel preparative-TLC (Hex/AcOEt, 4:1), 22.1 mg (29%) of compound **41** were obtained as an amorphous brown solid; mp.: 186–188 °C; ^1^H-NMR (500 MHz, CDCl_3_) *δ*: 8.33 (1H, t, *J* = 1.9 Hz), 8.08 (1H, ddd, *J* = 8.0, 2.3, 1.0 Hz), 7.82 (2H, d, *J* = 8.9 Hz), 7.63 (1H, dd, *J* = 7.8, 1.0 Hz), 7.41 (1H, t, *J* = 8.0 Hz), 7.20 (2H, d, *J* = 9.1 Hz), 6.85–6.90 (3H, m), 6.67 (2H, d, *J* = 9.1 Hz), 4.28 (1H, brs), 3.83 (3H, s), 3.70 (3H, s); ^13^C-NMR (125 MHz, CDCl_3_) *δ*: 169.0 (C=O), 160.7 (C), 157.8 (C), 148.5 (C), 140.0 (C), 139.3 (CH), 134.3 (C), 132.3 (CH), 129.5 (CH), 129.0 (2CH), 127.9 (C), 126.8 (2CH), 123.5 (CH), 122.4 (C), 121.7 (CH), 114.1 (2CH), 114.0 (2CH), 90.4 (C), 55.4 (CH_3_), 55.2 (CH_3_); EIMS *m*/*z*: 432 (M^+^, 41), 311 (20), 310 (100), 150 (88), 135 (15), 122 (59), 104 (17); HREIMS: 432.1342 (calcd for C_24_H_20_N_2_O_6_ 432.1321).

#### 2.2.39. 1-cyclohexyl-5-hydroxy-3-(4-methoxyphenyl)-5-(4-nitrophenyl)-1*H*-pyrrol-2(5*H*)-one (**42**)

Following the general procedure, a mixture of (*E*)-4′-nitro-4-methoxychalcone (50 mg, 0.18 mmol) and cyclohexyl isocyanide (44.8 µL, 0.35 mmol) in H_2_O (1 mL) was irradiated at 150 °C for 1 h and after purification on silica gel preparative-TLC (Hex/AcOEt, 4:1), 38.8 mg (54%) of compound **42** were obtained as an amorphous orange solid; mp.: 159–161 °C; ^1^H-NMR (500 MHz, CDCl_3_) *δ*: 8.19 (2H, d, *J* = 9.0 Hz), 7.84 (2H, d, *J* = 9.0 Hz), 7.69 (2H, d, *J* = 9.0 Hz), 6.89 (2H, d, *J* = 9.0 Hz), 6.70 (1H, s), 3.82 (3H, s), 3.16–3.24 (2H, m), 2.14–2.18 (1H, m), 2.00–2.04 (1H, m), 1.72 (1H, d, *J* = 12.7 Hz), 1.60–1.69 (2H, m), 1.54 (1H, d, *J* = 12.3 Hz), 1.39 (1H, d, *J* = 12.7 Hz), 1.07–1.27 (2H, m), 0.93–1.04 (1H, m); ^13^C-NMR (125 MHz, CDCl_3_) *δ*: 168.7 (C=O), 160.6 (C), 147.9 (C), 145.2 (C), 138.4 (CH), 135.4 (C), 129.1 (2CH), 127.5 (2CH), 123.7 (2CH), 122.7 (C), 114.0 (2CH), 89.9 (C), 55.4 (CH_3_), 53.3 (CH), 30.8 (CH_2_), 29.7 (CH_2_), 26.2 (CH_2_), 26.1 (CH_2_), 25.2 (CH_2_); EIMS *m*/*z* 408 (M^+^, 100), 392 (22), 327 (25), 326 (83), 310 (44), 309 (18), 283 (16), 149 (74), 108 (48), 104 (22), 98 (18), 55 (14); HREIMS: 408.1697 (calcd for C_23_H_24_N_2_O_5_ 408.1685).

#### 2.2.40. 1-benzyl-5-hydroxy-3-(4-methoxyphenyl)-5-(4-nitrophenyl)-1*H*-pyrrol-2(5*H*)-one (**43**)

Following the general procedure, a mixture of (*E*)-4′-nitro-4-methoxychalcone (50 mg, 0.18 mmol) and benzyl isocyanide (43.9 µL, 0.35 mmol) in H_2_O (1 mL) was irradiated at 150 °C for 1 h and after purification on silica gel preparative-TLC (Hex/AcOEt, 4:1), 40.4 mg (55%) of compound **43** were obtained as an orange oil; ^1^H-NMR (500 MHz, CDCl_3_) *δ*: 8.05 (2H, d, *J* = 8.8 Hz), 7.88 (2H, d, *J* = 8.8 Hz), 7.49 (2H, d, *J* = 8.8 Hz), 7.10–7.16 (5H, m), 6.91 (2H, d, *J* = 8.8 Hz), 6.86 (1H, s), 4.51 (1H, d, *J* = 15.0 Hz), 4.28 (1H, d, *J* = 15.0 Hz), 3.83 (3H, s), 3.26 (1H, s); ^13^C-NMR (125 MHz, CDCl_3_) *δ*: 169.4 (C=O), 160.8 (C), 147.8 (C), 144.4 (C), 139.1 (CH), 137.4 (C), 134.7 (C), 129.0 (2CH), 128.7 (2CH), 128.3 (2CH), 127.5 (2CH), 127.4 (CH), 123.6 (2CH), 122.5 (C), 114.1 (2CH), 89.5 (C), 55.4 (CH_3_), 43.0 (CH_2_); EIMS *m*/*z*: 416 (M^+^, 100), 398 (25), 325 (46), 254 (91), 108 (27), 106 (30), 91 (100); HREIMS: 416.1380 (calcd for C_24_H_20_N_2_O_5_ 416.1372).

#### 2.2.41. 5-hydroxy-1,3-bis(4-methoxyphenyl)-5-(4-nitrophenyl)-1*H*-pyrrol-2(5*H*)-one (**44**)

Following the general procedure, a mixture of (*E*)-4′-nitro-4-methoxychalcone (50 mg, 0.18 mmol) and 4-methoxyphenyl isocyanide (48.5 mg, 0.35 mmol) in H_2_O (1 mL) was irradiated at 150 °C for 1 h and after purification on silica gel preparative-TLC (Hex/AcOEt, 4:1), 21.4 mg (28%) of compound **44** were obtained as a brown oil; ^1^H-NMR (500 MHz, CDCl_3_) *δ*: 8.11 (2H, d, *J* = 8.8 Hz), 7.86 (2H, d, *J* = 8.8 Hz), 7.57 (2H, d, *J* = 8.8 Hz), 7.21 (2H, d, *J* = 9.0 Hz), 6.88–6.92 (3H, m), 6.70 (2H, d, *J* = 9.0 Hz), 3.84 (4H, s), 3.71 (3H, s); ^13^C-NMR (125 MHz, CDCl_3_) *δ*: 168.7 (C=O), 160.8 (C), 157.9 (C), 147.8 (C), 144.7 (C), 139.0 (CH), 134.6 (C), 129.1 (2CH), 128.0 (C), 127.4 (2CH), 126.7 (2CH), 123.8 (2CH), 122.3 (C), 114.1 (4CH), 90.5 (C), 55.4 (CH_3_), 55.3 (CH_3_); EIMS *m*/*z*: 432 (M^+^, 77), 311 (22), 310 (100), 150 (63), 122 (45), 103 (13); HREIMS: 432.1339 (calcd for C_24_H_20_N_2_O_6_ 432.1321).

#### 2.2.42. 1-cyclohexyl-5-hydroxy-3-(3-methoxyphenyl)-5-(4-nitrophenyl)-1*H*-pyrrol-2(5*H*)-one (**45**)

Following the general procedure, a mixture of (*E*)-4′-nitro-3-methoxychalcone (50 mg, 0.17 mmol) and cyclohexyl isocyanide (44.8 µL, 0.34 mmol) in H_2_O (1 mL) was irradiated at 150 °C for 1 h and after purification on silica gel preparative-TLC (Hex/AcOEt, 4:1), 37.4 mg (52%) of compound **45** were obtained as an amorphous brown solid; mp.: 134–136 °C; ^1^H-NMR (500 MHz, CDCl_3_) *δ*: 8.19 (2H, d, *J* = 8.8 Hz), 7.68 (2H, d, *J* = 8.8 Hz), 7.47 (1H, s), 7.33 (1H, d, *J* = 7.6 Hz), 7.25–7.29 (1H, m), 6.92 (1H, dd, *J* = 8.0, 1.7 Hz), 6.72 (1H, s), 3.84 (3H, s), 3.74 (1H, brs), 3.16–3.20 (1H, m), 2.12–2.16 (1H, m), 2.00–2.04 (1H, m), 1.72 (1H, d, *J* = 12.5 Hz), 1.64 (2H, d, *J* = 12.5 Hz), 1.53 (1H, d, *J* = 12.1 Hz), 1.39 (1H, d, *J* = 12.1Hz), 1.06–1.26 (2H, m), 0.93–1.03 (1H, m); ^13^C-NMR (125 MHz, CDCl_3_) *δ*: 168.4 (C=O), 159.6 (C), 147.9 (C), 145.0 (C), 141.1 (CH), 135.7 (C), 131.5 (C), 129.6 (CH), 127.5 (2CH), 123.7 (2CH), 120.0 (CH), 115.5 (CH), 112.9 (CH), 89.8 (C), 55.4 (CH_3_), 53.4 (CH), 30.7 (CH_2_), 29.7 (CH_2_), 26.2 (CH_2_), 26.1 (CH_2_), 25.1 (CH_2_); EIMS *m*/*z*: 408 (M^+^, 100), 326 (79), 311 (31), 310 (84), 150 (86), 104 (26), 98 (40). HREIMS: 408.1684 (calcd for C_23_H_24_N_2_O_5_ 408.1685).

#### 2.2.43. 1-benzyl-5-hydroxy-3-(3-methoxyphenyl)-5-(4-nitrophenyl)-1*H*-pyrrol-2(5*H*)-one (**46**)

Following the general procedure, a mixture of (*E*)-4′-nitro-3-methoxychalcone (50 mg, 0.17 mmol) and benzyl isocyanide (43.9 µL, 0.34 mmol) in H_2_O (1 mL) was irradiated at 150 °C for 1 h and after purification on silica gel preparative-TLC (Hex/AcOEt, 4:1), 36.0 mg (49%) of compound **46** were obtained as an amorphous orange solid; mp.: 186–187 °C; ^1^H-NMR (500 MHz, CDCl_3_) *δ*: 8.07 (2H, dd, *J* = 8.7, 1.5 Hz), 7.48–7.55 (3H, m), 7.45 (1H, d, *J* = 7.6 Hz), 7.31 (1H, t, *J* = 7.6 Hz), 7.14 (5H, brs), 6.88–6.93 (2H, m), 4.56 (1H, d, *J* = 15.0 Hz), 4.26 (1H, d, *J* = 15.0 Hz), 3.84 (3H, s), 3.00 (1H, s); ^13^C-NMR (125 MHz, CDCl_3_) *δ*: 168.9 (C=O), 159.7 (C), 147.9 (C), 143.9 (C), 141.4 (CH), 137.4 (C), 135.3 (C), 131.1 (C), 129.7 (CH), 128.7 (2CH), 128.4 (2CH), 127.5 (2CH), 127.4 (CH), 123.7 (2CH), 119.9 (CH), 115.8 (CH), 112.7 (CH), 89.5 (C), 55.4 (CH_3_), 43.1 (CH_2_); EIMS *m*/*z*: 416 (M^+^, 98), 338 (17), 282 (17), 254 (15), 106 (59), 92 (15), 91 (100); HREIMS: 416.1361 (calcd for C_24_H_20_N_2_O_5_ 416.1372).

#### 2.2.44. 5-hydroxy-3-(3-methoxyphenyl)-1-(4-methoxyphenyl)-5-(4-nitrophenyl)-1*H*-pyrrol-2(5*H*)-one (**47**)

Following the general procedure, a mixture of (*E*)-4′-nitro-3-methoxychalcone (50 mg, 0.17 mmol) and 4-methoxyphenyl isocyanide (48.5 mg, 0.34 mmol) in H_2_O (1 mL) was irradiated at 150 °C for 1 h and after purification on silica gel preparative-TLC (Hex/AcOEt, 7:3), 21.3 mg (28%) of compound **47** were obtained as an orange oil; ^1^H-NMR (500 MHz, CDCl_3_) *δ*: 8.12 (2H, d, *J* = 9.0 Hz), 7.57 (2H, d, *J* = 9.0 Hz), 7.50–7.52 (1H, m), 7.41 (1H, dd, *J* = 7.4, 0.9 Hz), 7.29 (1H, td, *J* = 8.0 Hz), 7.21 (2H, d, *J* = 9.1 Hz), 6.99 (1H, s), 6.95 (1H, ddd, *J* = 8.3, 2.6, 0.9 Hz), 6.70 (2H, d, *J* = 9.1 Hz), 3.95 (1H, brs), 3.82 (3H, s), 3.71 (3H, s); ^13^C-NMR (125 MHz, CDCl_3_) *δ*: 168.4 (C=O), 159.6 (C), 157.9 (C), 147.9 (C), 144.3 (C), 141.6 (CH), 135.0 (C), 131.0 (C), 129.7 (CH), 127.8 (C), 127.4 (2CH), 126.7 (2CH), 123.8 (2CH), 119.9 (CH), 115.9 (CH), 114.1 (2CH), 112.7 (CH), 90.5 (C), 55.4 (CH_3_), 55.3 (CH_3_); EIMS *m*/*z*: 432 (M^+^, 65), 310 (29), 150 (43), 123 (14), 122 (100), 104 (14); HREIMS: 432.1339 (calcd for C_24_H_20_N_2_O_6_ 432.1321).

#### 2.2.45. 1-cyclohexyl-5-hydroxy-3-(2-methoxyphenyl)-5-(4-nitrophenyl)-1*H*-pyrrol-2(5*H*)-one (**48**)

Following the general procedure, a mixture of (*E*)-4′-nitro-2-methoxychalcone (50 mg, 0.18 mmol) and cyclohexyl isocyanide (44.8 µL, 0.35 mmol) in H_2_O (1 mL) was irradiated at 150 °C for 1 h and after purification on silica gel preparative-TLC (Hex/AcOEt, 4:1), 27.4 mg (38%) of compound **48** were obtained as an amorphous yellow solid; mp.: 158–160 °C; ^1^H-NMR (500 MHz, CDCl_3_) *δ*: 8.26 (1H, dd, *J* = 7.8, 1.7 Hz), 8.19 (2H, d, *J* = 9.0 Hz), 7.72 (2H, d, *J* = 9.0 Hz), 7.33 (1H, ddd, *J* = 9.2, 7.5, 1.7 Hz), 7.18 (1H, s), 7.01 (1H, td, *J* = 7.5, 1.0 Hz), 6.92 (1H, dd, *J* = 8.4, 0.7 Hz), 3.79 (3H, s), 3.18–3.27 (2H, m), 2.11–2.15 (1H, m), 2.00–2.05 (1H, m), 1.57–1.74 (3H, m), 1.53 (1H, d, *J* = 12.4 Hz), 1.36 (1H, d, *J* = 12.4 Hz), 1.05–1.24 (2H, m), 0.93–1.03 (1H, m); ^13^C-NMR (125 MHz, CDCl_3_) *δ*: 169.0 (C=O), 158.1 (C), 147.9 (C), 145.5 (C), 144.5 (CH), 131.4 (C), 130.5 (CH), 130.2 (CH), 127.5 (2CH), 123.6 (2CH), 120.5 (CH), 119.0 (C), 110.7 (CH), 90.1 (C), 55.4 (CH_3_), 53.2 (CH), 30.7 (CH_2_), 29.7 (CH_2_), 26.2 (CH_2_), 26.1 (CH_2_), 25.2 (CH_2_); EIMS *m*/*z*: 408 (M^+^, 100), 326 (42), 310 (65), 149 (99), 108 (27), 104 (29), 98 (34); HREIMS: 408.1703 (calcd for C_23_H_24_N_2_O_5_ 408.1685).

#### 2.2.46. 1-benzyl-5-hydroxy-3-(2-methoxyphenyl)-5-(4-nitrophenyl)-1*H*-pyrrol-2(5*H*)-one (**49**)

Following the general procedure, a mixture of (*E*)-4′-nitro-2-methoxychalcone (50 mg, 0.18 mmol) and benzyl isocyanide (43.9 µL, 0.35 mmol) in H_2_O (1 mL) was irradiated at 150 °C for 1 h and after purification on silica gel preparative-TLC (Hex/AcOEt, 4:1), 26.4 mg (36%) of compound **49** were obtained as an amorphous orange solid; mp.: 219–221 °C; ^1^H-NMR (500 MHz, CDCl_3_) *δ*: 8.38 (1H, dd, *J* = 7.8, 1.6 Hz), 8.07 (2H, d, *J* = 8.9 Hz), 7.53 (2H, d, *J* = 8.9 Hz), 7.34–7.38 (2H, m), 7.12–7.18 (5H, m), 7.07 (1H, t, *J* = 7.6 Hz), 6.94 (1H, d, *J* = 8.3 Hz), 4.55 (1H, d, *J* = 15.0 Hz), 4.24 (1H, d, *J* = 15.0 Hz), 3.81 (3H, s), 2.93 (1H, brs); ^13^C-NMR (125 MHz, CDCl_3_) *δ*: 169.7 (C=O), 158.2 (C), 147.9 (C), 145.1 (CH), 144.5 (C), 137.6 (C), 130.8 (C), 130.5 (CH), 130.4 (CH), 128.7 (2CH), 128.8 (2CH), 128.3 (2CH), 127.5 (2CH), 127.3 (CH), 123.6 (2CH), 120.6 (CH), 118.8 (C), 110.7 (CH), 89.7 (C), 55.4 (CH_3_), 43.0 (CH_2_); EIMS *m*/*z*: 416 (M^+^, 100), 400 (25), 145 (46), 252 (27), 149 (27), 108 (28), 106 (56); HREIMS: 416.1354 (calcd for C_24_H_20_N_2_O_5_ 416.1372).

#### 2.2.47. 5-hydroxy-3-(2-methoxyphenyl)-1-(4-methoxyphenyl)-5-(4-nitrophenyl)-1*H*-pyrrol-2(5*H*)-one (**50**)

Following the general procedure, a mixture of (E)-4´-nitro-2-methoxychalcone (50 mg, 0.17 mmol) and 4-methoxyphenyl isocyanide (48.5 mg, 0.35 mmol) in H_2_O (1 mL) was irradiated at 150 °C for 1 h and after purification on silica gel preparative-TLC (Hex/AcOEt, 4:1), 16.1 mg (21%) of compound **50** were obtained as an amorphous brown solid; mp.: 178–180 °C; ^1^H-NMR (500 MHz, CDCl_3_) *δ*: 8.30 (1H, dd, *J* = 7.8, 1.6 Hz), 8.12 (2H, d, *J* = 9.1 Hz), 7.61 (2H, d, *J* = 9.1 Hz), 7.40 (1H, s), 7.36 (1H, t, *J* = 7.6 Hz), 7.22 (2H, d, *J* = 9.1 Hz), 7.04 (1H, td, *J* = 7.6, 1.0 Hz), 6.94 (1H, d, *J* = 8.4 Hz), 6.72 (2H, d, *J* = 9.1 Hz), 3.82 (3H, s), 3.71 (4H, s); ^13^C-NMR (125 MHz, CDCl_3_) *δ*: 169.1 (C=O), 158.2 (C), 157.9 (C), 147.8 (C), 145.1 (CH), 145.0 (C), 131.0 (C), 130.5 (2CH), 128.1 (C), 127.4 (2CH), 126.9 (2CH), 123.7 (2CH), 120.7 (CH), 118.8 (C), 114.1 (2CH), 110.8 (CH), 90.6(C), 55.5 (CH_3_), 55.3 (CH_3_); EIMS *m*/*z*: 432 (M^+^, 99), 311 (21), 310 (97), 150 (95), 123 (16), 122 (100), 104 (23); HREIMS: 432.1351 (calcd for C_24_H_20_N_2_O_6_ 432.1321).

### 2.3. Computational Studies

#### 2.3.1. Protein Preparation and Docking Studies

Docking studies were carried out using Glide v8.6 (Schrödinger, LLC, New York, NY, USA, 2020). The X-ray coordinates of hERα ligand binding domains were extracted from the Protein Data Bank (PDB code 3ERT). The PDB structures were prepared for docking using the Protein Preparation Workflow accessible from the Maestro program (Maestro, version 12.3; Schrodinger, LLC, New York, NY, USA, 2020). The binding sites were enclosed in a grid box of 20 Å3. The three-dimensional structures of the ligands were generated and prepared using LigPrep implemented in Maestro 12.3. The geometries were optimized using the OPLS_2005 force field. Finally, the ligands were docked using the extra precision mode (XP). The selection of the best-docked pose for each ligand was performed using the XP Pose Rank [30,31].

#### 2.3.2. In Silico ADME and Drug-Likeness Analyses

The physicochemical parameters and ADME profile were acquired using the QikProp program version 6.3 (Schrödinger, LLC, New York, NY, USA, 2020 in Fast mode and based on the method of Jorgensen. Preparation of compounds and the 2D-to-3D conversion was performed using the LigPrep tool, a module of the Small-Molecule Drug Discovery Suite in the Schrödinger software package, followed by MacroModel v12.3 (Schrödinger, LLC, New York, NY, USA, 2020). A conformational search was executed using Molecular Mechanics, followed by a minimization of the energy of each conformer. For ADME studies, the global minimum energy conformer of each compound was employed. The drug-likeness analysis of 5-hydroxy-2*H*-pyrrol-2-one compounds was predicted using Lipinski’s rules [32].

### 2.4. Biological Evaluation

#### 2.4.1. Reagents

Estradiol (17β-E2, E2) was purchased from Sigma-Aldrich (St. Louis, MO, USA). The pure ERα and ERβ agonists, 1,3,5-tris(4-hydroxyphenyl)-4-propyl-1*H*-pyrazole (PPT) and 2,3-bis(4-hydroxyphenyl) propionitrile (DPN), and the antagonists 4-hydroxy-tamoxifen (4-OHTAM), and ICI-182,780 (ICI) were purchased from Tocris (Bristol, UK). Dimethyl sulfoxide (DMSO), used to prepare chemical stock solutions and vehicles (VEH), was purchased from Sigma-Aldrich. Stock solutions of E2, PPT, DPN, diethylstilbestrol (DES), testosterone (T) and dexamethasone (DEX) were prepared in pure ethanol and stored at −20 °C until used.

#### 2.4.2. Cells

Cell lines used in the in vitro experiments were purchased from the American Type Culture Collection (ATCC; Rockville, MD, USA). All cells were grown in a humidified incubator with 5% CO_2_ at 37 °C. Culture media were supplemented with 10% fetal bovine serum (FBS), L-glutamine (2 mM), and antibiotics (50 UI/mL penicillin and 50 μg/mL streptomycin (PEST)) which were purchased from Biowest (Nuaillé, France). Human ERα+ BC cell lines, including MCF-7, MCF-7/BUS, and T47D, were grown in RPMI-1640 (Biowest, Nuaillé, France) cell culture medium without phenol red, supplemented with 10 mM HEPES and 1 mM sodium pyruvate. The T47D-KBluc cell line, an ERα+ BC cell line that stably expresses a triplet ERE promoter-luciferase reporter plasmid (EREx3-Luc) [33], was maintained in the same medium as the previous ones but adding the selection antibiotic G418 (Thermo Fisher Scientific, Waltham, MA, USA). The ERα− human BC MDA-kb2 cell line that stably expresses an androgen receptor (AR)- and glucocorticoid receptor (GR)-responsive pMMTV.neo.luc reporter gene [34] was grown in the RPMI-1640 medium. The human SK-BR-3 cell line, a HER2+ BC cell, was grown in McCoy’s 5A medium supplemented with 10% fetal bovine serum (FBS), L-glutamine (2 mM), PEST, 1 mM sodium pyruvate and 10 mM HEPES. The TNBC cell lines, BT-549, MDA-MB-231 and Hs-578T were maintained in RPMI-1640 supplemented with 10% FBS, L-glutamine (2 mM), and PEST, 10 mM HEPES and 1 mM sodium pyruvate or DMEM with phenol red (Biowest, Nuaillé, France) culture media. The Ishikawa cell line, human endometrial ERα+ cancer cells, were grown in MEM without phenol red with Earle’s Salts culture media (Corning, New York, NY, USA). The non-tumorigenic human breast MCF-10A cell line was cultured in RPMI-1640 supplemented with 1 mM sodium pyruvate, 10 mM HEPES supplemented with MEGM Mammary Epithelial Cell Growth Medium Bulletkit (Lonza, Basel, Switzerland), and cholera toxin (Sigma-Aldrich). The non-malignant monkey kidney cells (VERO) were grown in DMEM. Human peripheral blood mononuclear cells (PBMCs) were isolated from healthy volunteers by density gradient centrifugation with Ficoll-Paque PLUS (GE Healthcare Bio-Sciences AB, Uppsala, Sweden) and cultured in RPMI-1640 medium. 

#### 2.4.3. Transcriptional Activity Assays

The ER+ BC cell line T47D-KBluc was used for the chemical screening; these cells are stably transfected with the pGL2.TATA.Inr.Luc.ne containing three ER-responsive elements (3xERE) [33]. When indicated, cells were deprived of E2 by using RPMI without phenol red, and 10% dextran-coated charcoal-stripped FBS (10DCC-FBS) (Biowest, Nuaillé, France). Dosing media was further modified by reduction to 5DCC-FBS. Then, T47D-KBluc cells were screened using E2 positive, E2 negative (VEH), antagonist (E2 plus ICI), and background (VEH plus ICI) controls on every plate. For agonist assessment, cells were treated with a test compound (3–6 h) with or without E2. For antagonist assessment, the incubation of T47D-KBluc cells with test compounds was performed in the presence of 0.1 nM E2. This concentration of E2 corresponded to the maximal luciferase activity (Emax). To further evaluate the estrogenic/antiestrogenic activities of test compounds, the dose-effect relationship of E2 (from 0.01 pM to 1 nM) was tested in the absence or in the presence of 5 μM of selected 5-hydroxy-2*H*-pyrrol-2-one compounds. Likewise, chemical screening was achieved by using the TNBC cell line MDA-kb2 that stably expresses pMMTV.neo.luc, an AR and GR-responsive reporter gene [34]. Cells were incubated with test compounds in the absence (VEH) or presence of 100 nM T or 100 nM DEX, a dose corresponding to the Emax of T- or DEX-dependent luciferase activity, respectively. Renilla-based reporter HEK-293 cells and Ba/F3 cells (Leeporter^TM^, Abeomics, San Diego, CA, USA), whose transcriptional activities are regulated by STAT3 and STAT5, respectively, were also used to explore ER-independent effects of 5-hydroxy-2*H*-pyrrol-2-one compounds; these cells were treated with the pure agonists hIL6 (10 ng/mL) (Biosupplies, Pepprotech, EE. UU.) or mIL3 (30 ng/mL) (Miltenyi Biotec, Bergisch Gladbach, Germany), respectively. T47D-KBluc cells or MDA-kb2 cells were seeded at 100,000 cells in 24 well plates (Thermo Fisher Scientific, Waltham, MA, USA ) and allowed to adhere overnight. Next, cells were washed with cold phosphate-buffered saline, harvested, and lysed in 100 µL Passive Lysis Buffer (Promega, Madison, WI, USA). Finally, Firefly-Luciferase or Renilla activities were measured with the kit Luciferase Assay Reagent (Promega, Madison, WI, USA) or Renilla Luciferase Assay kits (Thermo Scientific, Hanover Park, IL, USA), respectively, according to the light emission (RLU) provided by the microplate reader Clarity R2 (BioTek, Shoreline, WA, USA). RLU were normalized by protein concentration that was determined with the Bradford protein assay (Bio–Rad, Irvine, CA, USA) [35]. In parallel, MTT assays were carried out to detect cell viability. Maximal transcriptional activity from pure agonist E2 was expressed as Emax, which allowed the determination of EC_50_ and IC_50_ values.

#### 2.4.4. Cell Viability Assays

Cells were seeded at exponential growth densities (2500–20,000 cells per well) in 96-well plates (BD Falcon, Paris, France), and treated with VEH (0.05% DMSO), test compounds (0.01 to 25 μM) or ER antagonist ICI (0.01 to 25 μM) and 4-OHTAM (0.01 to 25 μM) for 48–72 h. Straightforward, the tetrazolium salt 3-(4,5-methyltiazol-2yl-)-2,5diphenyl-tetrazolium bromide (MTT) (Applichen, Darmstadt, Germany) was added to cells, incubated for 2–4 h at 37 °C, and lysed in 10% SDS. The mitochondrial metabolization of the tetrazolium salt was determined by the analysis of the optical density at 595 nm [36], measured with the iMark microplate Reader (Bio–Rad, Irvine, CA, USA).

#### 2.4.5. Human ERα Competitor Binding Assay

The LanthaScreenTM TR-FRET Nuclear Receptor (NR) Fluorescence Polarization (FP) binding assay (LanthaScreenTM TR-FRET Competitive Binding Assay Screening Protocol and Assay Conditions, 2016, SelectScreenTM Profiling Service, Life Technologies, Carlsbad, CA, USA) was used for evaluating the potential binding of 5-hydroxy-2*H*-pyrrol-2-one compounds to LBD of hERα. This kit uses the rhERα protein and a tight-binding selective fluorescent ligand, the FluormoneTM tracer. The assay is optimized to bind 80% of the tracer without right-shifting IC_50_ values. Compounds that displace the tracer tumbles rapidly, resulting in a low FP value, but the FP value remains high in the presence of compounds that do not displace the tracer from the complex. The shift in FP values in the presence of test compounds (from 0.10 pM to 20 μM) was used to detect the relative affinity of compounds for the rhERα protein. GraphPad software 8 (GraphPad Software, San Diego, CA, USA) was used to acquire dose-response competition curves that were fitted by nonlinear regression analyses to obtain IC_50_ values.

#### 2.4.6. Rat ER Competitor Binding Assay

Animal studies were approved by the Bioethics Committee of the University of Las Palmas de Gran Canaria (OEBA-ULPGC 40/2020), and the experiments were carried out according to OECD guidelines (ER-RVC OPPTS 890.1250). ER was obtained from rat uterine cytosol (RUC). Briefly, RUC was extracted from 8–12 weeks old Sprague-Dawley rats 13–16 days after they were ovariectomized under ketamine (75 mg/kg)/medetomidine (1 mg/kg) anesthesia [37]. Uteri were removed, trimmed free of adipose tissue, blotted, weighed and frozen on liquid nitrogen until RUC isolation. Then, 100 mg of uteri per ml of ice-cold TEGM buffer (10 mM Tris, 1.5 mM EDTA, 10% glycerol, 3 mM MgCl_2_, 1 mM PMSF, 1 mM DTT, pH 7.4) were homogenized by using a Polytron PT3000 homogenizer (Kinematica, Malters, Switzerland) at 15,000 rpm for 3 bursts of 30″ each. The homogenate was sedimented, and the supernatant was centrifuged at 105,000 g for 60 min at 4 °C to obtain RUC. Protein concentration was determined by Bradford´s assay (Bio-Rad, Irvine, CA, USA), and 2 mg/mL of RUC was taken from all samples [35]. Aliquots of 100 μL RUC were incubated with 3 nM [^3^H]E2 (Estradiol [2,4,6,7-^3^H(N)]; SA: 70–115 Ci/mmol; >97% purity) (PerkinElmer) in the absence or presence of increasing concentrations of unlabeled competitors (from 0.1E-9 M to 50E-6 M) for 18 h at 4 °C [37]. Then, 200 μL of DCC suspension (0.8% charcoal: 0.08% dextran; *w:w*) in cold TE buffer was added to each tube and incubated for 10 min before DCC was centrifuged at 3000 g for 10 min at 4 °C. Supernatant (200 μL) was obtained to measure total and non-specific bound radioactivity in TRICARB 4810 LSC counter (PerkinElmer). Corrections were made for non-specific binding by using 500-fold excess of unlabeled DES (BioSigma, Cantarana, Italy). For ER competition assays, intra-assay data were normalized by reference to specific binding determined with 5 nM [^3^H]E2. Then, data were expressed as specific [^3^H]E2 binding, which was displaced by rising concentrations of unlabeled test compounds. Dose-response competition curves were fitted to four-parameter logistic equations by nonlinear regression analyses in GraphPad software 8 (GraphPad Software, San Diego, CA, USA) to obtain IC_50_.

#### 2.4.7. Real-Time Monitoring of 2D-3D Tumor Growth

To monitor long-term growth, human ER+ BC (T47-D, MCF-7, MCF-7/BUS), ER+ endometrial (Ishiwaka, High Point, NC, USA), or non-malignant breast (MCF-10A) cells were seeded at 3000–6000 cells per well. Cells were grown in a complete medium and treated with VEH (0.05% DMSO), test compounds (0.03–25 μM), ICI (0.03–25 μM) or 4-OHTAM (0.03–25 μM). When indicated, cells were estrogen-deprived in a 10DCC-FBS supplemented medium for one week. Then, cells were grown in 5DCC-FBS supplemented medium and treated with VEH (0.05% DMSO), test compounds (0.03–10E-6 M), ICI (0.03–10 nM) or 4-OHTAM (0.01–10 nM), in the absence or presence of E2 (0.1 nM). Cytotoxicity was monitored by using YOYO-1 (Invitrogen, Waltham, MA, USA). Cell proliferation and cytotoxicity were monitored by sequential real-time microphotographs in an IncuCyte HD real-time imaging system (Essen BioScience, Hertfordshire, UK) for 5–10 days. The area under the curve (AUC) was then analyzed to know both the kinetics and dose-effect relationships. To monitor the effects of 5-hydroxy-2*H*-pyrrol-2-one compounds on cell spheroids, MCF-7 or T47-D cells were seeded at 1000 cells per well in round-bottom, ultralow attachment, 96-well plates (Corning, New York, NY, USA) and growth in RPMI supplemented with 10%FBS, glutamine (2 mM) and PEST for 48–72 h to form spheroids. Next, cells were treated with VEH (0.05% DMSO) or test compounds for 5–10 days. Spheroid size and cell cytotoxicity were recorded with the Incucyte SX5 Live-Cell Analysis Instrument (Sartorius AG, Göttingen, Germany). To calculate the spheroid growth area, confluence measurements were taken considering the largest brightfield object area (µm^2^). Cytotoxicity was monitored by using YOYO-1 (Essen Bioscience, Hertfordshire, UK).

#### 2.4.8. Alkaline Phosphatase Assay

Human endometrium cells (Ishiwaka) were cultured in MEM without phenol red with Earle’s Salts culture, which was supplemented with estrogen-deprived 10 DCC-FBS du-ring 5 days [38]. Then, 100000 cells were plated per well in 12-well plates to be treated with VEH (0.05% DMSO), test compounds (0.3–10 μM), ICI (0.1 μM) and 4-OHTAM (0.3–3 μM), for 24, 48 and 72 h, in the absence or in the presence of E2 (10 nM). Cells were washed with ice-cold PBS, and Alkaline Phosphatase (ALP) activity was measured by using ALP Assay Kit (Abcam, Cambridge, MA, USA). The substrate p-nitrophenyl phosphate (pNPP) and the ALP enzyme were used to detect ALP activity by the analysis of the optical density at 405 nm in the iMark microplate Reader (Bio-Rad, Irvine, CA, USA). Maximal ALP activity from pure agonist E2 was expressed as Emax (100%), which allowed the determination of EC_50_ and IC_50_ values. Protein concentration was quantified with the bicinchoninic acid assay (BCA) kit (Bio-Rad).

#### 2.4.9. Cell Cycle and Apoptosis Analysis

For cell cycle studies, unsynchronized T47D cells were seeded at 250,000 cells/well in RPMI (Biowest, Nuaillé, France) 10% FBS medium supplemented with L-glutamine (2 mM), HEPES (10 mM), sodium pyruvate (1 mM) and PEST. Cells were treated with VEH (0.05% DMSO), 4-OHTAM (5 µM) or test compounds (5 µM) for 24 to 72 h. Cells were then washed in ice-cold PBS and fixed in cold 70% ethanol at −20 °C overnight. Then, the cell pellet was resuspended in FACs buffer (2 mM EDTA, 2% FBS in PBS) and incubated with Ki67-APC antibody (Miltenyi Biotec, Auburn, CA, USA) and DAPI (4′,6-diamidino-2-phenylindole; Sigma-Aldrich) for 20 min at room temperature. Finally, cells were washed and resuspended with FACs Buffer for cell cycle analyses. For apoptosis studies, an Annexin V-FITC Apoptosis Kit (Miltenyi Biotec) was used. Briefly, treated cells were collected in 100 μL of ice-cold AnnexinV binding buffer (10 mM HEPES pH 7.4, 140 mM NaCl, 2.5 mM CaCl_2_) containing 10 μL of Annexin-V FITC and incubated for 15 min at room temperature. After that, cells were washed with 1 mL binding buffer, sedimented at 300× *g* 10 min, and resuspended with 500 μL binding buffer containing 5 μL of propidium iodide (PI; 100 μg/mL) prior to acquisition in MACSQuant10 cytometer (Miltenyi Biotec) where 10,000 or 20,000 events were used for cell cycle and apoptosis analysis, respectively.

#### 2.4.10. Immunoblotting

T47D cells (1,000,000 cells) were seeded in non-estrogen depleted RPMI medium supplemented with 10% FBS, L-Glutamine/HEPES/sodium pyruvate/PEST and treated with VEH (0.05% DMSO), test compounds (5–10 µM), ICI (5 µM) or 4-OHTAM (1 µM) for 24 to 72 h. Then, they were washed with ice-cold PBS supplemented with 1 mM ortovanadate and lysed with RIPA buffer containing protease and phosphatase inhibitors (Thermo Fisher Scientific, Waltham, MA, USA). Equal protein amounts were analyzed by SDS–PAGE, and transferred to nitrocellulose membranes (Thermo Fisher Scientific, Waltham, MA, USA), blocked with Tris-buffered saline/0.05% Tween 20 (TBS-T) containing 5% blotto (Merck, Darmstadt, Germany) or 5% BSA for total and phosphorylated proteins, respectively. Immunoblotting was carried out with mouse anti-ERα F-10 (Santa Cruz Biotech, Santa Cruz, CA, USA), rabbit total anti-STAT5 (Cell Signaling Technology, Leiden, The Netherlands) or mouse anti-polyubiquitin (EMD Millipore, Burlington, MA, USA). After washing, membranes were incubated with horseradish peroxidase-conjugated secondary antibodies (Bio-Rad) for 1 h at room temperature. The β-actin antibody (Santa Cruz Biotech, CA, USA) was used as the loading control. Finally, protein bands were visualized by using Clarity™ Western ECL Substrate (Bio-Rad) in the ChemiDoc XRS system (Bio-Rad) and were analyzed with Quantity One software (Bio-Rad).

#### 2.4.11. Immunofluorescence

T47D cells were seeded with steroid-deprived FBS (10% DCC-FBS) RPMI medium supplemented with L-Glutamine/HEPES/sodium pyruvate/PEST. Then, cells were seeded at 175,000 cells/well in 5% DCC-FBS in 6-well plates containing 22 × 22 mm coverslips (Menzelgläser, vWR, Radnor, PA, USA). The T47-D cells were then pretreated with VEH (0.05% DMSO), a test compound (5 µM), or 4-OHTAM (5 µM) for 24 h. Next, E2 (1 nM) was added to the cultures for 30 min. After this, cells were washed with PBS, fixed in 4% paraformaldehide (PFA; Sigma-Aldrich, St. Louis, MO, USA) for 15 min, blocked-permeabilized in PBS-0.5% BSA-0.1% Triton X-100 (Thermo Scientific, Waltham, MA, USA) for 1 h, and, finally, incubated with mouse ERα (Santa Cruz Biotech, CA, USA) and rabbit β-catenin (C2206 Sigma-Aldrich, St. Louis, MO, USA) primary antibodies in the same blocking solution overnight at 4 °C within a hand-made humid chamber. After three washing with PBS-0.5% BSA-0.1% Triton X-100 (Thermo Scientific, Waltham, MA, USA), cells were incubated with anti-mouse Alexa fluor 488 (Life Technologies, CA, USA) or anti-rabbit Alexa 647 (Life Technologies, Carlsbad, CA, USA) secondary antibodies, containing DAPI (4′,6-diamidino-2-phenylindole; Sigma-Aldrich, St. Louis, MO, USA), for 1 h at room temperature [39]. Finally, slides were mounted with Fluormount-G^®^ (Thermo Fisher Scientific, Waltham, MA, USA), and cells were visualized using a confocal microscope Zeiss LSM 800 (Carl Zeiss AG, Jena, Germany). The fluorescent signal was quantified by using a segmentation method for image analysis [40].

#### 2.4.12. Quantitative Real-Time PCR

For gene expression analysis, MCF-7 and Ishikawa cells were deprived of steroid hormones by using a 10DCC-FBS medium for five days. Then, 1,500,000 cells/dish were seeded in 5DCC-FBS, followed by treatment with test compounds. Total RNA was isolated by using TRItidy G™ (Panreac Applichem ITW Reagents, Darmstadt, Germany). The concentration and purity of RNA were determined with a NanoDrop 1000 spectrophotometer (Wilmington, NC, USA). Then, 1 µg of total RNA was reversed and amplified with the iScriptTM kit (Bio-Rad) following the manufacturer’s protocol. SYBR Green PCR Master mix (Applied Biosystems, Bedford, MA, USA) reagent and exon-specific primers for ERα were used for Real-time quantitative PCR (qPCR) [41]. Gene expression was analyzed with an Mx3005P qPCR System (Agilent, Santa Clara, CA, USA), and cycle threshold values were calculated using MxPro qPCR Software (Agilent, CA, USA). Target gene expression was normalized to the expression of the 18S housekeeping gene. Data were expressed as relative expression values according to the comparative CT method for qPCR [42].

#### 2.4.13. Drug Combination Assays

To investigate the potential synergism between test compounds and 4-OHTAM they were tested alone or in combination on MCF-7 and MCF-10A cells. Cells were seeded at 5000 and 7500 cells, per well, respectively, and cell viability was assessed by MTT assays 72 h after treatment. Briefly, cells were incubated with constant ratio combinations of indicated test compounds by doubling dilutions of the individual drugs over a wide range of concentrations [43]. Inhibition of cell viability, relative to untreated controls, was assigned as the effect and ranged from 0 (no cell viability inhibition) to 1 (100% cell viability inhibition). Dose-effect curves of the individual or combined compounds were plotted and assessed by the median effect method of Chou and Talalay [43] using Calcusyn software 2.0 (Biosoft, Cambridge, UK), thus obtaining the combination index (CI) values. CI values less than 1, equal to 1, and greater than 1 indicated synergism, addition, and antagonism, respectively.

#### 2.4.14. Statistical Analysis

Dose-response curves were fitted by nonlinear regression analyses in GraphPad Prism 8.4.3 software (GraphPad Software, La Jolla, CA, USA). Then, the concentrations required to reduce the agonistic effect of E2 by 50% (IC_50_) or to increase basal level by 50% (EC_50_) were determined. Differences between the means of the two groups were analyzed with a two-tailed Student’s *t*-test, whereas one-way ANOVA followed by a post hoc test was performed to compare means from more than two groups. The data shown are the result of two to four independent experiments with at least three replicates per experimental condition. Results are expressed as the mean ± SEM. Statistical significance was considered when *p* < 0.05.

## 3. Results and Discussion

### 3.1. Virtual Screening and Synthesis of Highly Functionalized 5-hydroxy-2H-pyrrol-2-ones as ER Modulators

**Figure 1:**
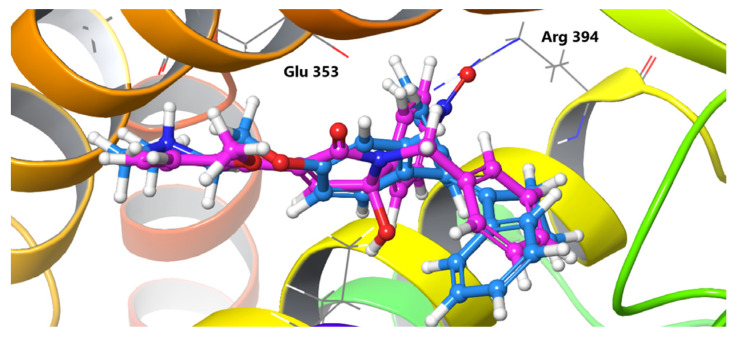
Predicted binding modes of hydroxy-diaryl-1,5-dihydro-2*H*-pyrrol-2-one scaffold (Magenta, docking score value: −8.75 kcal/mol) into the binding site of ERα and superimposed on the crystal structure of 4-hydroxy-tamoxifen (blue, PDB 3ERT).

First, virtual screening of the crystal structures of ERα using an in-house chemical library with structural features consistent with the pharmacophoric model defined for the non-steroidal SERMs was carried out [44,45]. The screened library included different scaffolds such as lignans, aurones, chalcones, triazoles, pyrazoles and 2*H*-pyrrol-2-ones. Therefore, those compounds presenting at least one of the key interactions exhibited by 4-OHTAM, such as hydrogen bond interactions with residues Glu 353 and Arg 394, as well as the hydrophobic interaction with the residue Phe 404, were selected. From this screening, the hydroxy-diaryl-1,5-dihydro-2*H*-pyrrol-2-one scaffold was selected since it showed good docking score values and similar poses to those of reported SERMs such as 4-OHTAM or raloxifene (Figure 1).

**Scheme 1:**
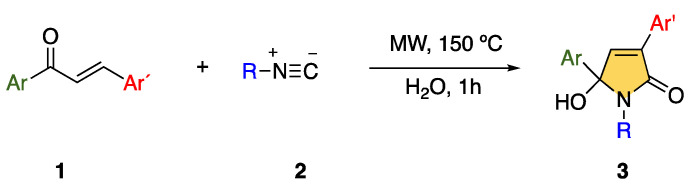
Preparation of 5-hydroxy-3,5-diaryl-1,5-dihydro-2*H*-pyrrol-2-ones.

Thus, based on the previous results, the synthesis of a set of 5-hydroxy-2*H*-pyrrol-2-one derivatives was addressed to evaluate their biological activity as ER modulators. In this sense, 5-hydroxy-3,5-diaryl-1,5-dihydro-2*H*-pyrrol-2-ones (**3**) were synthesized using a one-pot two-component reaction following a modified methodology described by Adib et al. [46] from 1,3-diaryl-2-propen-1-ones (chalcones) (**1**) and commercial isocyanides (**2**) under microwave irradiation at 150 °C in water (Scheme 1).

The chalcones were obtained from commercial sources or prepared via the Claisen-Schmidt condensation reaction between substituted acetophenone and the appropriately substituted aromatic aldehyde in basic conditions (20% aqueous KOH in ethanol).

**Figure 2:**
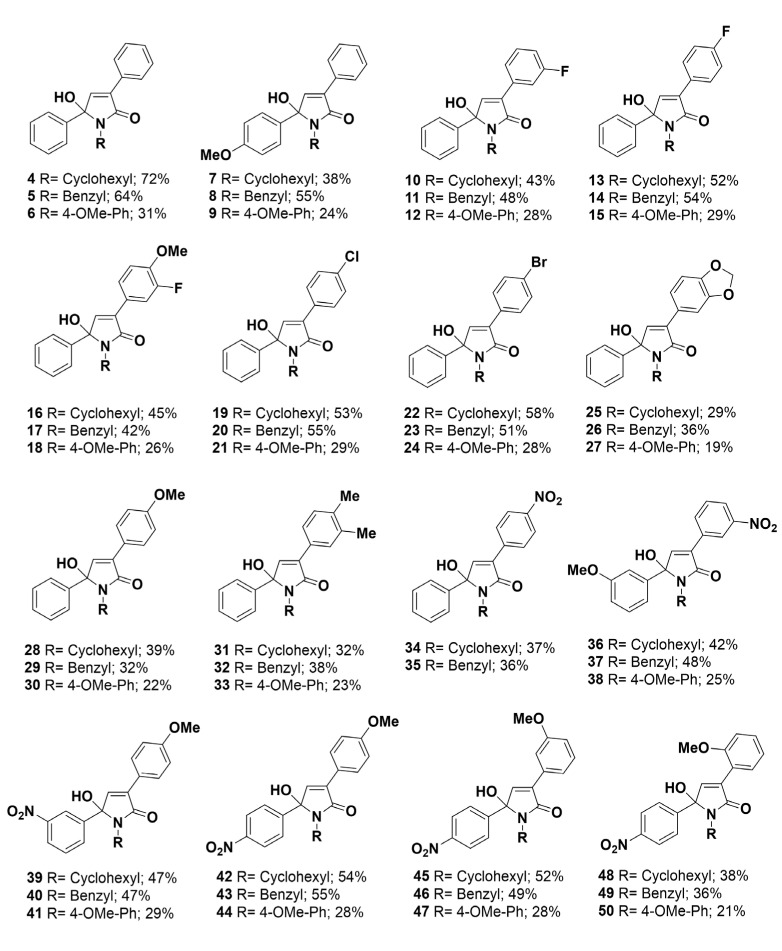
Structures and yields of 5-hydroxy-3,5-diaryl-1,5-dihydro-2*H*-pyrrol-2-ones (**4**–**50**).

The structures and the isolated yields of the synthesized pyrrolidones are shown in Figure 2. Diversely substituted pyrrolidones could be prepared in moderated yields from cyclohexyl, benzyl and 4-methoxyphenyl isocyanides, demonstrating the versatility of this process. All obtained compounds were docked on the crystal structures of ERα, and the corresponding docking score values are included in Supplementary Figure S1 and Table S1.

### 3.2. 5-hydroxy-2H-pyrrol-2-ones Inhibit Cell Viability of ER+ Breast Cancer Cells

The effects of 5-hydroxy-2*H*-pyrrol-2-ones on cell viability were explored in ER+ and ER-cancer cells as well as in non-malignant cells. Table 1 shows the best results obtained for the viability of ER+ cancer and non-malignant cells. Notably, treatment of E2-non-depleted cells with compounds **32**, **35**, **43** and **49** decreased the viability of ER+ cancer cells (i.e., MCF-7, MCF-7/BUS, T47D and Ishikawa cells) with IC_50_ values lower than 10 μM. The antitumoral potency for compound **32** (IC_50_ from 0.27 to 10 μM) or compound **35** (IC_50_ from 1 to 5 μM) in ER+ cancer cells was relatively higher compared to several 5-hydroxy-2*H*-pyrrol-2-one analogs with IC_50_ values over 10 μM (Table 1) and other reported pyrrolidone-type compounds [24,25]. Interestingly, compounds **32** and **35** also decreased the viability of ER− BC cells (i.e., SK-BR-3, BT-549, Hs-578T, MDA-MB-231), but with relatively lower potency than ER+ BC cells (Table 1). Clinically relevant, both compounds were less potent to reduce the viability of non-malignant kidney cells (i.e., VERO) and PBMC cells, but exerted potent inhibition of viability in MCF-10A cells, a model of non-malignant human breast cells. This finding opens the possibility that molecular targets of **32** and **35** compounds are expressed in both ER+ BC as well as in non-malignant breast cells, thus suggesting there might be different molecules apart from ER acting as targets of these compounds.

### 3.3. 5-hydroxy-2H-pyrrol-2-ones Modulate ER-Dependent Transcription in Breast Cancer Cells

Next, the analysis of the 5-hydroxy-2*H*-pyrrol-2-ones (**4**–**50**) was performed in a target-based screening by using stably transfected T47D-KBluc cells, a human ER+ BC cell line which contains 3xER responsive element (ERE) coupled to the luciferase reporter gene [33]. As expected, maximal ER-dependent transcriptional activity (i.e., Emax) was induced with the pure agonist E2 (EC_50_ = 4.48 ± 0.42 pM) in a dose-effect dependent manner, an effect that was abolished by the co-incubation with the antiestrogens ICI-182.780 (ICI) (IC_50_ = 0.2 ± 0.08 nM) and 4-OHTAM (IC_50_ = 0.38 ± 0.009 μM), agreeing with previous studies [47]. The estrogenic and antiestrogenic activities of the 5-hydroxy-2*H*-pyrrol-2-ones (from 1 μM to 10 μM) were analyzed in the absence or presence of E2, respectively, and data from the more representative compounds are summarized in Table 2. The results showed that some compounds (10 μM) (e.g., compound **16**) exerted a partial induction of ER-dependent transcriptional activity. In addition, other compounds (**4**, **8**, **26**, **35**, **38**, **40**, **46**, **50**) reduced E2-induced luciferase activity until dropping to 20% Emax. Compounds that displayed an Emax inhibition of E2-stimulated cells over 50%, admitted IC_50_ calculations with values ranging from 3 μM to >10 μM (Table 2).

**Figure 3:**
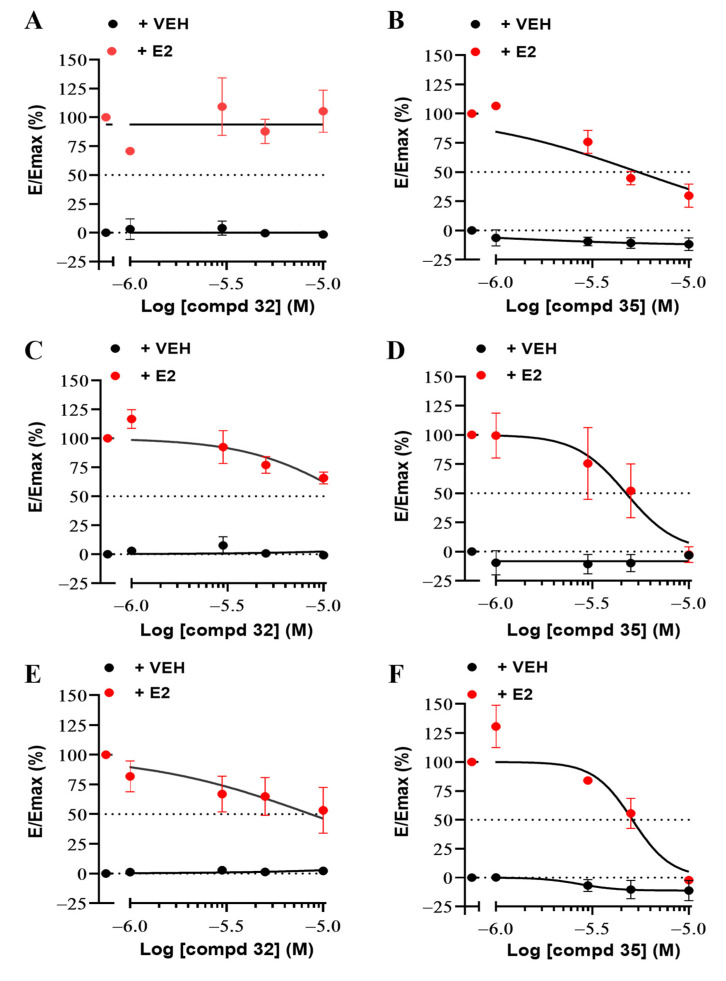
5-hydroxy-2*H*-pyrrol-2-ones **32** and **35** show a time-dependent antagonism on ER-dependent transcriptional activity. E2-depleted T47D-KBluc cells were pretreated with compounds **32** (**A**,**C**,**E**) or **35** (**B**,**D**,**F**) in a dose-response (1–10 µM) for (**A**,**B**) 3 h, (**C**,**D**) 12 h or (**E**,**F**) 24 h, before addition of vehicle (VEH; 0.05% DMSO, ●) or E2 (0.1 nM, ●) for 16–24 h. Relative Luciferase activity (RLU) was measured as described in the Material and Methods. The maximal luciferase activity or Emax (15.19 ± 1.71-fold induction) was induced by E2, and the efficacy (E) of each treatment, as compared with Emax, was calculated (E/Emax%). Dotted lines are shown at 0% and 50% of E/Emax. Non-linear regression analysis was applied with GraphPad Prism software 8.4.3 to calculate IC_50_ values. Data are expressed as mean ± SEM for at least three independent experiments, where each treatment was tested in triplicate.

Surprisingly, it was found that the inhibitory potency of some compounds, such as **32** on E2-induced luciferase activity (IC_50_ >10 μM) (Table 2) was not correlated with its potency to inhibit the viability of ER+ cancer cells (Table 1). In order to elucidate this apparent paradox, the time-dependent effect of compounds **32** and **35** on E2-induced luciferase activity was assessed in T47D-KBluc cells [33]. Interestingly, whereas the maximal antagonism of compound **32** (IC_50_ = 14.76 ± 7.61 μM) required at least 12 h (Figure 3A,C,E), the antagonism of compound **35** (IC_50_ = 8.20 ± 1.19 μM) on E2-induced luciferase activity was rapidly observed after 3 h exposure (Figure 3B,D,F).

**Figure 4:**
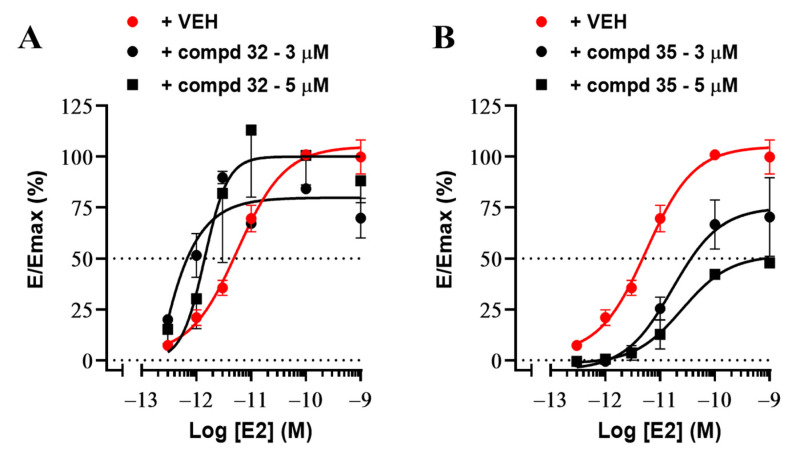
Differential inhibitory effects of 5-hydroxy-2*H*-pyrrol-2-ones **32** and **35** on ER-dependent transcriptional activity. E2-depleted T47D-KBluc cells were pretreated with vehicle (VEH, 0.05% DMSO, ●), compound **32** (**A**) or **35** (**B**) (both at 3 µM, ● or 5 µM, ■) for 3 h, followed by increasing E2 concentrations (0.3 pM–1 nM) for 16–24 h. Relative Luciferase activity (RLU) was measured as described in the Material and Methods. The maximal luciferase activity or Emax (15.19 ± 1.71-fold induction) was induced by E2, and the efficacy (E) of each treatment, as compared with Emax, was calculated (E/Emax%). Dotted lines are shown at 0% and 50% of E/Emax. Non-linear regression analysis was applied with GraphPad Prism software 8.4.3 to calculate the EC_50_ values. Data are expressed as mean ± SEM for at least three independent experiments, where each treatment was tested in triplicate.

To further assess the type of antagonism of compounds **32** and **35** on E2-induced luciferase activity, a dose-effect relationship of E2 (from 0.3 pM to 1 nM) was analyzed in the absence (E2+VEH) or in the presence of a constant concentration of compound **32** or **35** (Figure 4). Interestingly, the combination of compound **32** with E2 increased its potency to induce ER-dependent transcriptional activity (from EC_50_ = 4.57 ± 0.42 pM (E2+VEH) to 1.41 ± 0.39 pM at 5 μM and 0.90 ± 0.1 pM at 3 μΜ (E2+compound **32**), mean ± SEM). However, compound **32** reduced the capacity of E2 to reach its maximal activity (i.e., Emax) (from 99.76 ± 8.44% (E2+VEH) to 69.85 ± 9.84% at 3 μM (E2+compound **32**), mean ± SEM), thereby suggesting the antiestrogenic with partial agonism condition of the compound on ER-dependent transcriptional activity (Figure 4A). In contrast, Figure 4B shows that compound **35** significantly reduced the potency of E2 to induce ER-dependent transcriptional activity (from EC_50_ = 4.48 ± 0.42 pM to 16.80 ± 1.72 pM at 3 μM, and to 25.20 ± 2.8 pM at 5 μM, mean ± SEM) and to reach Emax (from IC_50_ =104.90 ± 2.01% to 75.11 ± 12.37% at 3 μM, and to 51.57 ± 13.08% at 5 μM, mean ± SEM), thus indicating a pure antagonism involved in the antiestrogenic action of compound **35**. Furthermore, these data indicate that compounds **32** and **35** differ in their respective molecular mechanism of antiestrogen action. 

Since there is high homology among ligand binding domains of ER, androgen (AR) and glucocorticoid (GR) receptors [16], we further assessed the effects of compounds **32** and **35** on AR- and GR-dependent transcriptional activity. Thus, the triple negative BC (TNBC) cell line MDA-kb2 [34] was screened with compounds **32** and **35** in the absence or presence of 100 nM Testosterone (T) or 100 nM Dexamethasone (DEX), the lowest concentrations that produced maximal androgenic- or glucocorticoid-induced luciferase activity in this cell line, respectively. Neither 4-OHTAM nor compounds **32** or **35** displayed cross-activation on T- or DEX-induced luciferase activity (Supplementary Figure S2). Likewise, since the Signal Transducer and Activator of Transcription (STAT) factors 3 and 5 are linked to ERα signaling in BC [48] and antitumoral effects of some SERMs target the expression of these two transcription factors [49], the effect of compounds **32** and **35** on cytokine activated STAT signaling was explored (Supplementary Figure S3). Interestingly, compound **32** inhibited IL6-induced STAT3 transcriptional activity in HEK293 cells (IC_50_ = 2.36 ± 0.75 μM), whereas compound **35** was inactive in these cells (Supplementary Figure S3A). Conversely, compound **35** inhibited IL3-stimulated STAT5 transcriptional activity in Ba/F3 cells (IC_50_ = 11.93 ± 1.73 μM), with no activity observed for compound **32** (Supplementary Figure S3B). This inhibition of STAT3 or STAT5 by compounds **32** and **35**, respectively, may influence the STAT-mediated activation of PI3K, AKT and mTOR signaling and, thereby, the biological responses regulated by these proteins [50]. There is compiling evidence regarding the functional connection between ERα and IL-6/STAT3 signaling in a tumoral context, although recent studies identified an IL-6/STAT3-activated transcriptional program in BC that is independent of ERα and promotes a more aggressive and metastatic phenotype [51]. Nonetheless, whether the inhibition of STAT3- or STAT5-regulated transcription contributes to the antiestrogenic effects of compounds **32** and **35** in ER+ BC cells deserves further research in order to elucidate if these transcription factors are direct targets of both compounds and, in contrast, their modulation is due to non-direct effects mediated by ER signaling.

### 3.4. 5-hydroxy-2H-pyrrol-2-ones 32 and 35 Bind to Human and Rat ERα

**Figure 5:**
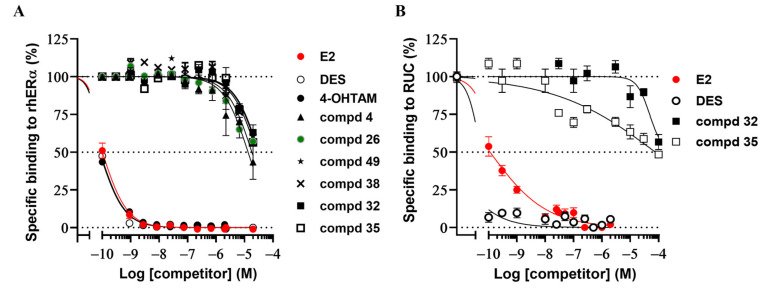
5-Hydroxy-2*H*-pyrrol-2-ones bind to rhERα and rat ERα with low affinity. (**A**) 5-Hydroxy-2*H*-pyrrol-2-ones **4**, **26**, **32**, **35**, **38**, **49** or control compounds (E2, DES or 4-OHTAM) competitors binding to rhERα was evaluated in 10-point titration competition curves (0.1 nM–20 µM) by using the LanthaScreen TR-FRET binding assay as described in Material and Methods. (**B**) The binding of competitors (compounds **32**, **35**, DES and E2) to native ERα was evaluated in 10-point titration competition curves (0.01 pM–100 µM) by using the radiolabeled E2 binding assay in rat uterine cytosol (RUC) as described in Material and Methods. Dotted lines are shown at 0%, 50% and 100% of Specific binding to rhERα. Non-lineal regression analysis was performed by using data from two independent experiments in GraphPad Prism 8.4.3. Data are expressed as mean ± SEM for two independent assays where each concentration was tested in duplicate.

Inhibition of ER activity by antiestrogens is mediated by competitive displacement of E2 from ER. Accordingly, LanthaScreenTM TR-FRET Competitive Binding Assay, which uses the competition of the selective fluorescent ligand FluormoneTM for the ligand binding domain of the recombinant human ERα (rhERα) (Kd = 0.23 nM), revealed that the ligand was displaced with high affinity by E2 (IC_50_ = 0.10 ± 0.00 nM), the synthetic estrogen diethylstilbestrol (DES) (IC_50_ = 0.08 ± 0.00 nM), and 4-OHTAM (IC_50_ = 0.08 ± 0.00 nM) (Table 3; Figure 5A). 

In contrast, 5-hydroxy-2*H*-pyrrol-2-one compounds **32** and **35** caused the half-maximal displacement of FluormoneTM from rhERα with IC_50_ that exceeded 10 μM (Figure 5A). Similarly, when the binding of competitors to native ERα was also evaluated by using the radiolabeled E2 binding assay in rat uterine cytosol (RUC) extracts (Figure 5B), non-linear regression analysis estimated that compounds **32** (IC_50_ = 128.4 ± 1.25 μM) and **35** (IC_50_ = 76.59 ± 1.30 μM) displaced radiolabeled E2 with relatively low affinity; these data together with luciferase assay results suggest that antiestrogenic effects of both compounds are in part mediated by their binding and blockade of ERα but also by alternative mechanisms that are independent of the receptor. Therefore, further studies will be neccesary to identify what other molecules are targets of compounds **32** and **35**.

### 3.5. 5-hydroxy-2H-pyrrol-2-ones 32 and 35 Inhibit Cell Growth of ER+ Breast Cancer Cells

**Figure 6:**
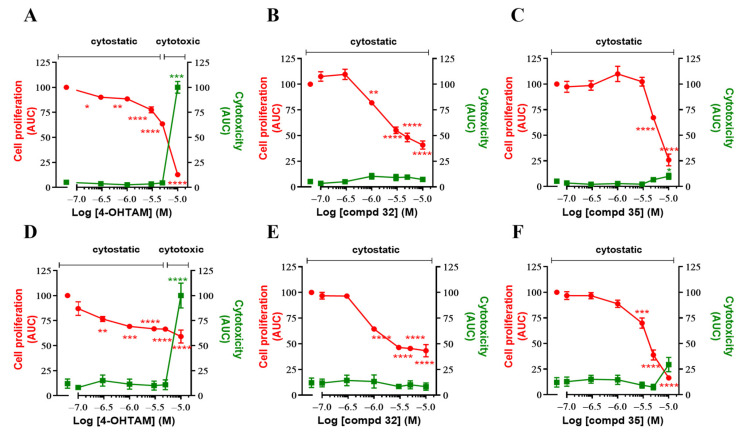
5-Hydroxy-2*H*-pyrrol-2-ones **32** and **35** reduce cell growth of human ER+ breast cancer cells with low cytotoxicity. (**A**–**C**) T47D and (**D**–**F**) MCF-7 cells were seeded in complete estrogenic media (10%FBS) and treated with vehicle (VEH, 0.05% DMSO) or different doses of compounds (0.01–10 µM) for 5 days. Data represent the area under the curve (AUC) by compounds dose in terms of proliferation in phase contrast (left *Y*-axis) and green-fluorescent cytotoxicity (right *Y*-axis). The mean ± SEM values are represented for each dose, comparing proliferation with respect to the maximum cell growth of VEH cells, and the maximum cytotoxicity with respect to the effect caused by the highest dose of 4-OHTAM. Statistical significance was assessed using one-way ANOVA with the Bonferroni post hoc test. * *p* < 0.05, ** *p* < 0.01, *** *p* < 0.001, **** *p* < 0.0001 vs. VEH-treated cells.

The effects of compounds **32** and **35** on the viability and growth of MCF-7 and T47D cells were next monitored by sequential 2D-3D real-time microphotographs for 5–8 days. First, long-term analysis on 2D cell growth showed that compound **32** (IC_50_ = 5.34 ± 2.47 μM for T47D cells (Figure 6B); IC_50_ = 1.52 ± 1.25 μM for MCF-7 cells (Figure 6E)) and compound **35** (IC_50_ = 6.98 ± 1.15 μM for T47D cells (Figure 6C); IC_50_ = 4.30 ± 0.77 μM for MCF-7 cells (Figure 6F)) exerted potent inhibition on T47D and MCF-7 cell growth (Figure 6). Interestingly, these compounds caused cell growth inhibition by a cytostatic rather than cytotoxic mechanism, as evidenced by the YOYO-1 cell labelling (images not shown). In addition, whereas the potency of compounds **32** and **35** to inhibit cell growth was similar to 4-OHTAM (IC_50_ = 5.54 ± 0.81 μM) in T47D cells (Figure 6A), it was relatively higher (IC_50_ = 5.53 ± 0.36 μM) in MCF-7 cells (Figure 6D). However, both compounds delayed their effects on cell growth until 48–72 h after treatment, whereas the effects of 4-OHTAM were faster. This cytostatic condition with slow but durable antiproliferative activity is generally associated with strong connections between compounds and targets or posttranscriptional and posttranslational effects after binding [52,53].

**Figure 7:**
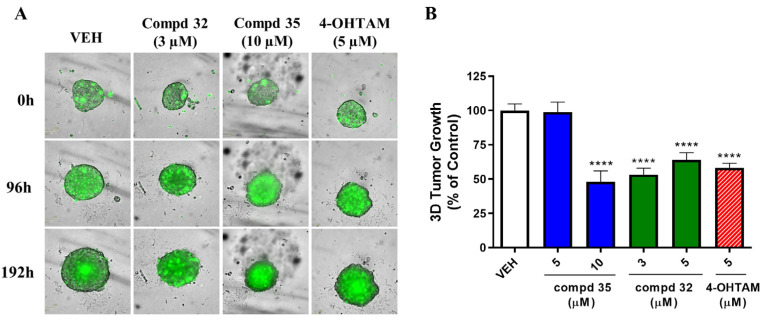
5-Hydroxy-2*H*-pyrrol-2-ones **32** and **35** prevent ER+ breast cancer cell spheroids growth. T47D tumor spheroids were treated with vehicle (VEH, 0.05% DMSO) or different doses of compounds (1–10 µM) for 8 days. (**A**) Representative brightfield microphotographs of YOYO-1-labeled tumor spheroids in the absence (VEH) or presence of compounds (compd **32**, 3 µM; compd **35**, 10 µM; and 4-OHTAM, 5 µM) at 0h, 96h and 192 h. (**B**) 3D-tumor growth quantification as mean ± SEM of the percentage (%) of tumor growth of each treatment with respect to untreated cells (VEH). Figures are representative of at least 3 independent experiments, each performed in triplicate. Statistical significance was assessed using one-way ANOVA with the Bonferroni post hoc test. **** *p* < 0.0001 vs. VEH-treated cells.

Since cell spheroids represent tumor cell biology better than 2D cultured cells [54], the effects of compounds **32** and **35** on the growth and viability of ER+ BC cell spheroids were monitored by sequential real-time microphotographs for 8 days. Remarkably, both treatments reduced the growth of T47D cancer cell spheroids (Figure 7); these results demonstrate that compounds **32** and **35** exhibit potent growth inhibition on 2D and 3D ER+ BC cell growth. A cytostatic mechanism, comparable to that observed with doses of 4-OHTAM lower than 10 μM, is also suggested by these data. However, whereas compound **32**, such as 4-OHTAM, caused a homogeneous reduction of spheroid cell viability, compound **35** induced irregular damage of the spheroid morphology; these findings also contribute to highlight that compounds **32** and **35** differ in their respective molecular mechanism of antitumoral effect. 

### 3.6. 5-hydroxy-2H-pyrrol-2-ones 32 and 35 Inhibit E2-Dependent Growth of Breast Cancer Cells

**Figure 8:**
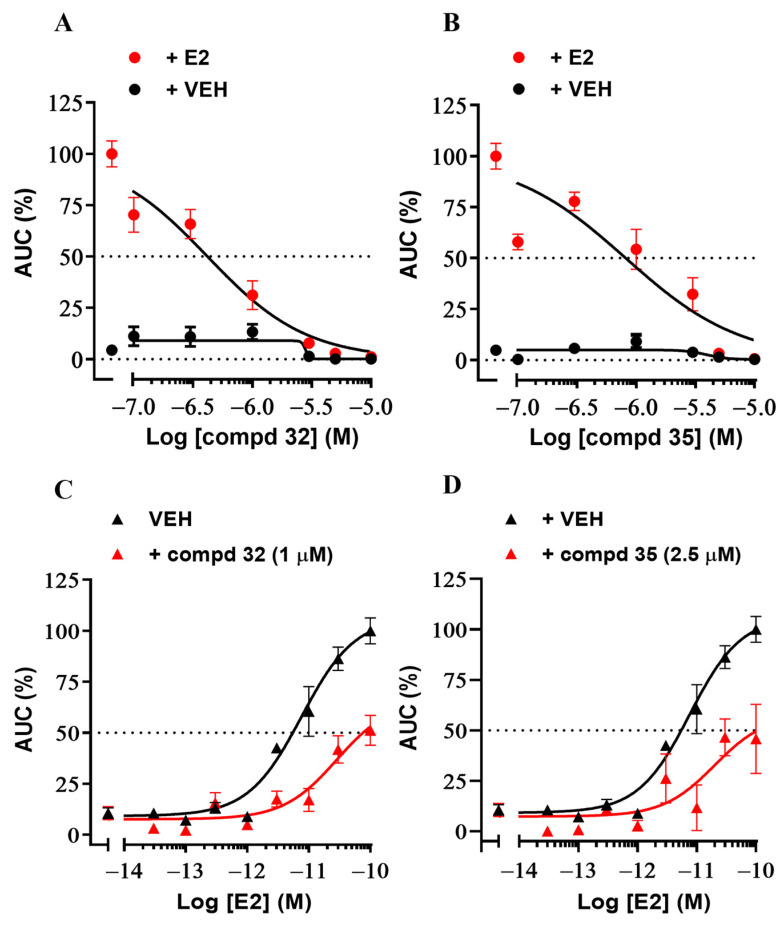
5-Hydroxy-2*H*-pyrrol-2-ones **32** and **35** exhibit antiestrogenic effects on E2-induced growth of ER+ breast cancer cells. (**A**,**B**) E2-depleted T47D cells were treated with vehicle (VEH, 0.05% DMSO), compound (**A**) **32** or (**B**) **35** (both, 0.01–10 µM) in the absence (VEH, ●) or presence of E2 (0.1 nM, ●) for 10 days. Cell growth kinetics were calculated by the area under the curve (AUC) for compounds alone (+VEH) or compounds+E2. Dotted lines are shown at 0% and 50% of AUC. Non-linear regression analysis was applied with GraphPad Prism software 8.4.3 to calculate EC_50_ or IC_50_ values. Data are expressed as mean ± SEM for at least three independent experiments, where each treatment was tested in triplicate. (**C**,**D**) Cells were treated with dose-response curves of E2 (0.03 pM–100 pM) alone (+ VEH, 0.05% DMSO, ▲) or in the presence of a constant concentration of compound (**C**) **32** (1 µM, ▲) or (**D**) **35** (2.5 µM, ▲) for 10 days. Cell growth kinetics were calculated by the AUC for E2+VEH or E2+compounds. Then non-linear regression analysis was applied to calculate their respective EC_50_ values.

Besides the inhibitory effect of the two selected 5-hydroxy-2*H*-pyrrol-2-ones in ER-dependent transcriptional activity and cell growth, the potential of compounds **32** and **35** in modulating E2-dependent growth of ER+ BC cells was assessed. Noteworthy, in E2-depleted conditions, these compounds did not increase the growth of ER+ BC cells, thus indicating they lacked estrogenic effects (Table 4; Figure 8). Nonetheless, compound **32** (IC_50_ = 0.42 ± 0.13 μM) (Figure 8A) and compound **35** (IC_50_ = 0.81 ± 0.46 μM) (Figure 8B) exhibited concentration-dependent inhibition of E2-induced growth of T47D cells. 

In order to further characterize the antiestrogenic activity of these compounds, a comprehensive dose-response analysis for the effects of E2 on T47D cell growth was carried out in the absence or in the presence of a constant concentration of compound **32** or **35** (Figure 8). As expected from their anticipated antiestrogenic effects, the potency of E2 (EC_50_ = 7.10 pM) was significantly reduced by compound **32** (to EC_50_ = 0.11 nM) (Figure 8C) and compound **35** (to EC_50_ = 0.15 nM) (Figure 8D). In addition, both lead compounds prevented E2 from restoring its maximum efficacy.

### 3.7. 5-hydroxy-2H-pyrrol-2-ones 32 and 35 Inhibit E2-Dependent Induction of ERα-Regulated Genes in ER+ Breast and Endometrial Cancer Cells

**Figure 9:**
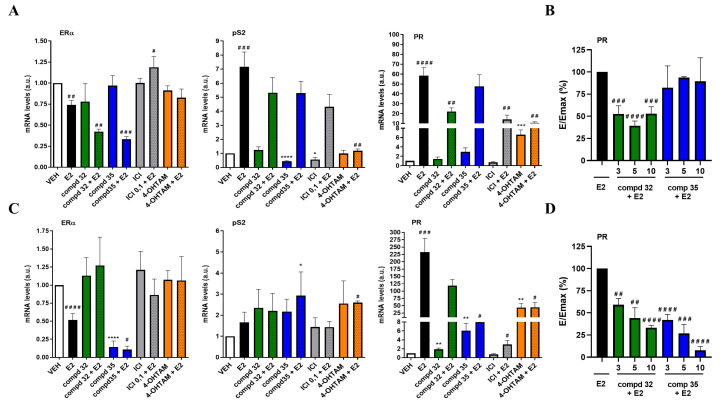
5-Hydroxy-2*H*-pyrrol-2-ones **32** and **35** exhibit antiestrogenic effects on E2-induced gene expression in human ER+ breast and endometrial cancer cells. ERα, pS2 and PR mRNA gene expression were analyzed by quantitative real-time PCR (q-RT-PCR) using 18S as a housekeeping gene in E2-depleted MCF-7 or Ishikawa cells. Cells were pretreated with compounds **32**, **35**, ICI or 4-OHTAM for 3 h, before the addition of vehicle (VEH; 0.05% DMSO) or E2 (10 nM) for 24 h. (**A**) MCF-7 and (**C**) Ishikawa cells were treated with compounds **32** (5 µM), **35** (10 µM), ICI (0.1 µM) or 4-OHTAM (1 µM) in the absence (+VEH) and presence of E2. Graphs represent relative mRNA levels versus VEH-treated cells. Statistical significance was assessed by comparing the means of each treatment (compound+VEH) using a two-tailed T-Student. * *p* < 0.05; ** *p* < 0.01; *** *p* < 0.001; **** *p* < 0.0001 versus VEH gene expression levels or for compound+E2 comparisons, # *p* < 0.05; ## *p* < 0.01; ### *p* <0.001; #### *p* < 0.0001 vs. E2 induced gene expression. (**B**) MCF-7 and (**D**) Ishikawa cells were treated with E2 (10 nM) or increasing doses of compounds **32** or **35** (3–10 µM) + E2, and PR gene expression was evaluated in terms of efficacy (E), calculating E/Emax (%) according to E2 Emax (100%). Statistical significance was assessed by using one-way ANOVA with the Bonferroni post hoc test. ## *p*<0.01; ### *p*<0.001; #### *p* < 0.0001 vs. E2-treated cells.

Estrogenic effects on the endometrium and ovary are one of the major limitations in the identification of new SERMs for the treatment of ER+ BC since they increase the risk of developing endometrial cancer. Indeed, this clinical challenge turns up in women treated with TAM or its metabolites [55]. For this reason, the validation of the antiestrogenic effects of potential new compounds in endometrial models is currently a mandatory requirement [17,56]. In this sense, Ishikawa cells are positively regulated by estrogens, thus representing an excellent option to identify potential agonistic and antagonistic properties of new compounds [17,57]. As mentioned above, 4-OHTAM inhibits ER+ BC cell growth but can, in turn, increase the risk of developing endometrial cancer, which is linked to its partial estrogenic effects in this tissue [12,16,17]. The analysis of the gene expression experiments reported that the ER antagonists 4-OHTAM or ICI kept ERα gene expression intact and blocked E2-induced mRNA levels of PR and pS2, two ERα-target genes in MCF-7 (Figure 9A,B) and Ishikawa (Figure 9C,D) cancer cells. 5-Hydroxy-2*H*-pyrrol-2-one compounds **32** and **35** inhibited E2-induced ERα, pS2 and PR mRNA expression in MCF-7 (Figure 9A) and compound **35** notably downregulated E2-induced ERα and PR mRNA levels in Ishikawa cells (Figure 9C,D). Compound **32** exhibited a significative reduction of PR-activated gene expression in both breast (Figure 9B) and endometrial cancer cells (Figure 9D); these findings support the antiestrogenic effects of both derivatives in breast and endometrial cancer cells, probably through two different mechanisms. According to 4-OHTAM partial agonism in Ishikawa cells, the basal level of mRNA PR gene was induced 44-fold by its treatment when added in the absence of E2; this finding supports its partial estrogenic effect on endometrial tissue. However, the basal mRNA levels of the PR gene in Ishikawa cancer cells (Figure 9C) were induced 2.5- and 5-fold by compounds **32** and **35**, respectively. This partial agonism, although less intense than 4-OHTAM, suggests that compounds **32** and **35** can exert relatively weak estrogenic transcriptional activity in endometrial cancer cells.

**Figure 10:**
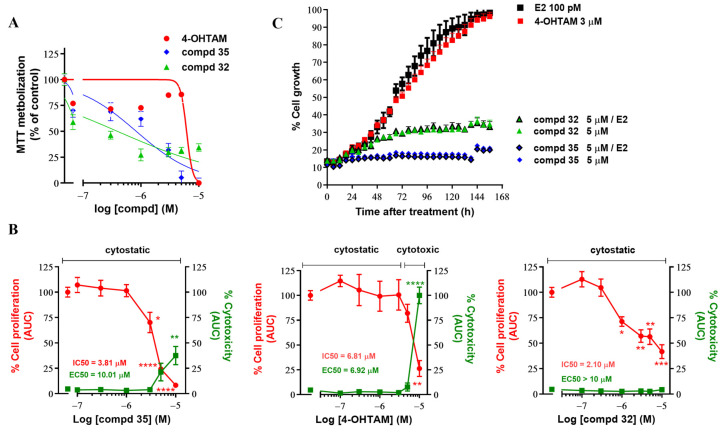
5-Hydroxy-2*H*-pyrrol-2-ones **32** and **35** inhibit ER+ endometrial cancer cell viability and proliferation in both serum-completed and E2-depleted media. Ishikawa cells were treated with vehicle (VEH, 0.05% DMSO), 4-hydroxy-tamoxifen (4-OHTAM; 0.01–10 µM) or compounds **32** or **35** (0.01–10 µM) in serum-completed media (10%FBS). (**A**) Cell viability was assessed after 72 h treatment by MTT assays as described in Materials and Methods. (**B**) Cell proliferation and cytotoxicity were assessed after 5 days monitorization. Data represent the area under the curve (AUC) by compounds dose in terms of proliferation in phase contrast (left *Y*-axis) and green-fluorescent cytotoxicity (right *Y*-axis). The mean ± SEM values are represented for each dose, comparing proliferation with respect to the maximum cell growth of VEH cells and the maximum cytotoxicity induced by 4-OHTAM at the highest dose. Data are expressed as mean ± SEM from at least triplicate independent experiments, where each compound concentration was assessed in triplicate. Statistical significance was assessed using one-way ANOVA with the Bonferroni post hoc test. * *p* < 0.05, ** *p* < 0.01, *** *p* < 0.001, **** *p* < 0.0001 vs. VEH-treated cells. (**C**) E2-depleted Ishikawa cells were treated with E2 (0.1 nM, ■), compound **32** (5 µM, ▲), compound **35** (5 µM, ◆) or 4-OHTAM (3 µM, ■) (in the absence (VEH) or presence of E2 for 7 days. Cell growth kinetics were represented, and data were expressed as mean ± SEM for at least two independent experiments, where each treatment was tested in triplicate.

To better characterize the role of the two selected compounds in the endometrium, their effects on ER+ endometrial cancer cell viability and growth were explored (Figure 10). Interestingly, compound **32** (IC_50_ = 0.27 ± 0.09 µM) and **35** (IC_50_ = 1.29 ± 0.38 µM) caused potent cell viability inhibition of serum-completed media cultured Ishikawa cells, compared to 4-OHTAM (IC_50_ = 6.16 ± 0.80 µM) (Figure 10A). A real-time study of cell growth reported the antitumoral effect of both compounds that appears to be caused by a cytostatic mechanism (Figure 10B). Considering its partial agonism, lower doses of 4-OHTAM (3 μM) increased the growth of E2-depleted Ishikawa cells, whereas this effect was not observed with compounds **32** and **35** (Figure 10C).

**Figure 11:**
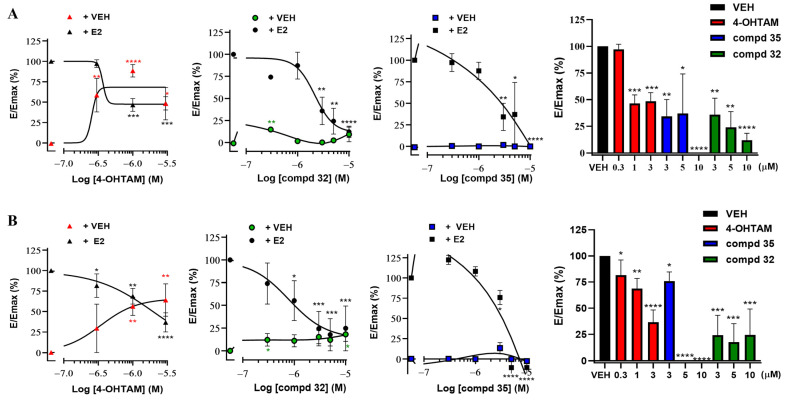
5-Hydroxy-2*H*-pyrrol-2-ones **32** and **35** exhibit antagonistic effects on E2-induced alkaline phosphatase enzymatic activity (ALP) in a time and dose dependent-manner in ER+ endometrial cancer cells. E2-depleted Ishikawa cells were treated with vehicle (VEH, 0.05% DMSO), 4-OHT (0.3–3 µM), compounds **32** or **35** (both, 0.3–10 µM) in the absence (+VEH) or presence of E2 (10 nM) for (**A**) 48 h and (**B**) 72 h. The left panels show dose-response curves of the studied compounds, which are represented by plotting the decimal logarithm of the concentration of the compound against the E/Emax (%), calculated from the Emax induced with E2. Doses of compounds in the absence of E2 (+VEH) are shown as ▲ for 4-OHTAM, ● for compound **32**, and ∎ for compound **35**, while doses of products + E2 (10 nM) are represented as ▲, ●, ∎, respectively. The right panels represent bar diagrams of the E/Emax (%) of compound doses + E2 (10 nM), calculated from the Emax (100%) induced by E2 after the corresponding treatments and times. Statistical significance was assessed using one-way ANOVA with Bonferroni post hoc test using GraphPad Prism software 8.4.3. * *p* < 0.05; ** *p* < 0.01; *** *p* < 0.001; **** *p* < 0.0001 versus E2-stimulated cells. Data are expressed as mean ± SEM for at least two independent experiments, where each treatment was tested in triplicate.

E2 positively regulates alkaline phosphatase (ALP) gene expression and activity in Ishikawa cells, and this effect represents a reliable biomarker of ERα activation [17,57]. In our experiments, ALP activity was 30-fold increased by E2 in Ishikawa cells (Figure 11). Interestingly, in the absence of E2, 4-OHTAM and compound **32** increased ALP activity until they reached 88% and 18% of E2-induced maximal activity (i.e., Emax), respectively; these results confirm the previously reported uterotrophic effects attributed to 4-OHTAM [55,58]. One of the most accepted hypotheses to explain these effects refers to the recruitment of the transcriptional factor NCOA-1, with uterotrophic properties [15,58]. In fact, it has been recently identified that 4-OHTAM acts as an agonist of GPER in endometrium and stimulates endometrial cells through a mechanism that may involve MAPK phosphorylation [59]. In contrast, ICI and raloxifene are ER antagonists in the endometrium since they do not induce NCOA-1 recruitment, nor do they agonize with GPER [59].

However, both 4-OHTAM and compound **32** inhibited E2-induced ALP activity after 48 h (Figure 11A) and 72 h (Figure 11B) treatment, which supports they exert partial antagonism in the endometrium. Nevertheless, compound **35** did not affect basal (i.e., in the absence of E2), but inhibited E2-induced ALP activity (Figure 10) thus suggesting that compound **35**, unlike 4-OHTAM and compound **32**, is a pure antagonist in endometrial cancer cells. Taken together, these results contribute to the characterization of compounds **32** and **35** as potent antitumoral drugs in ER+ cancer cells.

### 3.8. 5-hydroxy-2H-pyrrol-2-ones 32 and 35 Block Cell Cycle Entry and Induce Apoptosis in ER+ BC Cells

**Figure 12:**
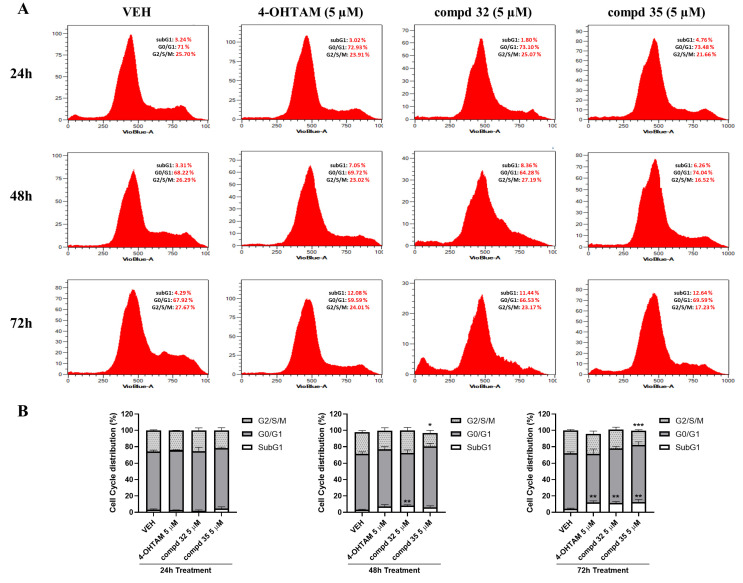
5-Hydroxy-2*H*-pyrrol-2-ones **32** and **35** block cell cycle entry in human ER+ breast cancer cells in a time-dependent manner. Cell cycle studies were performed in T47D cells treated with vehicle (VEH, 0.05% DMSO), 4-OHT (5 µM), compounds **32** or **35** (both, 5 µM) for 24 h, 48 h or 72 h. (**A**) Representative FACS images with the profiles of the cell cycle phases (subG1, G0/G1 and G2/S/M). (**B**) Quantification of the percentages of cells in each phase of the cycle for each treatment and time-point shown in panel A. Graphs are representative of at least three independent experiments, each performed in triplicate. Statistical significance was assessed by means comparisons of each treatment condition using a two-tailed T-Student. * *p* < 0.05; ** *p* < 0.01; *** *p* < 0.001 vs. VEH-treated cells in each phase of the cell cycle.

**Figure 13:**
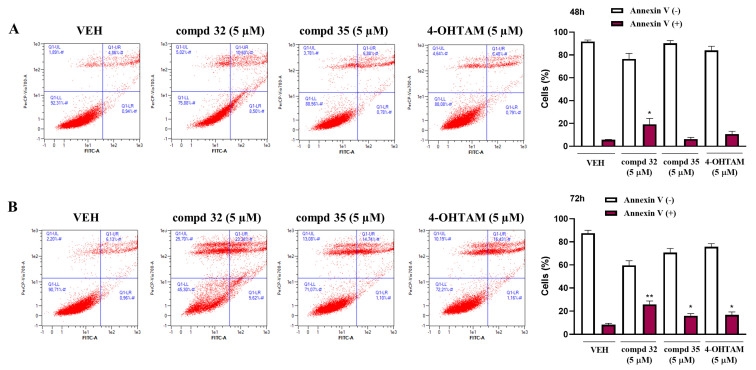
5-Hydroxy-2*H*-pyrrol-2-ones **32** and **35** induce apoptosis in human ER+ breast cancer cells in a time dependent-manner. Apoptosis was measured in T47D cells treated with vehicle (VEH, 0.05% DMSO), 4-OHT (5 µM), compounds **32** or **35** (both, 5 µM) for 48 h (**A**) or 72 h (**B**) using the Annexin V-FITC/Propidium Iodide (PI) apoptosis detection kit. (**A**,**B**,**Left panel**) Representative FACS images of phosphatidylserine translocation to the cell surface measurements, distinguishing viable cells (lower left quadrant, Q1-LL), cells in early apoptosis (lower right quadrant, Q2-LR) and cells in an advanced death phase (upper right quadrant, Q3-UR). (**A**,**B**,**Right panel**) Quantification of the percentages of viable Annexin V (–) (white bars) versus apoptotic Annexin V (+) (purple bars) cells for each treatment and time-point shown in left panel. Graphs are representative of at least three independent experiments, each performed in triplicate. Statistical significance was assessed by mean comparisons of each treatment condition using a two-tailed T-Student. * *p* < 0.05; ** *p* < 0.01 versus VEH-treated cells in each Annexin V group at each time-point.

To determine whether the effect of compounds on growth inhibition of ER+ BC cells was associated with cell cycle arrest and/or apoptosis, flow cytometry analyses were performed after treatment of T47D cells with compounds **32** and **35**. Interestingly, both molecules arrested the T47D cell cycle in a time and dose-dependent manner. Remarkably, compounds **32** and **35** significantly increased the percentage of cells in sub-G1 and G0/G1 phases after 48 h and 72 h of treatment (Figure 12). In addition, compound **35** decreased the G2/S/M phase level (from 26.29% to 16.52%) compared to non-treated cells after 48 h (Figure 12), and these differences were increased after 72 h treatment. When cell cycle blockade triggers an increase in sub-G1 cells, programmed cell death phenomena are probably involved in the presence of haploid or death cells [60]. Therefore, the evaluation of compounds regarding their ability to modulate apoptosis is essential for setting up their antitumoral capacity, since cancer cells can elude apoptosis in favor of their survival [61]. In this sense, the inhibition of compounds **32** and **35** on cell growth and cell cycle was correlated with a time-dependent increase of apoptotic T47D cancer cells, as shown in Figure 13.

### 3.9. 5-hydroxy-2H-pyrrol-2-ones 32 and 35 Decrease ERα Protein Level

**Figure 14:**
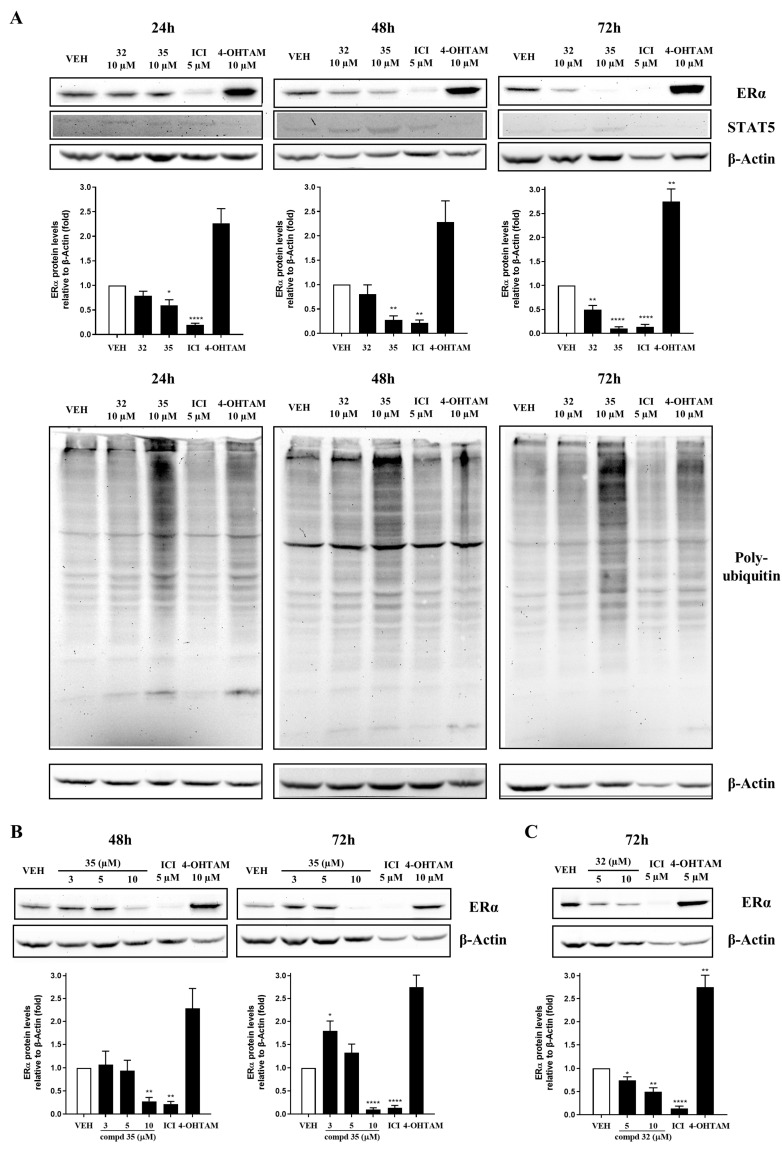
5-Hydroxy-2*H*-pyrrol-2-ones **32** and **35** decrease ERα protein levels in human ER+ breast cancer cells in a time and dose dependent-manner by two differentiated mechanisms. Immunoblotting detection of ERα (66 kDa isoform), total STAT5 and polyubiquitin from whole-cell extracts of T47D cells treated with vehicle (VEH, 0.05% DMSO), 4-OHTAM (10 μM), ICI (5 μM), compound **32** or **35** (3–10 μM) for 24 h, 48 h or 72 h. β-actin was used as a loading control for the experiments. Representative images from three independent experiments are shown. (**A**) Representative Western blot images for ERα, STAT5 and polyubiquitin after a dose-time study of relative protein expression. (**B**) Representative Western blot images for ERα, in a dose-response study with compound **35** after 48 h and 72 h treatment. (**C**) Representative Western blot images for ERα, in a dose-response study with compound **32** after 72 h treatment. Densitometric quantification from immunosignal values (mean ± SEM) of ERα relative to β-Actin (VEH: fold 1) is shown for each treatment and time studied. Statistical significance was assessed by the mean comparisons of each treatment condition using a two-tailed T-Student. * *p* < 0.05; ** *p* < 0.01 and **** *p* < 0.0001 versus VEH-treated cells at each time-point.

The hyperactivation of ERα is the main physiopathological feature of ER+ BC as its estrogen-mediated aberrant activation arranges the growth and survival of cancer cells [7,62]. Our immunoblot studies revealed that, as expected, the pure antiestrogen ICI [5,8,10] caused a rapid (from 3h) and steady reduction of ERα protein level in E2-non depleted T47D cells after 24, 48 and 72 h treatment; however, it was increased by 4-OHTAM (Figure 14), coinciding with the existing literature [49]. Similarly, compound **35** caused a time (Figure 14A) and dose-dependent (Figure 14B) decrease of ERα protein content, whereas compound **32** needed a longer time of cell exposure (Figure 14A,C). In contrast, whereas 4-OHTAM reduced the total STAT5 protein level, this effect was not provoked by compounds **32** and **35** (Figure 14A). Interestingly, compound **35** treatment significantly increased polyubiquitinated protein level in T47D cells from 24 h to 72 h (Figure 14D), thus suggesting it holds a mechanism of activation of proteasomal degradation similar to ICI [63,64] or bazedoxifen [49] through 26S subunit [65]. However, these results require further validation using proteasome inhibitors [66]. If positive, these data would show that compound **35** may emerge as a new potential SERD with an interesting biological profile in ER+ breast cancer that competes against E2 and blocks ERα gene and protein expression by promoting its proteasomal degradation. 

To confirm previous data, the effects of compound **35** on ERα protein expression were also explored in T47D E2-deprived BC cells by using confocal microscopy (Supplementary Figure S4). As expected, E2 caused maximal translocation of ERα in cell nuclei after 30 min. In contrast, compound **32** showed no effects, whereas compound **35** significantly decreased ERα protein levels both in nuclei and cytoplasmic compartments. Interestingly, compound **35**, in the absence as well as in the presence of E2, decreased ERα mRNA levels in ER+ BC cells (Figure 9A). In contrast to the mechanism of 4-OHTAM, both protein polyubiquitination and loss of ERα contribute to the antiestrogenic mechanism of compound **35** in ER+ BC cells; these results, together with the low affinity to ERα revealed in the binding assays, lead to hypothesize that proteins involved in the regulation of proteasomal degradation could be potential targets of compound 35. However, if protein polyubiquitination induced by compound **35** contributes to ERα protein turnover through proteasome activation still deserves further research. Full, uncropped immunoblot images are shown in Supplementary Figure S5.

### 3.10. 5-hydroxy-2H-pyrrol-2-ones 32 and 35 Potentiate Antitumoral Effect of 4-OHTAM on ER+ Breast Cancer Cells

**Figure 15:**
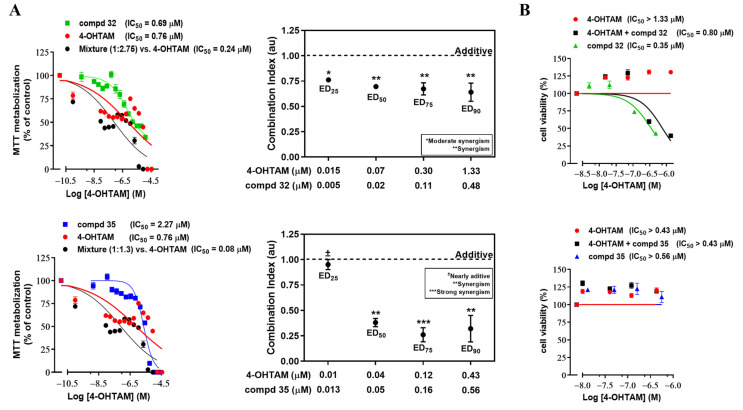
A combination of 5-hydroxy-2*H*-pyrrol-2-ones **32** and **35** with 4-OHTAM promote synergistic antitumoral effects in human ER+ breast cancer cells with scarce or null effects in human non-malignant breast cells. (**A**) Cell viability inhibition was measured in MCF-7 cells. **Left panel**: MCF-7 cells were treated with compounds **32** (∎; 0.0004–12.8 µM), **35** (∎; 0.0014–22.4 µM) 4-OHTAM (●; 0.001–35.2 µM) or with the respective combination of compound-4-OHTAM (● mixture) maintaining a constant ratio [compound **32**:4-OHTAM (1:2.75)] or [compound **35**:4-OHTAM (1:1.3)] for 72 h. **Right panel**: Isobologram analysis (determination of the combination index, CI) of the cell viability data graphed in the left panel. The effects ranged from 0 (no cell viability inhibition) to 1 (100% viability inhibition) and were analyzed using Calcusyn^®^ software, as described in the Material and Methods. Data are representative of two independent experiments, where each treatment was tested in four replicates. CI values (mean ± SEM) for ED_25_, ED_50_, ED_75_ and ED_90_ express additive (±) or different grades of synergistic (*, **, ***) effects. (**B**) Cell viability inhibition was measured in MCF-10A cells. Cells were treated with: compound **32** (▲; 0.005–0.48 µM), 4-OHTAM (● ; 0.015–1.33 µM) or its combination; or compound **35** (▲ ; 0.013–0.56 µM), 4-OHTAM (● ; 0.01–0.43 µM) or its combination, according to the CI values (mean ± SEM) for ED_25_, ED_50_, ED_75_ and ED_90_ calculated in panel A. Cell viability were assessed after 72 h treatment by MTT assays as described in Materials and Methods. Non-linear regression analysis was applied with GraphPad Prism software 8.4.3 to calculate IC_50_ values.

Another historically posed challenge linked to the clinical use of 4-OHTAM and other SERMs is the generation of endocrine resistance mainly due to the use of high doses or prolonged treatments [7,65]. In this sense, drug combination may allow the reaching of synergistic pharmacological effects that reduce drug toxicity, resistance and/or increase drug efficacy [43,67]. Hence, to determine whether compounds **32** and/or **35** enhance the antitumoral effects of 4-OHTAM in ER+ BC cells, dose-response synergistic assays combining each compound with 4-OHTAM were performed (Figure 15A). For this purpose, MCF-7 cells were incubated (72 h) with a broad range of compound **32** or compound **35** and 4-OHTAM doses, maintaining a constant ratio combination design [compound **32**:4-OHTAM (1:2.75)] and [compound **35**:4-OHTAM (1:1.3)]. Interestingly, we observed that both combinations potentiated the inhibitory effects induced by 4-OHTAM on MCF-7 cell viability (from 3 to 10-fold) compared to 4-OHTAM alone (Figure 15A). Accordingly, isobologram and Chou-Talalay analyses of the combination index (CI) [43] showed that combinations of compound **32** (CI for ED_25_ = 0.762 ± 0.02; CI for ED_50_ = 0.696 ± 0.01; CI for ED_75_ = 0.6725 ± 0.06; CI for ED_90_ = 0.6395 ± 0.09) or compound **35** (CI for ED_50_ = 0.38 ± 0.04; CI for ED_75_ = 0.26 ± 0.07; CI for ED_90_ = 0.32 ± 0.13) with 4-OHTAM acted synergistically in promoting antitumoral effects (Figure 15A); these compound combinations were also studied in non-malignant MCF-10A breast cells (Figure 15B). Interestingly, data showed that the compound **32**/4-OHTAM combination affected MCF-10A cell viability to the same extent as compound **32** alone, while this effect was not observed for the compound **35**/4-OHTAM combination (Figure 15B). Therefore, the combinatorial evaluation showed that compound **35** could potentiate the antitumoral effects of 4-OHTAM which might enhance its efficacy on ER+ BC cells, thus entailing a very relevant clinical advance.

### 3.11. In Silico ADME Predictions of 5-hydroxy-2H-pyrrol-2-ones

In terms of pharmacological research and drug development, unfavorable pharmacokinetic profiles are often responsible for the failure of many drug candidates. Thereafter, the incorporation of these predictive parameters in the selection of new potential drugs is considered a relevant step. Properties such as drug-likeness, permeability, solubility, bioavailability, and oral absorption provide insights into key aspects for the development of new drugs [32,68]. Thus, compounds **32** and **35** were further explored by predictions of pharmacokinetic properties in order to understand their pharmacokinetic profiles. ADME properties were calculated using the Qikprop program, which provided predicted values for physically and pharmaceutically relevant parameters and its recommended range of values (Supplementary Table S2). 

As a first test of the drug-likeness of the ligands, we applied Lipinski’s rule of 5 [32]. As can be seen, the partition coefficient (QPlogPo/w), the hydrogen bond donors (HB donor), the hydrogen bond acceptors (HB acceptor), the molecular weight (mol. wt.), and the human oral absorption percentage exhibited satisfactory values. Importantly, the aqueous solubility of compounds **32** and **35**, which is a crucial property for its bioavailability, was satisfactory with respect to their QPlogS values. The high absorption and permeability of the compounds were confirmed by the non-violation of any of Lipinski’s rules [32], by the high values for parameters concerning cell permeability as blood–brain barrier mimics MDCK cell permeability (QPPMDCK), and by the predicted Caco-2 cells permeability (QPPCaco) used as a model for the gut–blood barrier. QPlogKhsa is the prediction of binding to human serum albumin, and the compounds lie within the expected range for 95% of known drugs. The QPlogBB (brain/blood) barrier coefficient was satisfactory for the most active compounds. As shown in Supplementary Table S2, 5-hydroxy-2H-pyrrol-2-ones **32** and **35** exhibited very good drug-likeness, as well as meeting all the pharmacokinetic criteria, thus may be considered potential candidate leads.

## 4. Conclusions

Collectively, the results from this study identify novel 5-hydroxy-2*H*-pyrrol-2-ones that exert antiestrogenic activities on ER+ breast and endometrial cancers. Notably, within the chemical library, compound **32** works as a partial antagonist, whereas compound **35** is a potent pure antagonist that provokes protein polyubiquitination, ERα downregulation and cell cycle arrest of ER+ BC cells. Although both compounds modulate the expression of ERα, they show low binding affinity to the receptor. Therefore, they might be targeting other molecules to exert their effects. Clinically relevant, the absence of agonistic activity by compound **35** in endometrial cells might prevent pro-tumoral effects linked to partial agonism of current SERMs, besides potentiating the antiestrogenic effects of 4-OHTAM on ER+ BC cells. Both compounds **32** and **35** displayed good ADME values, highlighting those related to permeability, toxicity, and protein-plasma interactions; these predictions, linked to the values of biological activity, are promising and, therefore, deserve deeper investigation for further optimization of these novel 5-hydroxy-2*H*-pyrrol-2-one compounds.

## Figures and Tables

**Table 1:** Effects of the most active 5-hydroxy-2*H*-pyrrol-2-ones on the viability of ER breast cancer (ER+ and ER−), ER+ endometrial and non-malignant cells. Cells were treated with vehicle (VEH, 0.05% DMSO), doxorubicin (DOXO; 0.01–5 µM), ICI 182,780 (ICI; 0.01–50 µM), 4-hydroxy-tamoxifen (4-OHTAM; 0.01–10 µM) or compounds (0.01–10 µM) for 72 h in the presence of 10%FBS growth media. The percentage of cell viability related to the VEH-treated cells was assessed by MTT assay as described in Materials and Methods. The calculated IC_50_ values are expressed as mean ± SEM from at least duplicate independent experiments, where each compound concentration was assessed in triplicate; nd indicates a non-determined IC_50_ value.

	ER+ Cells				ER− Cells				Non-Malignant Cells		
	MCF-7	MCF-7/BUS	T47D	ISHIKAWA	SK-BR-3	BT-549	Hs-578T	MDA-MB-231	MCF-10A	VERO	PBMC
ID Compound	IC_50_ (μM)	IC_50_ (μM)	IC_50_ (μM)	IC_50_ (μM)	IC_50_ (μM)	IC_50_ (μM)	IC_50_ (μM)	IC_50_ (μM)	IC_50_ (μM)	IC_50_ (μM)	IC_50_ (μM)
	Mean ± SEM	Mean ± SEM	Mean ± SEM	Mean ± SEM	Mean ± SEM	Mean ± SEM	Mean ± SEM	Mean ± SEM	Mean ± SEM	Mean ± SEM	Mean ± SEM
**DOXO**	0.07 ± 0.01	0.05 ± 0.02	0.15 ± 0.02	0.09 ± 0.03	0.04 ± 0.01	0.19 ± 0.04	0.37 ± 0.05	0.25 ± 0.07	0.09 ± 0.01	2.62 ± 0.24	1.47 ± 0.69
**4-OHTAM**	4.43 ± 1.03	0.09 ± nd	5.9 ± 0.76	6.16 ± 0.8	7.85 ± 1.95	>10 ± 0	>10 ± 0	>10 ± 0	5.3 ± 0.28	10.79 ± 0.13	nd ± nd
**ICI**	0.52 ± 0.16	0.0006 ± 0	14.91 ± 5.19	>10 ± 0	>50 ± 0	>25 ± 0	>25 ± 0	>25 ± 0	>10 ± 0	>10 ± 0	nd ± nd
**4**	>10 ± nd	nd ± nd	>10 ± 0	>10 ± 0	>10 ± nd	nd ± nd	>10 ± nd	>10 ± nd	7.17 ± 2.07	>25 ± 0	nd ± nd
**9**	>10 ± nd	>10 ± 0	>10 ± 0	nd ± nd	>10 ± nd	>10 ± nd	>10 ± nd	>10 ± 0	nd ± nd	nd ± nd	nd ± nd
**16**	>10 ± nd	>10 ± 0	>10 ± 0	>10 ± 0	>10 ± nd	>10 ± nd	>10 ± nd	>10 ± nd	nd ± nd	nd ± nd	nd ± nd
**26**	>10 ± nd	nd ± nd	>10 ± 0	10.11 ± 0.11	>10 ± nd	nd ± nd	>10 ± nd	9.71 ± 0.3	nd ± nd	nd ± nd	nd ± nd
**32**	2.54 ± 0.49	5.32 ± 2.09	10.64 ± 0.58	0.27 ± 0.09	1.51 ± 0.09	4.89 ± 0.96	>10 ± 0	7.63 ± 2.38	0.08 ± 0	14.74 ± 1.31	>30 ± 0
**35**	2.59 ± 0.34	2.64 ± 0.88	5.31 ± 0.47	1.29 ± 0.38	2 ± 0.18	4.19 ± 0	8.22 ± 1.78	9.08 ± 0.93	3.16 ± 0.09	9.61 ± 0.16	17.26 ± 6.48
**38**	>10 ± nd	nd ± nd	>10 ± 0	>10 ± 0	>10 ± nd	nd ± nd	>10 ± nd	>10 ± nd	>10 ± 0	>25 ± 0	nd ± nd
**43**	6.54 ± 0.41	2.85 ± 1.24	8.57 ± 0.43	3.47 ± 0.94	2.16 ± 0.05	6.23 ± 3.48	>10 ± nd	>10 ± 0	nd ± nd	nd ± nd	nd ± nd
**49**	3.47 ± 1.12	5 ± 1.79	>10 ± 0	4.82 ± 1.84	>10 ± nd	>10 ± nd	>10 ± nd	7.94 ± 2.07	0.31 ± 0.03	nd ± nd	nd ± nd
**50**	6.17 ± 2.66	nd ± nd	nd ± nd	nd ± nd	>10 ± nd	nd ± nd	nd ± nd	nd ± nd	nd ± nd	nd ± nd	nd ± nd

**Table 2:** Chemical screening of representative 5-hydroxy-2*H*-pyrrol-2-ones on ER-dependent transcriptional activity. T47D-KBluc cells were seeded in E2-depleted growth media (5%DCC-FBS) and pretreated with (A) compounds (10 µM) or with (B) a dose-response (1 µM–10 µM) for 3 h, before the addition of vehicle (VEH; 0.05% DMSO) or E2 (0.1 nM) for 16–24 h. ICI 182,780 (ICI; 0.03 –10 nM) and 4-hydroxy-tamoxifen (4-OHTAM; 0.03–1 µM) were used as antagonism controls. Relative Luciferase activity (RLU) was measured as described in the Material and Methods. The maximal luciferase activity or Emax (15.19 ± 1.71-fold induction) was induced by E2, and the efficacy (E) of each treatment, as compared with Emax, was calculated (E/Emax%). Non-linear regression analysis was applied with GraphPad Prism software 8.4.3 to calculate IC_50_ values. Data are expressed as mean ± SEM for at least three independent experiments, where each treatment was tested in triplicate; nd indicates a non-determined IC_50_ value.

	A		B
	VEH	E2	
ID Compound	E/Emax (%)	E/Emax (%)	IC_50_ (µM)
	Mean ± SEM	Mean ± SEM	Mean ± SEM
**E2**	99.98 ± 11.29	nd	nd
**4-OHTAM**	−7.14 ± 3.57	−10.62 ± 0.64	<0.03 ± 0
**ICI**	−9.15 ± 3.99	−9.89 ± 2.33	1.90 × 10^−4^ ± 3.00 × 10^−5^
**4**	0.01 ± 1.02	39.73 ± 14.82	3.4 ± 1.64
**5**	3.33 ± 1.32	111.82 ± 25.16	>10 ± 0
**8**	0.58 ± 1.79	68.75 ± 13.82	>10 ± 0
**10**	4.02 ± 2.12	92.97 ± 14.37	>10 ± 0
**16**	16.37 ± 7.86	83.04 ± 7.49	>10 ± 0
**20**	−0.29 ± 1.67	77.75 ± 27.24	>10 ± 0
**21**	−1.43 ± 1.53	119.51 ± 23.29	>10 ± 0
**23**	1.32 ± 3.15	77.59 ± 28.28	>10 ± 0
**25**	3.89 ± 3.73	70.34 ± 11.06	>10 ± 0
**26**	0.11 ± 2.78	49.7 ± 15.05	8.35 ± 1.35
**32**	−0.32 ± 0.73	105.29 ± 18.23	>10 ± 0
**35**	−11.82 ± 5.37	29.72 ± 9.92	8.2 ± 1.19
**37**	−0.67 ± 0.92	126.11 ± 21.36	>10 ± 0
**38**	−0.87 ± 3.05	61.65 ± 5.52	3.58 ± 2.4
**39**	1.94 ± 0.88	101.35 ± 0.27	>10 ± 0
**40**	−3.22 ± 1.32	31.89 ± 11.43	8.77 ± 0.59
**43**	−1.01 ± 2.56	172.56 ± 28.04	>10 ± 0
**46**	−6.02 ± 3.4	54.1 ± 13.33	6.54 ± 3.47
**49**	3.08 ± 4.84	97.95 ± 13.32	>10 ± 0
**50**	−0.16 ± 0.45	33.68 ± 11.2	nd ± nd

**Table 3:** Binding affinities of 5-hydroxy-2*H*-pyrrol-2-one compounds to rhERα. The binding of competitors (compounds **4**, **26**, **32**, **35**, **38**, **49** or controls: E2, DES or 4-OHTAM) to rhERα was evaluated in 10-point titration competition curves (0.1 nM–20 µM) by using the LanthaScreen TR-FRET competitive binding assay as described in the Material and Methods. Non-linear regression analysis was performed in GraphPad Prism 8.4.3 to calculate their IC_50_ values. Data are expressed as mean ± SEM from two independent assays where each concentration was tested in duplicate.

ID Compound	IC_50_ (µM)
	Mean ± SEM
**E2**	1.01 × 10^−4^ ± 3.16 × 10^−10^
**DES**	8.40 × 10^−5^ ± 4.00 × 10^−11^
**4-OHTAM**	7.98 × 10^−5^ ± 2.95 × 10^−12^
**4**	13.3 ± 4.66 × 10^−7^
**26**	18.2 ± 4.83 × 10^−8^
**32**	32.4 ± 7.67 × 10^−9^
**35**	27 ± 7.90 × 10^−8^
**38**	26.8 ± 6.38 × 10^−7^
**49**	30.6 ± 4.89 × 10^−8^

**Table 4:** Effects of 5-hydroxy-3,5-diaryl-1,5-dihydro-2*H*-pyrrol-2-ones on E2-induced proliferation of ER+ breast cancer cells. T47D estrogen-depleted cells were treated with vehicle (VEH, 0.05% DMSO), compounds (0.01–10 µM), ICI 182,780 (ICI, 0.03–10 nM), 4-hydroxy-tamoxifen (4-OHTAM, 0.01–10 nM) or 1,3,5-tris(4-hydroxyphenyl)-4-propyl-1*H*-pyrazole (PPT, 0.01–3 nM) in the absence (+VEH) or presence of E2 (0.1 nM) for 10 days. Cell growth kinetics were calculated by the area under the curve (AUC) for compounds alone (+VEH) or compounds+E2. Non-linear regression analysis was applied with GraphPad Prism software 8.4.3 to calculate EC_50_ or IC_50_ values. Data are expressed as mean ± SEM for at least three independent experiments, where each concentration was tested in triplicate; null indicates a non-defined IC_50_ or EC_50_ calculation.

	AUC (%)	
**ID Compound**	IC50 (μM)	EC50 (μM)
	Mean ± SEM	Mean ± SEM
**E2**	nd ± nd	7.10 × 10^−7^ ± 2.60 × 10^−7^
**PPT**	nd ± nd	1.57 × 10^−4^ ± 6.29 × 10^−5^
**4-OHTAM**	1.18 × 10^−4^ ± 0	null ± null
**ICI**	2.83 × 10^−4^ ± 1.12 × 10^−4^	null ± null
**4**	4.97 ± 0.35	null ± null
**9**	>10 ± 0	null ± null
**16**	2.86 ± 2.1	null ± null
**32**	0.42 ± 0.13	null ± null
**35**	0.81 ± 0.46	null ± null
**38**	4.27 ± 0.34	null ± null
**43**	3.95 ± 0.75	null ± null
**49**	1.89 ± 0.63	null ± null

## Data Availability

The datasets generated during and/or analyzed during the current study are available from the corresponding author upon reasonable request.

## Supplementary Materials

The following supporting information can be downloaded at: https://www.mdpi.com/article/10.3390/cancers14215174/s1, Figure S1: ^1^H-NMR and ^13^C-NMR spectra of compounds **4**–**50**; Figure S2: Effects of 5-hydroxy-2*H*-pyrrol-2-one compounds **32** and **35** on Androgen (AR) and Glucocorticoid (GR) Receptors-mediated transcription; Figure S3: Effects of 5-hydroxy-2*H*-pyrrol-2-one compounds **32** and **35** on STAT3 and STAT5-mediated transcription; Figure S4: Effects of 5-hydroxy-2*H*-pyrrol-2-one compounds **32** and **35** on ERα protein levels and location studied by immunofluorescence assays; Figure S5: Full uncropped immunoblot images; Table S1: Molecular docking study of the 5-hydroxy-3,5-diaryl-1,5-dihydro-2*H*-pyrrol-2-one compounds (**4**–**50**); Table S2: Computational pharmacokinetic parameters (predictive ADME) of the 5-hydroxy-3,5-diaryl-1,5-dihydro-2*H*-pyrrol-2-one enantiomers R and S of compounds **4**, **9**, **16**, **26**, **32**, **34**, **35**, **38**, **43**, **49**.

## Abbreviations

AIs, Aromatase inhibitors; AR, Androgen Receptor; BC, Breast Cancer; DES, Diethylstilbestrol; DEX, Dexamethasone; ER+, Estrogen Receptor-positive; E2, 17β-estradiol; GR, Glucocorticoid Receptor; 4-OHTAM, 4-Hydroxy-Tamoxifen; PAI-1, Plasminogen Activator Inhibitor-1; rhERα, recombinant human ERα; SERDs, Selective ERα Degraders; SERMs, Selective ERα Modulators; STAT, Signal transducer and activator of transcription; TAM, Tamoxifen; T, Testosterone; TNBC, Triple Negative Breast Cancer.
