# Supplementary material for: Discovery of Highly Functionalized 5-hydroxy-2H-pyrrol-2-ones That Exhibit Antiestrogenic Effects in Breast and Endometrial Cancer Cells and Potentiate the Antitumoral Effect of Tamoxifen
Source: Cancers (Basel). 2022 Oct 22;14(21):5174. doi: 10.3390/cancers14215174 (PMC9655618; doi:10.3390/cancers14215174)

Supplementary Figure S1. ^{1}H-NMR and ^{13}C-NMR spectra of compounds **4-50.**

^{1}H-NMR ($CDCl_3$, 600 MHz) of compound 4.

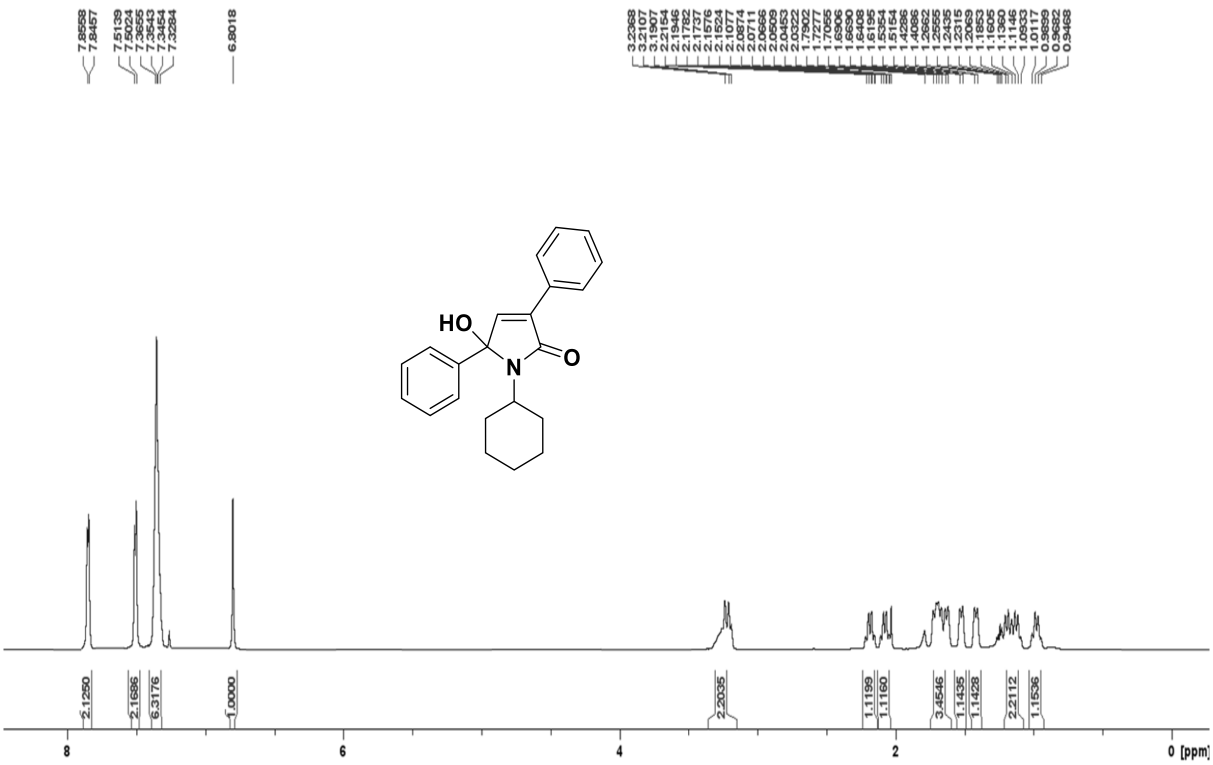

^{13}C-NMR ($CDCl_3$, 150 MHz) of compound 4.

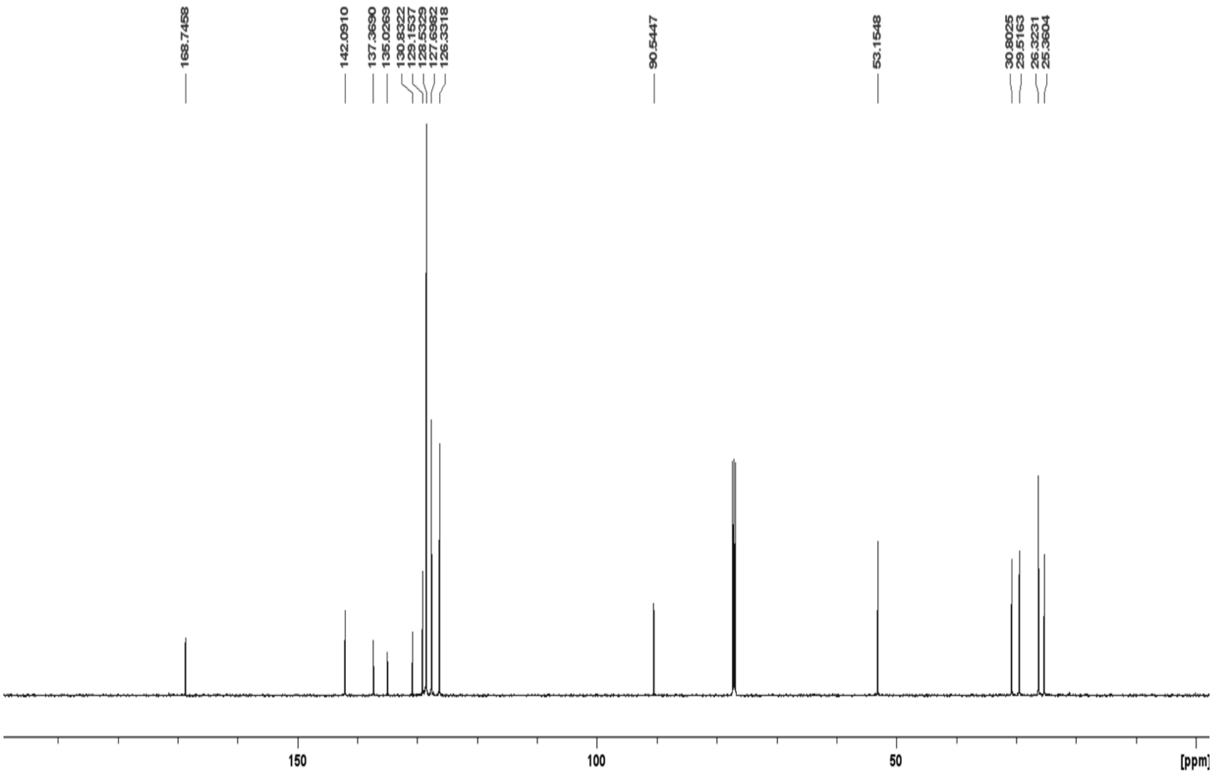

^{1}H-NMR ($CDCl_3$, 500 MHz) of compound 5.

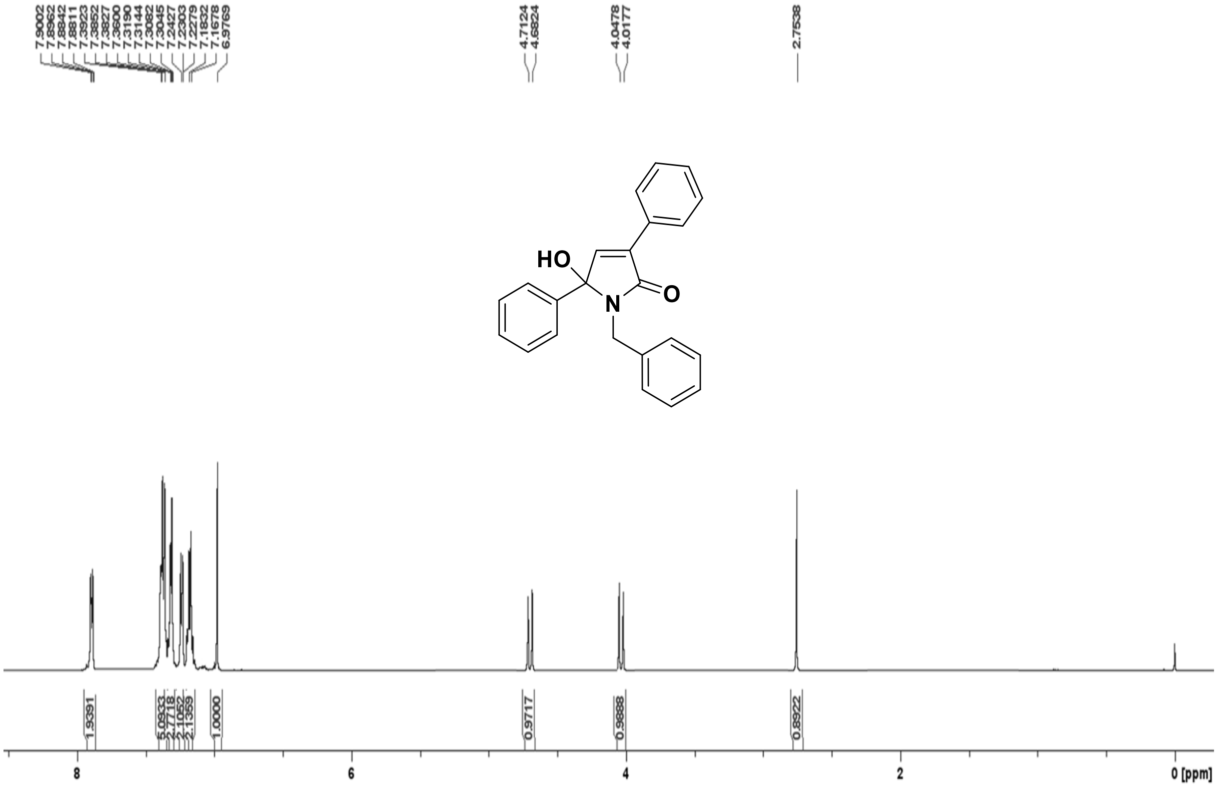

^{13}C-NMR ($CDCl_3$, 125 MHz) of compound 5.

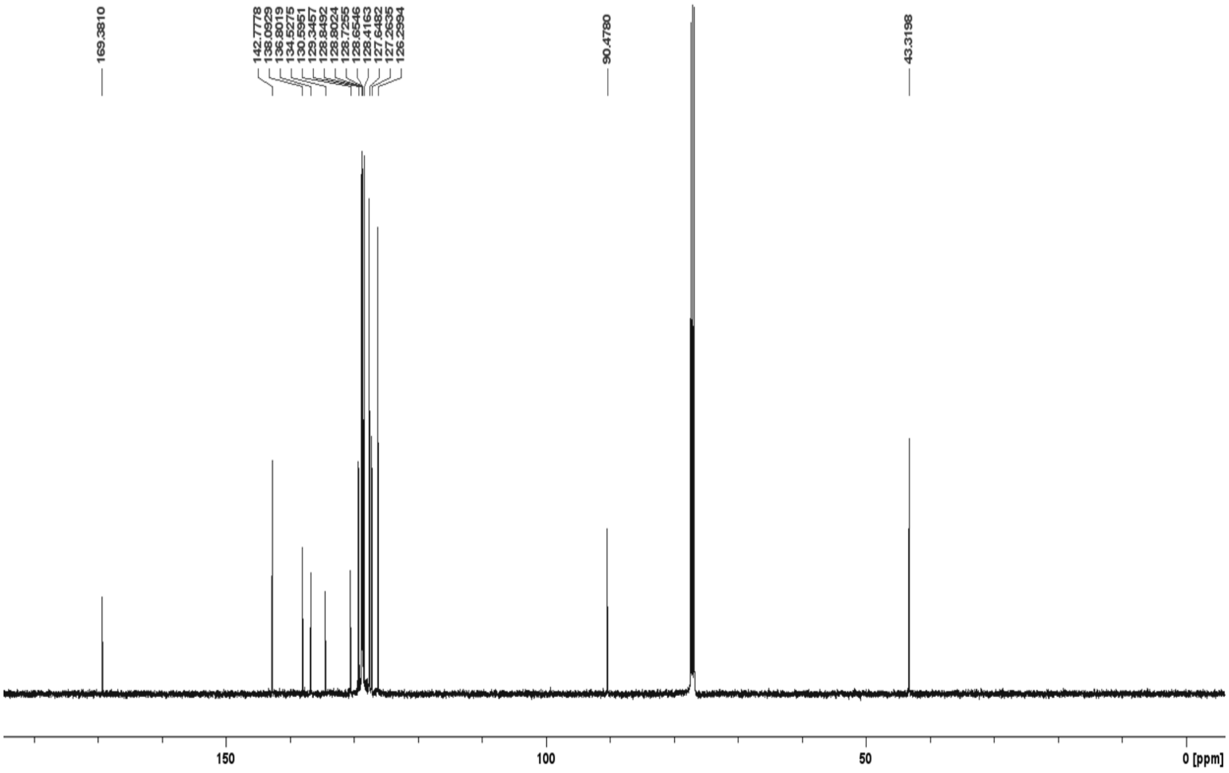

^{1}H-NMR ($CDCl_3$, 600 MHz) of compound 6.

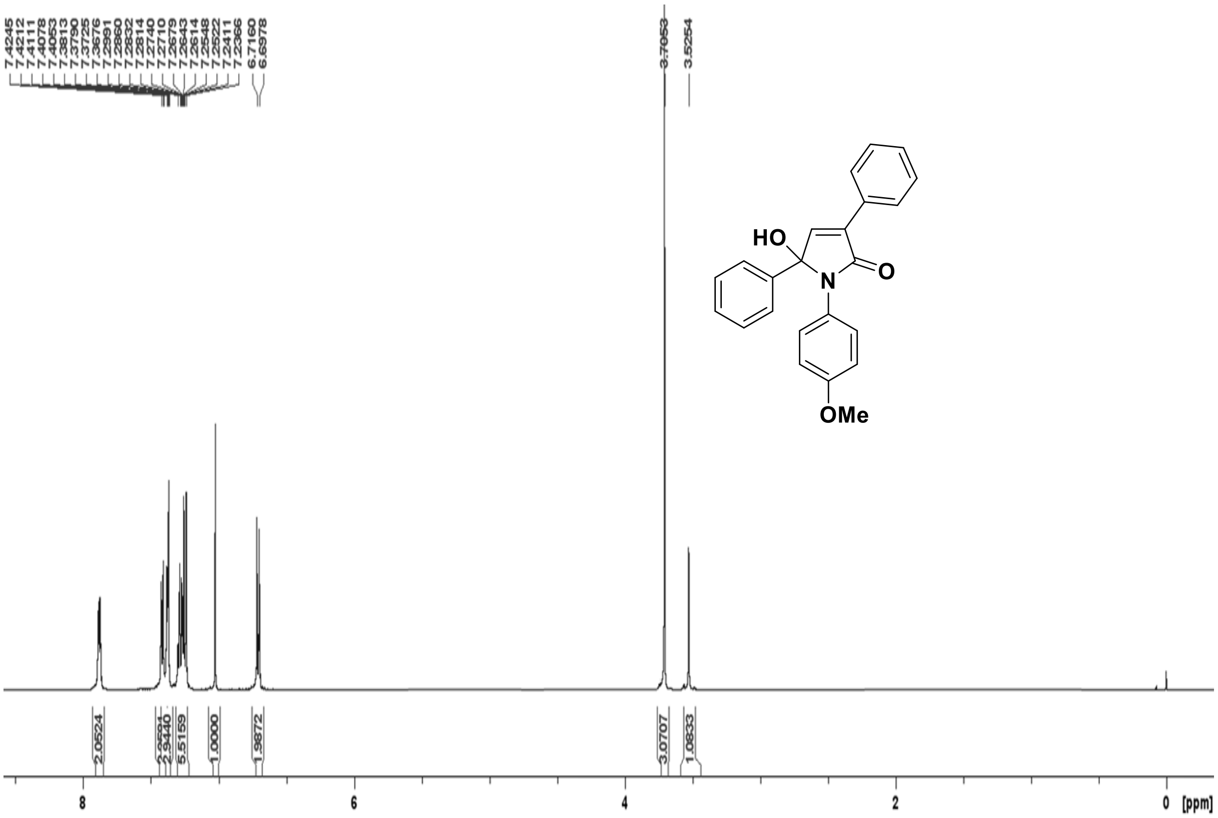

^{13}C-NMR ($CDCl_3$, 150 MHz) of compound 6.

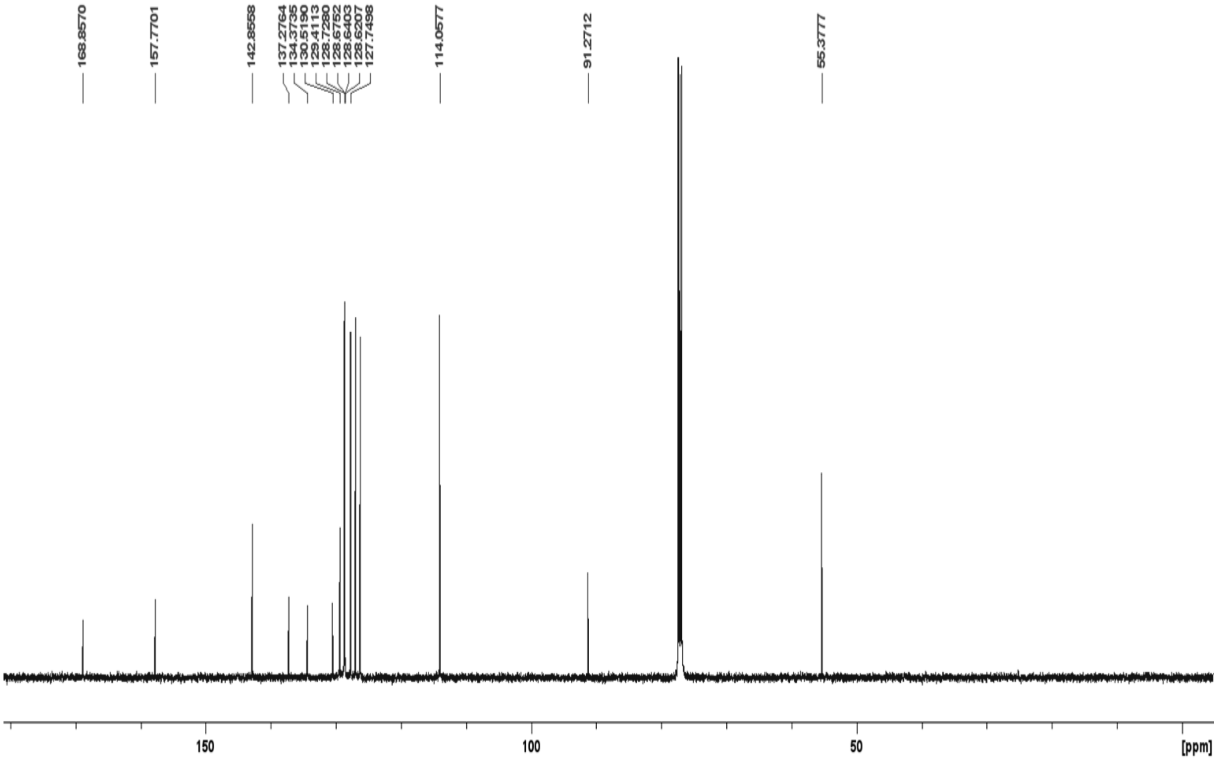

^{1}H-NMR ($CDCl_3$, 600 MHz) of compound 7.

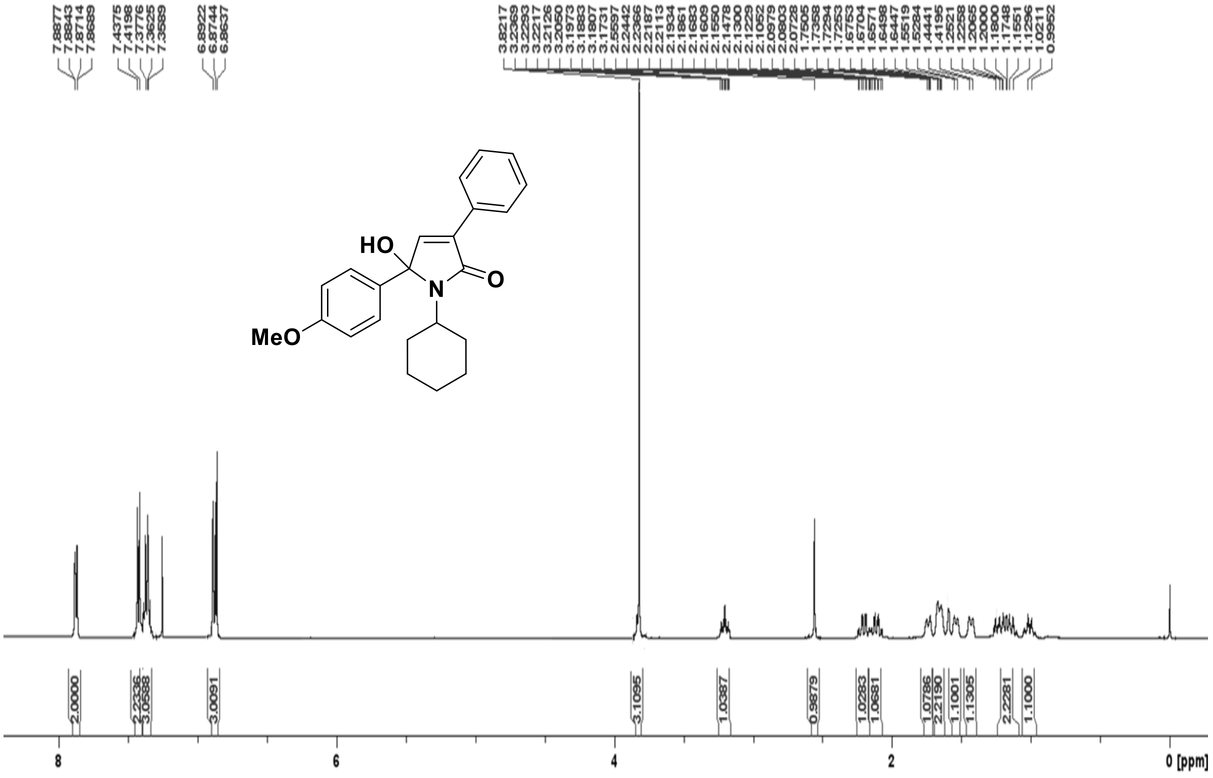

^{13}C-NMR ($CDCl_3$, 150 MHz) of compound 7.

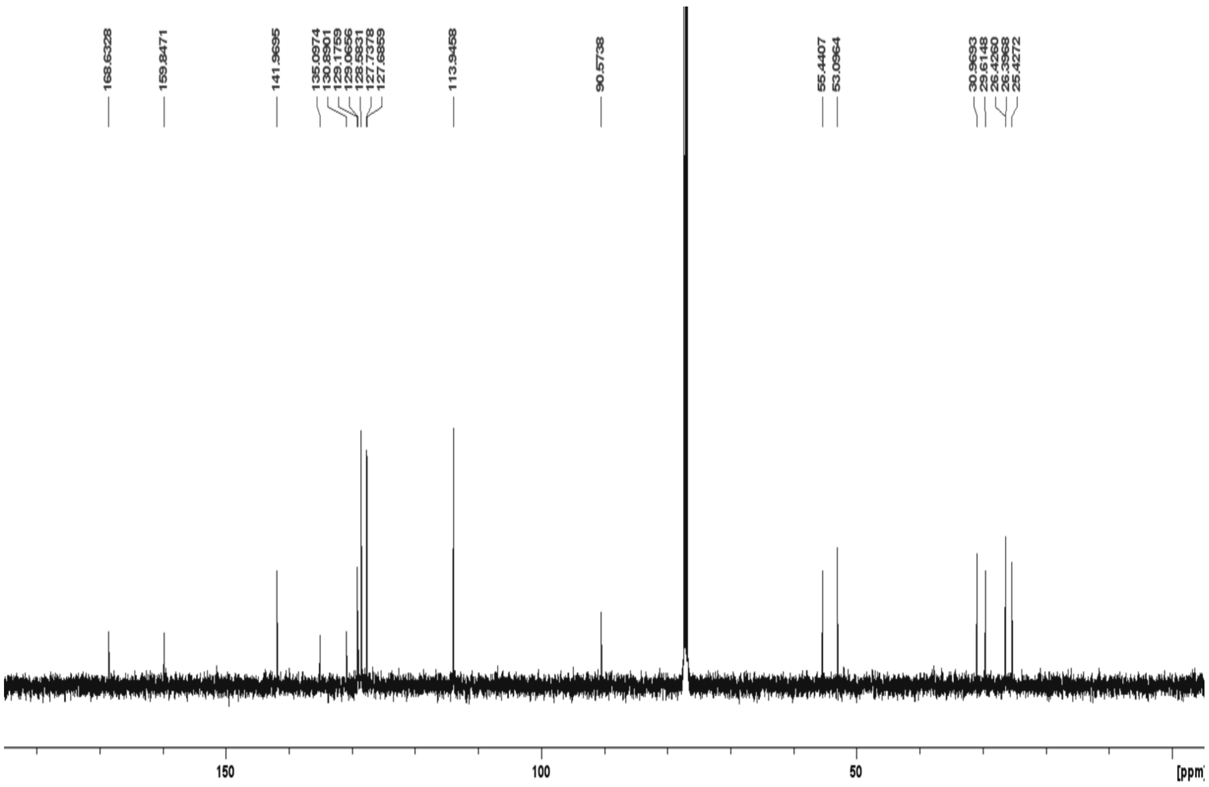

^{1}H-NMR ($CDCl_3$, 500 MHz) of compound 8.

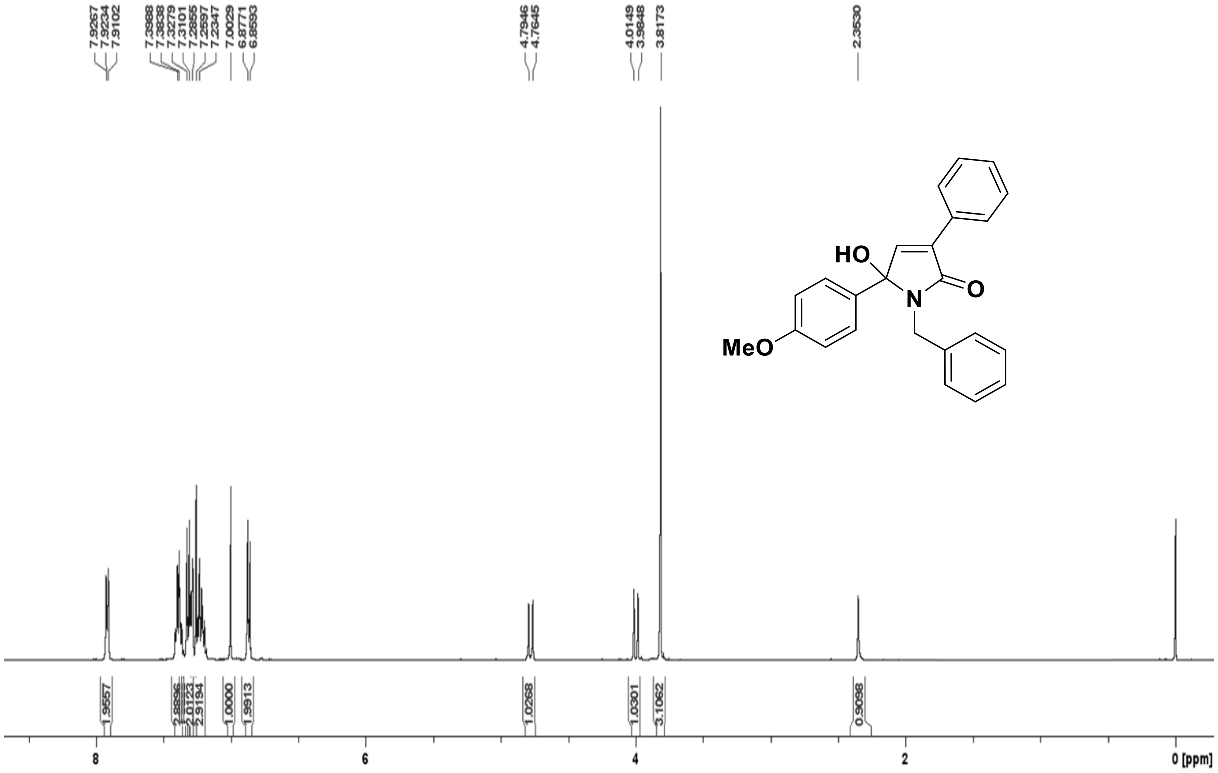

^{13}C-NMR ($CDCl_3$, 125 MHz) of compound 8.

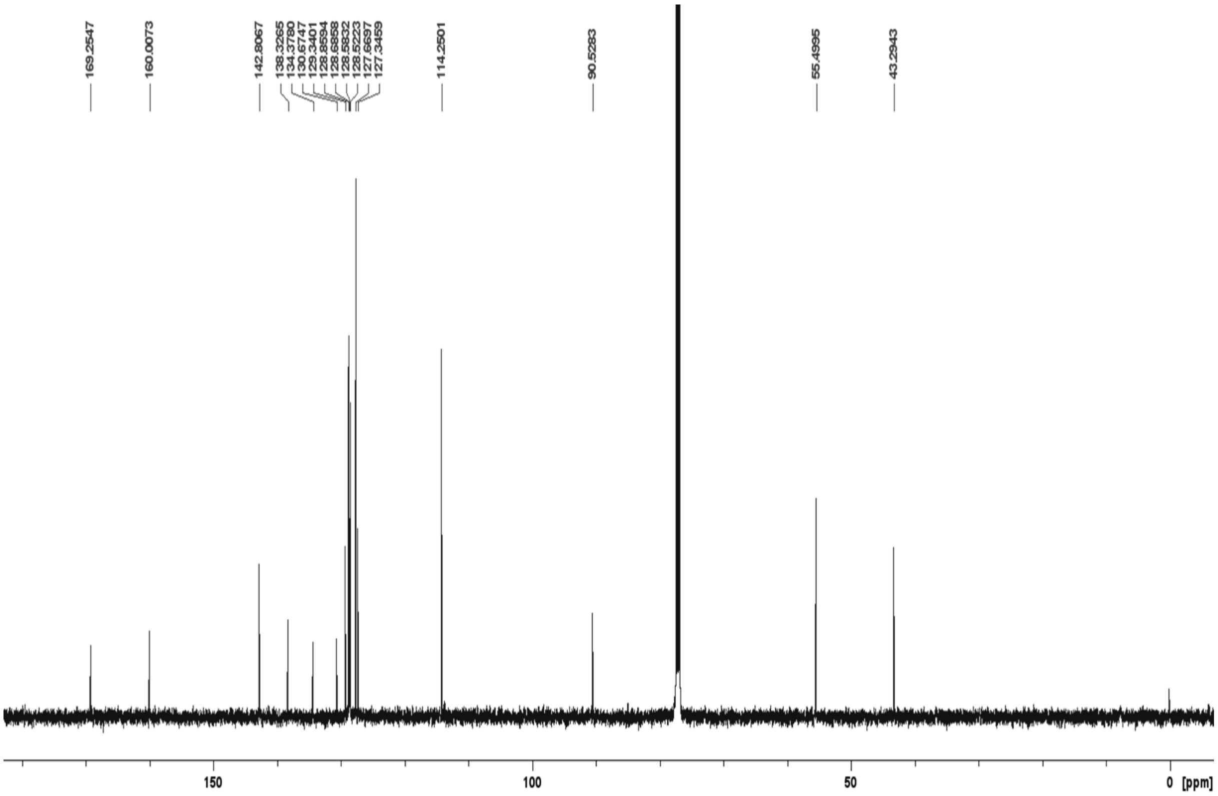

^{1}H-NMR ($CDCl_3$, 500 MHz) of compound 9.

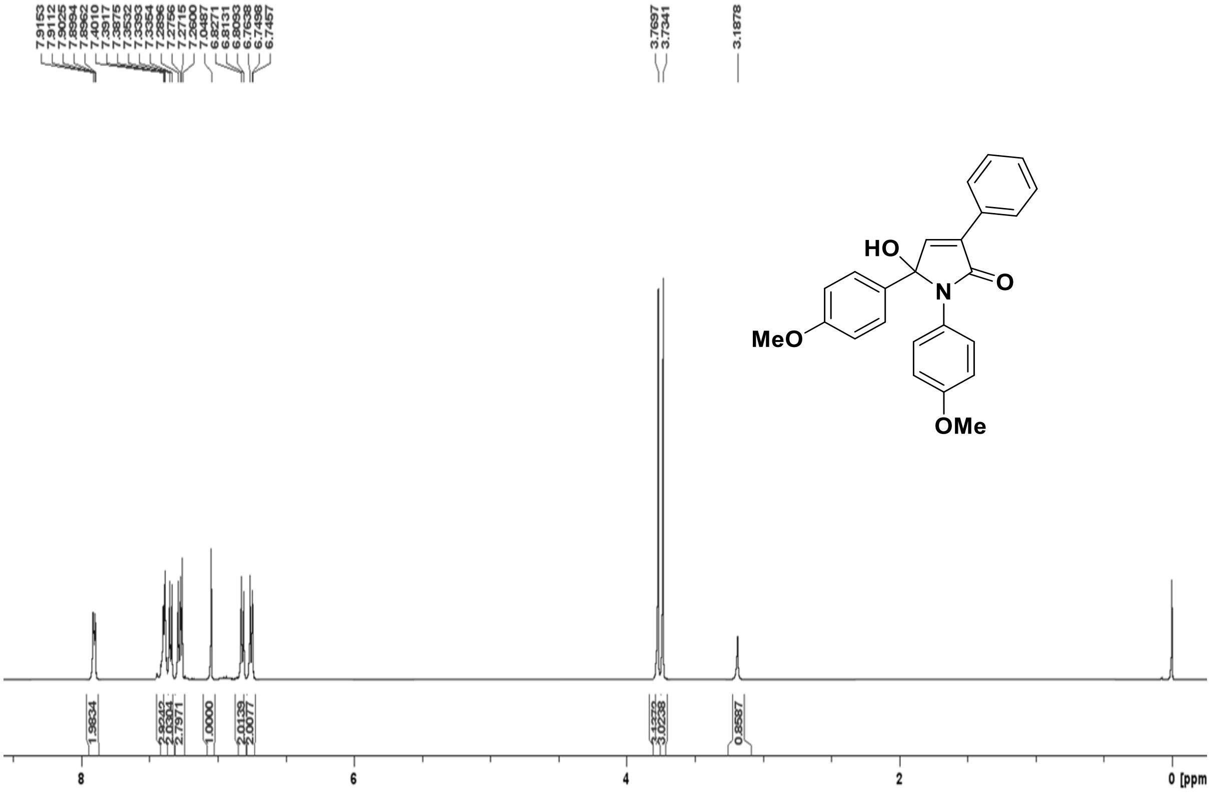

^{13}C-NMR ($CDCl_3$, 125 MHz) of compound 9.

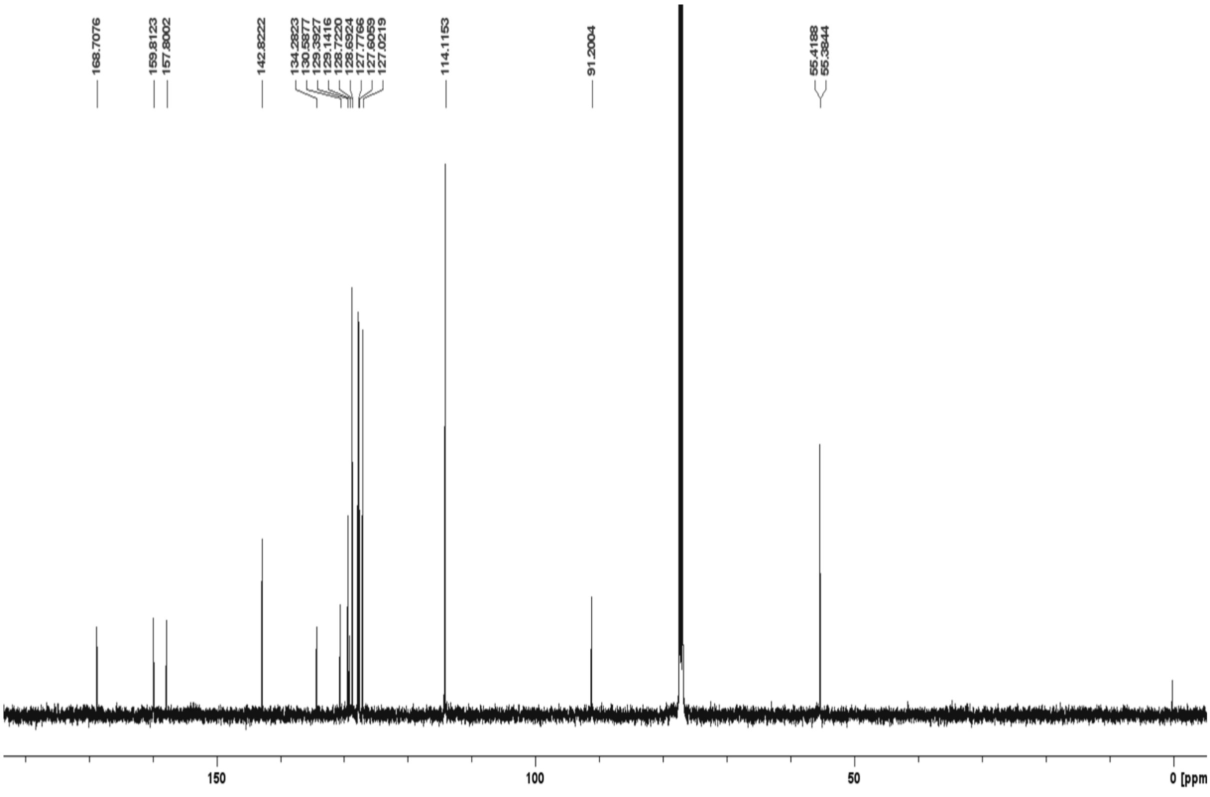

^{1}H-NMR ($CDCl_3$, 600 MHz) of compound 10.

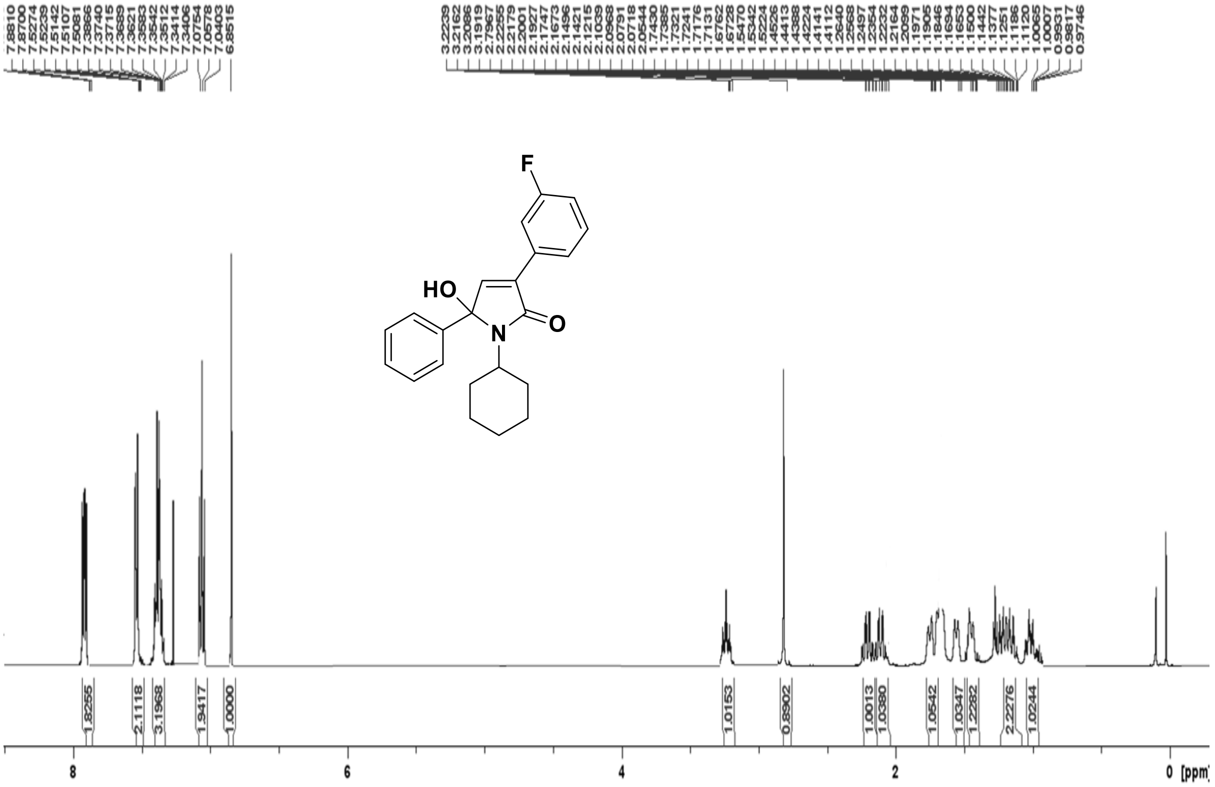

^{13}C-NMR ($CDCl_3$, 150 MHz) of compound 10.

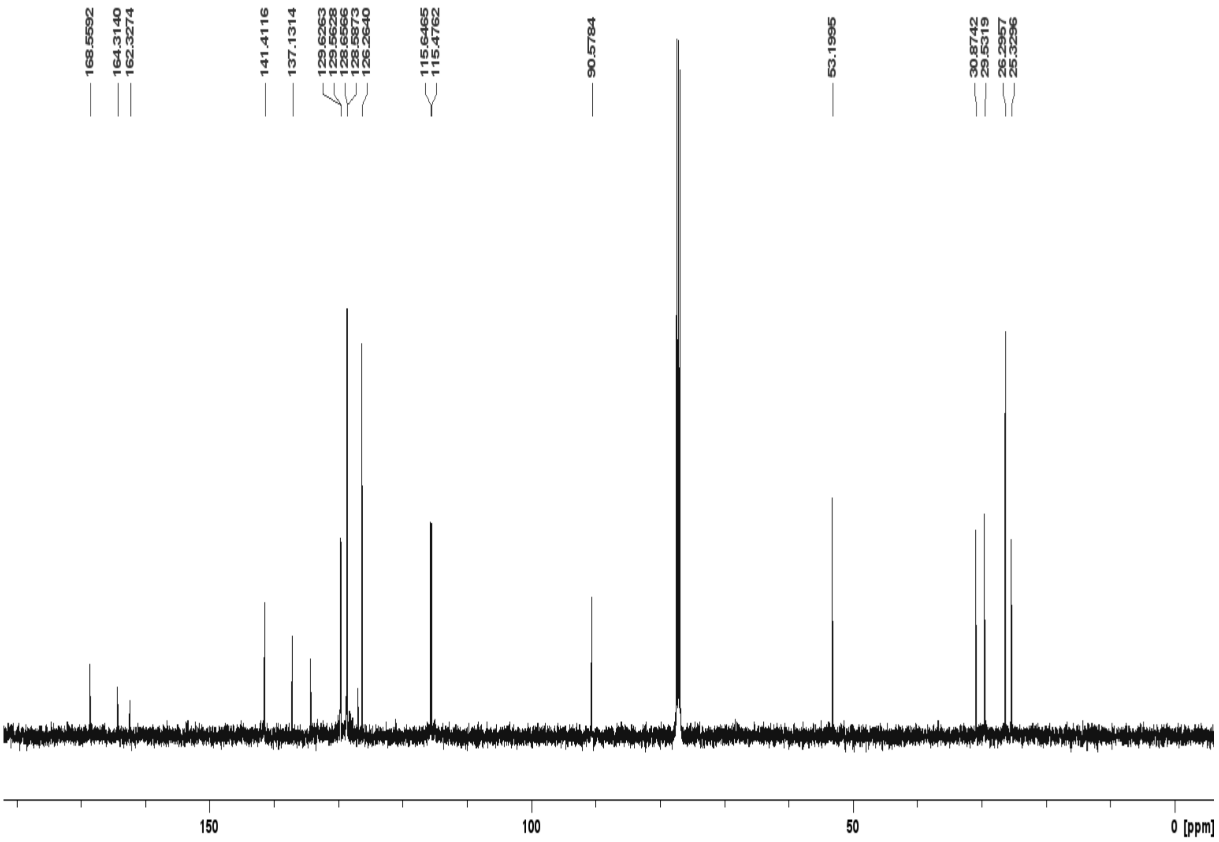

^{1}H-NMR ($CDCl_3$, 500 MHz) of compound 11.

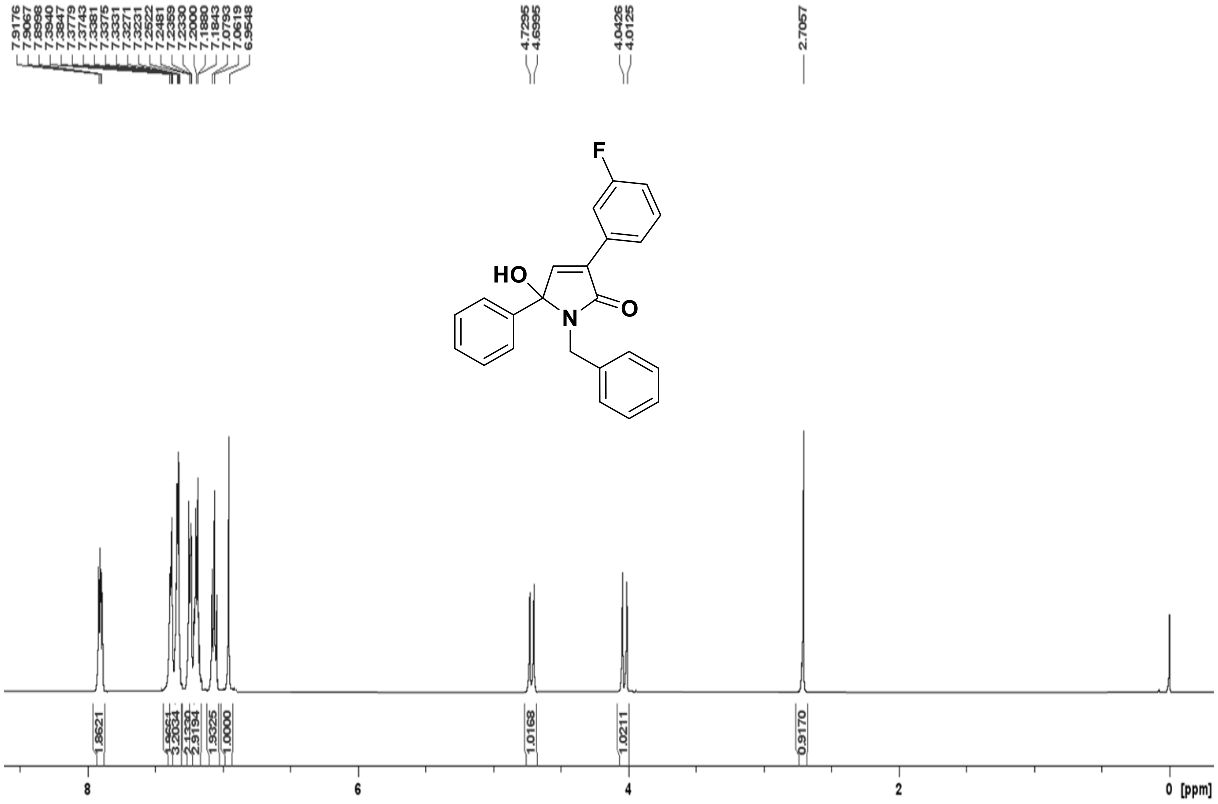

^{13}C-NMR ($CDCl_3$, 125 MHz) of compound 11.

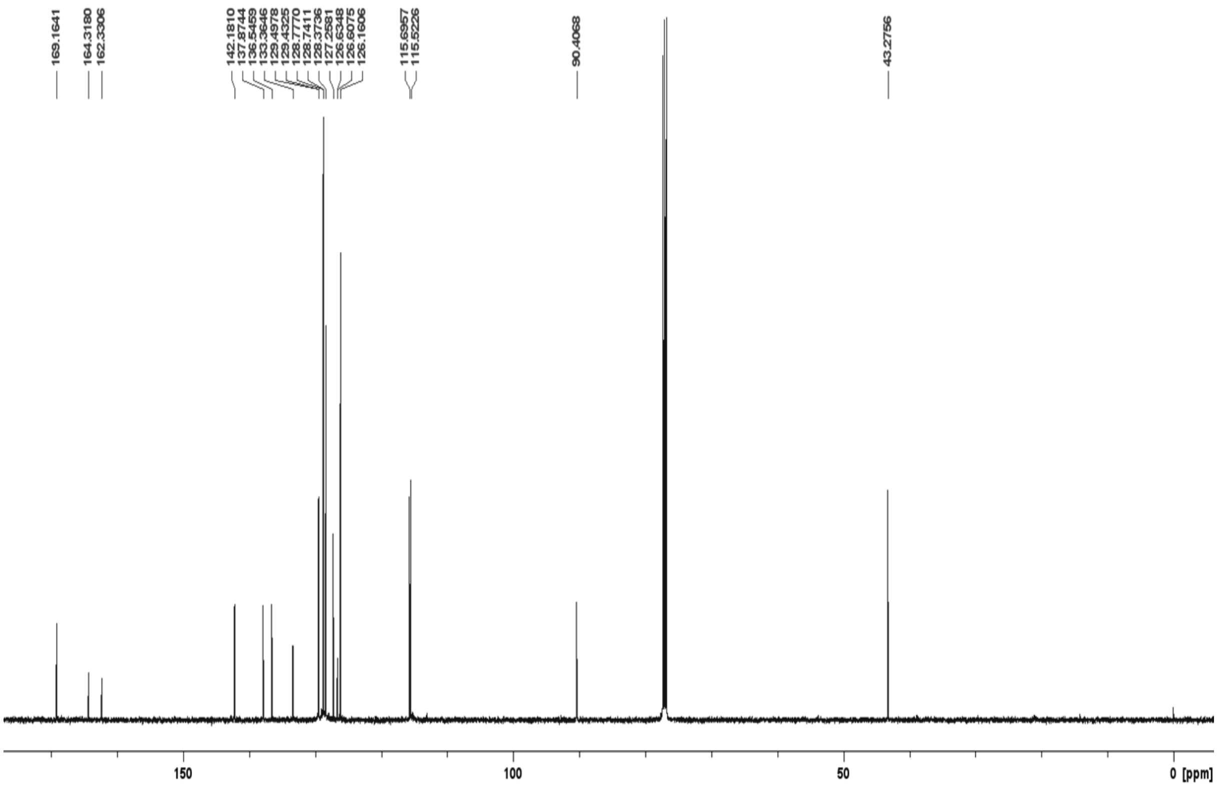

^{1}H-NMR ($CDCl_3$, 600 MHz) of compound 12.

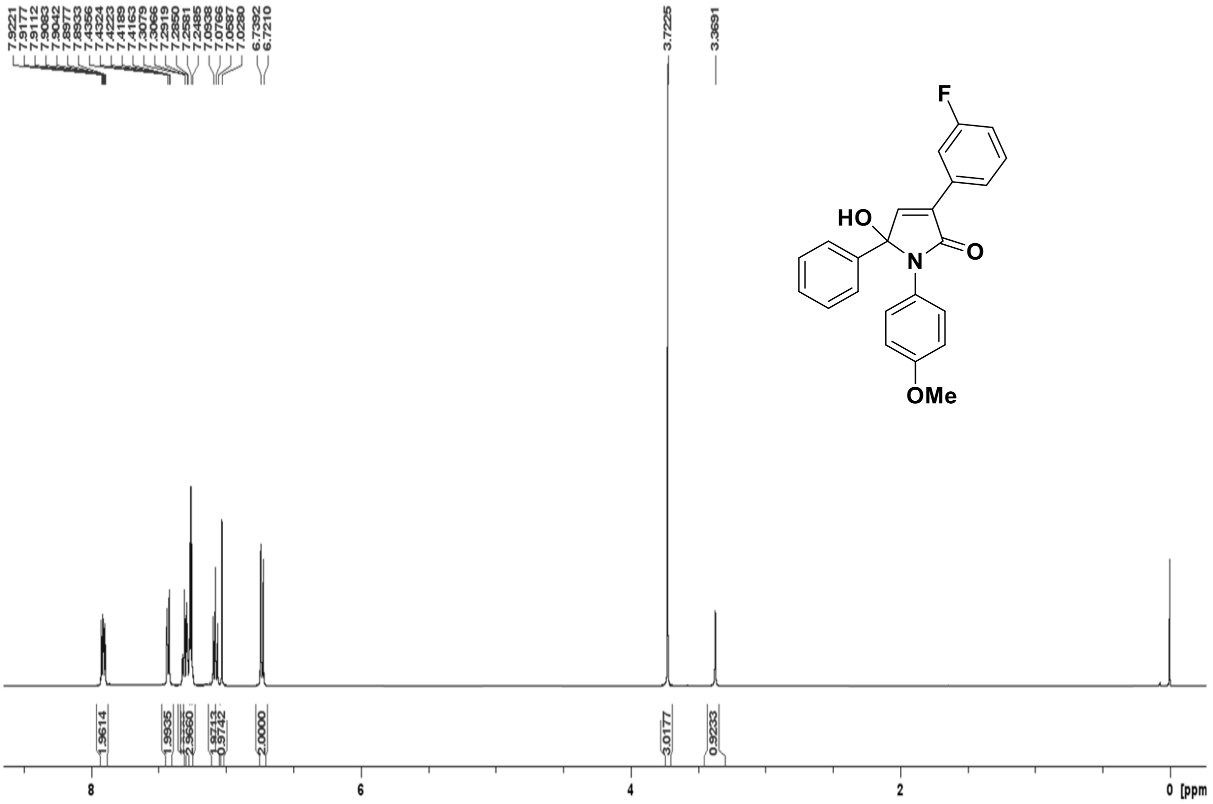

^{13}C-NMR ($CDCl_3$, 150 MHz) of compound 12.

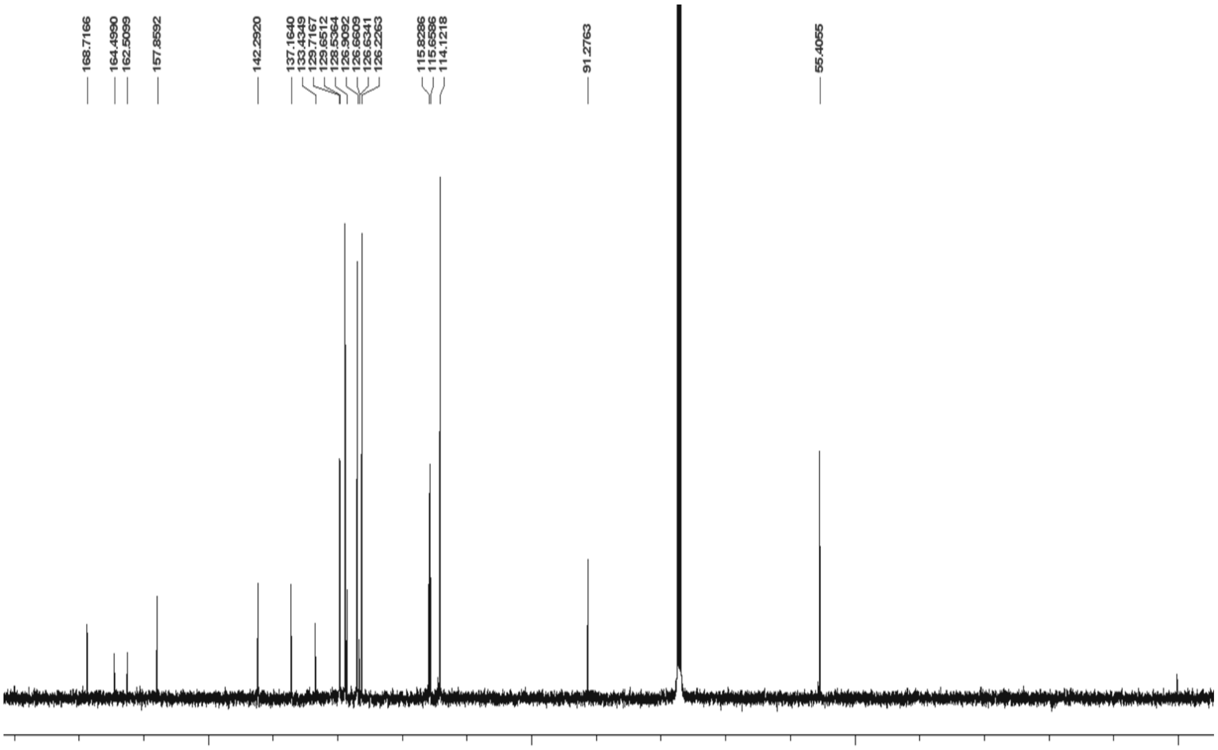

^{1}H-NMR ($CDCl_3$, 600 MHz) of compound 13.

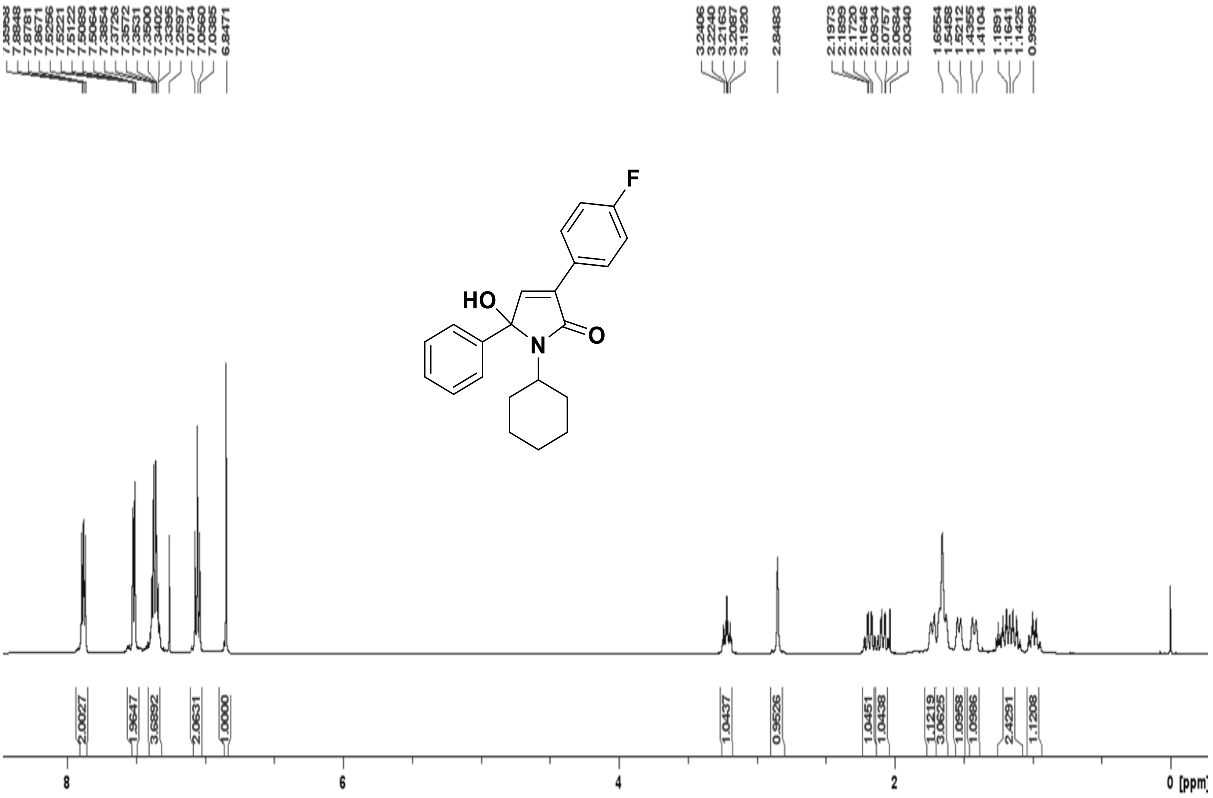

^{13}C-NMR ($CDCl_3$, 150 MHz) of compound 13.

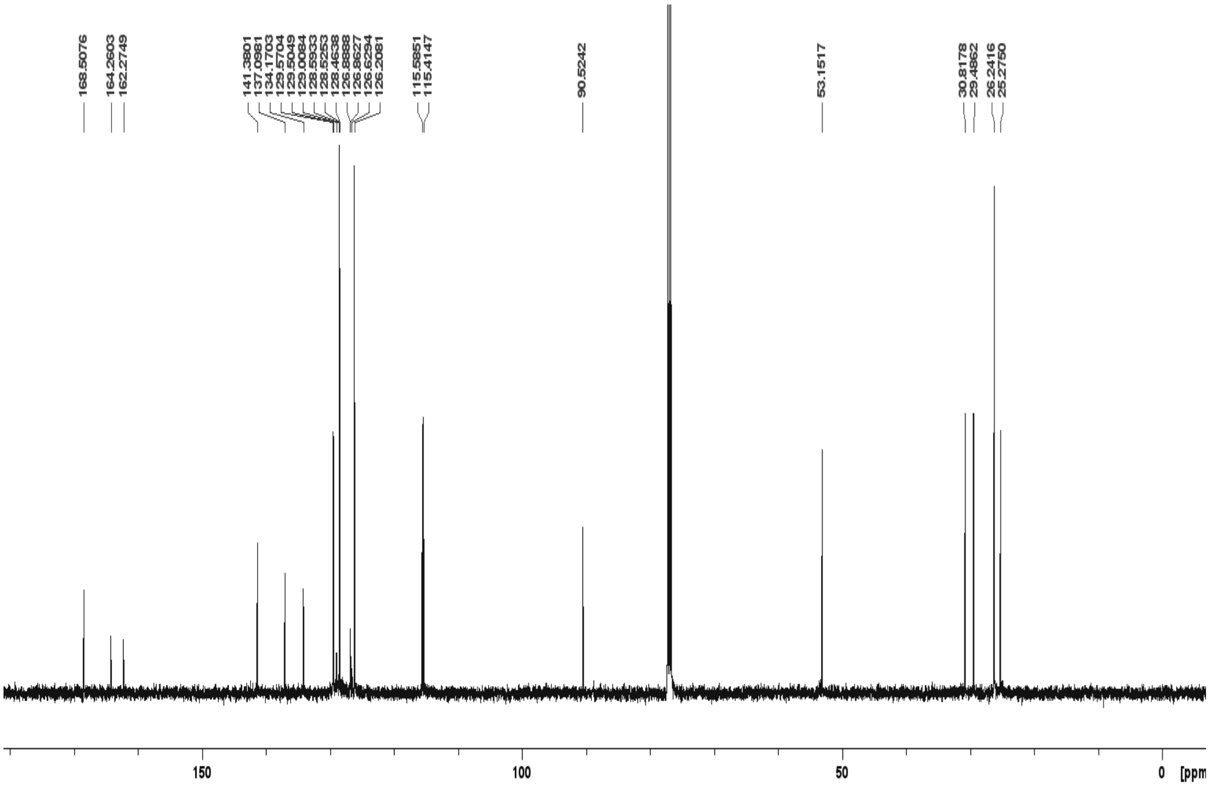

^{1}H-NMR ($CDCl_3$, 500 MHz) of compound 14.

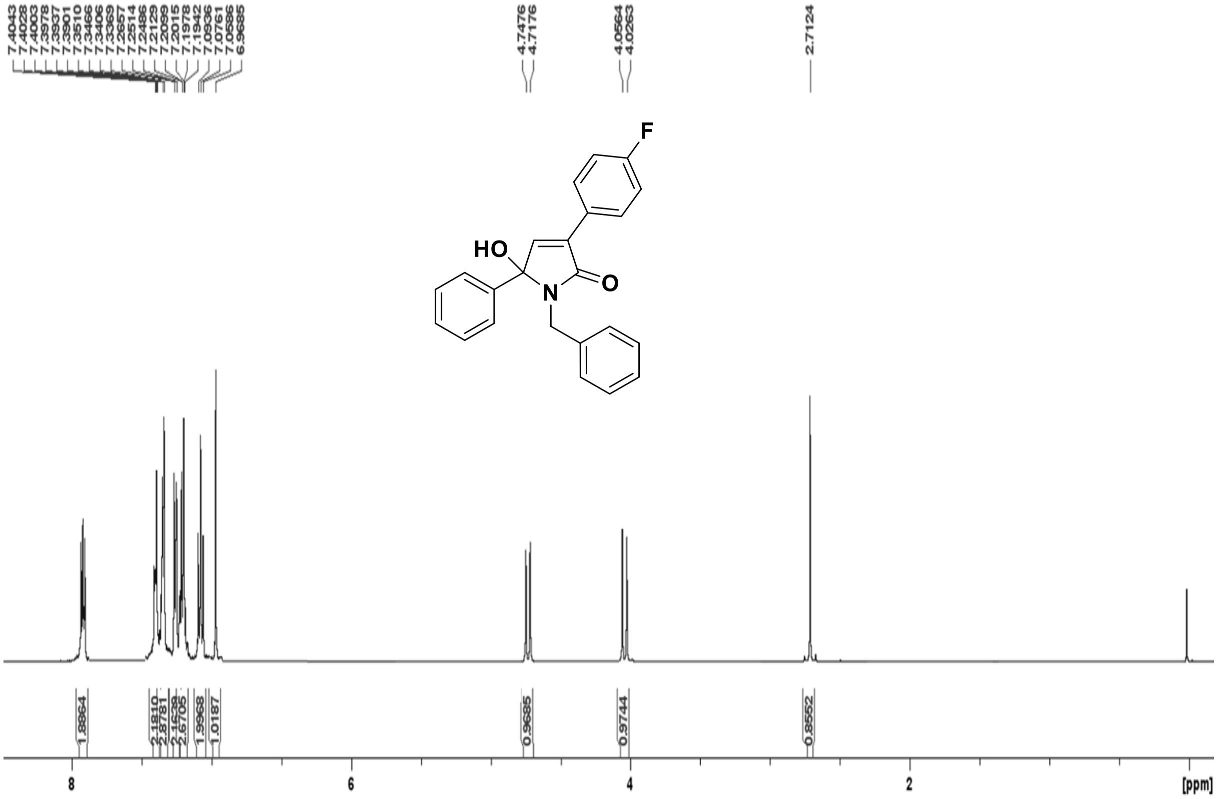

^{13}C-NMR ($CDCl_3$, 125 MHz) of compound 14.

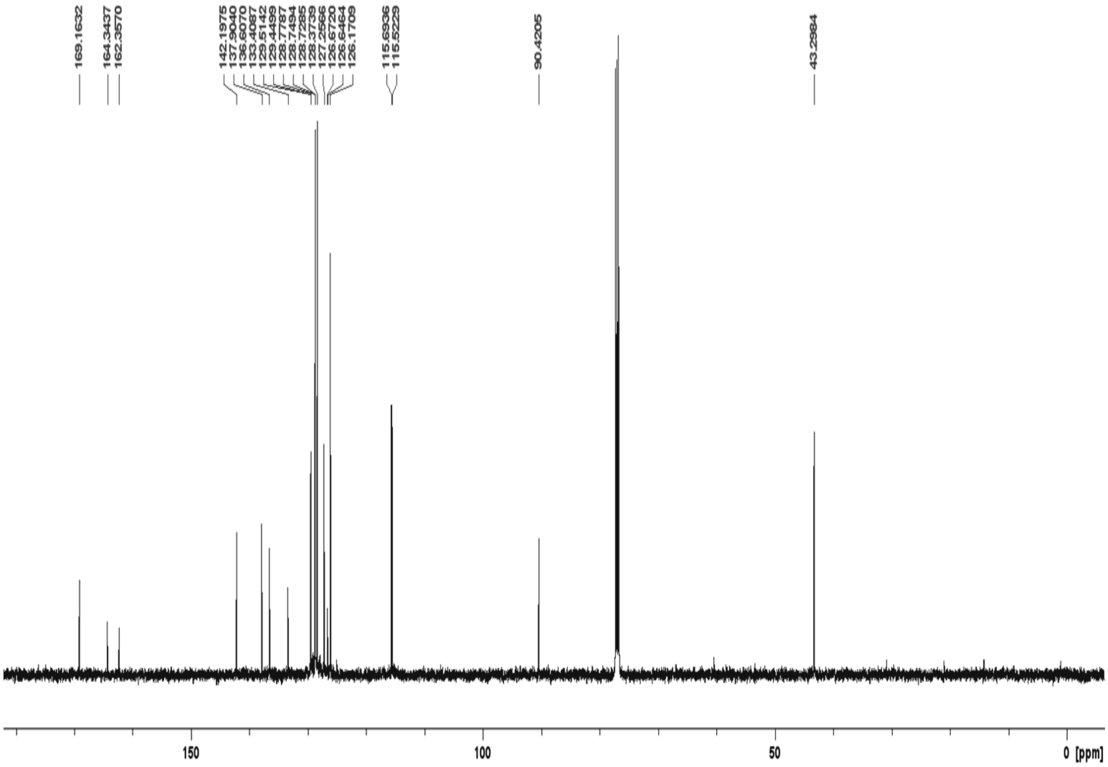

^{1}H-NMR ($CDCl_3$, 600 MHz) of compound 15.

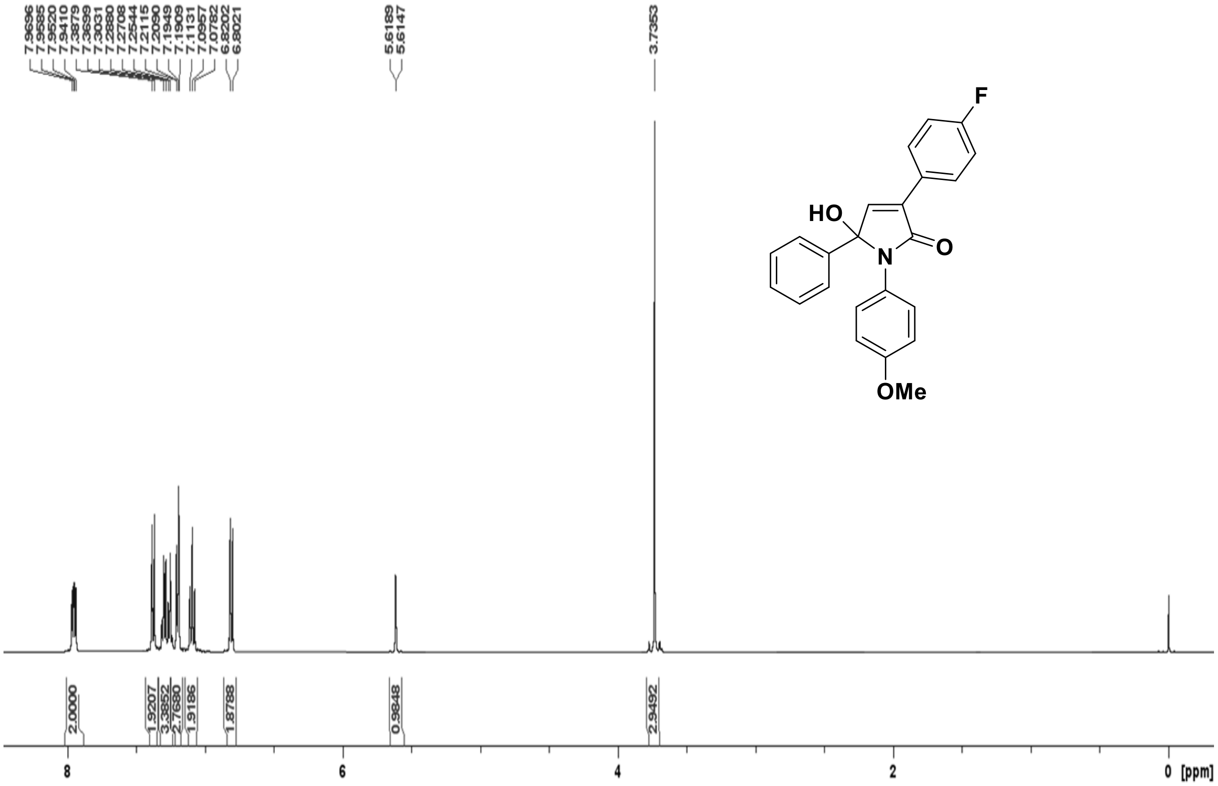

^{13}C-NMR ($CDCl_3$, 150 MHz) of compound 15.

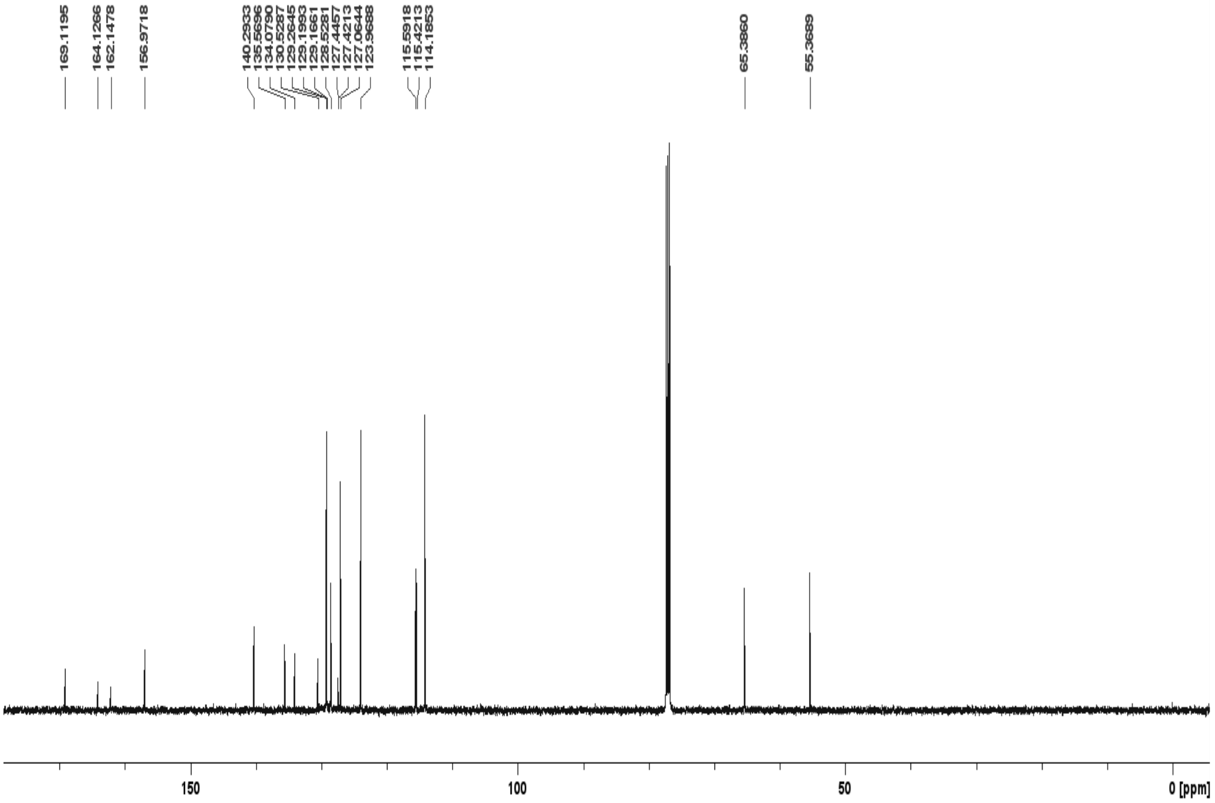

^{1}H-NMR ($CDCl_3$, 600 MHz) of compound 16.

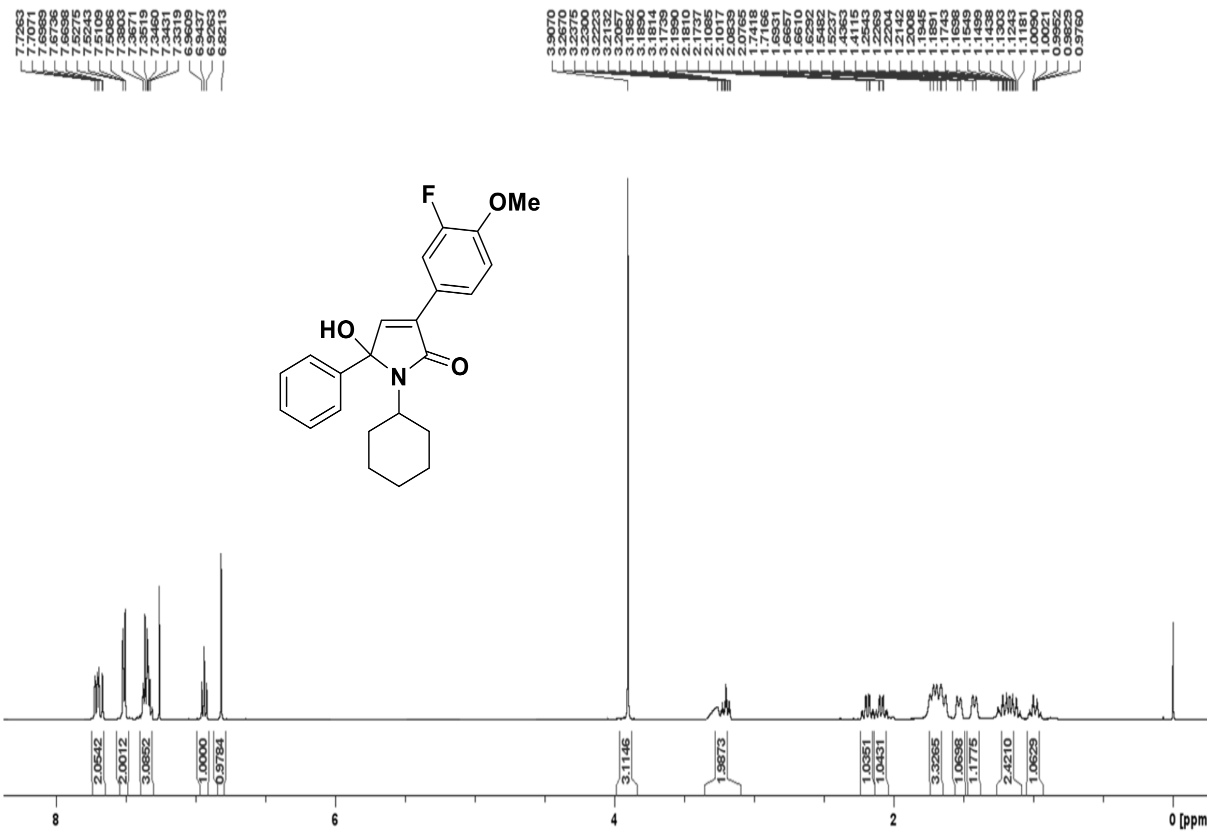

^{13}C-NMR ($CDCl_3$, 150 MHz) of compound 16.

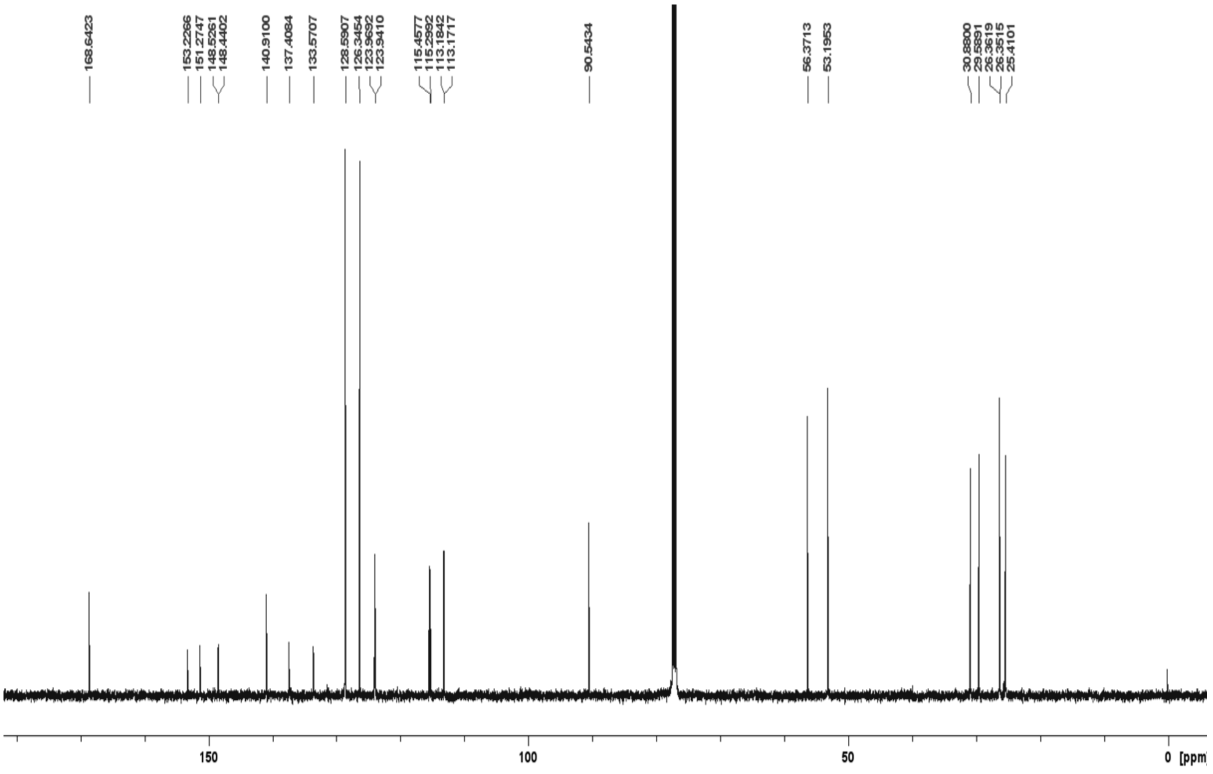

^{1}H-NMR ($CDCl_3$, 500 MHz) of compound 17.

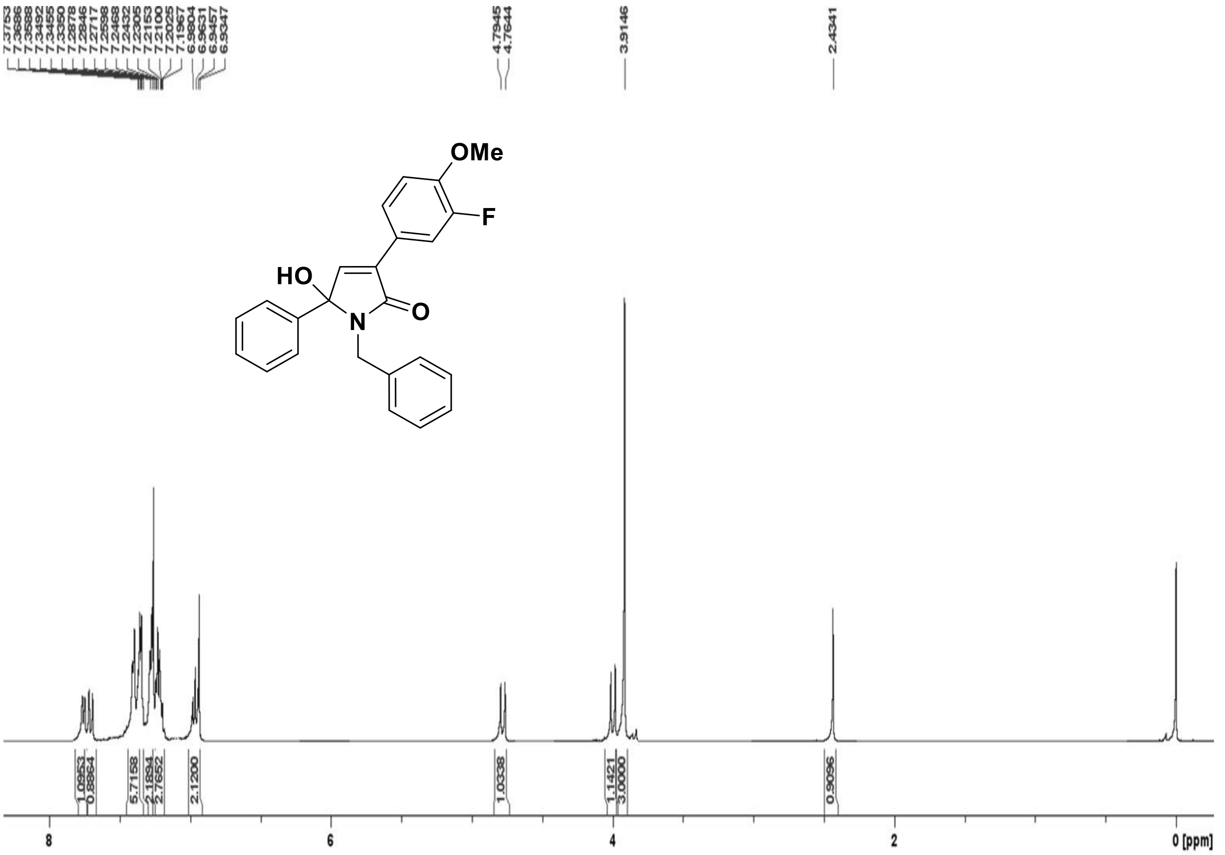

^{13}C-NMR ($CDCl_3$, 150 MHz) of compound 17.

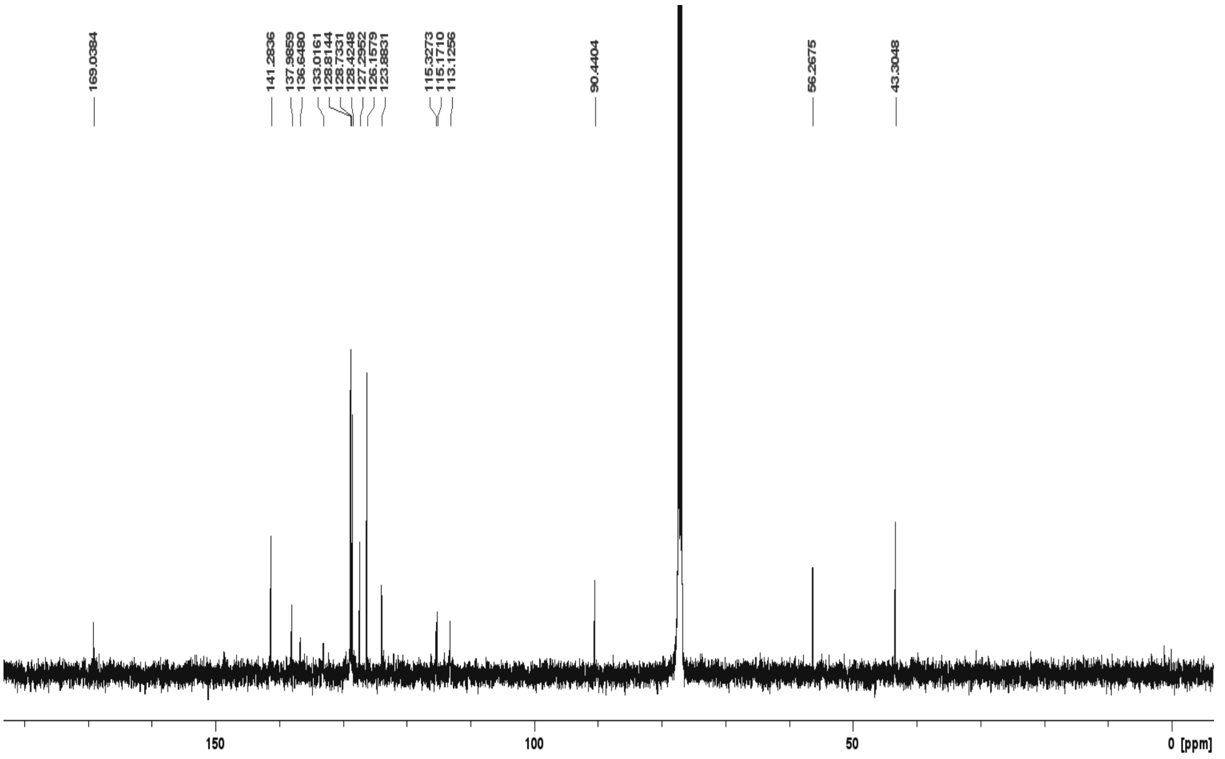

^{1}H-NMR (CDCl$_3$, 500 MHz) of compound 18.

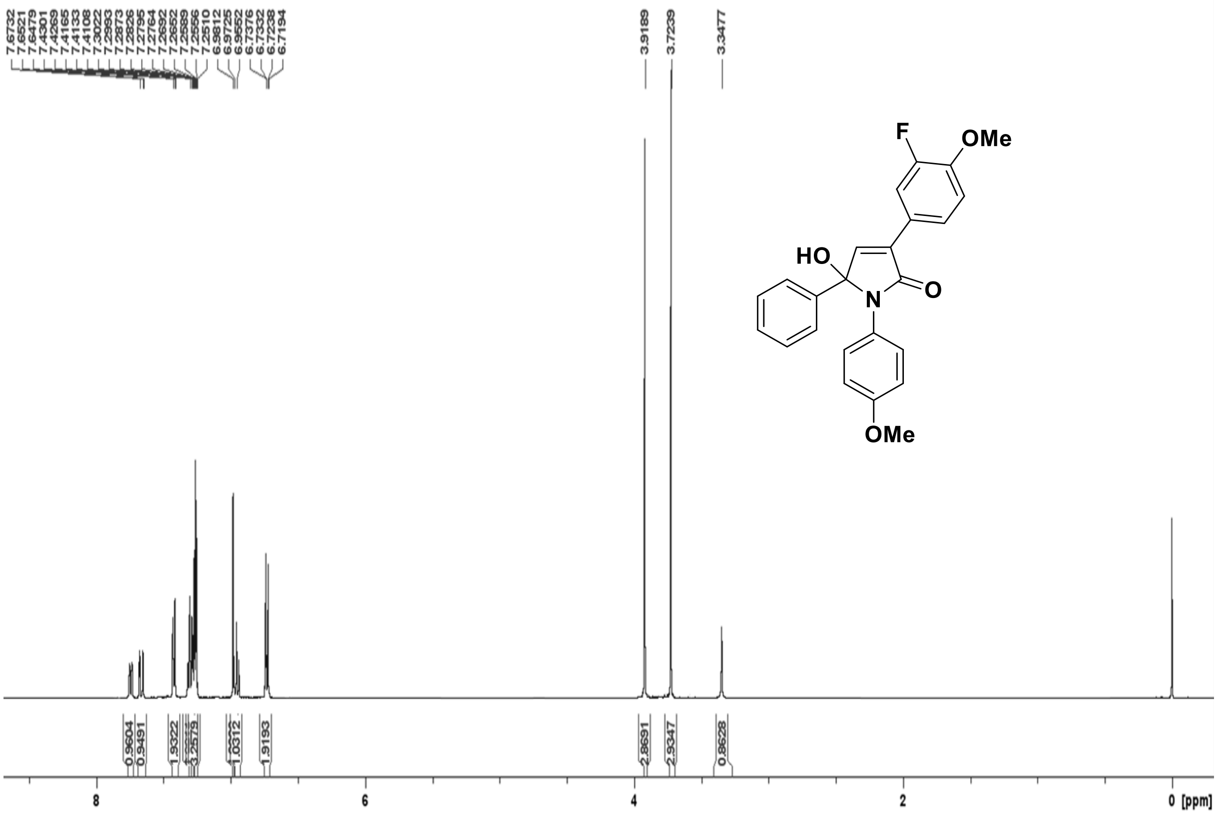

^{13}C-NMR (CDCl$_3$, 125 MHz) of compound 18.

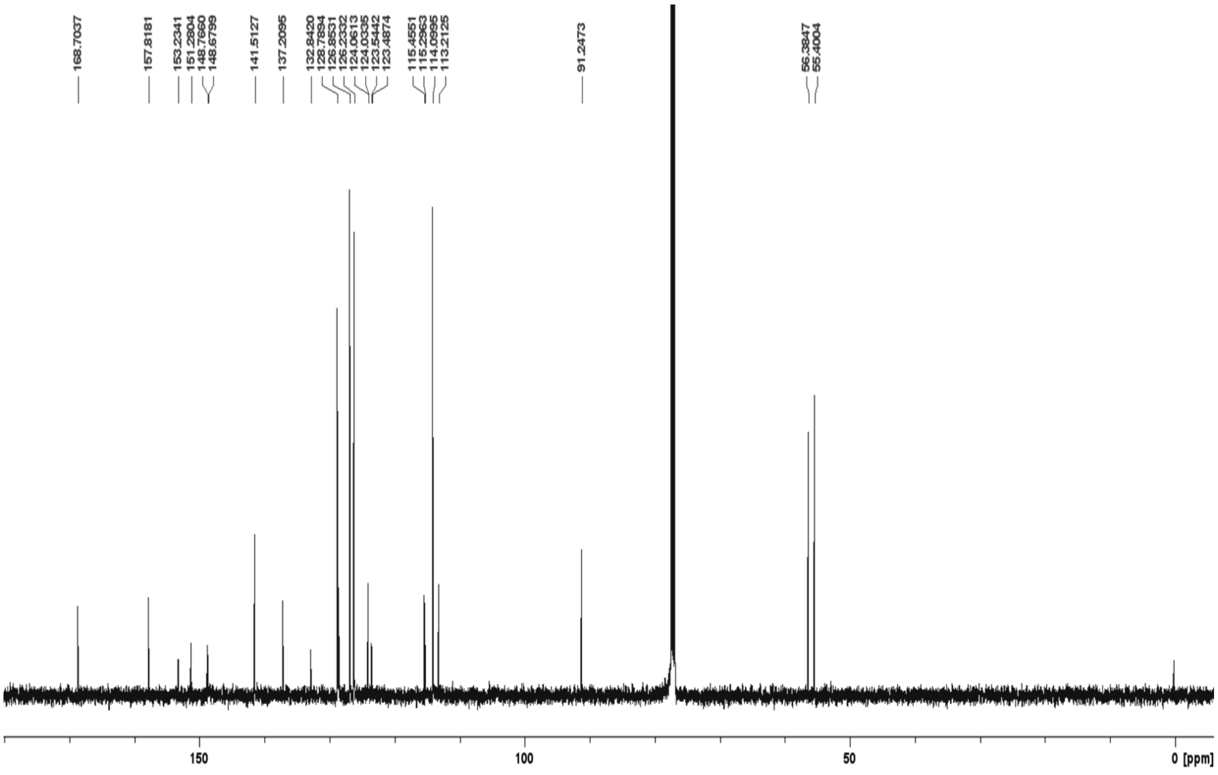

^{1}H-NMR ($CDCl_3$, 600 MHz) of compound 19.

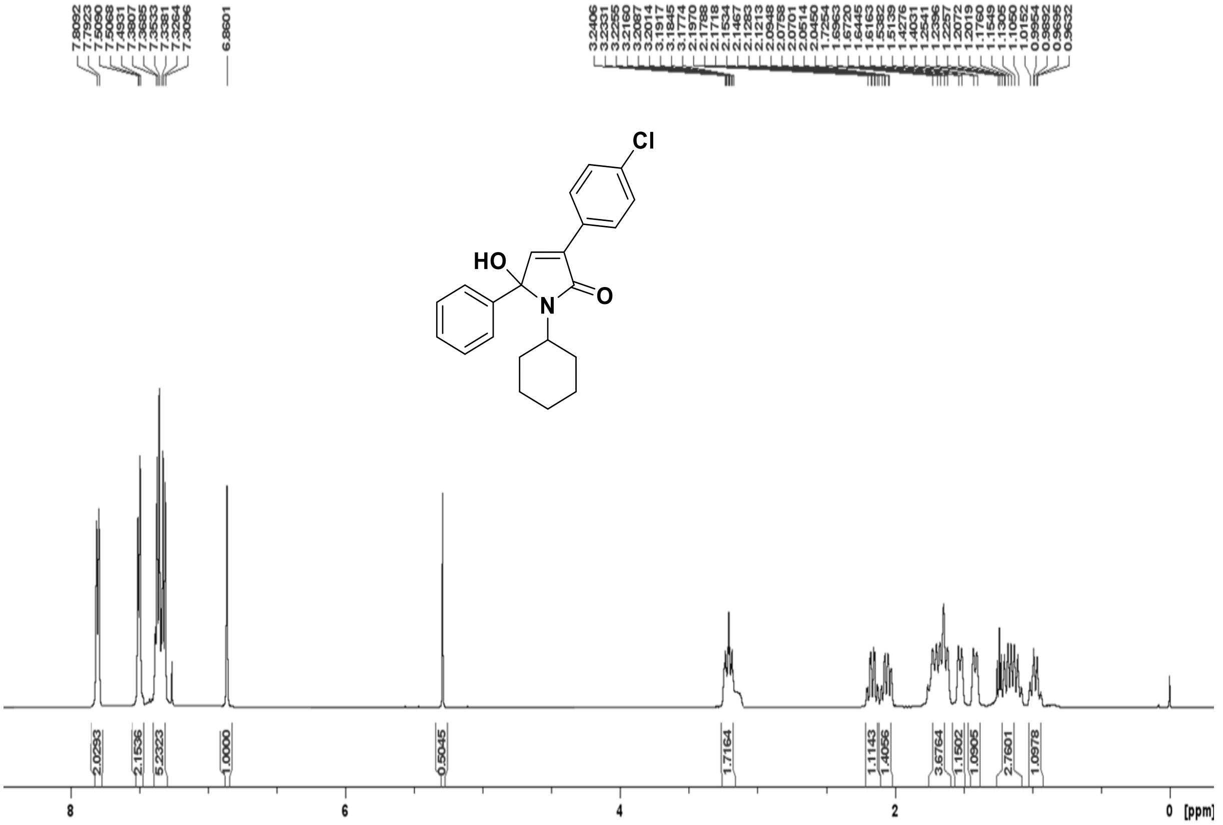

^{13}C-NMR ($CDCl_3$, 150 MHz) of compound 19.

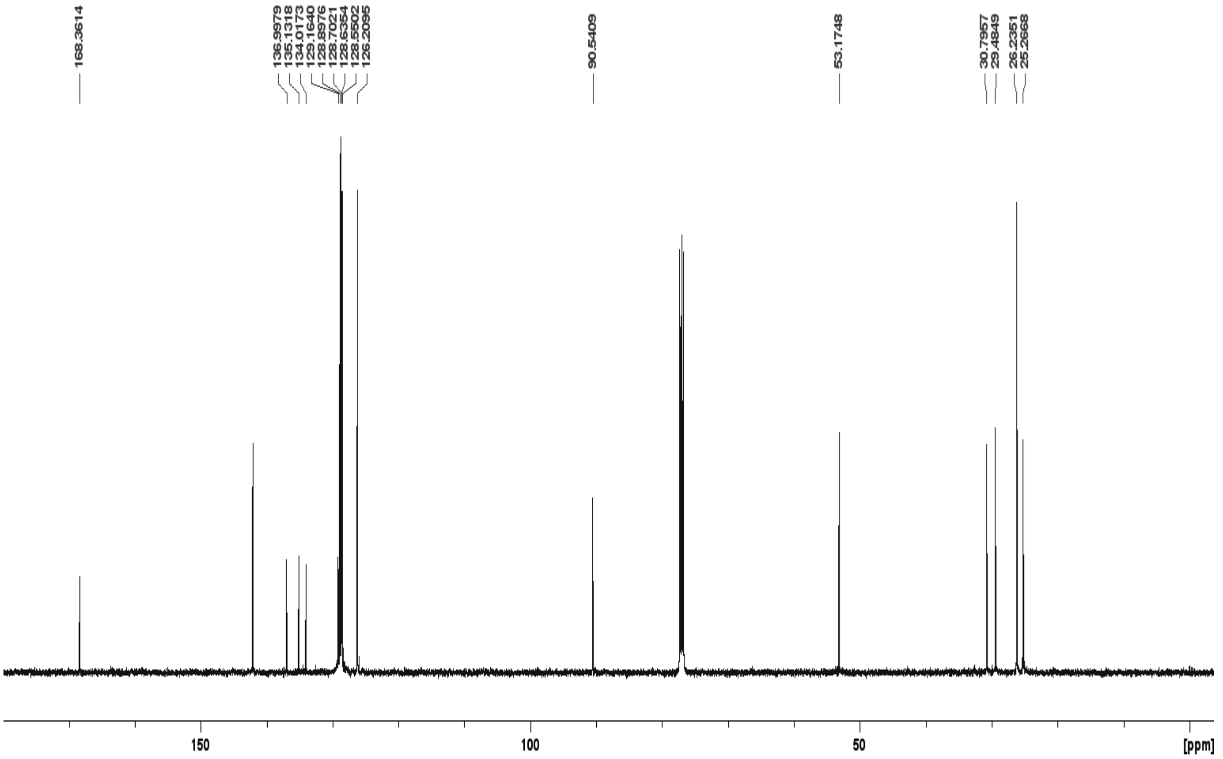

^{1}H-NMR ($CDCl_3$, 500 MHz) of compound 20.

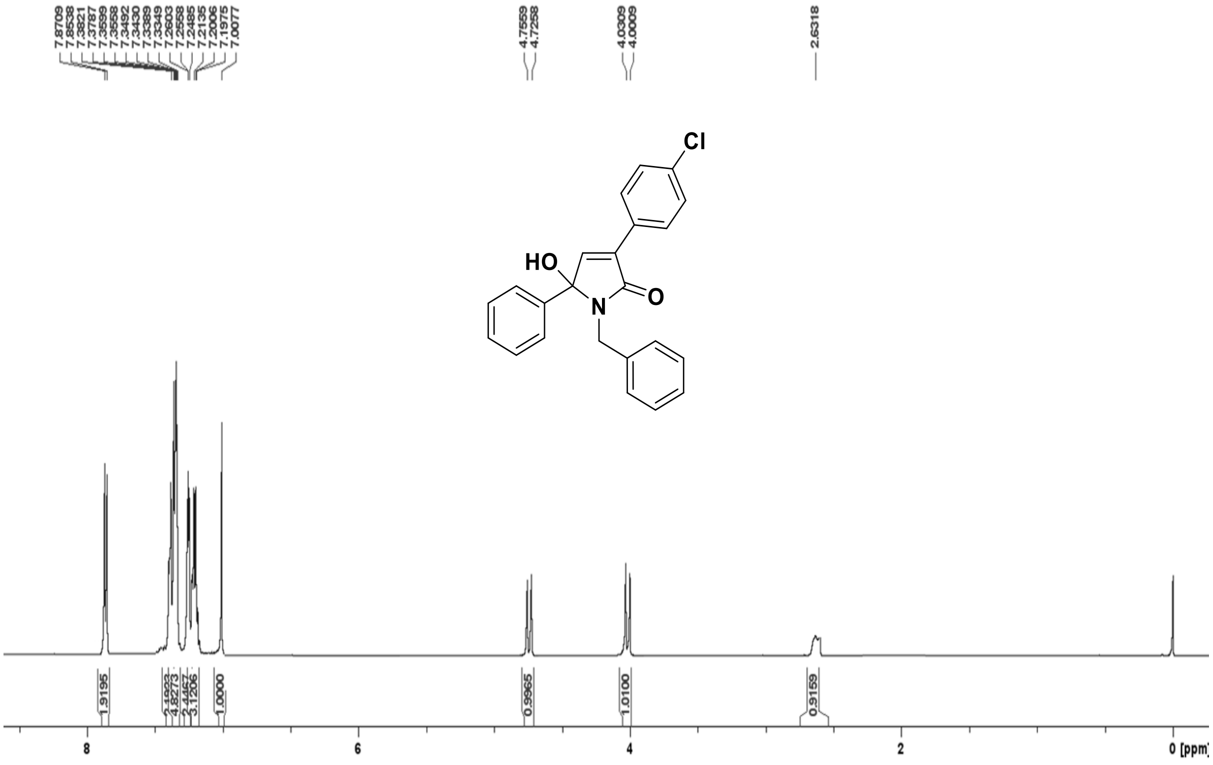

^{13}C-NMR ($CDCl_3$, 125 MHz) of compound 20.

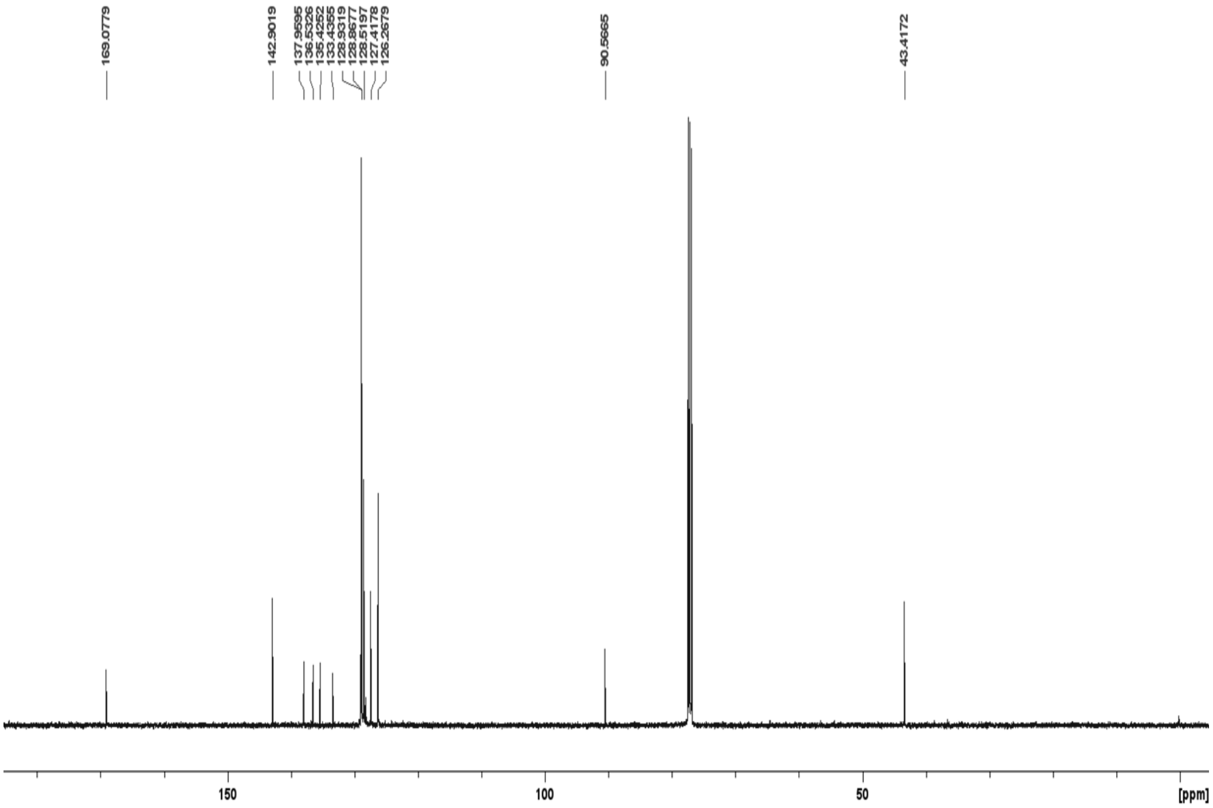

^{1}H-NMR ($CDCl_3$, 500 MHz) of compound 21.

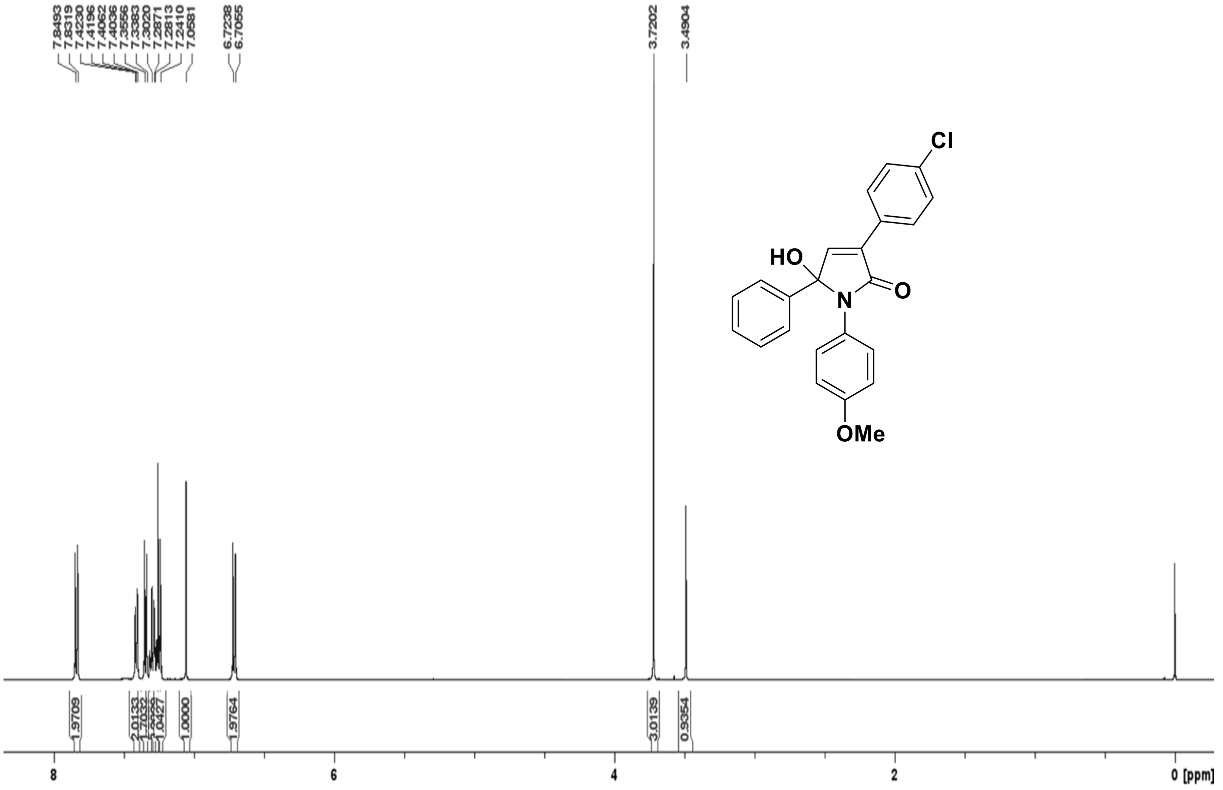

^{13}C-NMR ($CDCl_3$, 125 MHz) of compound 21.

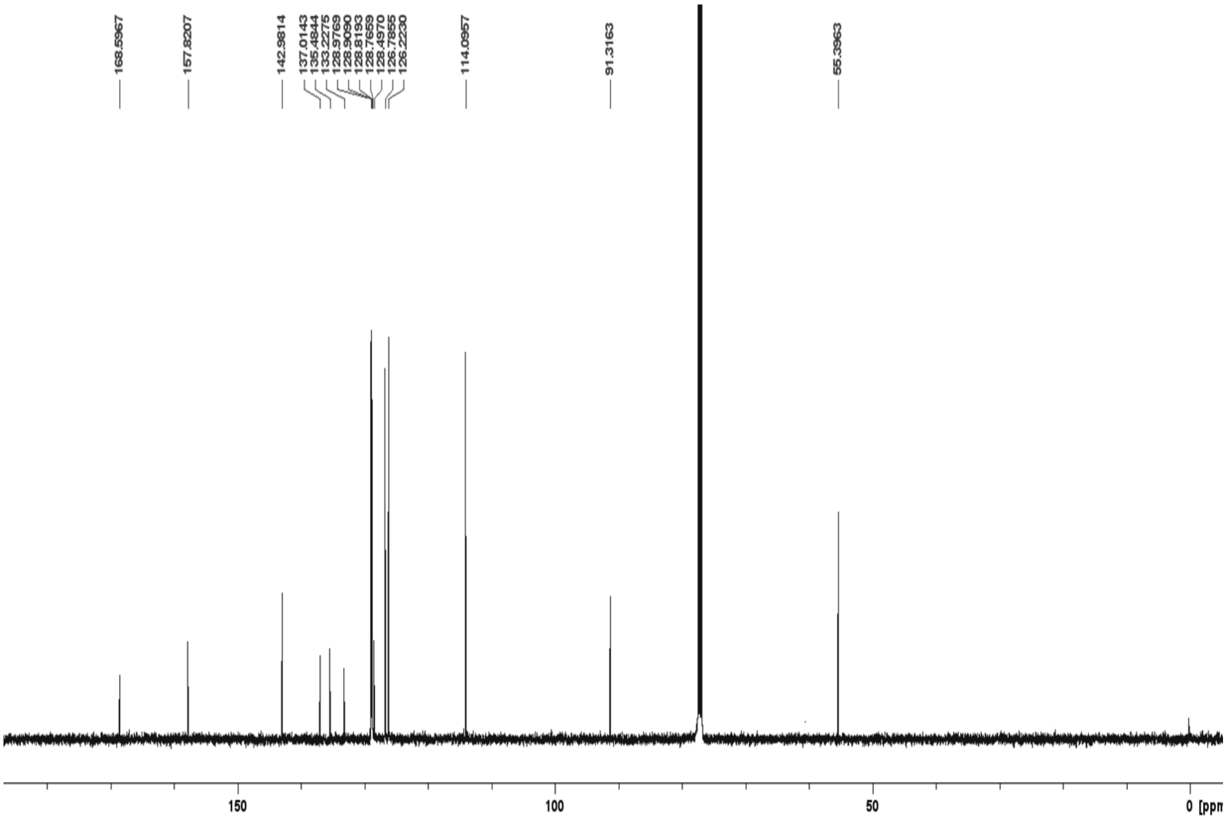

^{1}H-NMR ($CDCl_3$, 500 MHz) of compound 22.

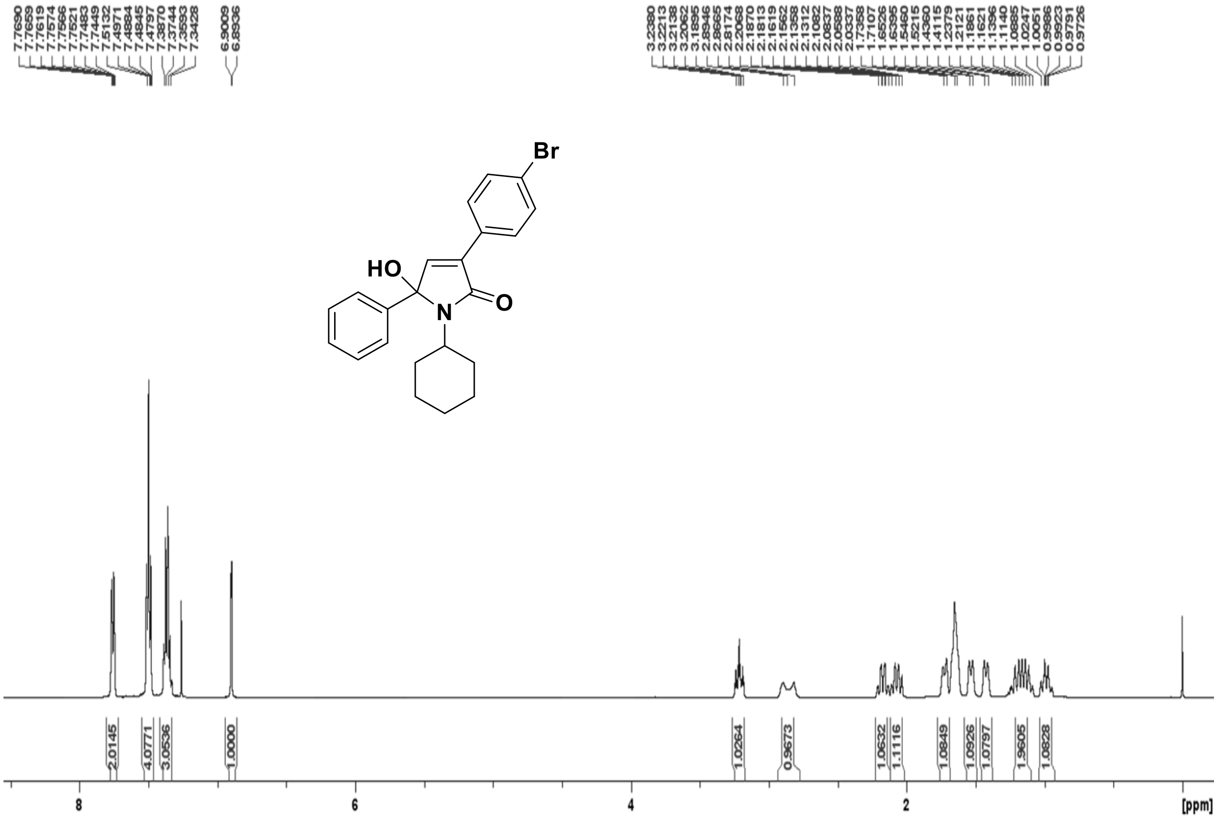

^{13}C-NMR ($CDCl_3$, 125 MHz) of compound 22.

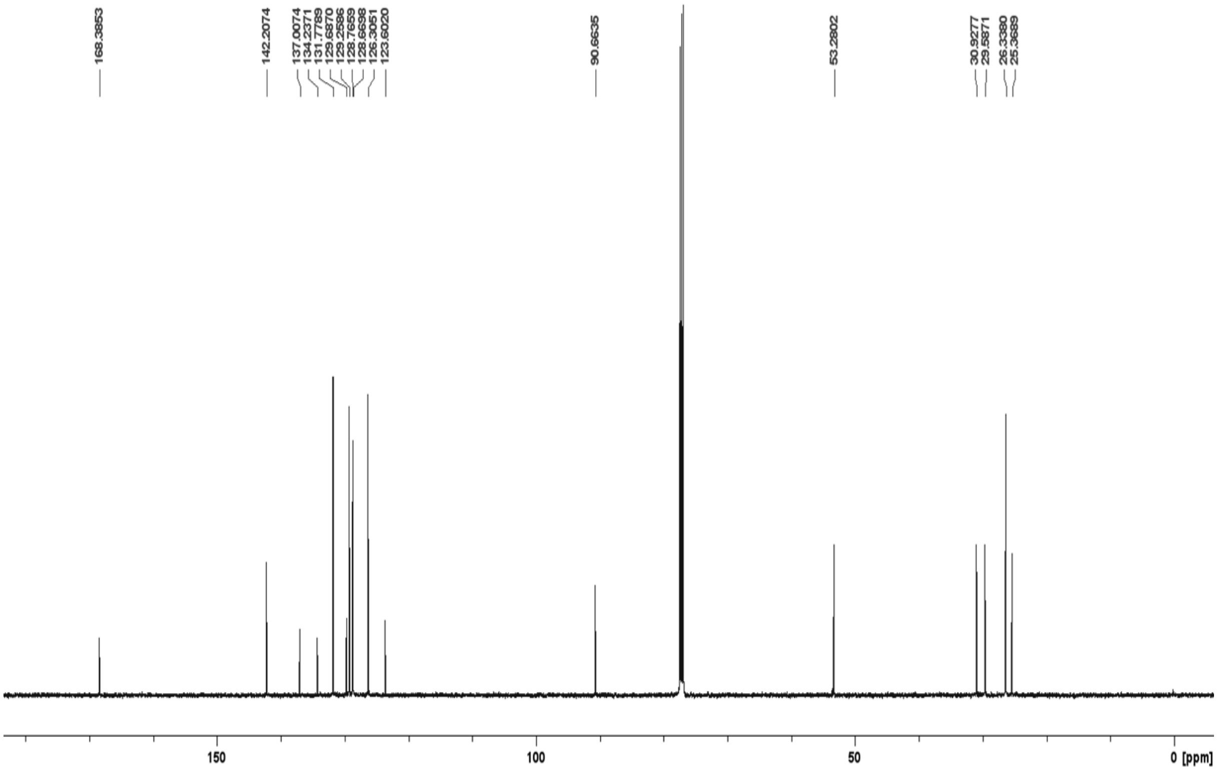

^{1}H-NMR (CDCl$_3$, 500 MHz) of compound 23.

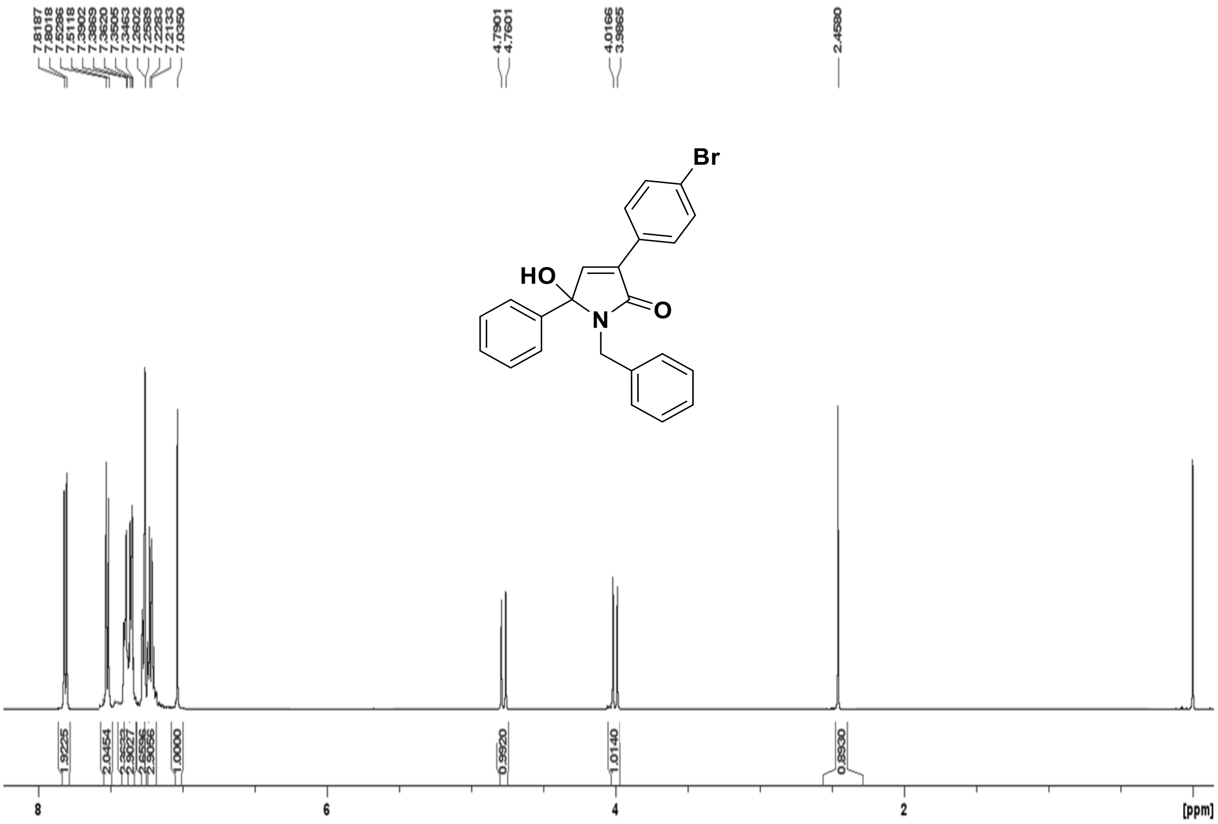

^{13}C-NMR (CDCl$_3$, 125 MHz) of compound 23.

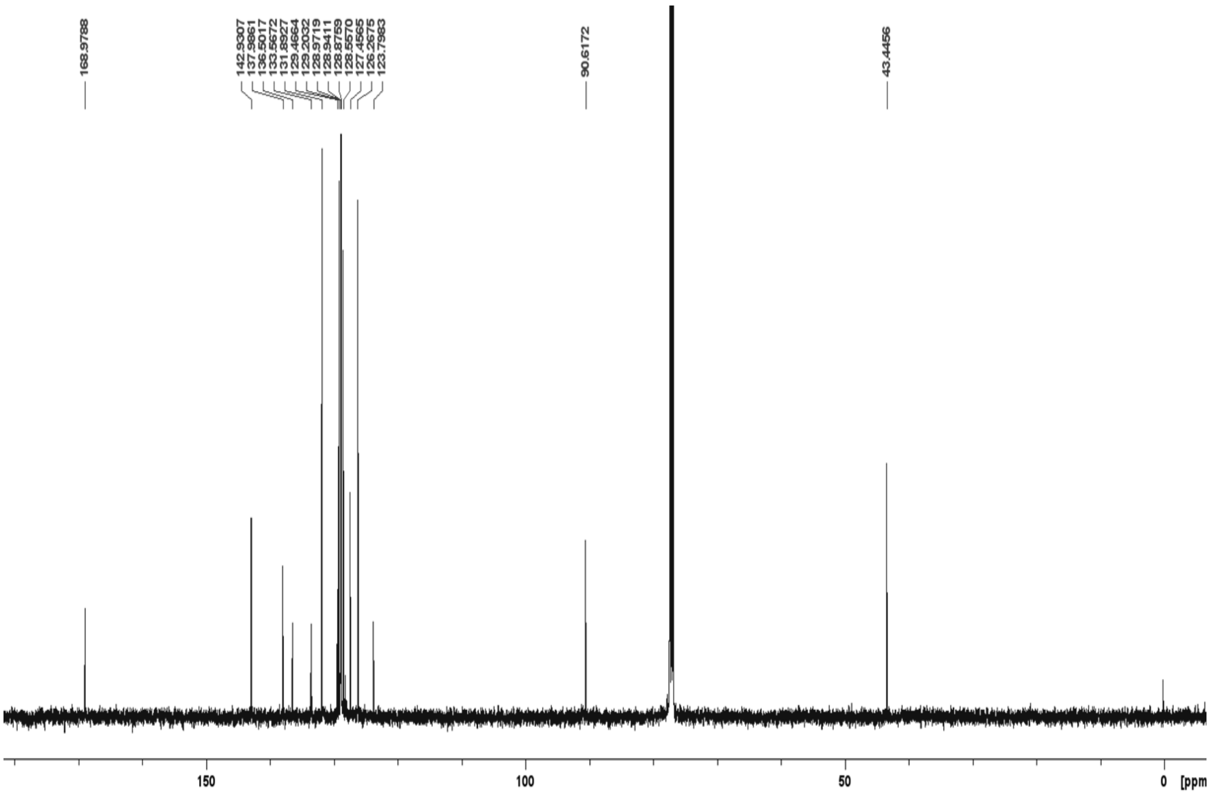

^{1}H-NMR ($CDCl_3$, 500 MHz) of compound 24.

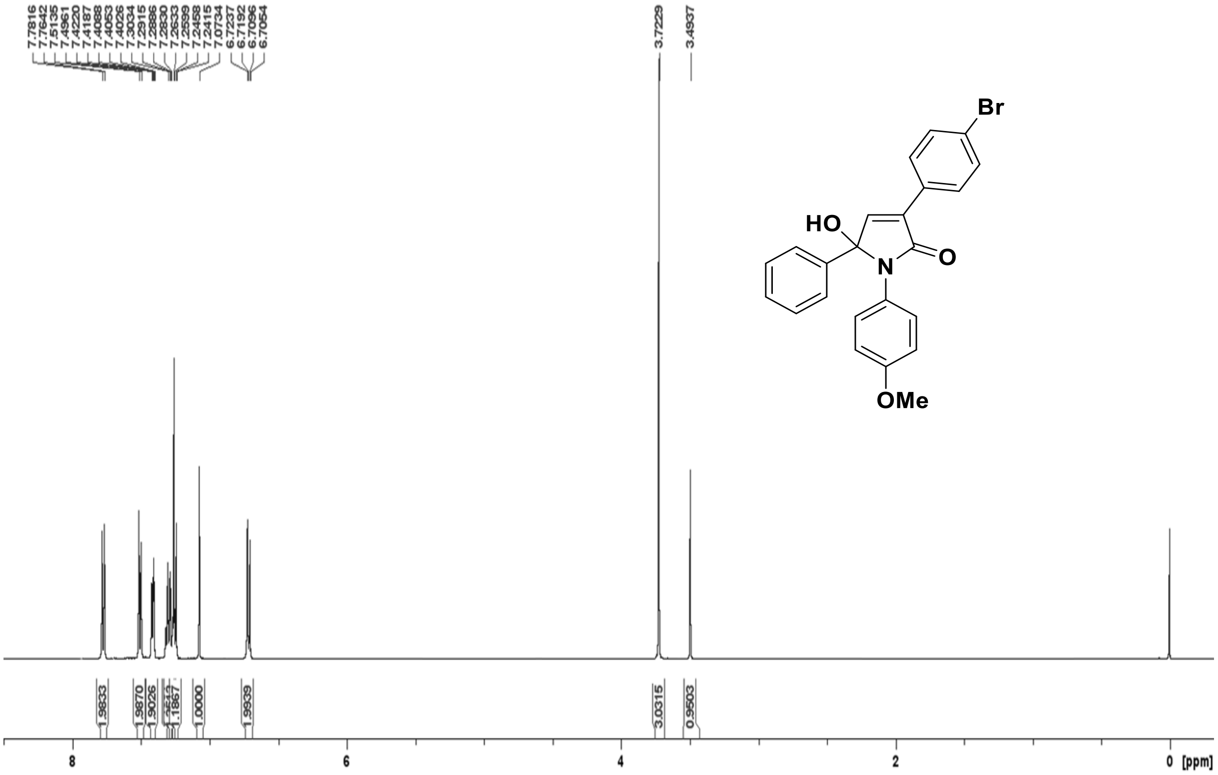

^{13}C-NMR ($CDCl_3$, 125 MHz) of compound 24.

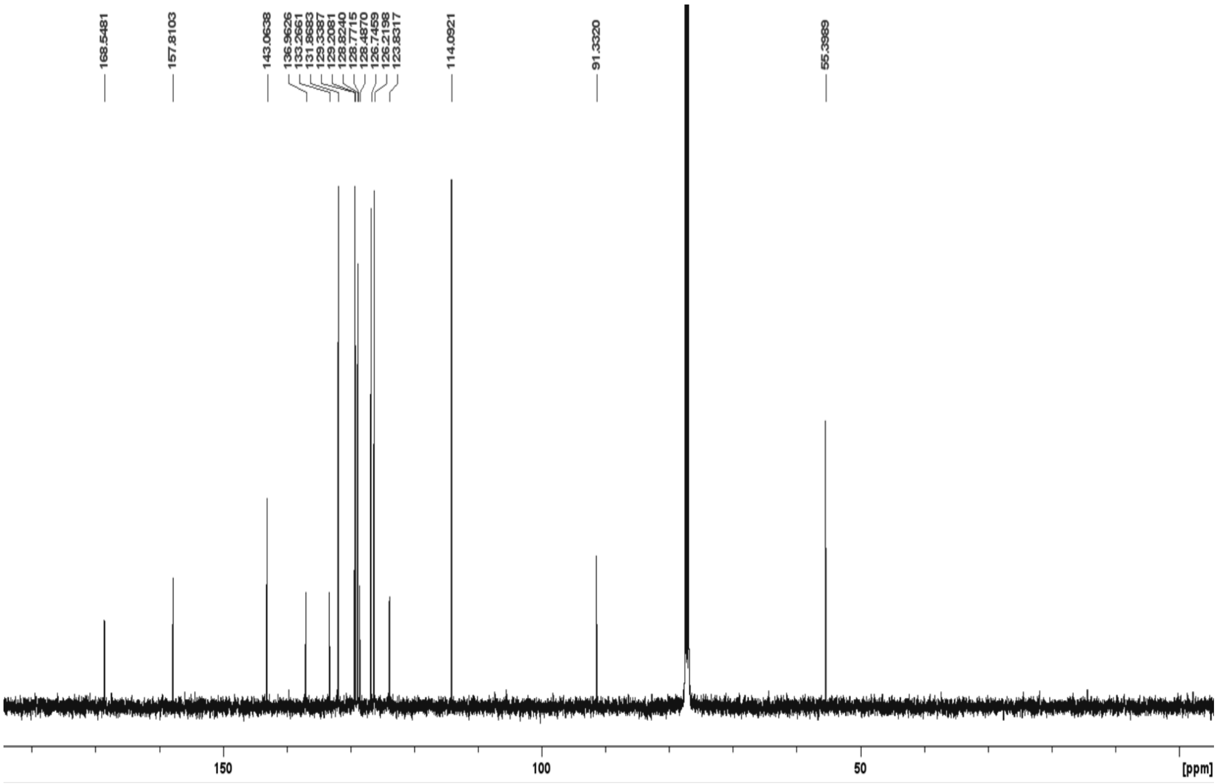

^{1}H-NMR ($CDCl_3$, 500 MHz) of compound 25.

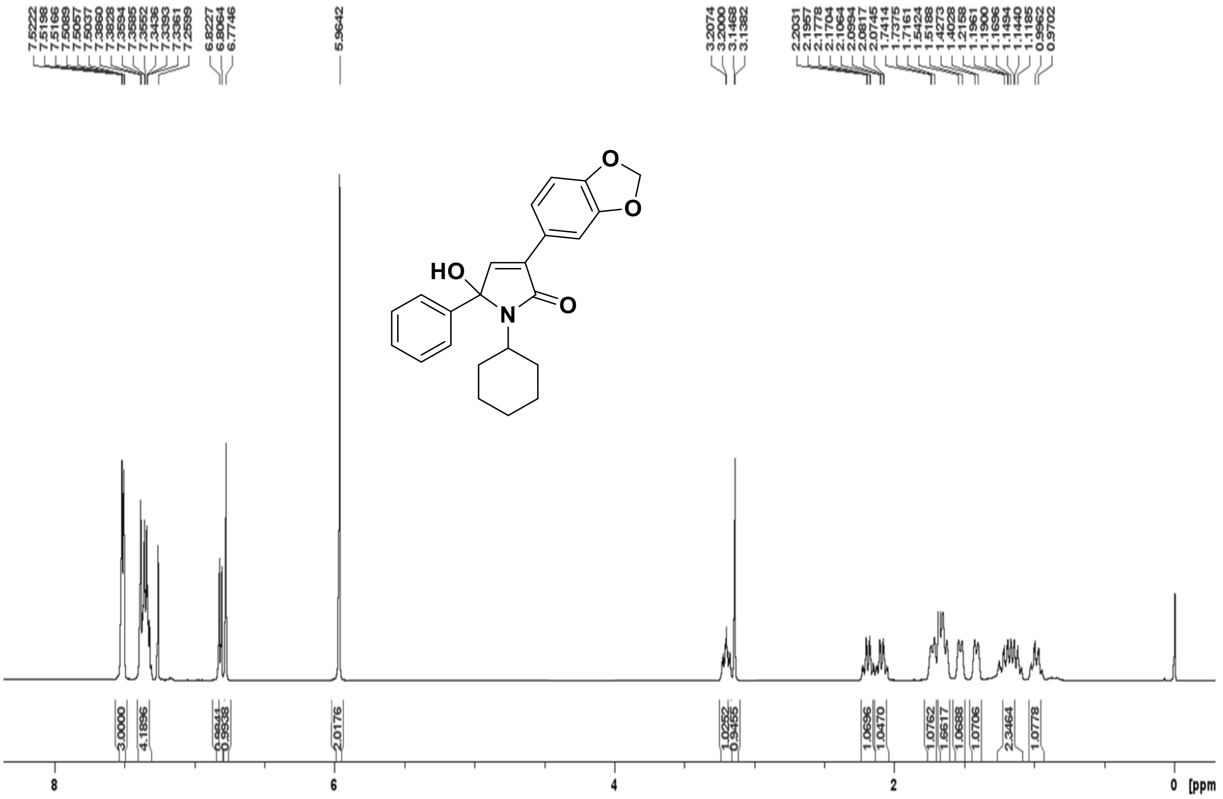

^{13}C-NMR ($CDCl_3$, 125 MHz) of compound 25.

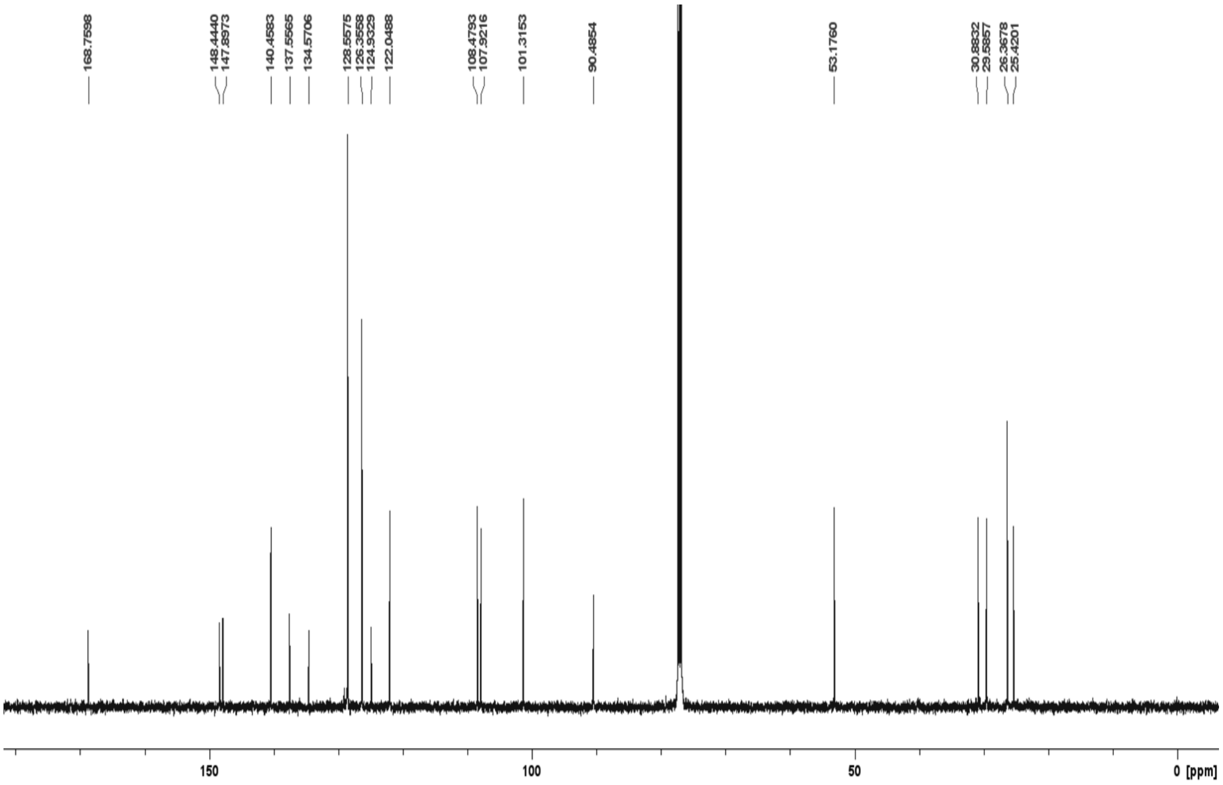

^{1}H-NMR (CDCl$_3$, 500 MHz) of compound 26.

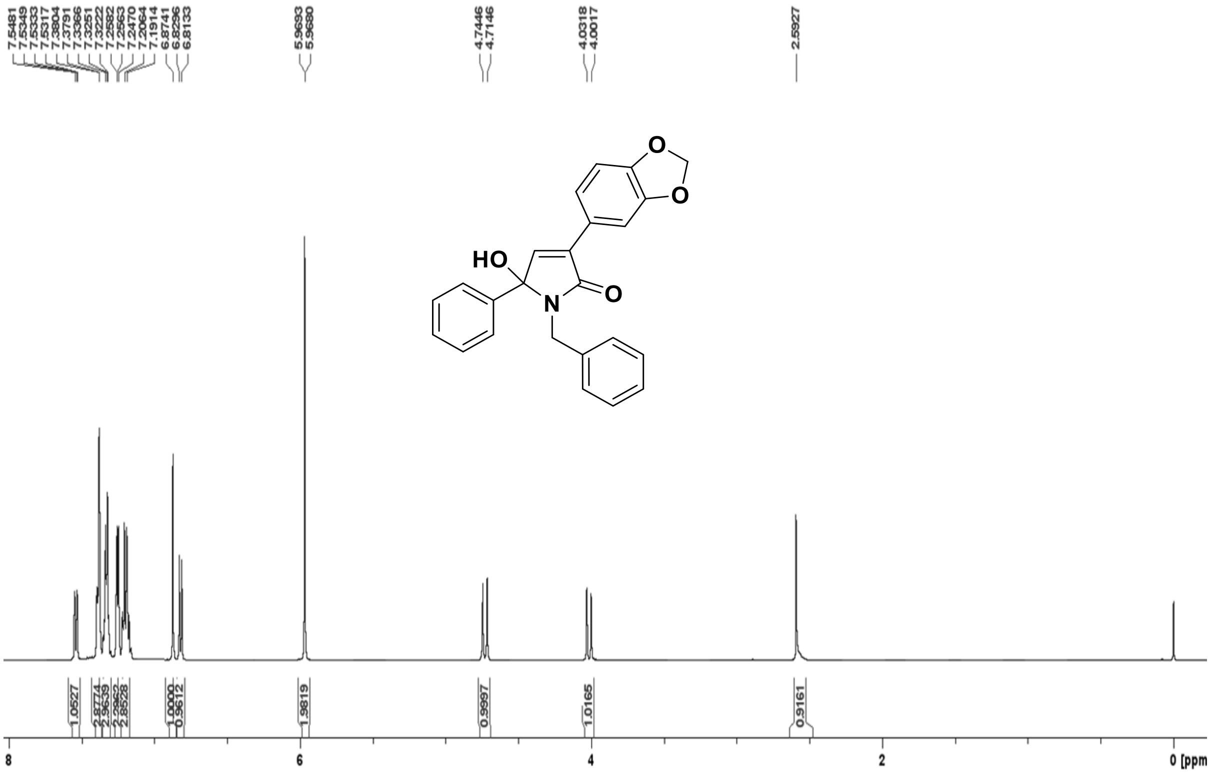

^{13}C-NMR (CDCl$_3$, 125 MHz) of compound 26.

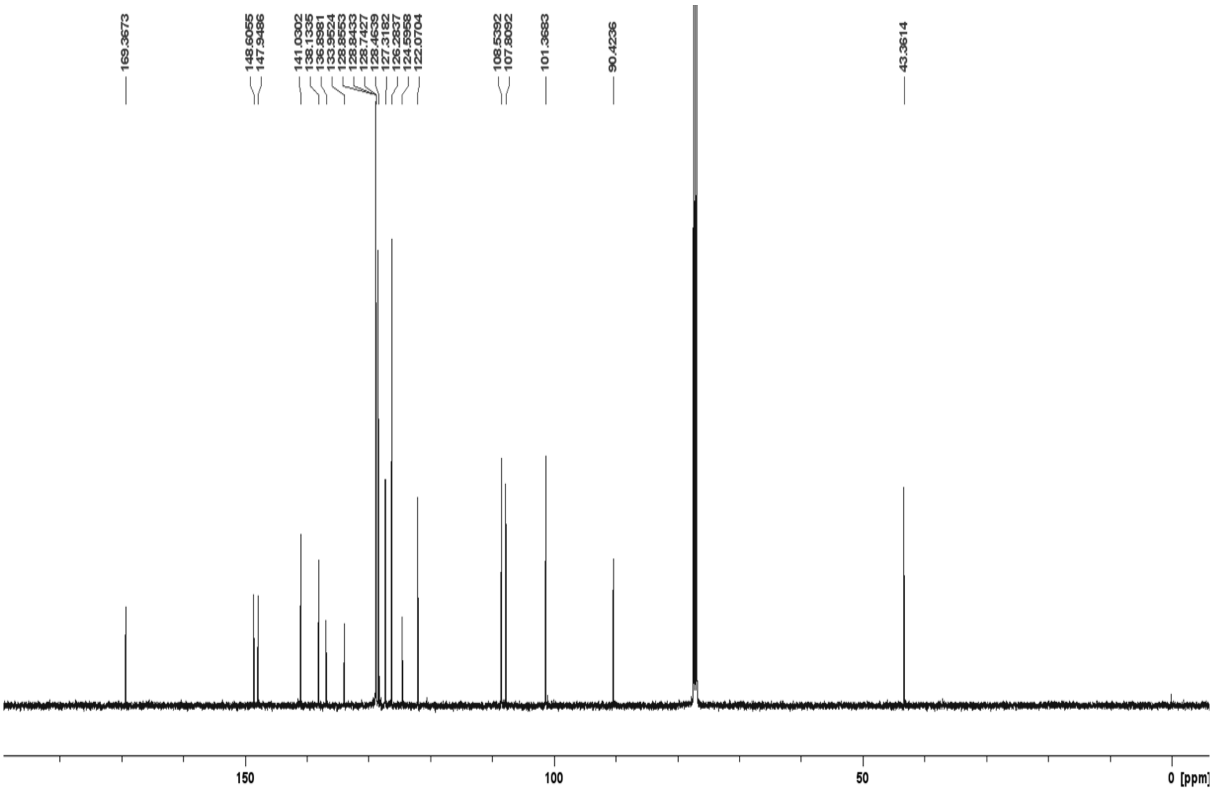

^{1}H-NMR ($CDCl_3$, 500 MHz) of compound 27.

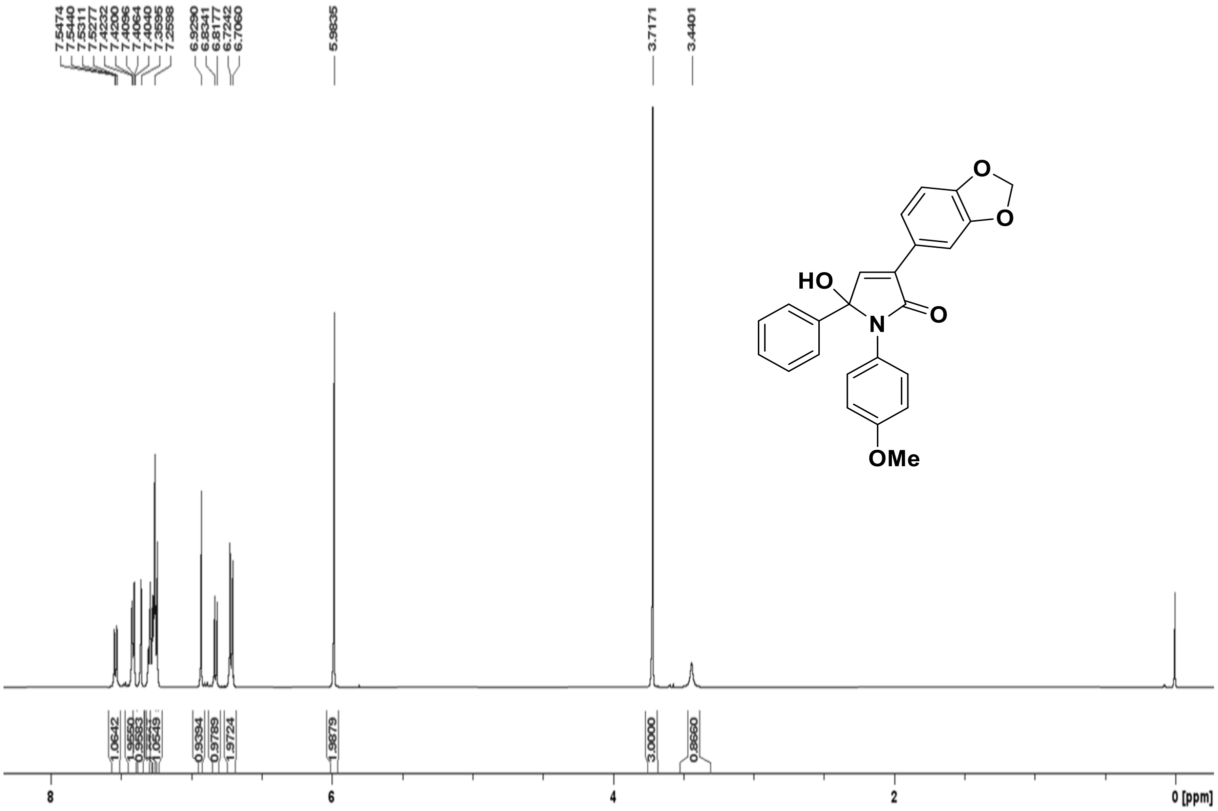

^{13}C-NMR ($CDCl_3$, 125 MHz) of compound 27.

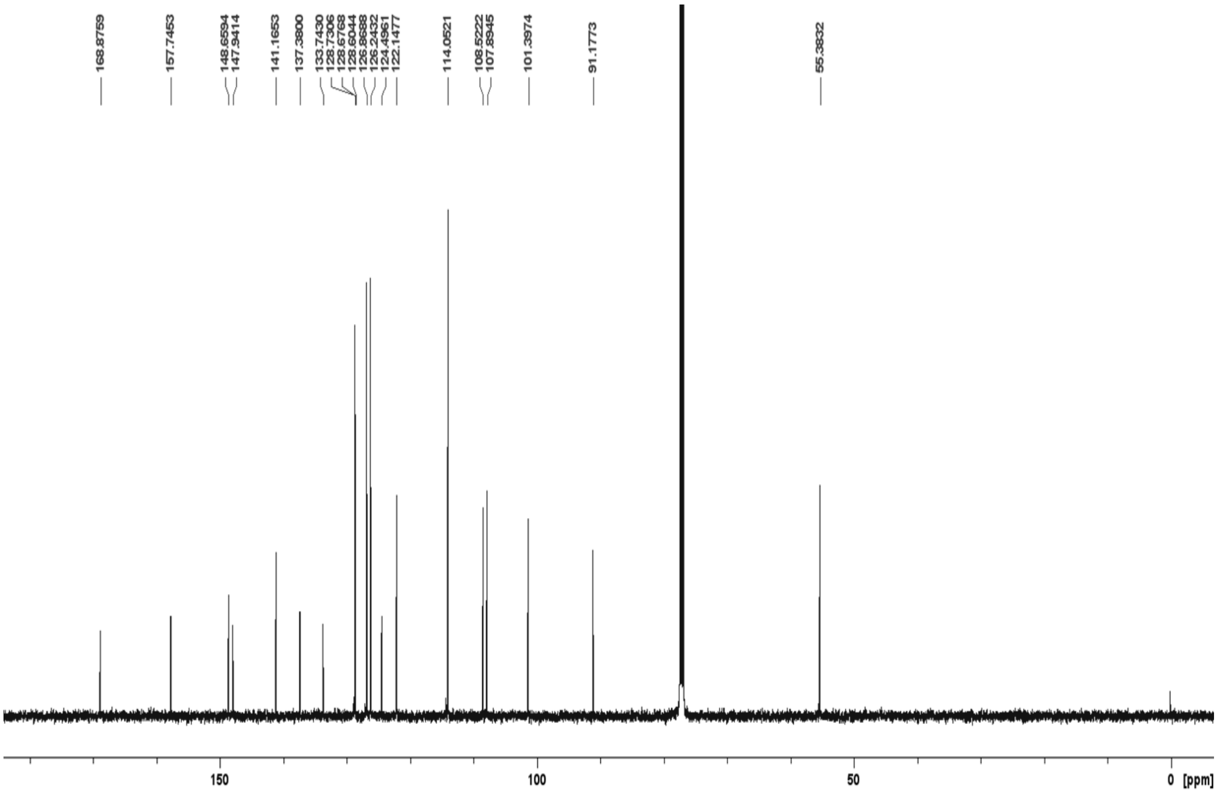

^{1}H-NMR (CDCl$_3$, 600 MHz) of compound 28.

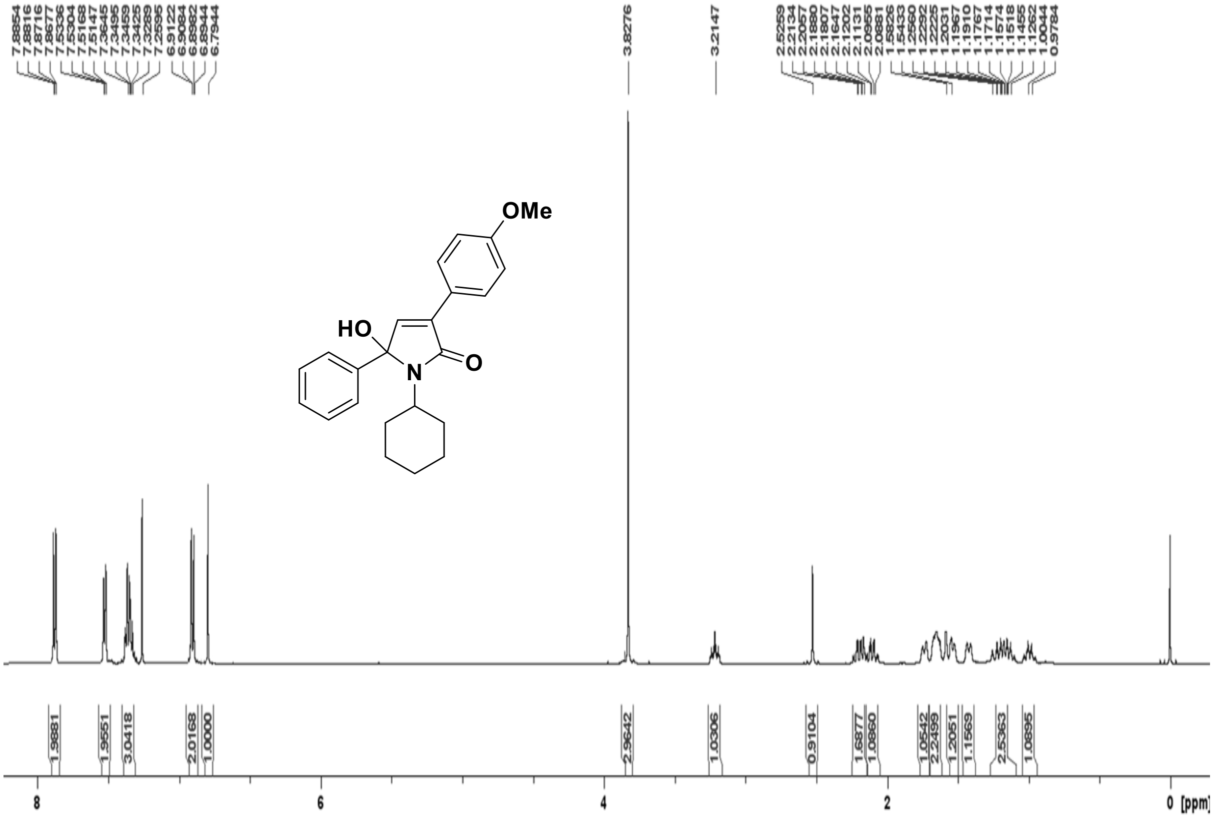

^{13}C-NMR (CDCl$_3$, 150 MHz) of compound 28.

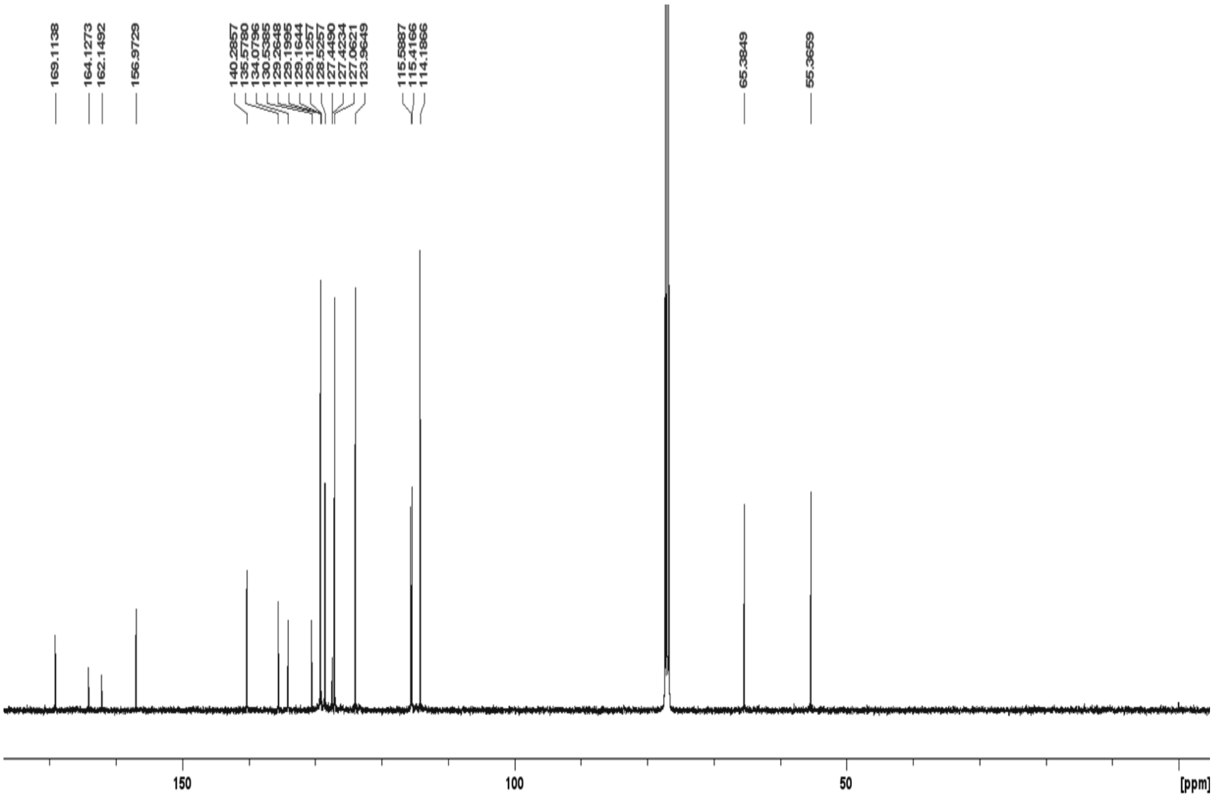

^{1}H-NMR ($CDCl_3$, 600 MHz) of compound 29.

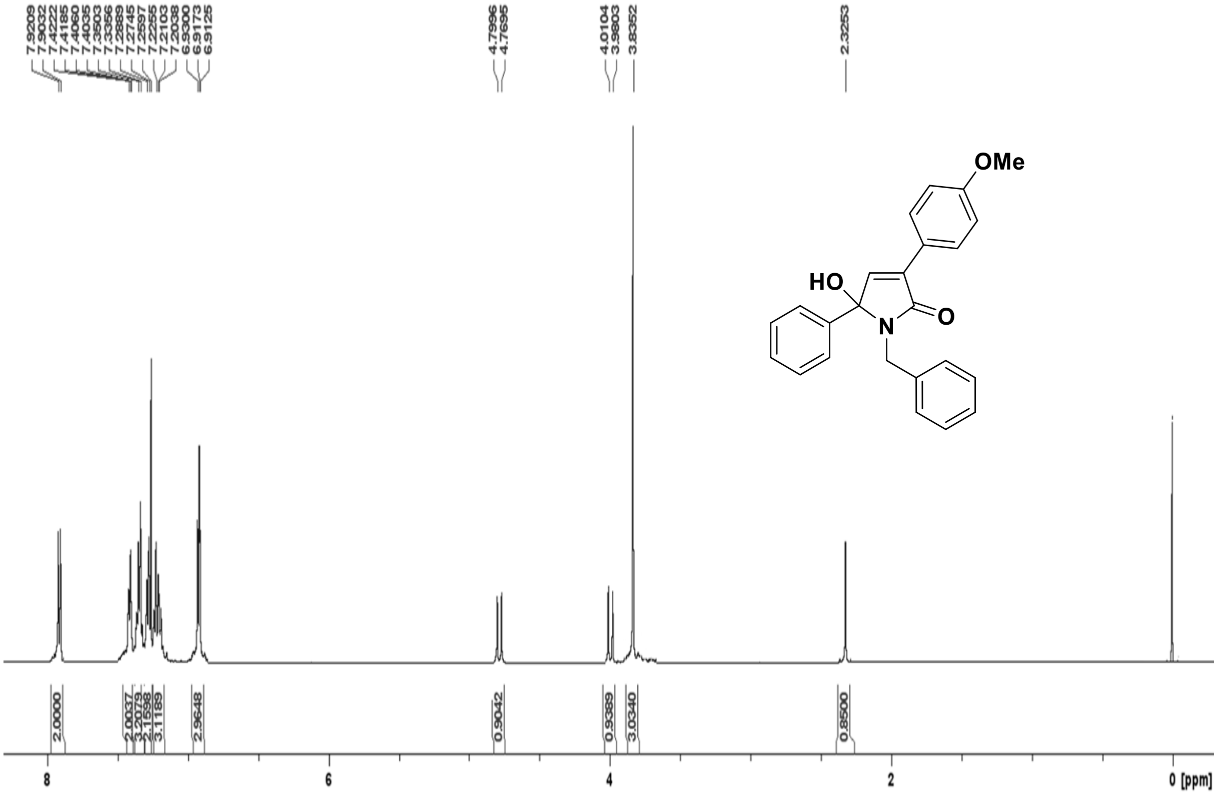

^{13}C-NMR ($CDCl_3$, 150 MHz) of compound 29.

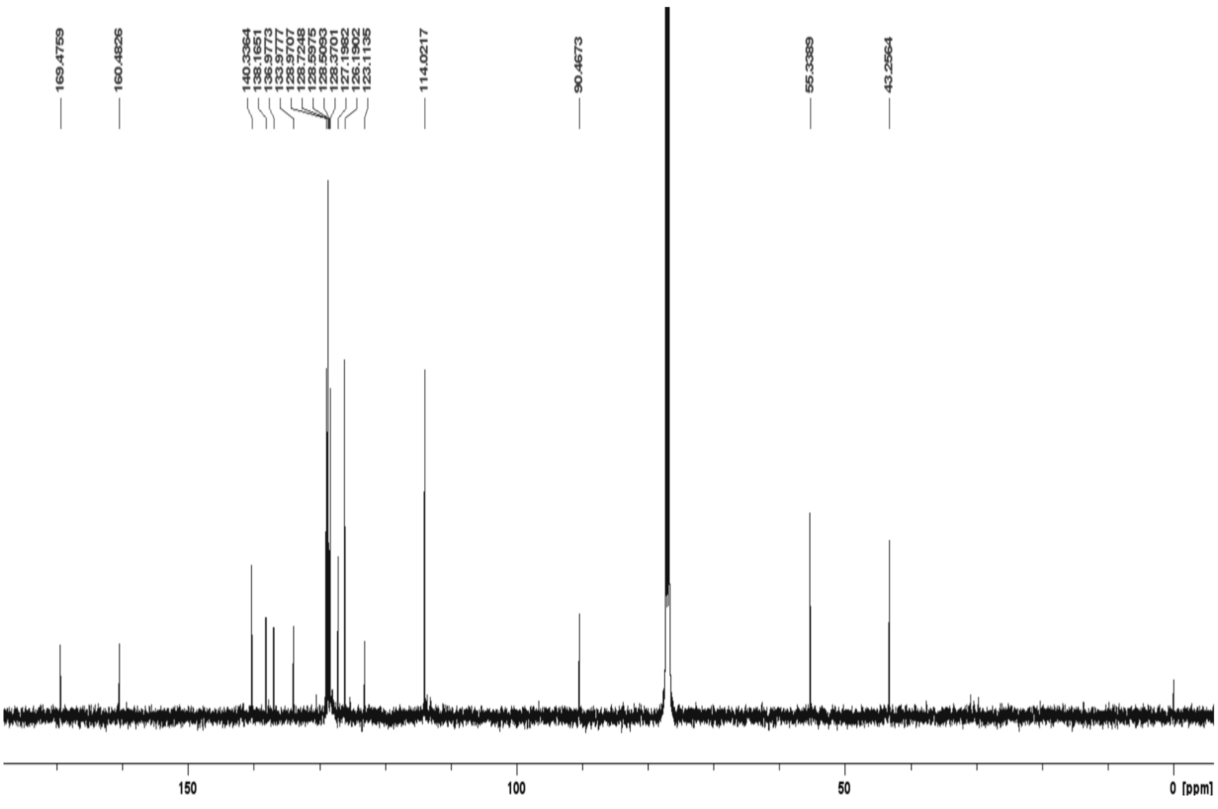

^{1}H-NMR ($CDCl_3$, 600 MHz) of compound 30.

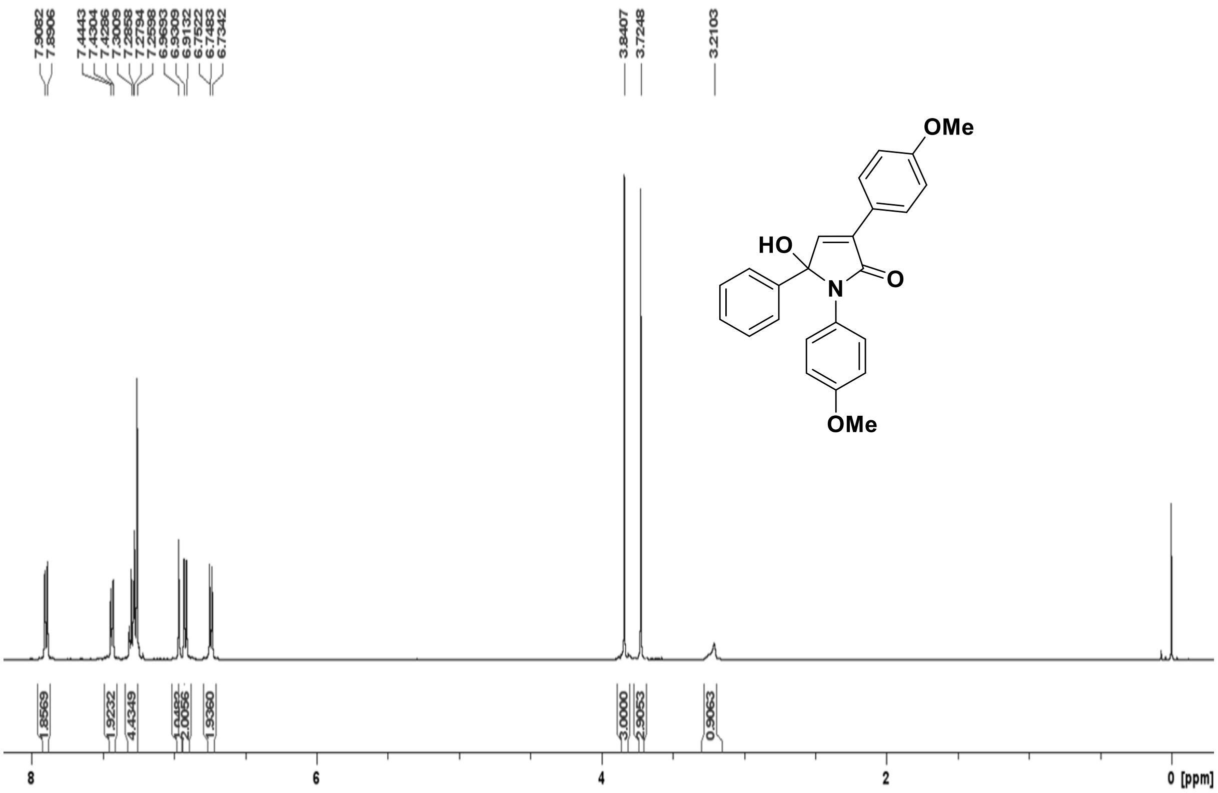

^{13}C-NMR ($CDCl_3$, 150 MHz) of compound 30.

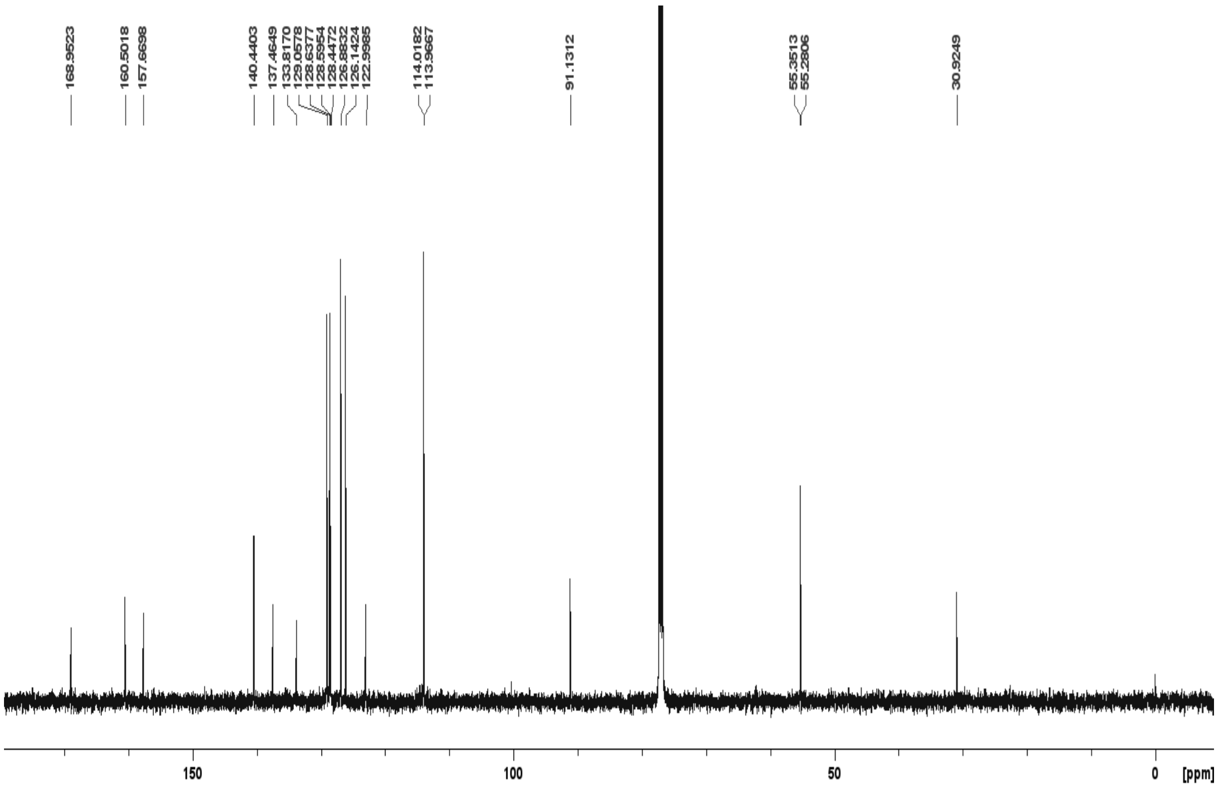

^{1}H-NMR ($CDCl_3$, 500 MHz) of compound 31.

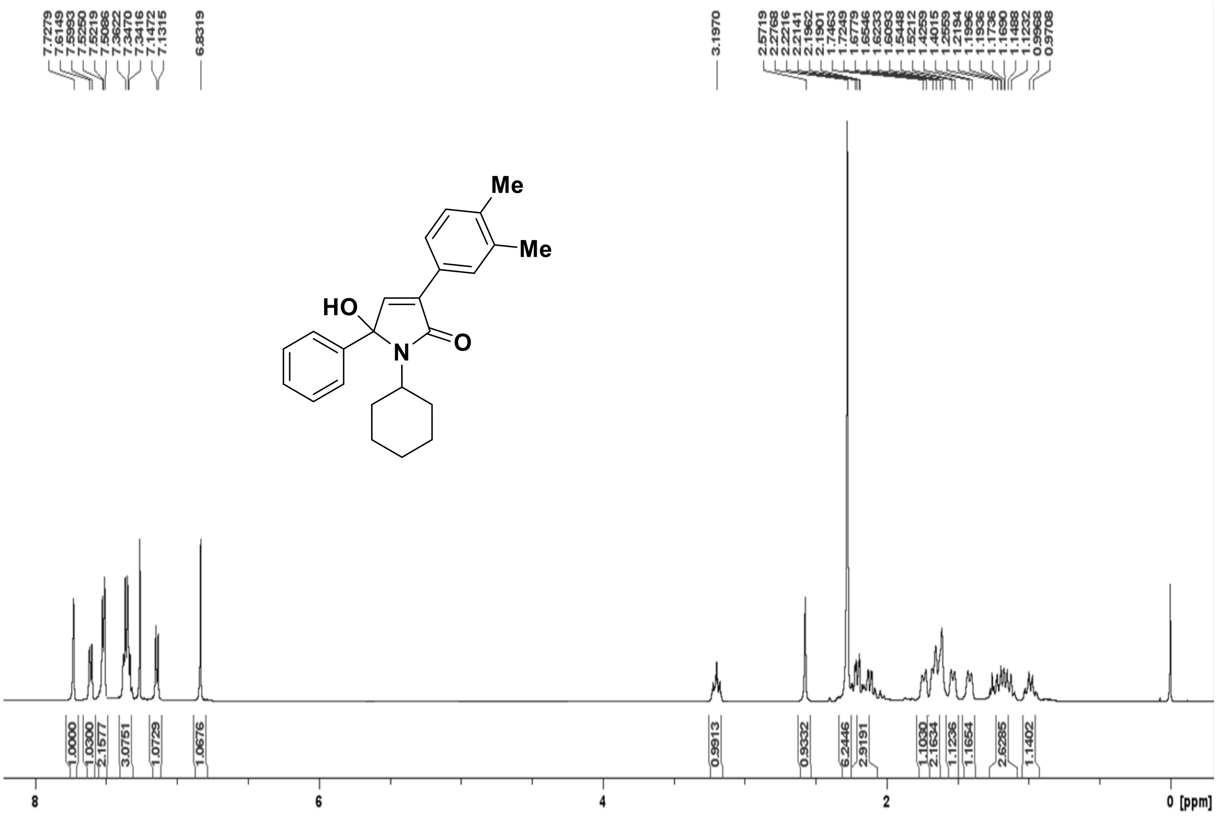

^{13}C-NMR ($CDCl_3$, 125 MHz) of compound 31.

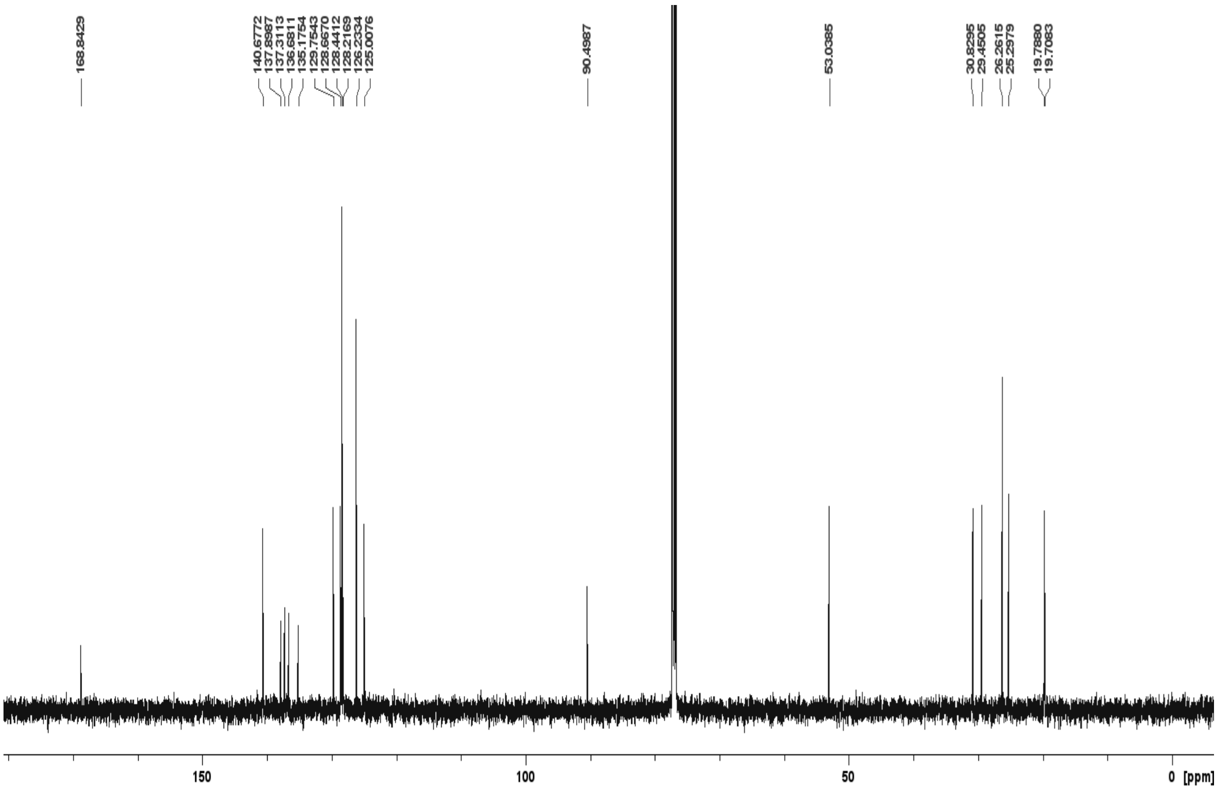

^{1}H-NMR (CDCl$_3$, 600 MHz) of compound 32.

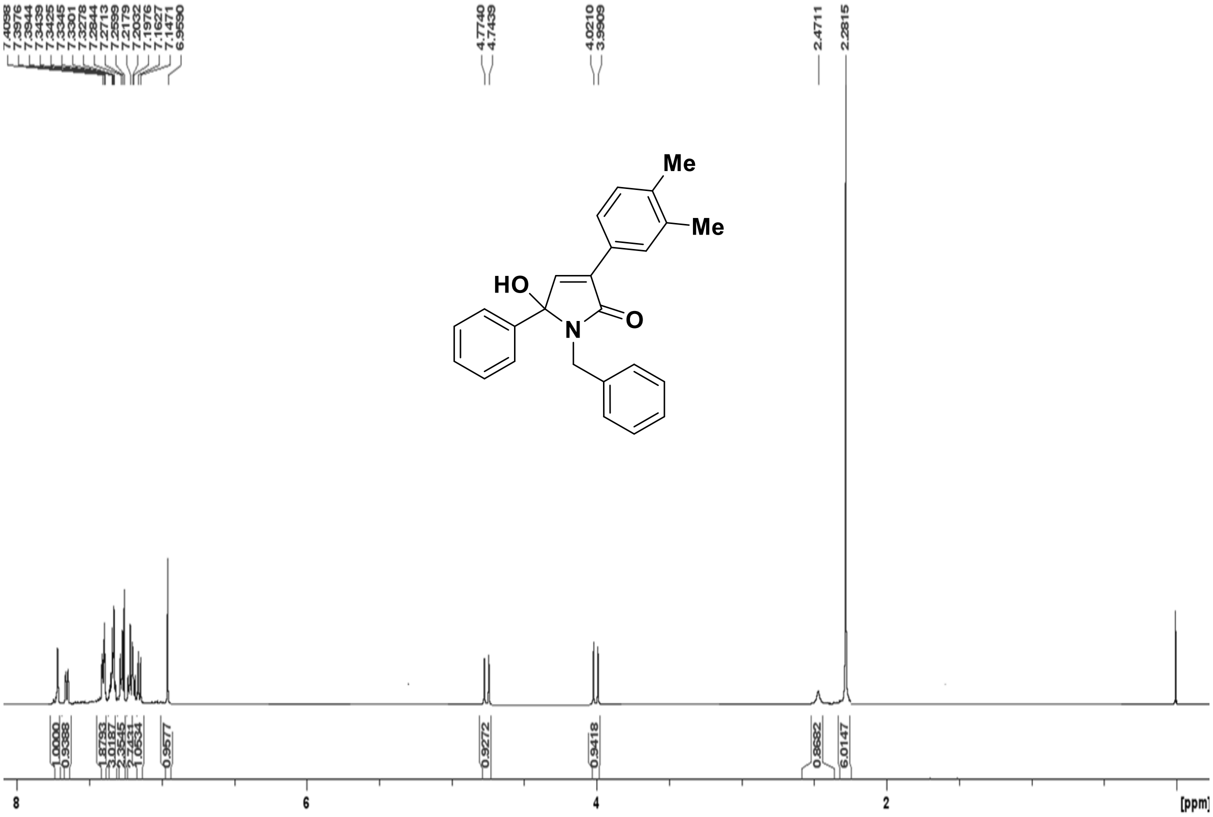

^{13}C-NMR (CDCl$_3$, 150 MHz) of compound 32.

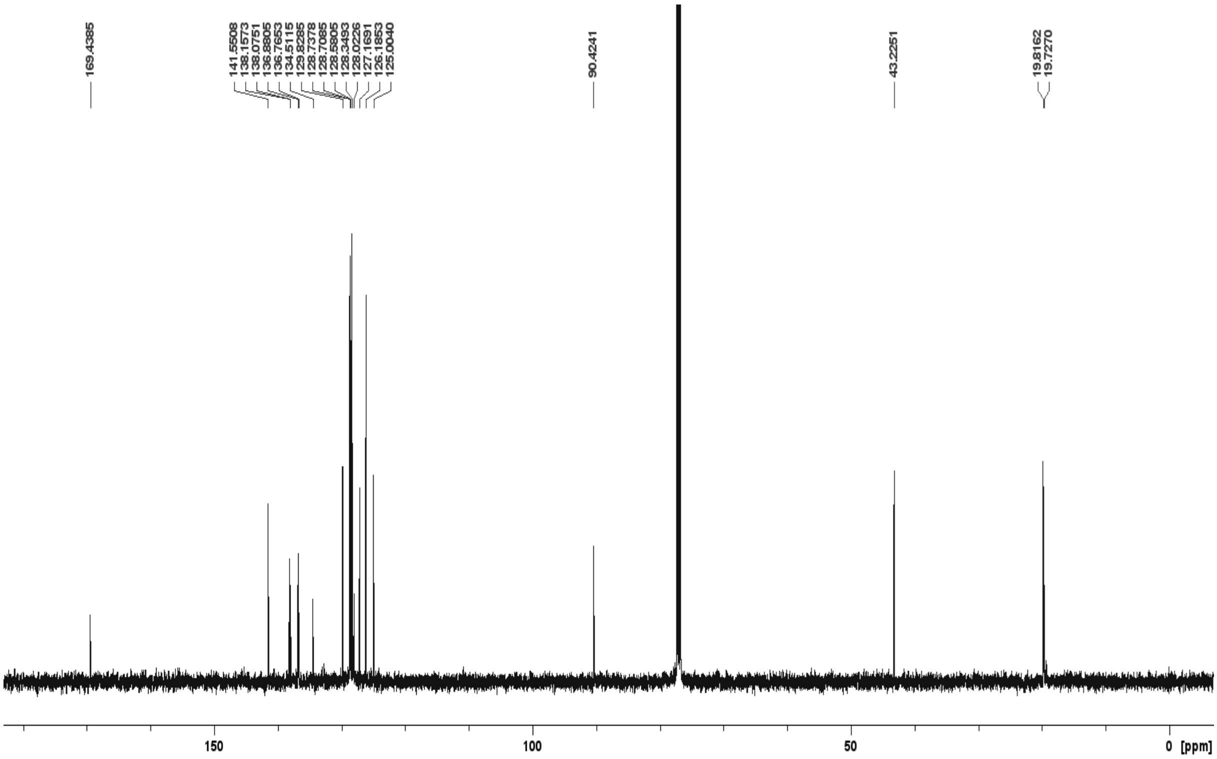

^{1}H-NMR (CDCl$_3$, 500 MHz) of compound 33.

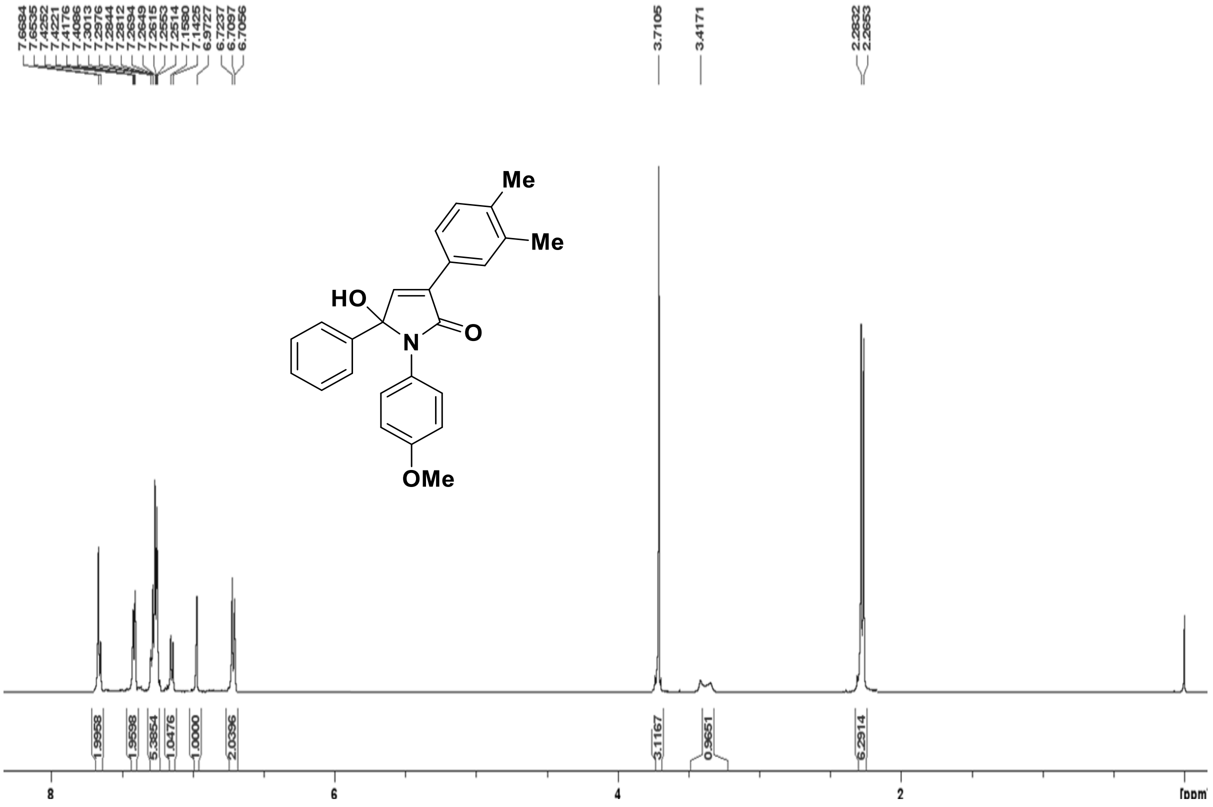

^{13}C-NMR (CDCl$_3$, 125 MHz) of compound 33.

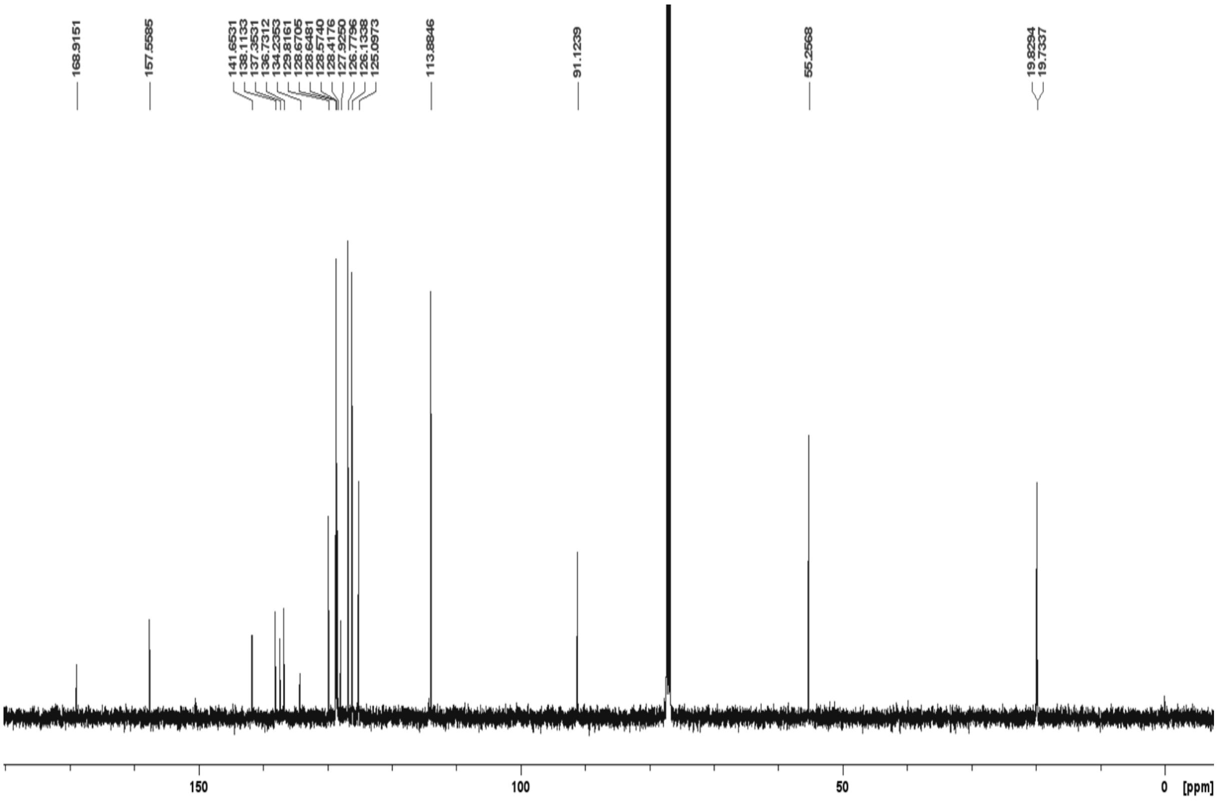

^{1}H-NMR ($CDCl_3$, 500 MHz) of compound 34.

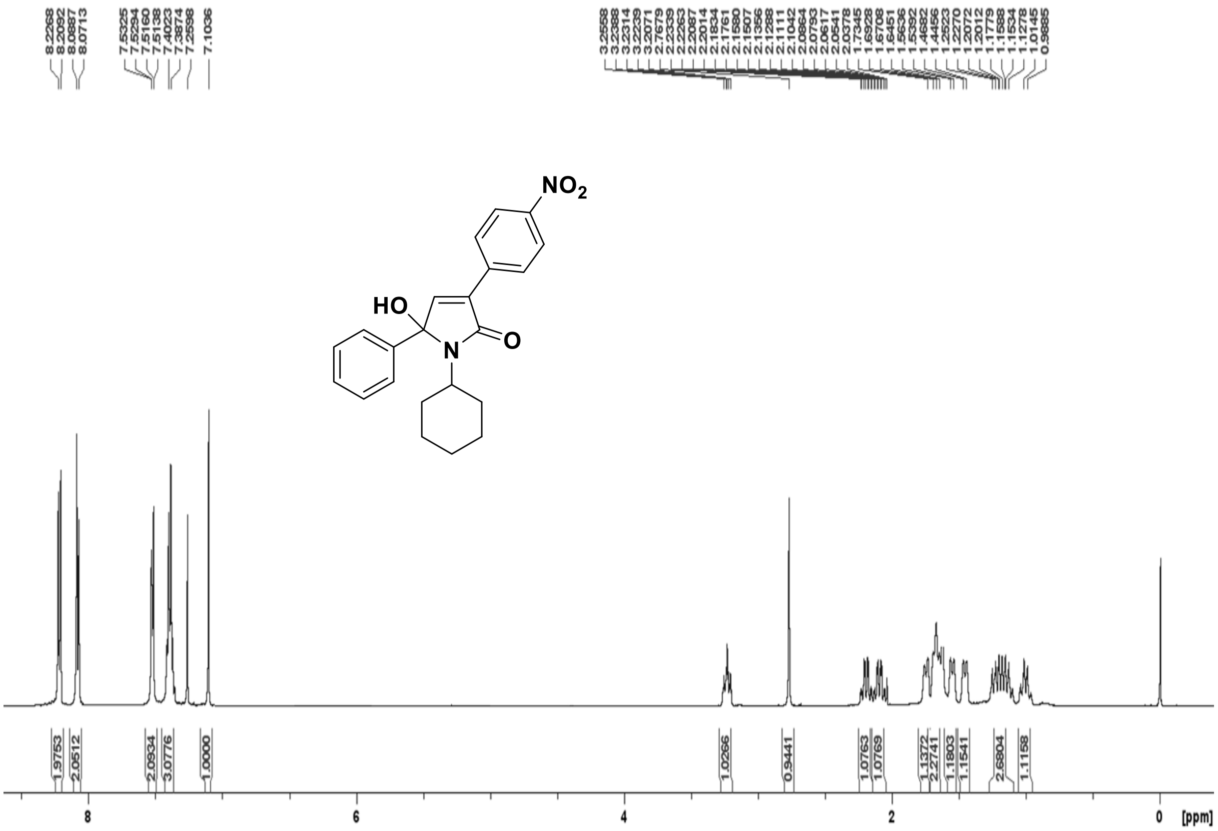

^{13}C-NMR ($CDCl_3$, 125 MHz) of compound 34.

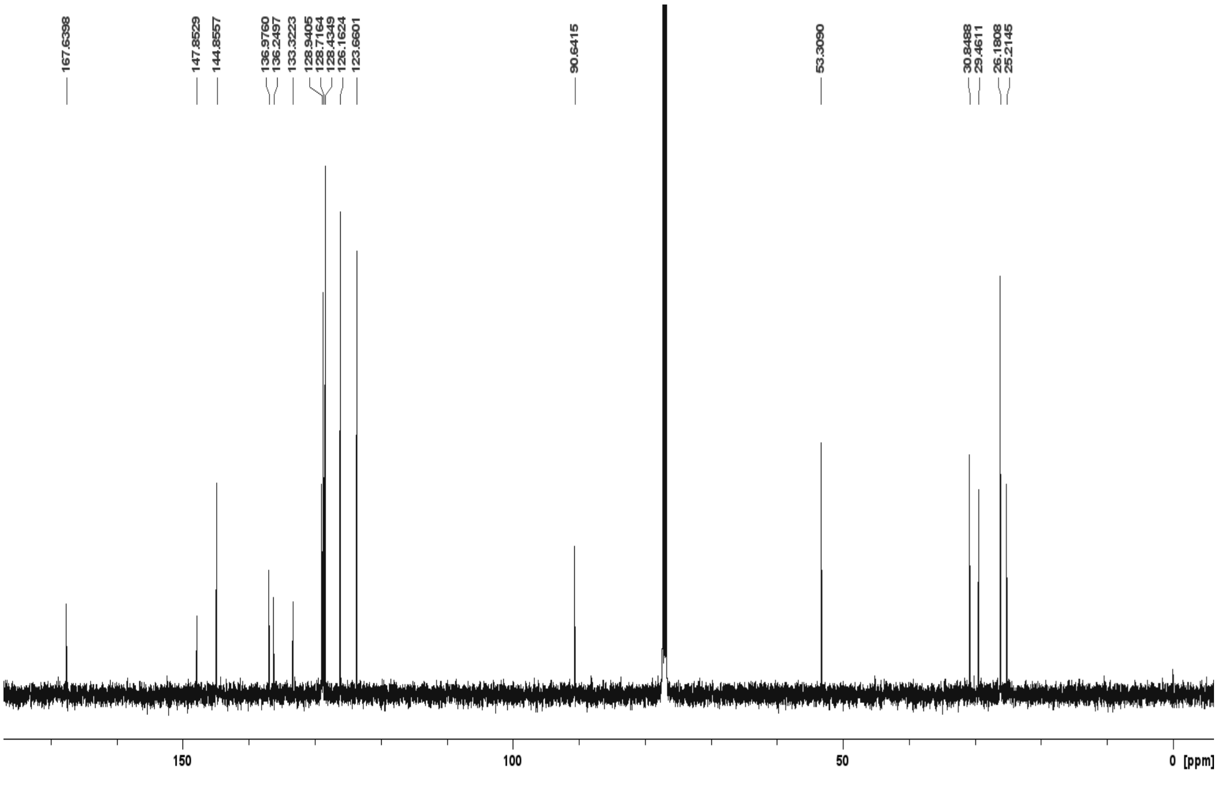

^{1}H-NMR ($CDCl_3$, 500 MHz) of compound 35.

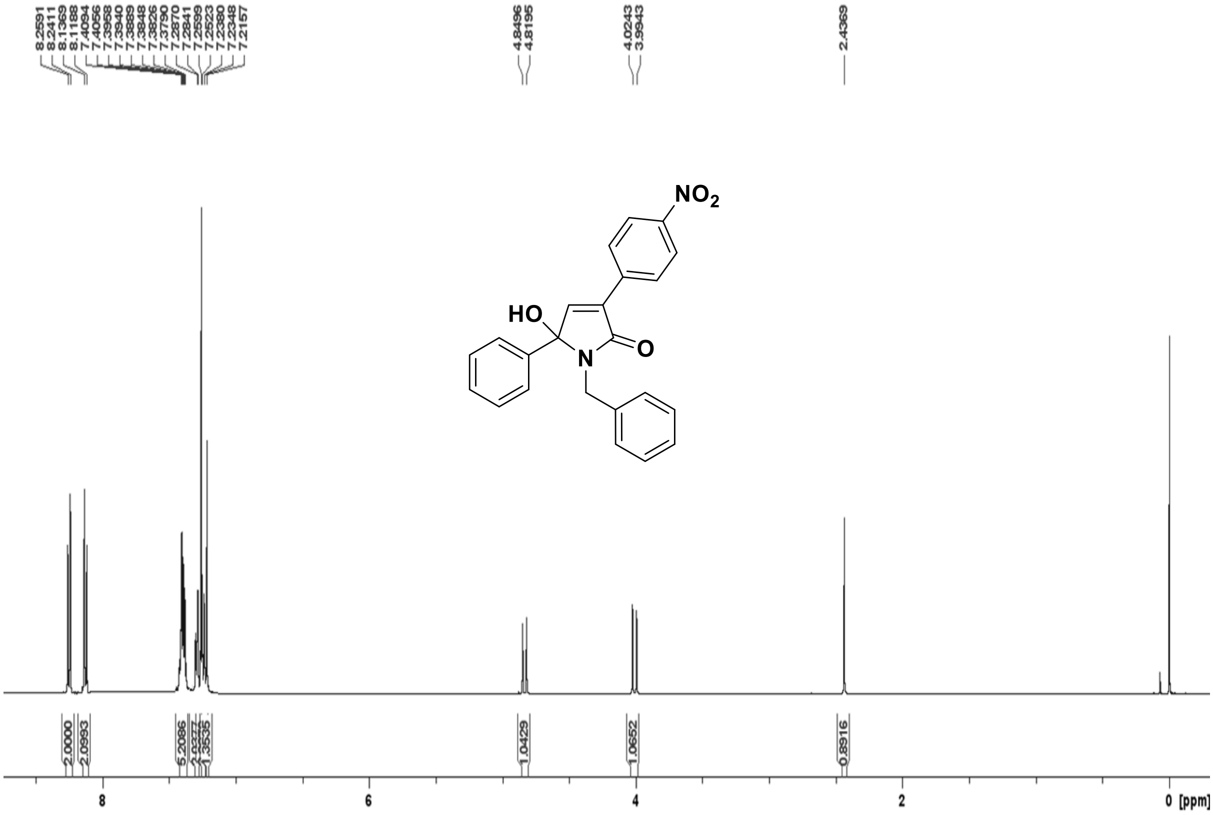

^{13}C-NMR ($CDCl_3$, 125 MHz) of compound 35.

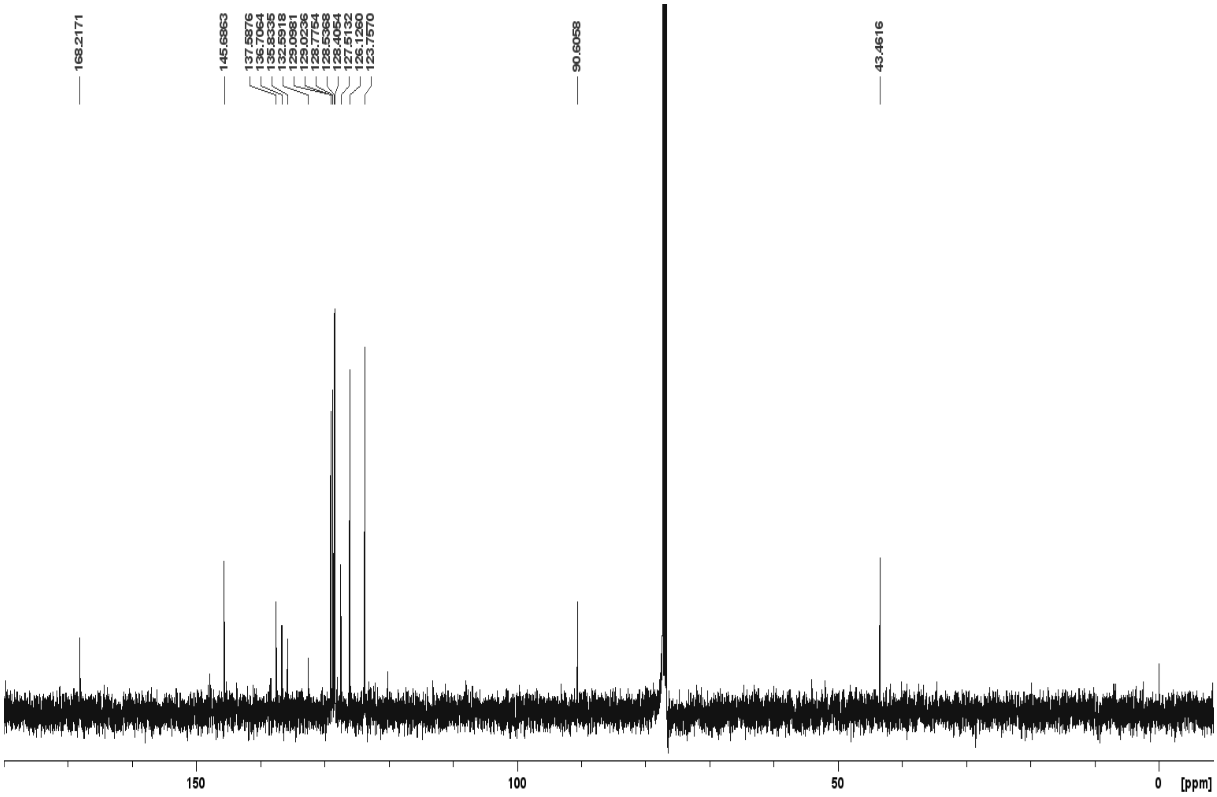

^{1}H-NMR ($CDCl_3$, 500 MHz) of compound 36.

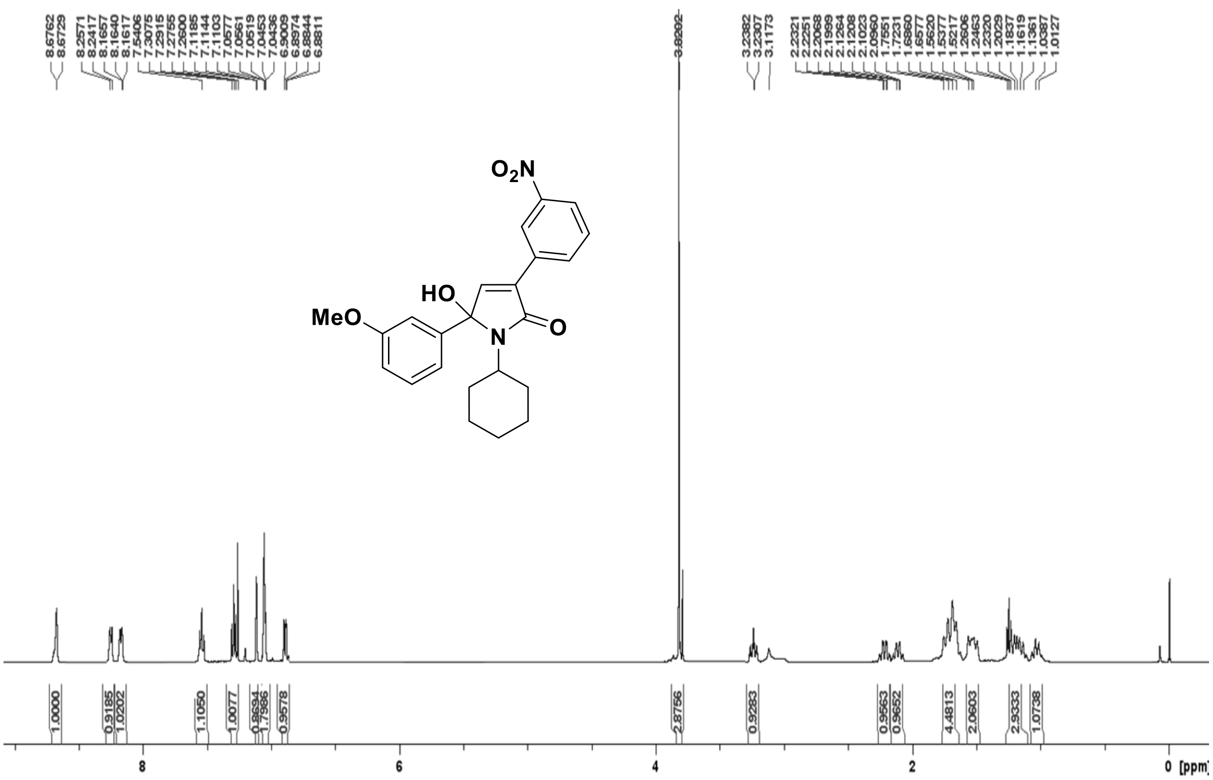

^{13}C-NMR ($CDCl_3$, 125 MHz) of compound 36.

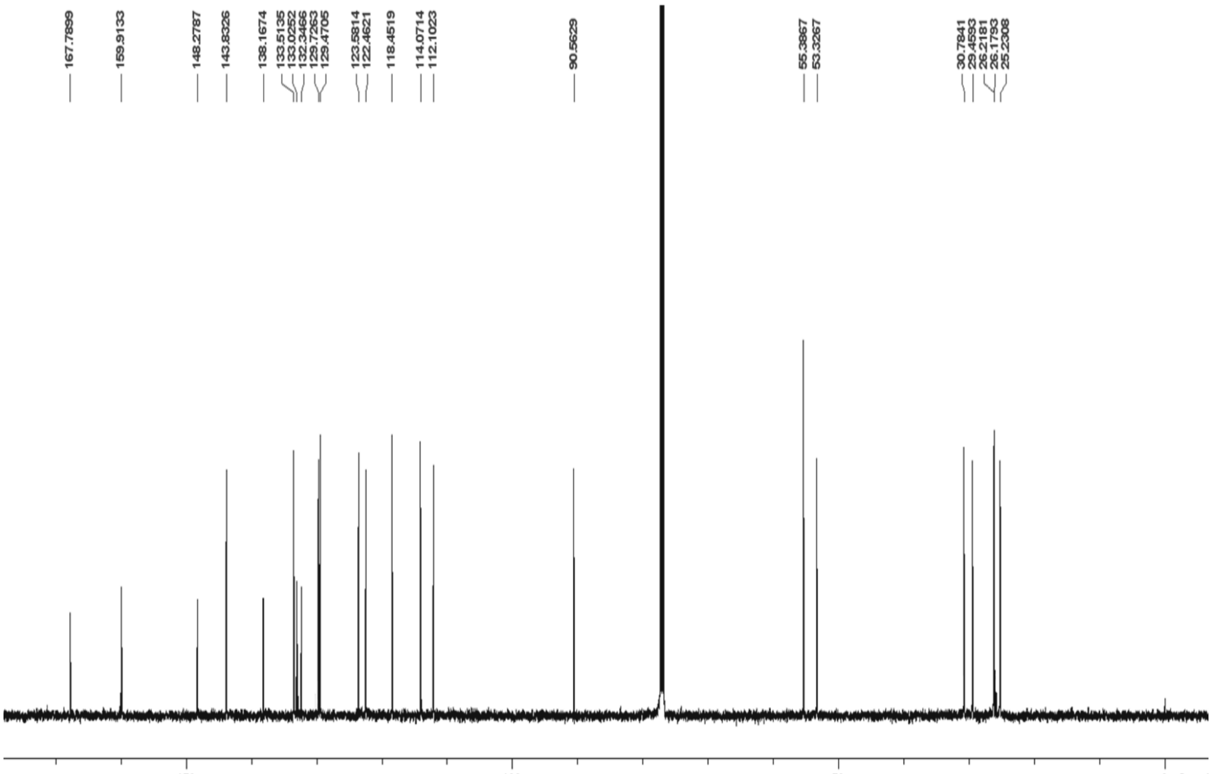

^{1}H-NMR ($CDCl_3$, 500 MHz) of compound 37.

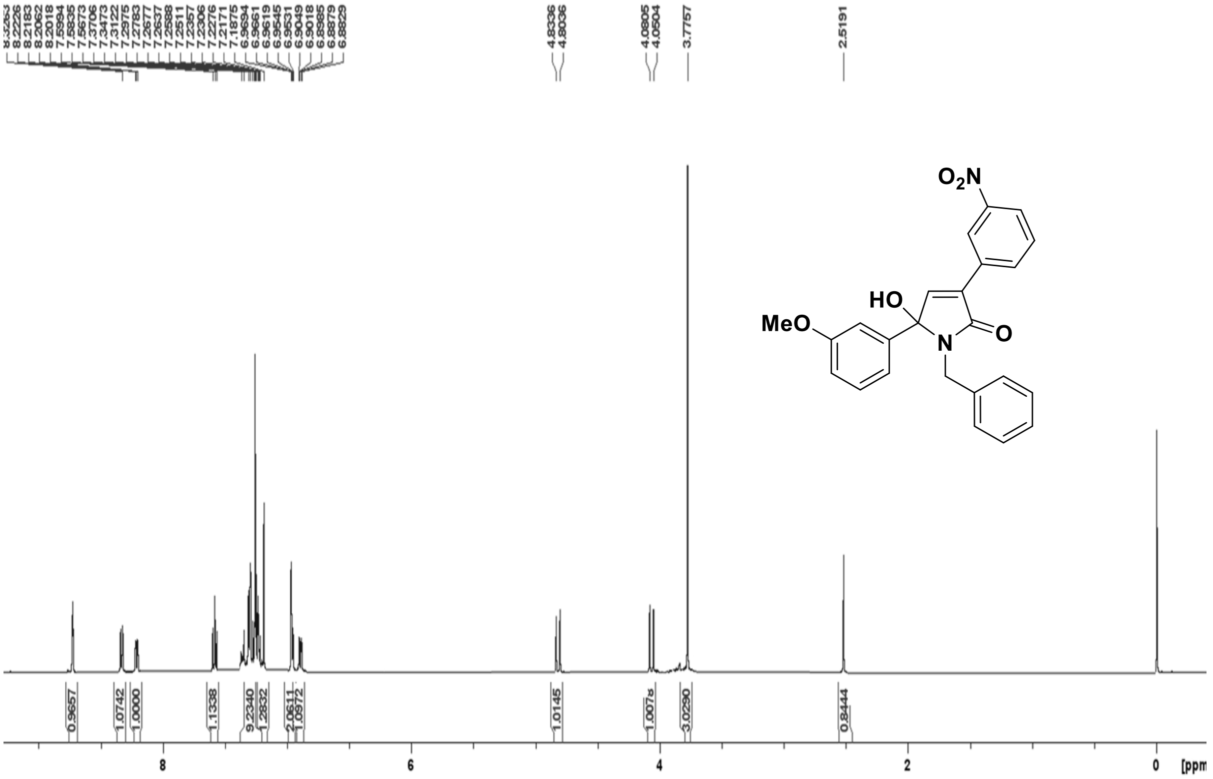

^{13}C-NMR ($CDCl_3$, 125 MHz) of compound 37.

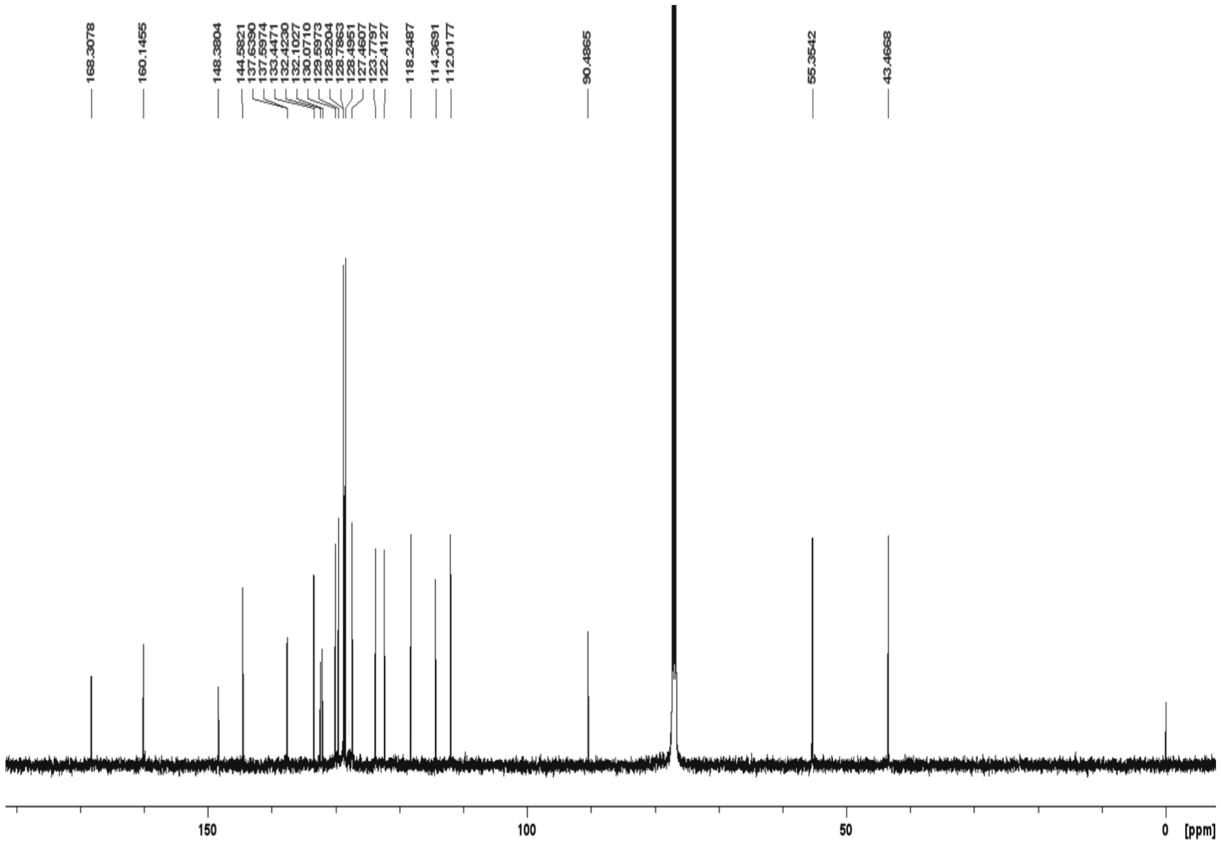

^{1}H-NMR ($CDCl_3$, 500 MHz) of compound 38.

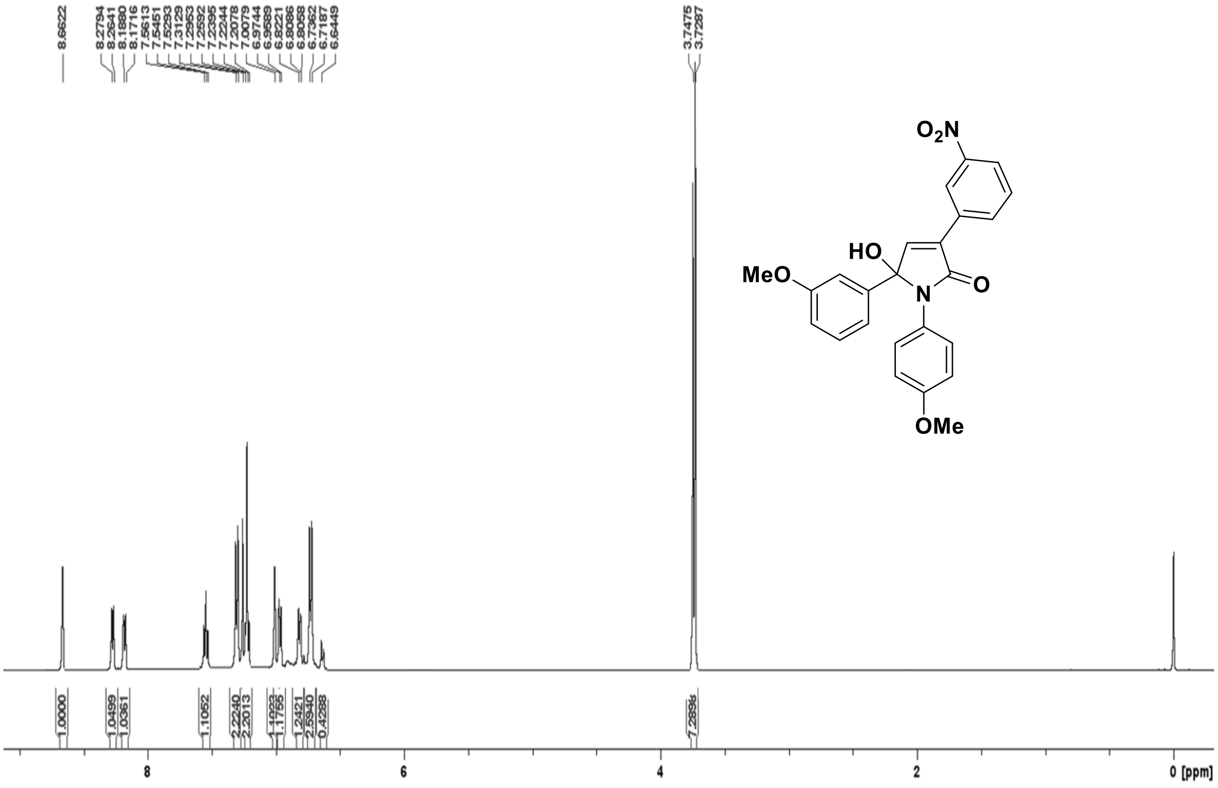

^{13}C-NMR ($CDCl_3$, 125 MHz) of compound 38.

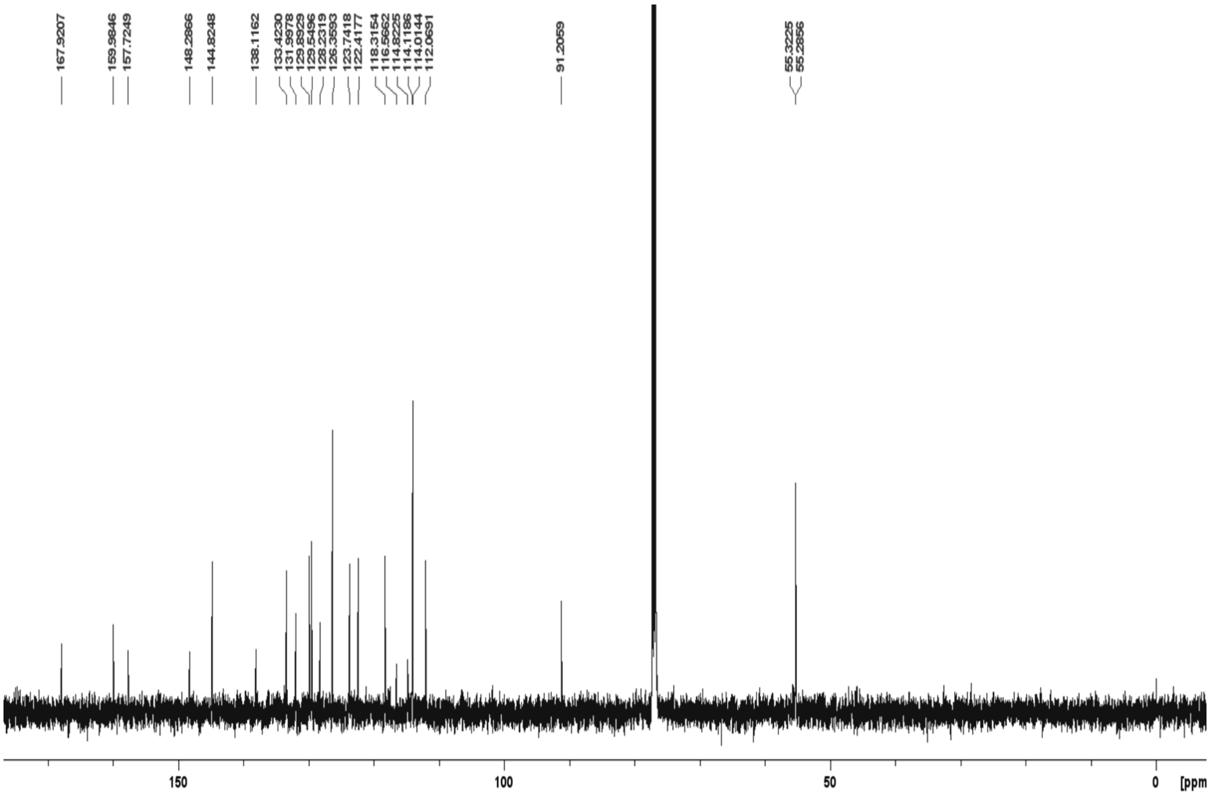

^{1}H-NMR ($CDCl_3$, 500 MHz) of compound 39.

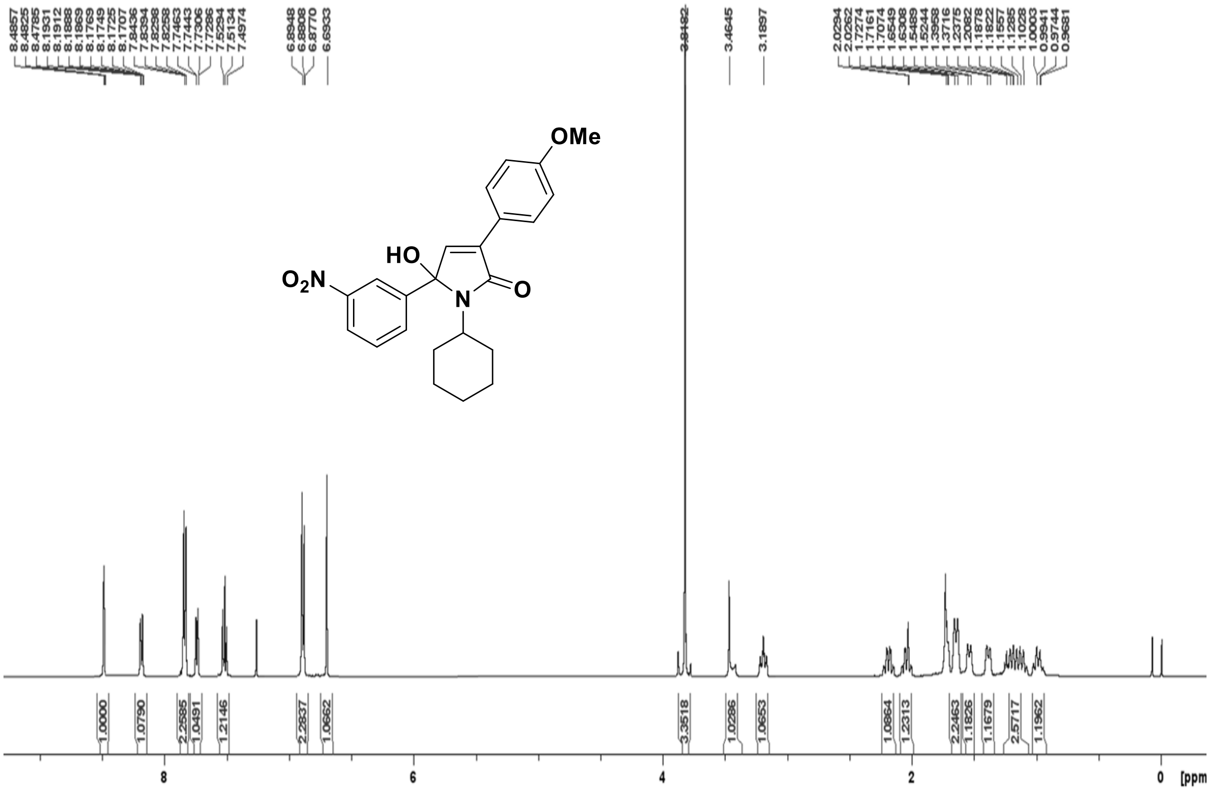

^{13}C-NMR ($CDCl_3$, 125 MHz) of compound 39.

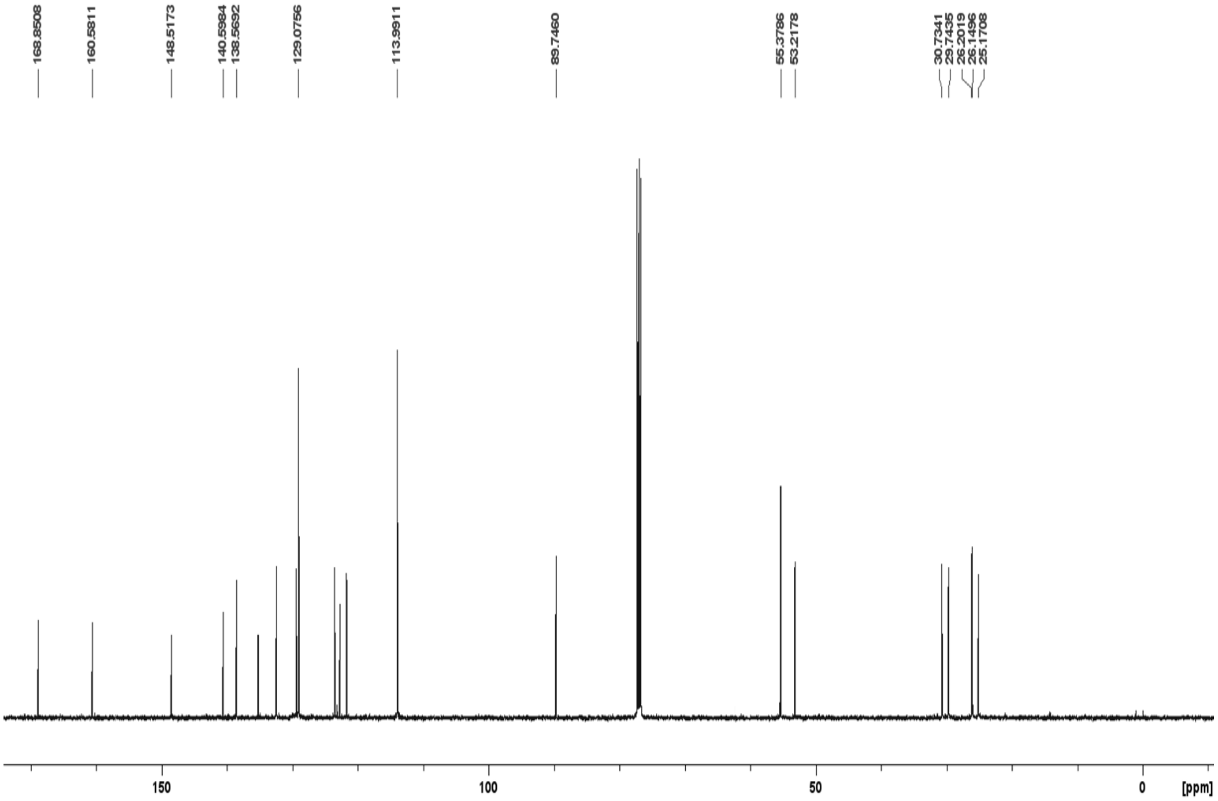

^{1}H-NMR ($CDCl_3$, 500 MHz) of compound 40.

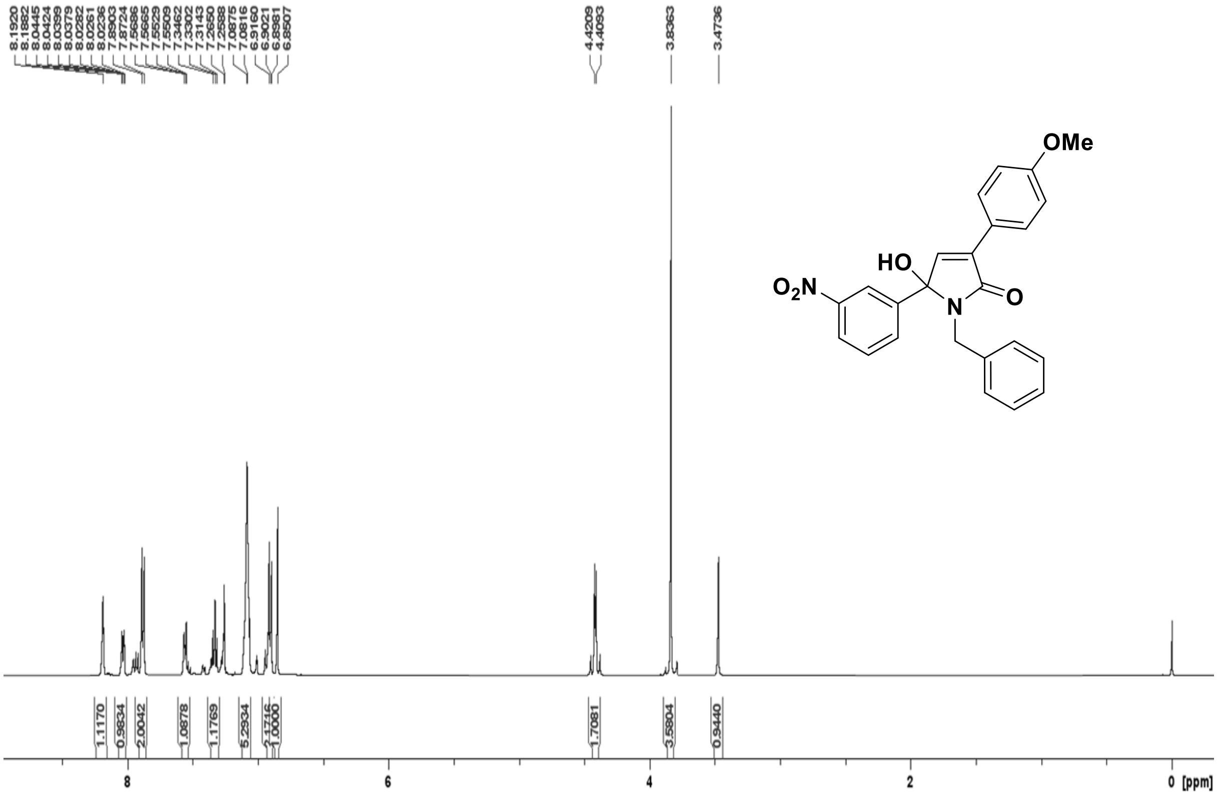

^{13}C-NMR ($CDCl_3$, 125 MHz) of compound 40.

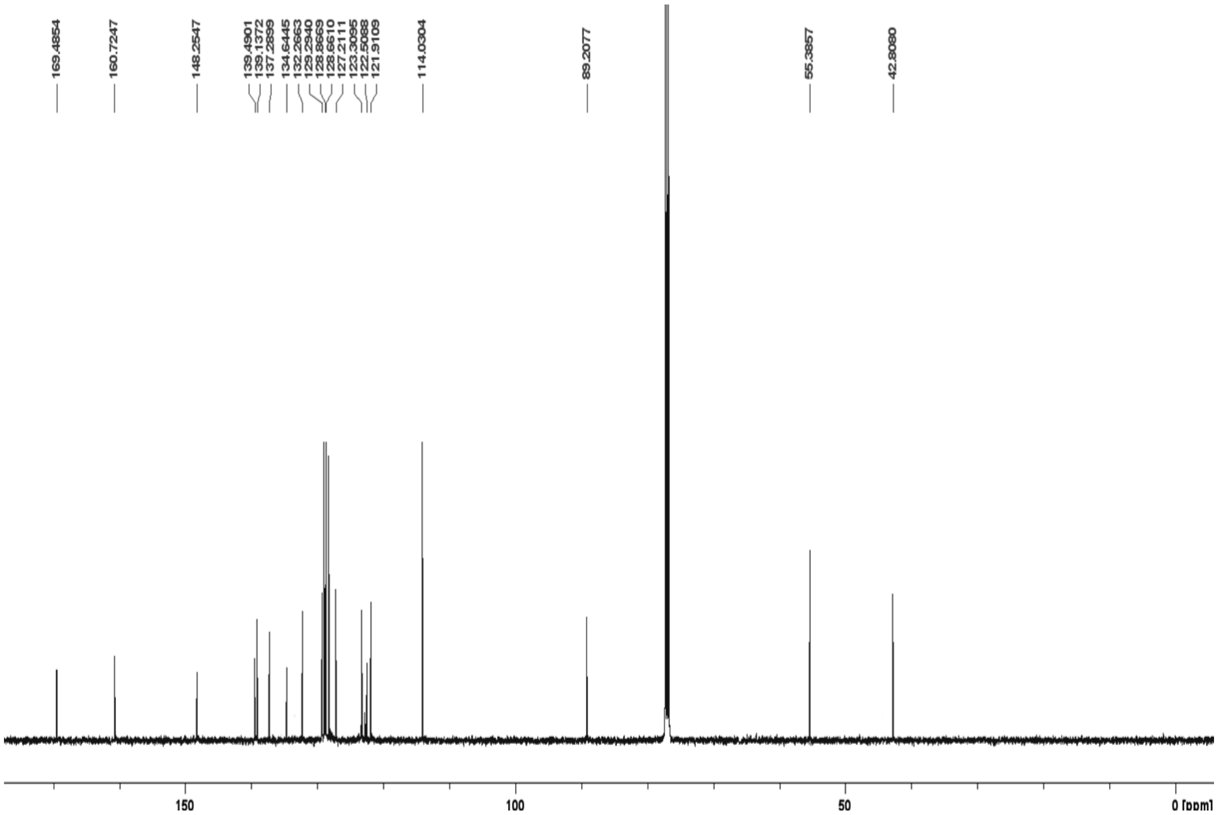

^{1}H-NMR (CDCl$_3$, 500 MHz) of compound 41.

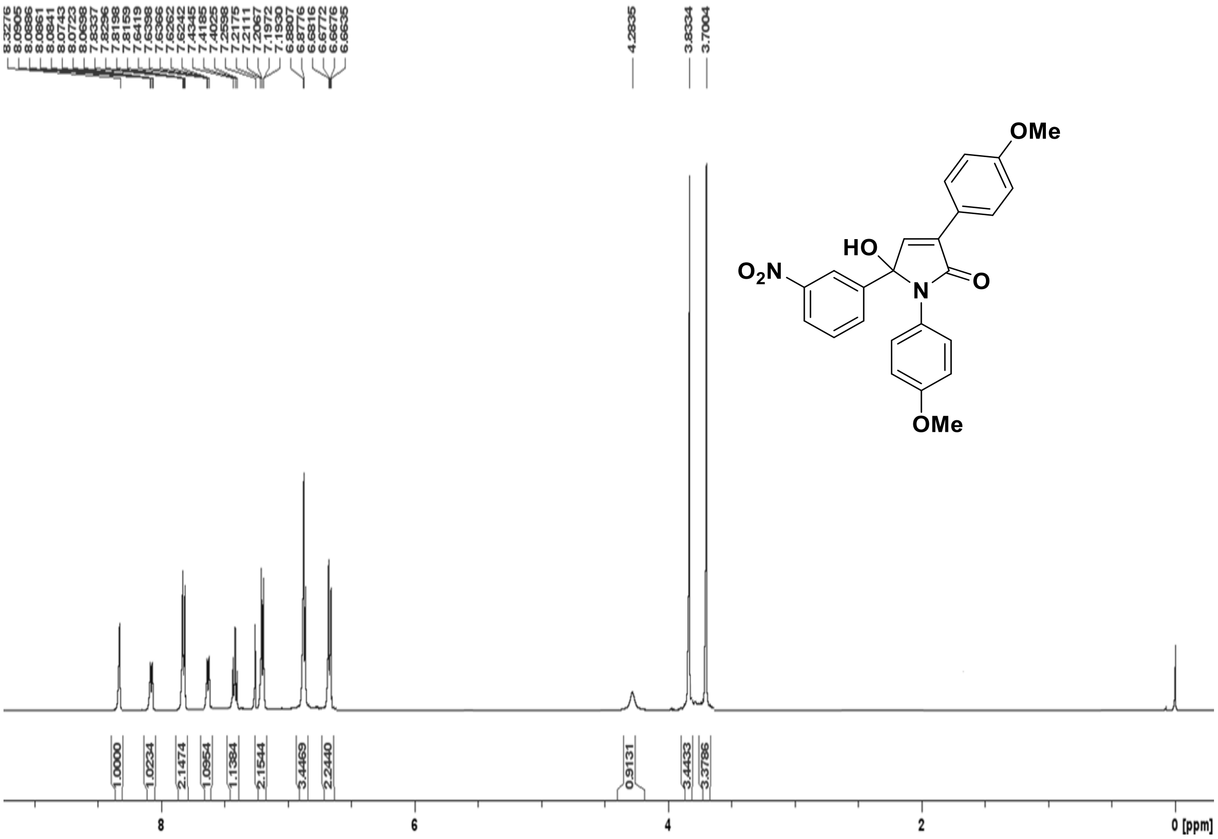

^{13}C-NMR (CDCl$_3$, 125 MHz) of compound 41.

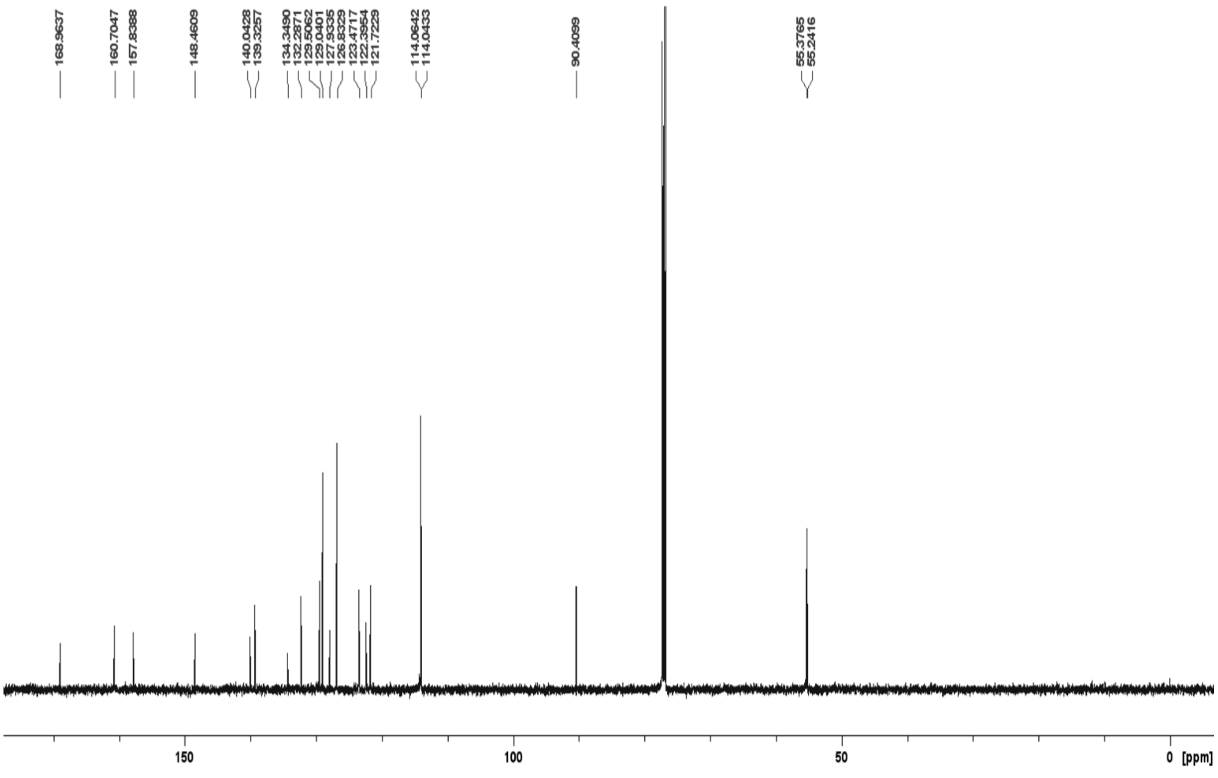

^{1}H-NMR ($CDCl_3$, 500 MHz) of compound 42.

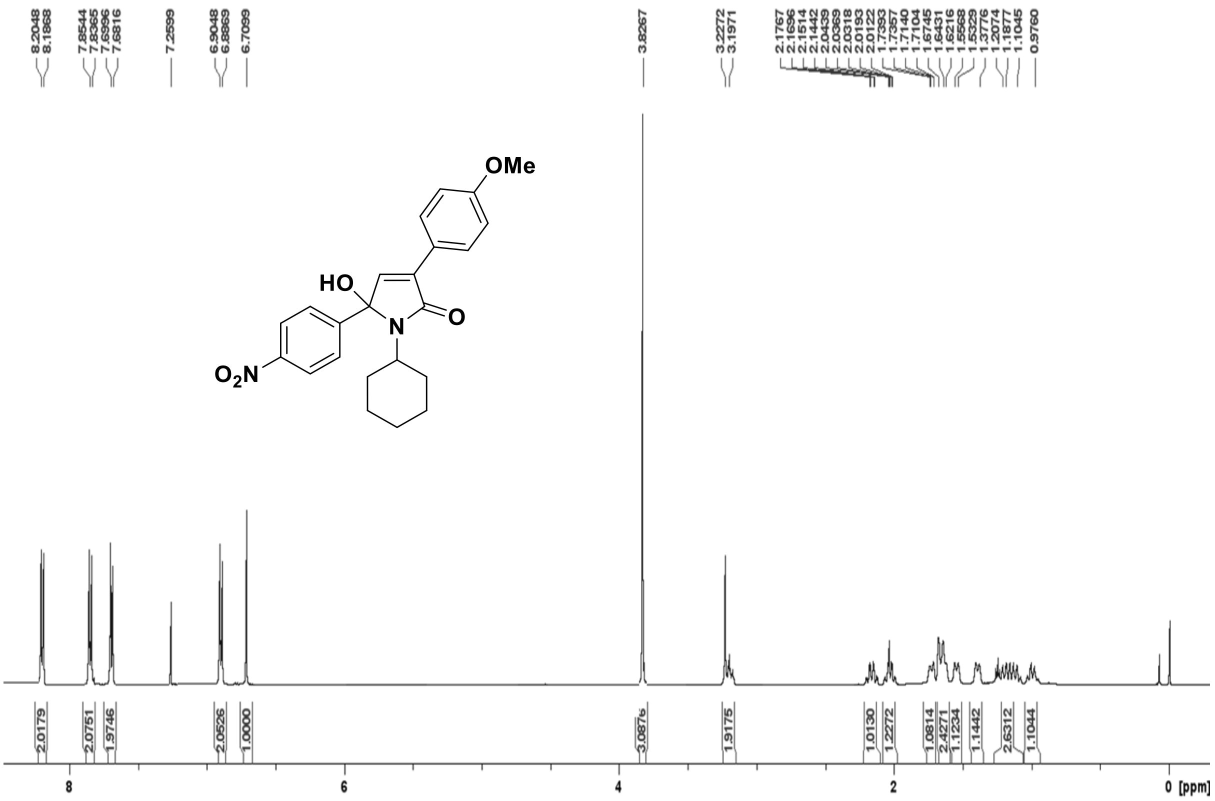

^{13}C-NMR ($CDCl_3$, 125 MHz) of compound 42.

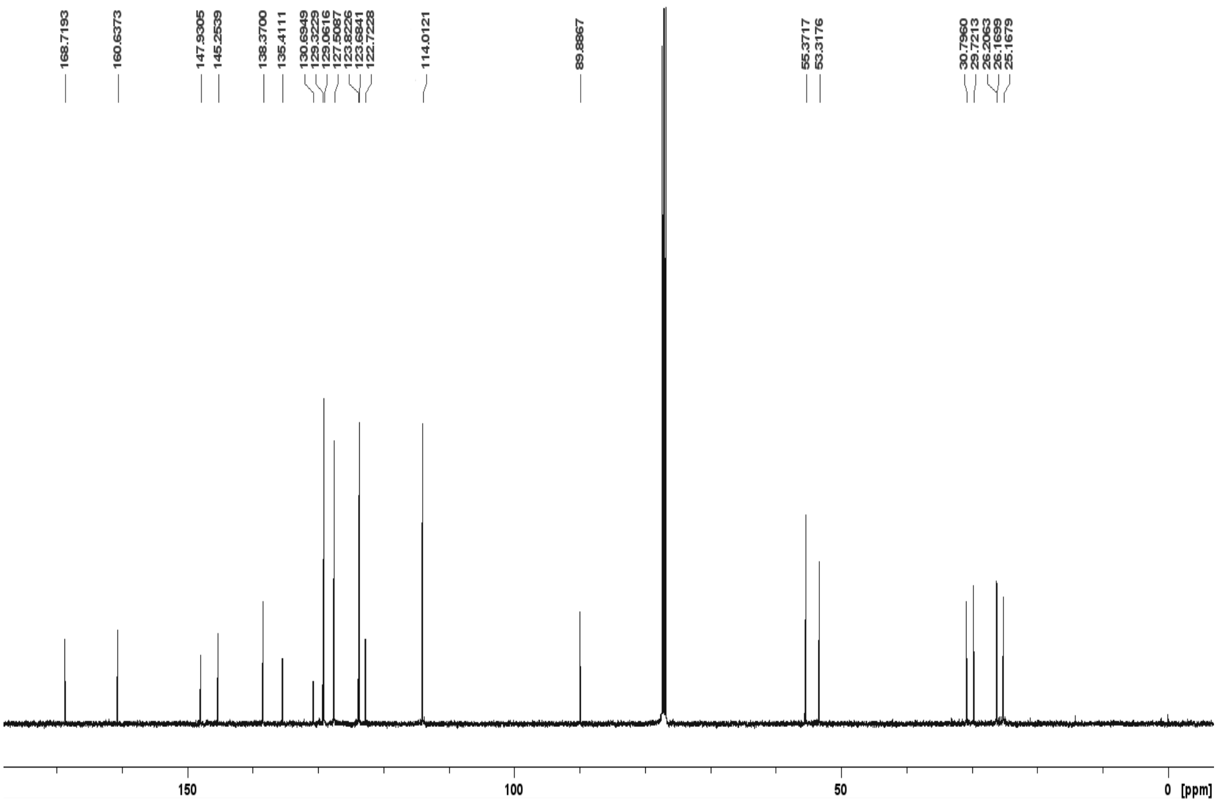

^{1}H-NMR ($CDCl_3$, 500 MHz) of compound 43.

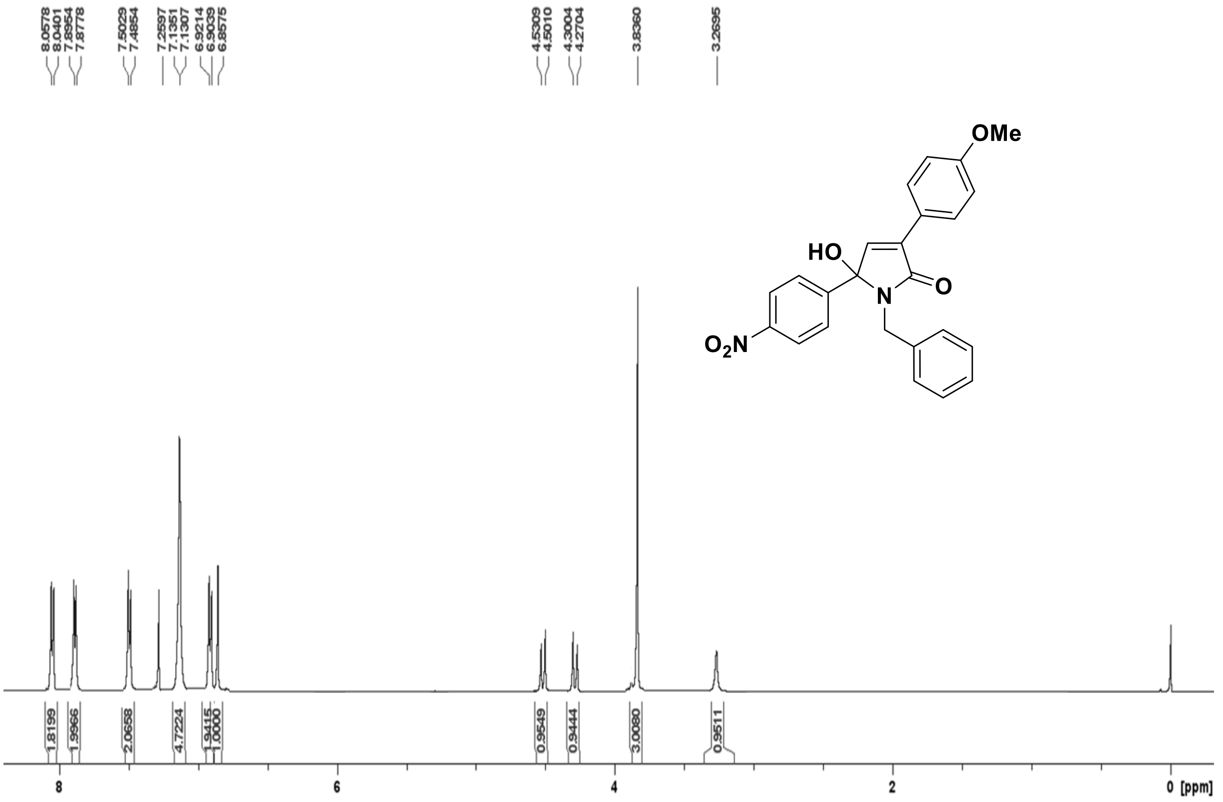

^{13}C-NMR ($CDCl_3$, 125 MHz) of compound 43.

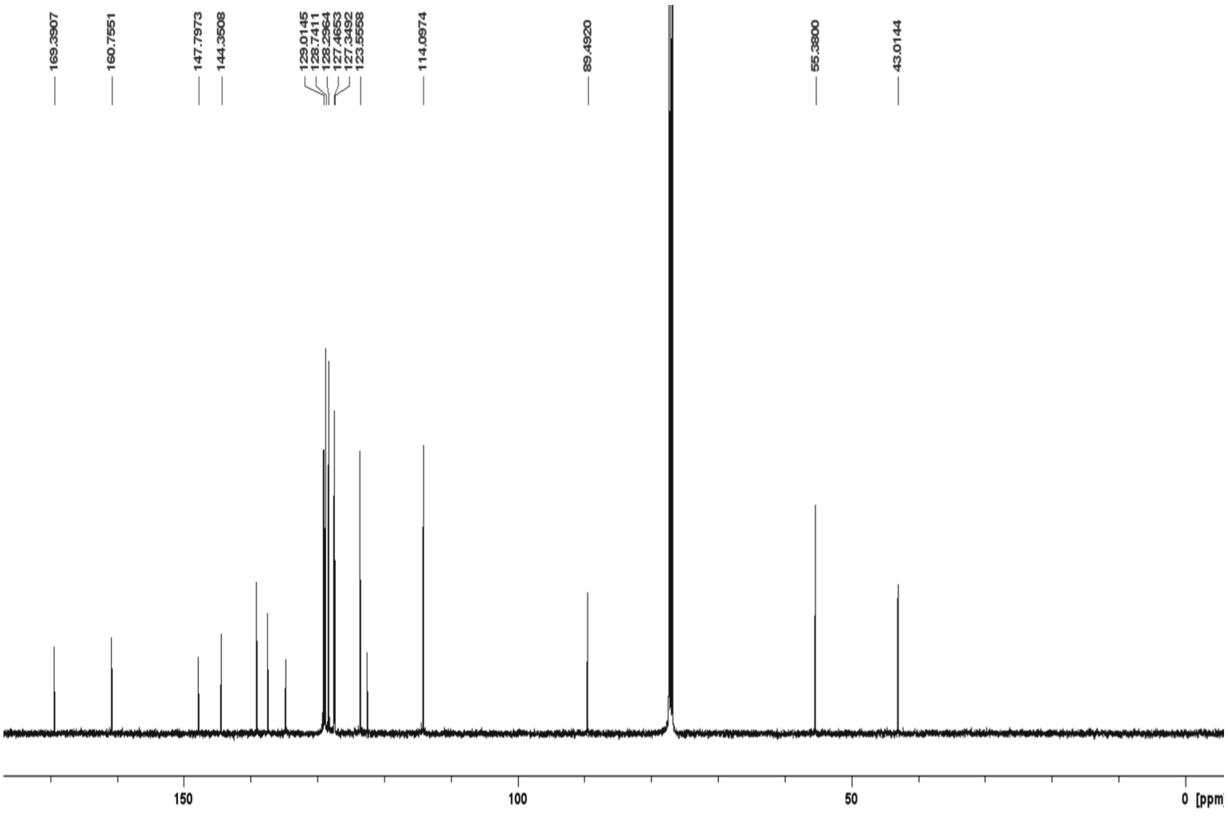

^{1}H-NMR ($CDCl_3$, 500 MHz) of compound 44.

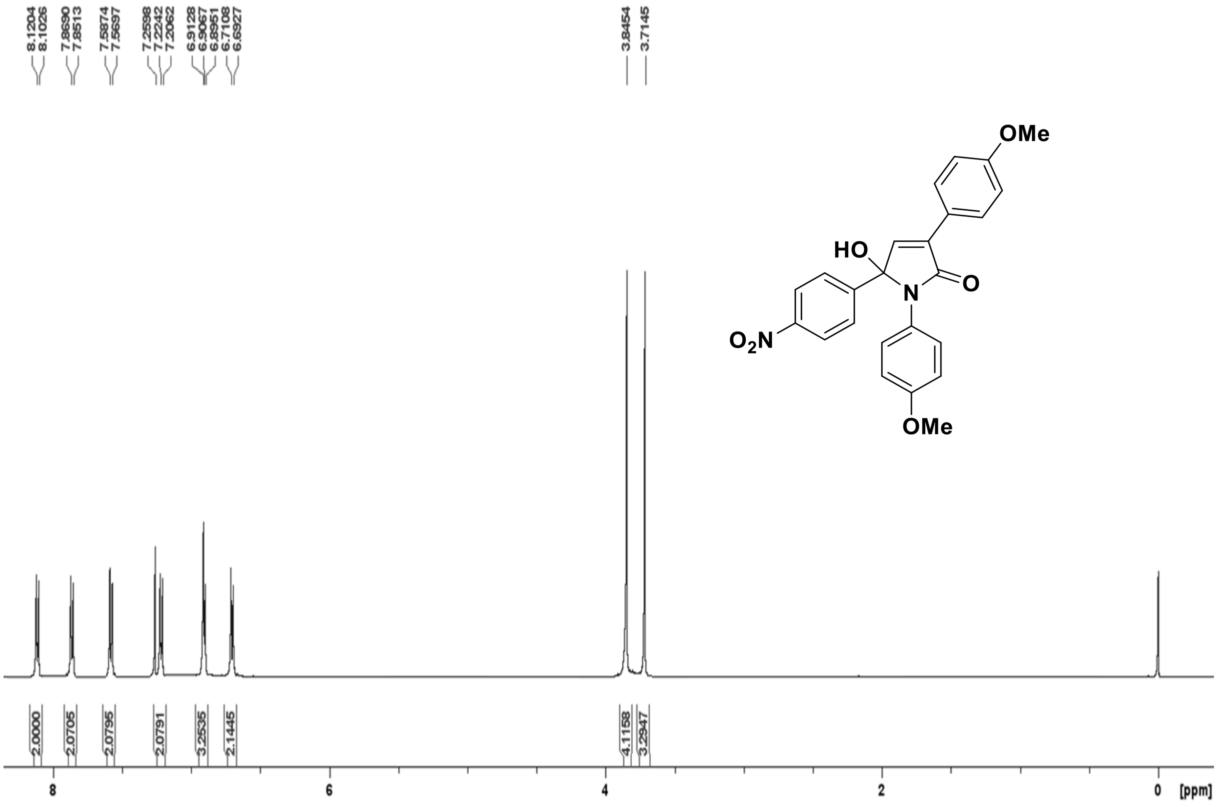

^{13}C-NMR ($CDCl_3$, 125 MHz) of compound 44.

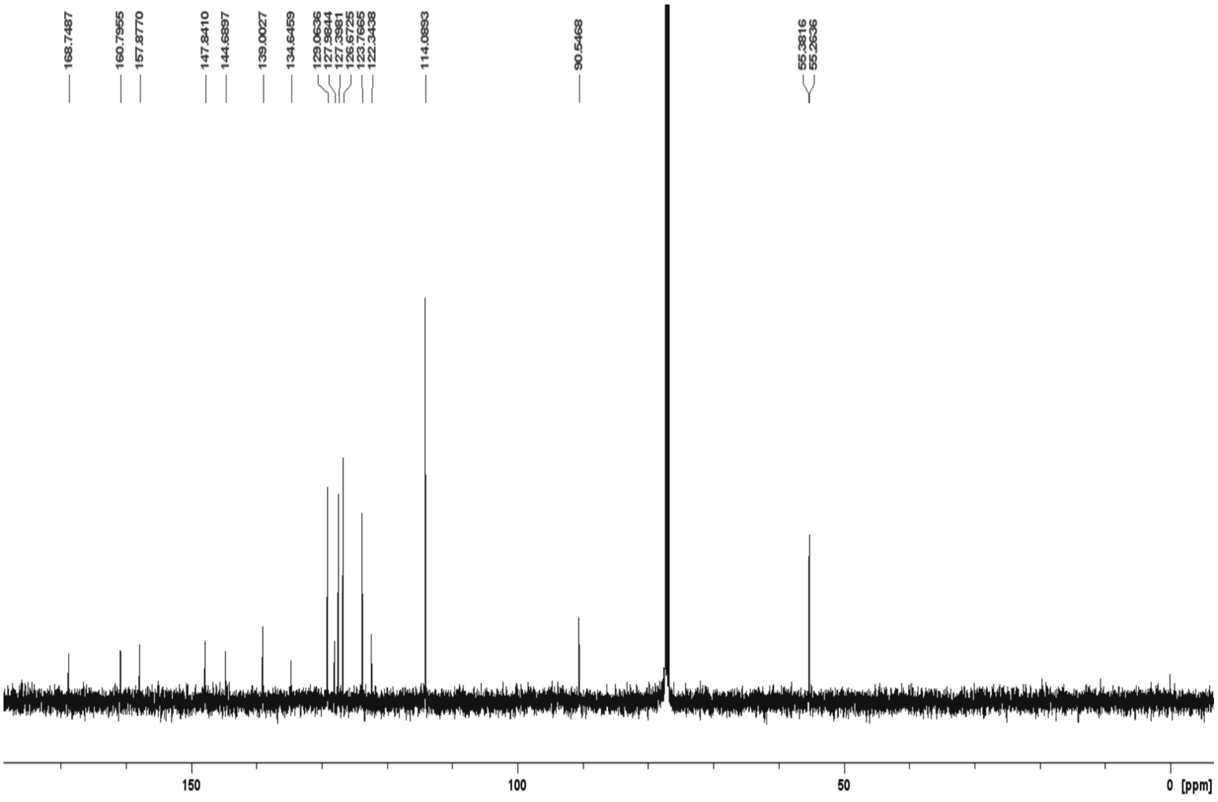

^{1}H-NMR ($CDCl_3$, 500 MHz) of compound 45.

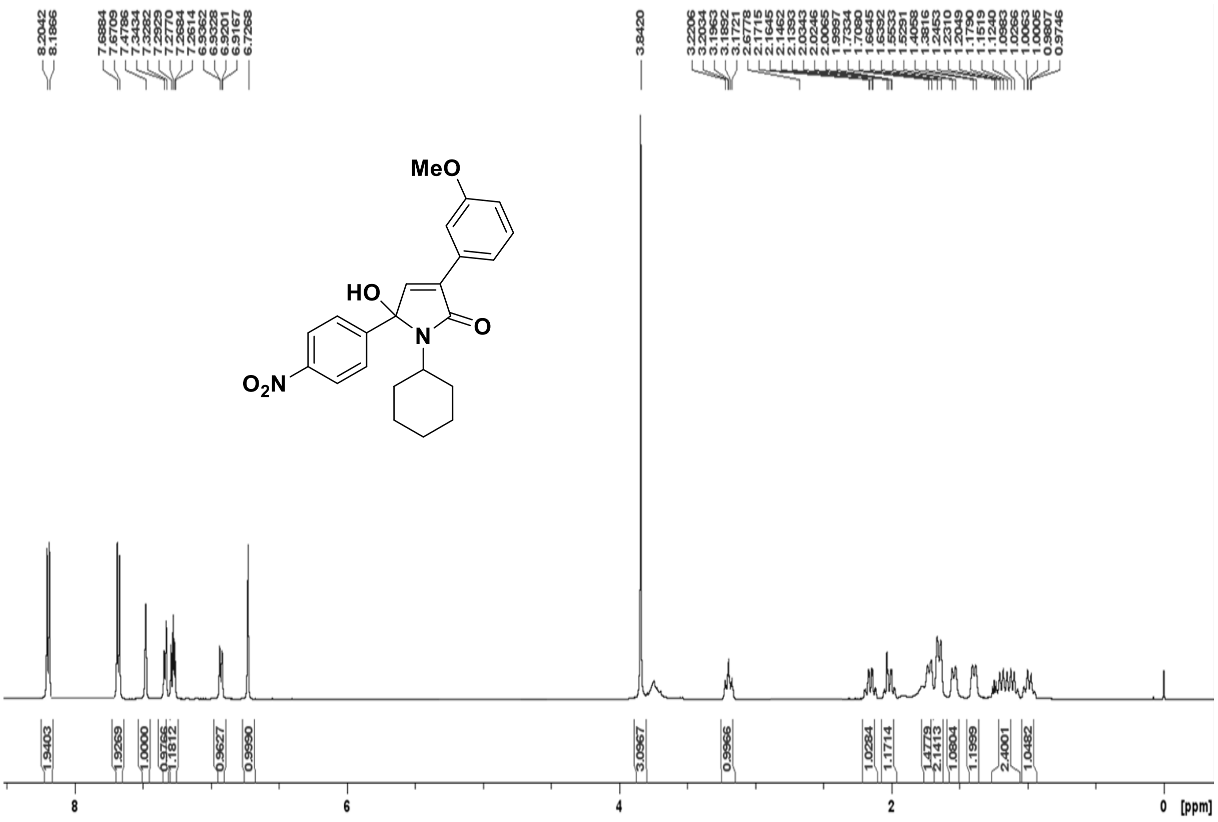

^{13}C-NMR ($CDCl_3$, 125 MHz) of compound 45.

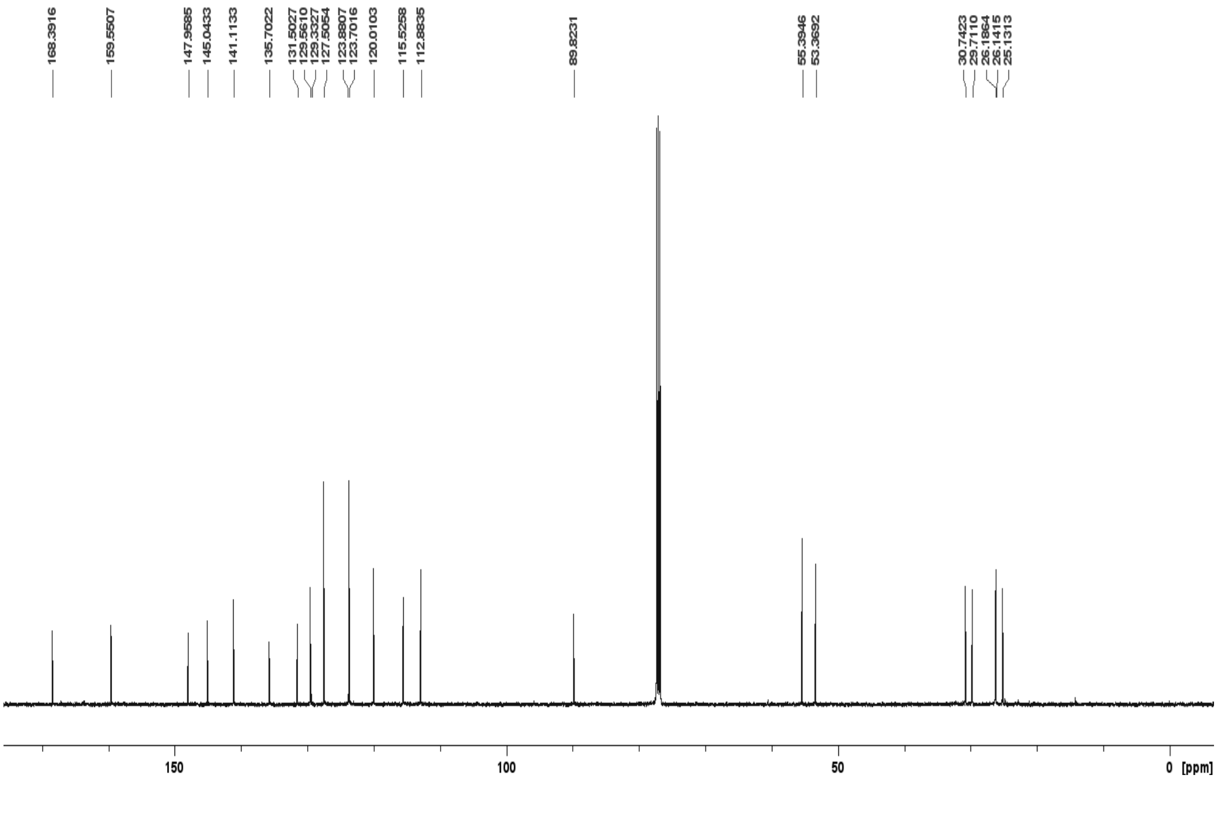

^{1}H-NMR ($CDCl_3$, 500 MHz) of compound 46.

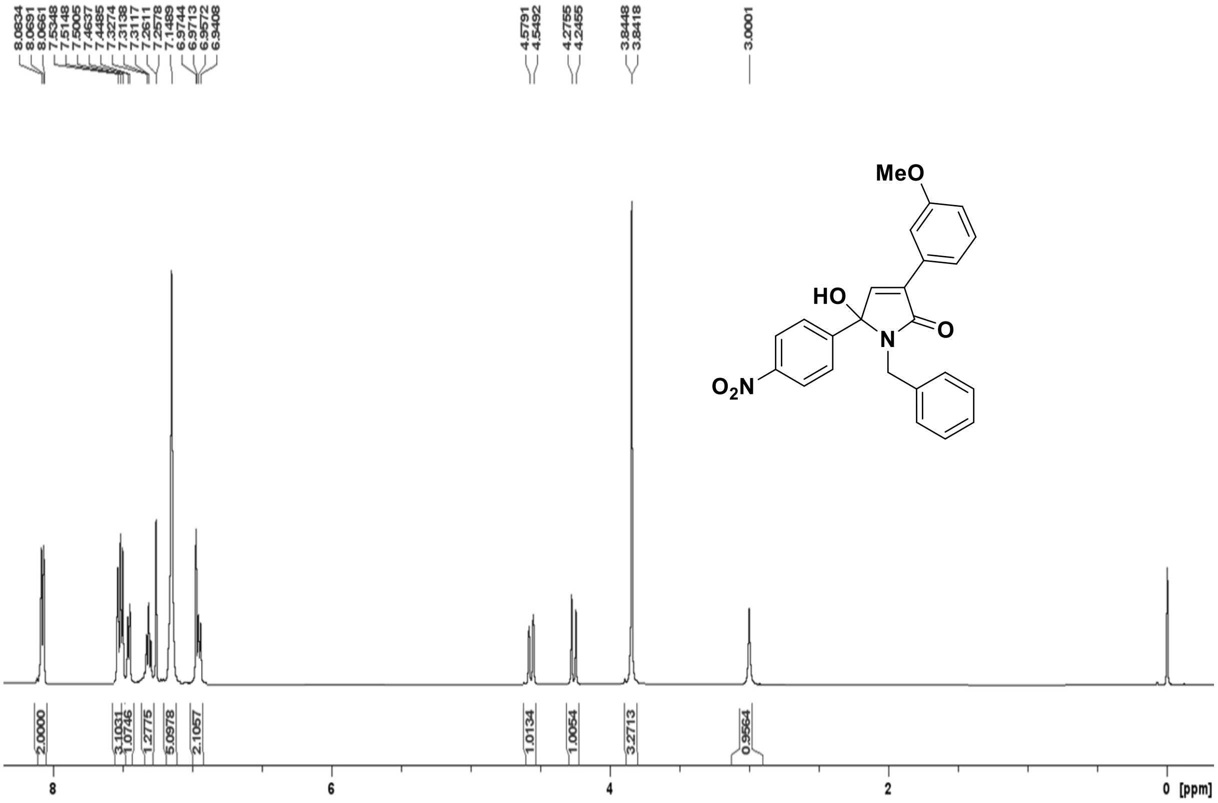

^{13}C-NMR ($CDCl_3$, 125 MHz) of compound 46.

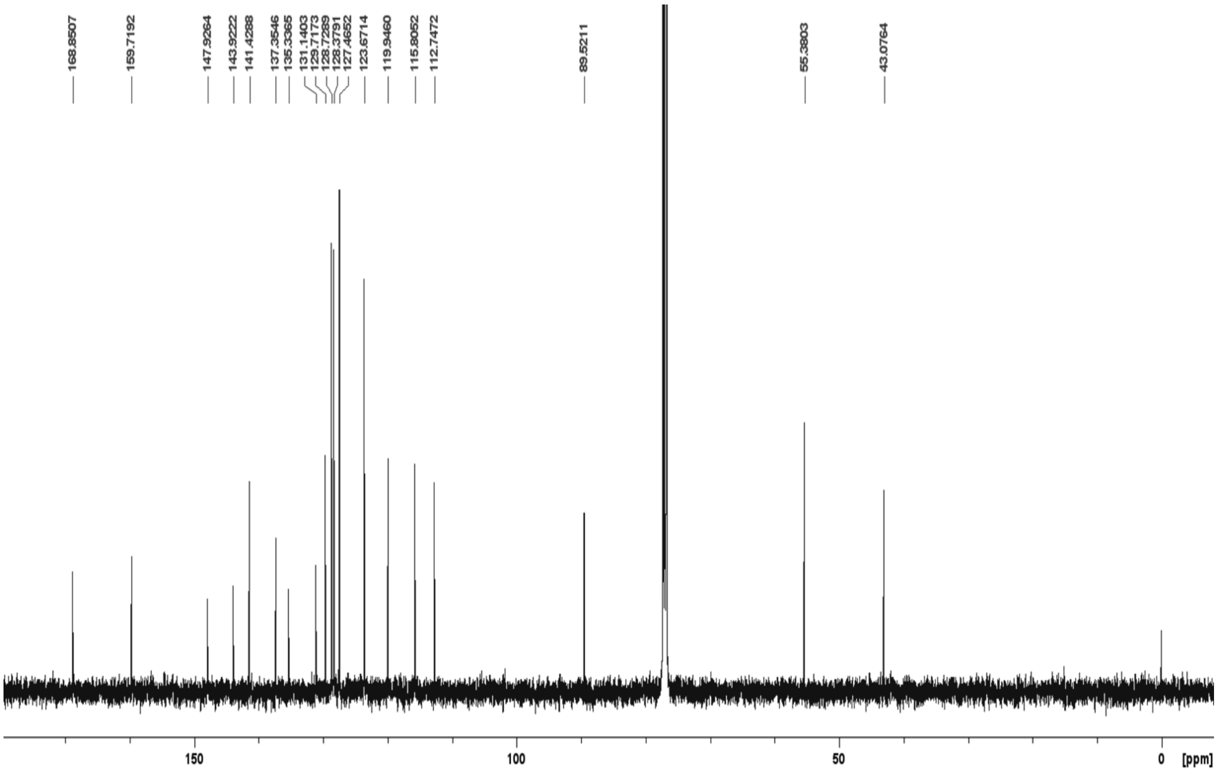

^{1}H-NMR ($CDCl_3$, 500 MHz) of compound 47.

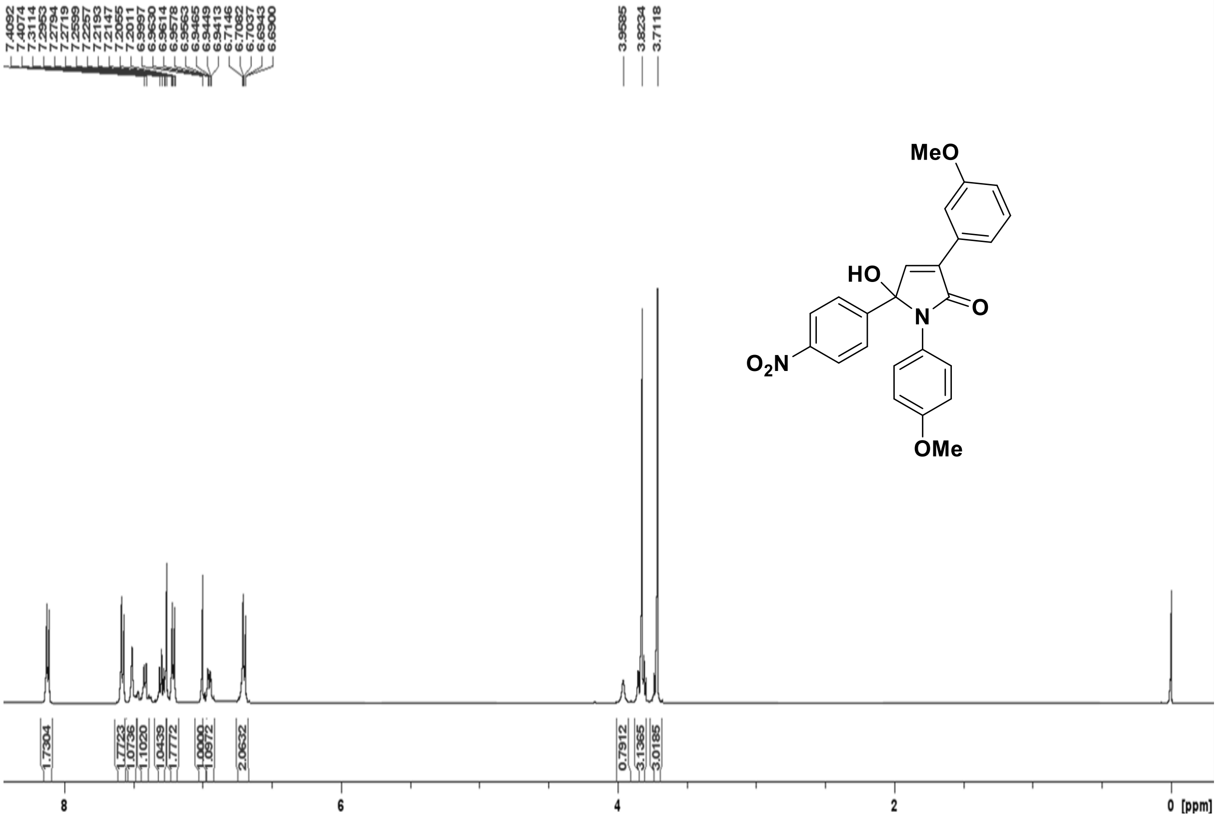

^{13}C-NMR ($CDCl_3$, 125 MHz) of compound 47.

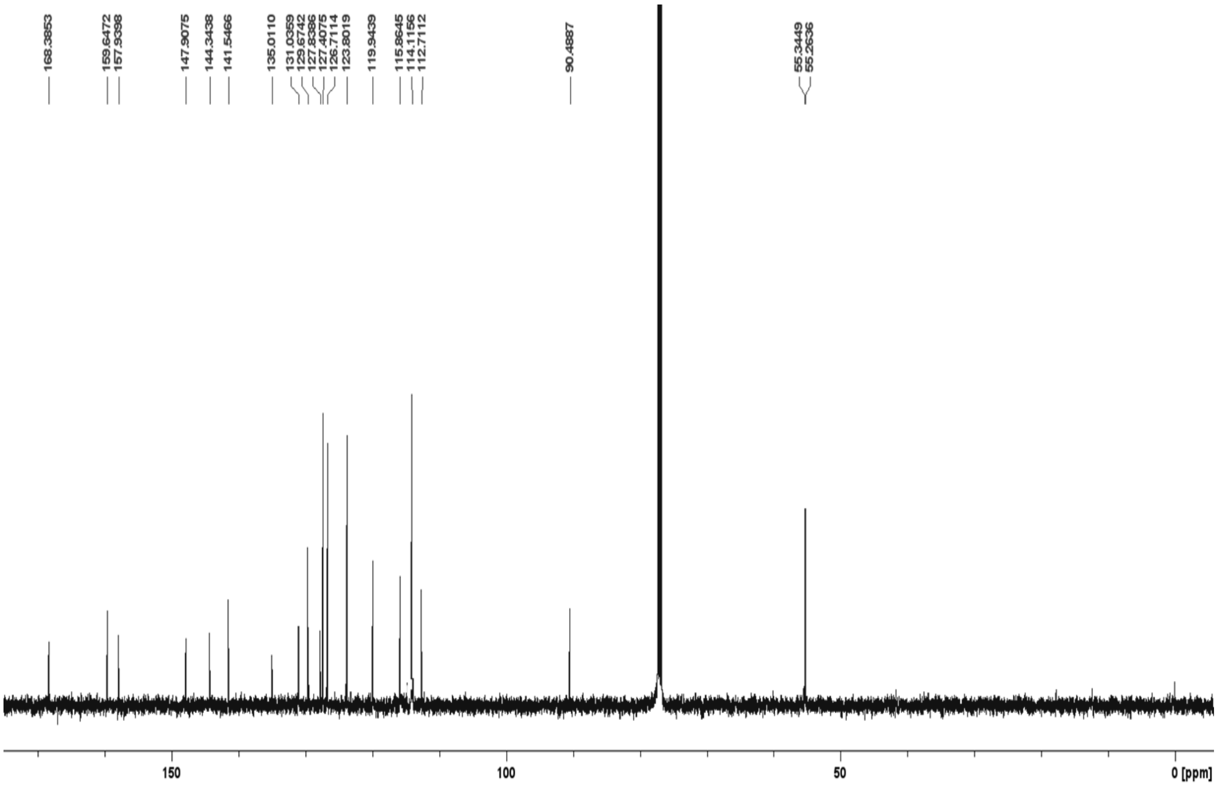

^{1}H-NMR ($CDCl_3$, 500 MHz) of compound 48.

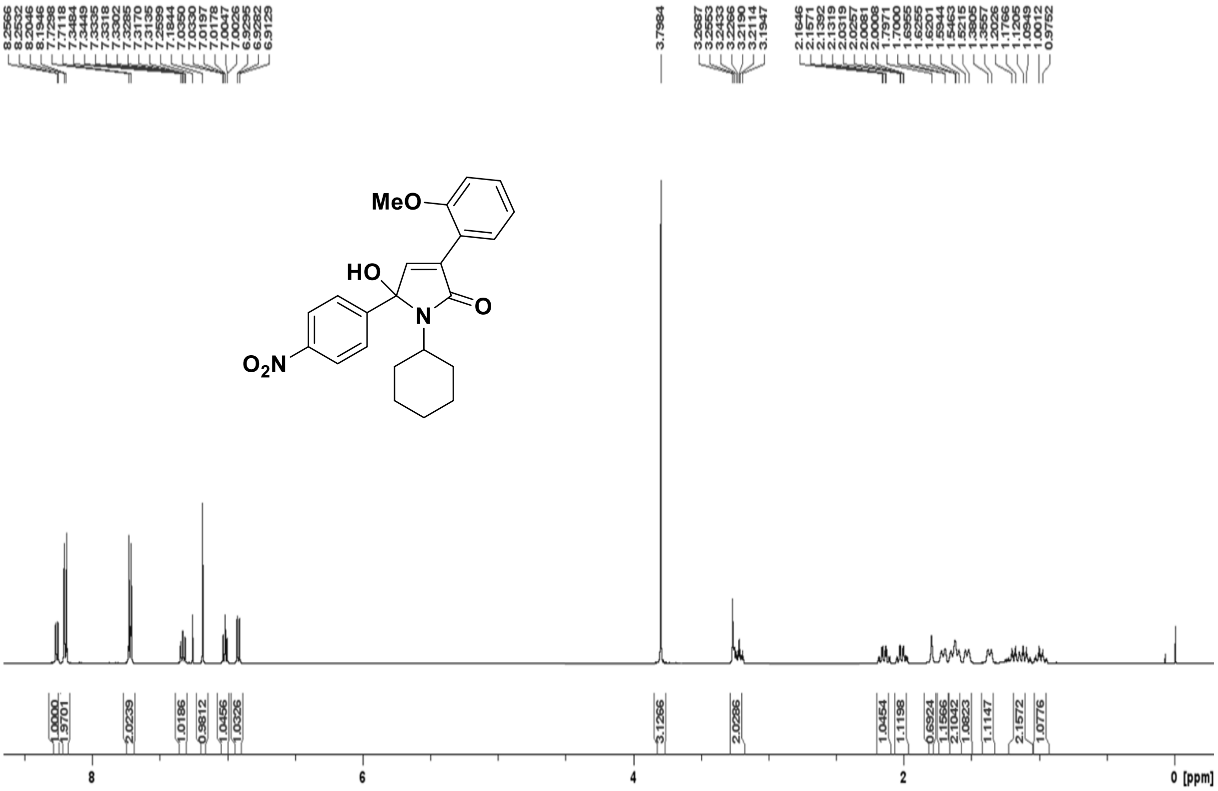

^{13}C-NMR ($CDCl_3$, 125 MHz) of compound 48.

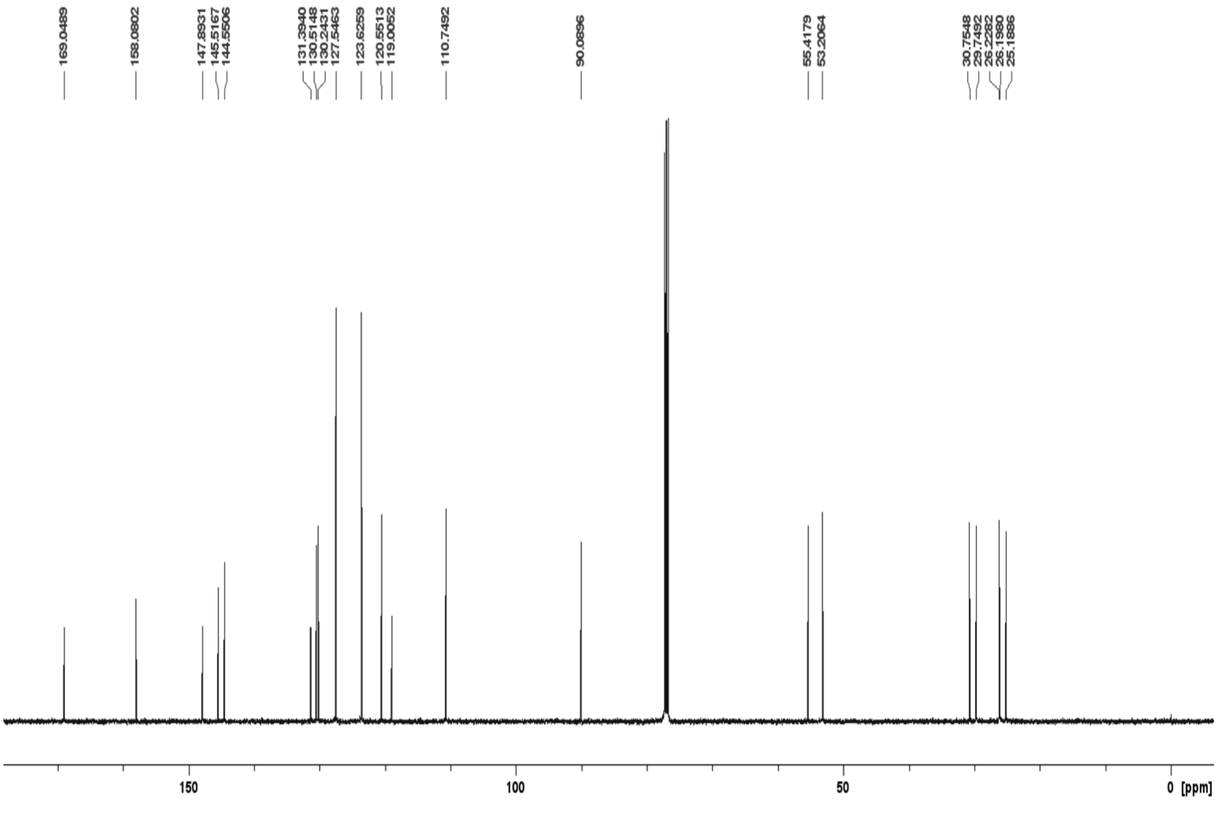

^{1}H-NMR ($CDCl_3$, 500 MHz) of compound 49.

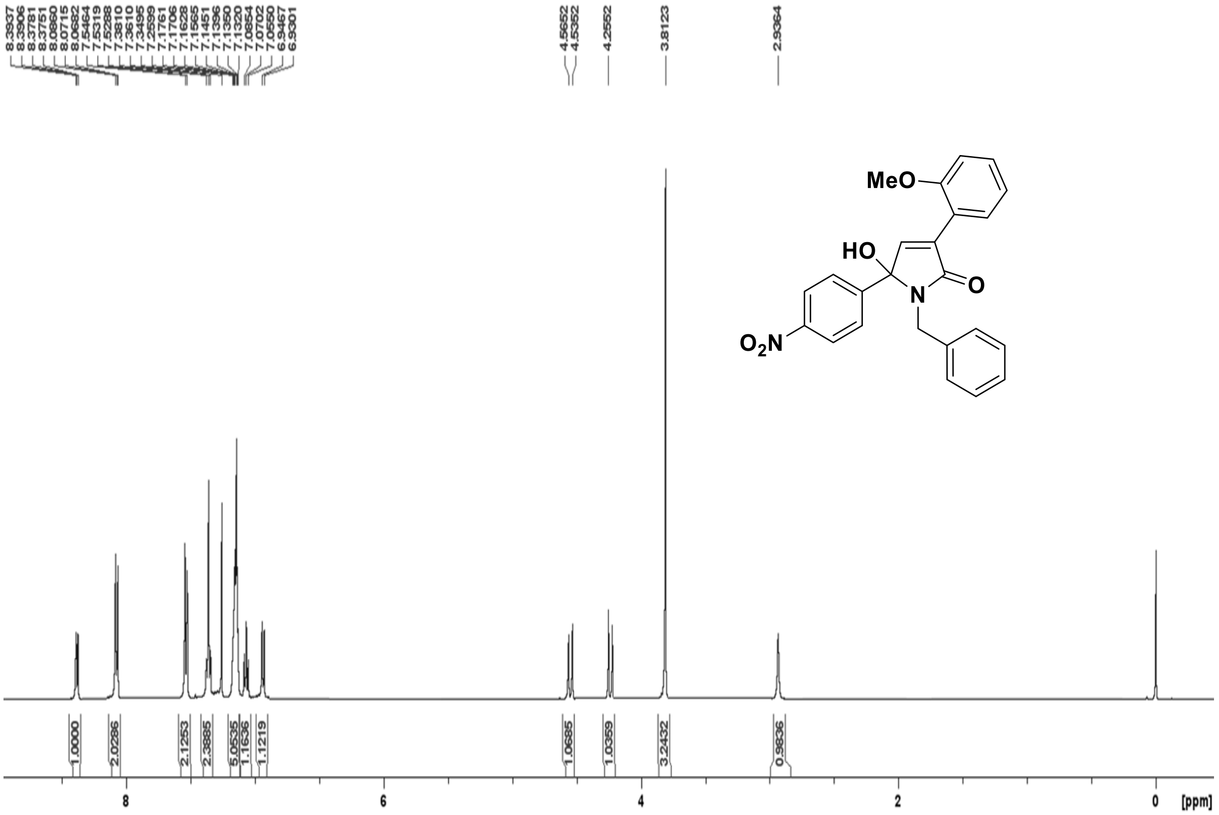

^{13}C-NMR ($CDCl_3$, 125 MHz) of compound 49.

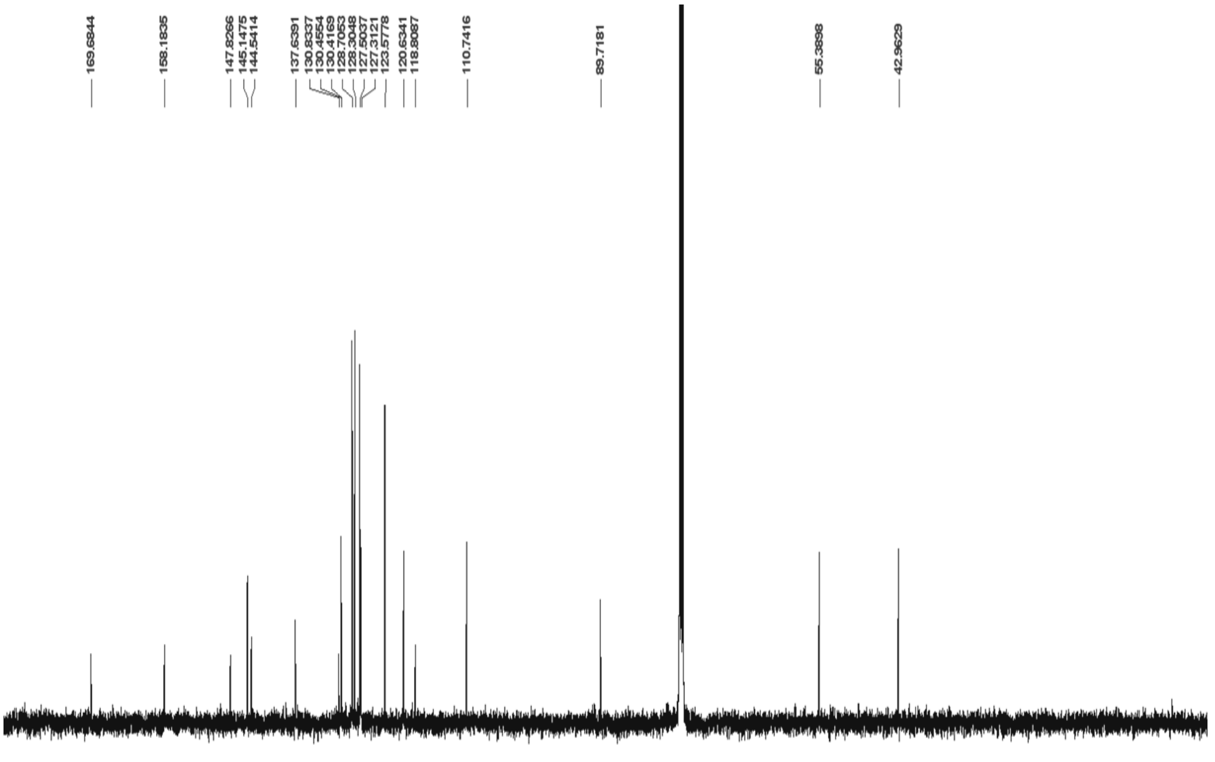

^{1}H-NMR ($CDCl_3$, 500 MHz) of compound 50.

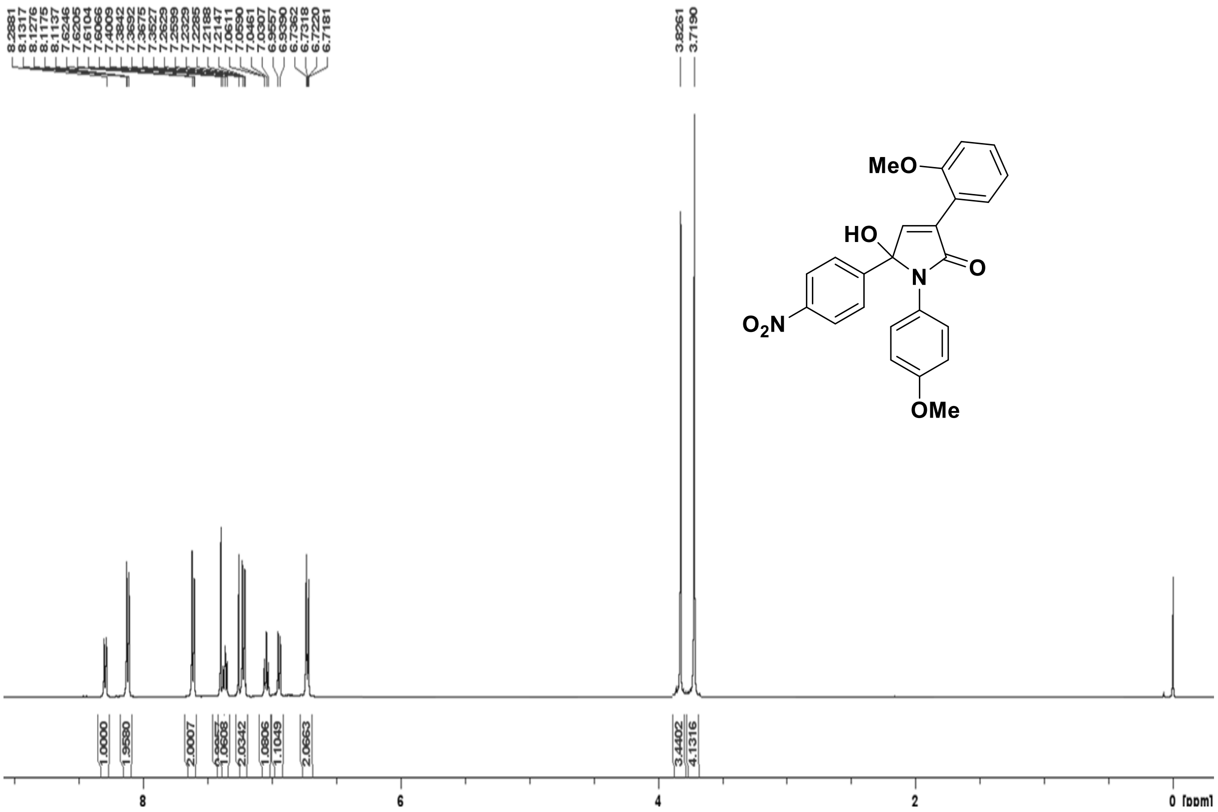

^{13}C-NMR ($CDCl_3$, 125 MHz) of compound 50.

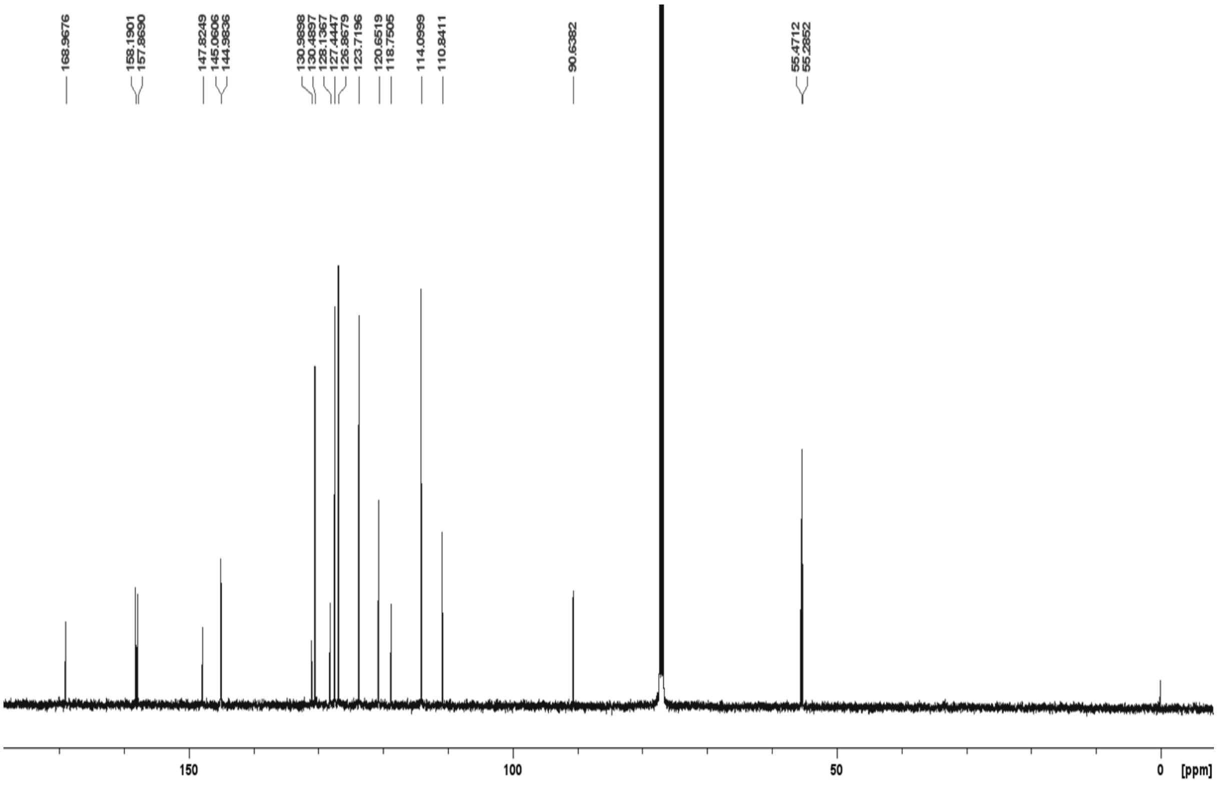

Supplement: Supplementary file 1 [file cancers-14-05174-s001.zip › Figure S1.pdf]
