# Supplementary material for: Discovery of Highly Functionalized 5-hydroxy-2H-pyrrol-2-ones That Exhibit Antiestrogenic Effects in Breast and Endometrial Cancer Cells and Potentiate the Antitumoral Effect of Tamoxifen
Source: Cancers (Basel). 2022 Oct 22;14(21):5174. doi: 10.3390/cancers14215174 (PMC9655618; doi:10.3390/cancers14215174)
Supplement: Supplementary file 1 [file cancers-14-05174-s001.zip › Figure S2.pdf]

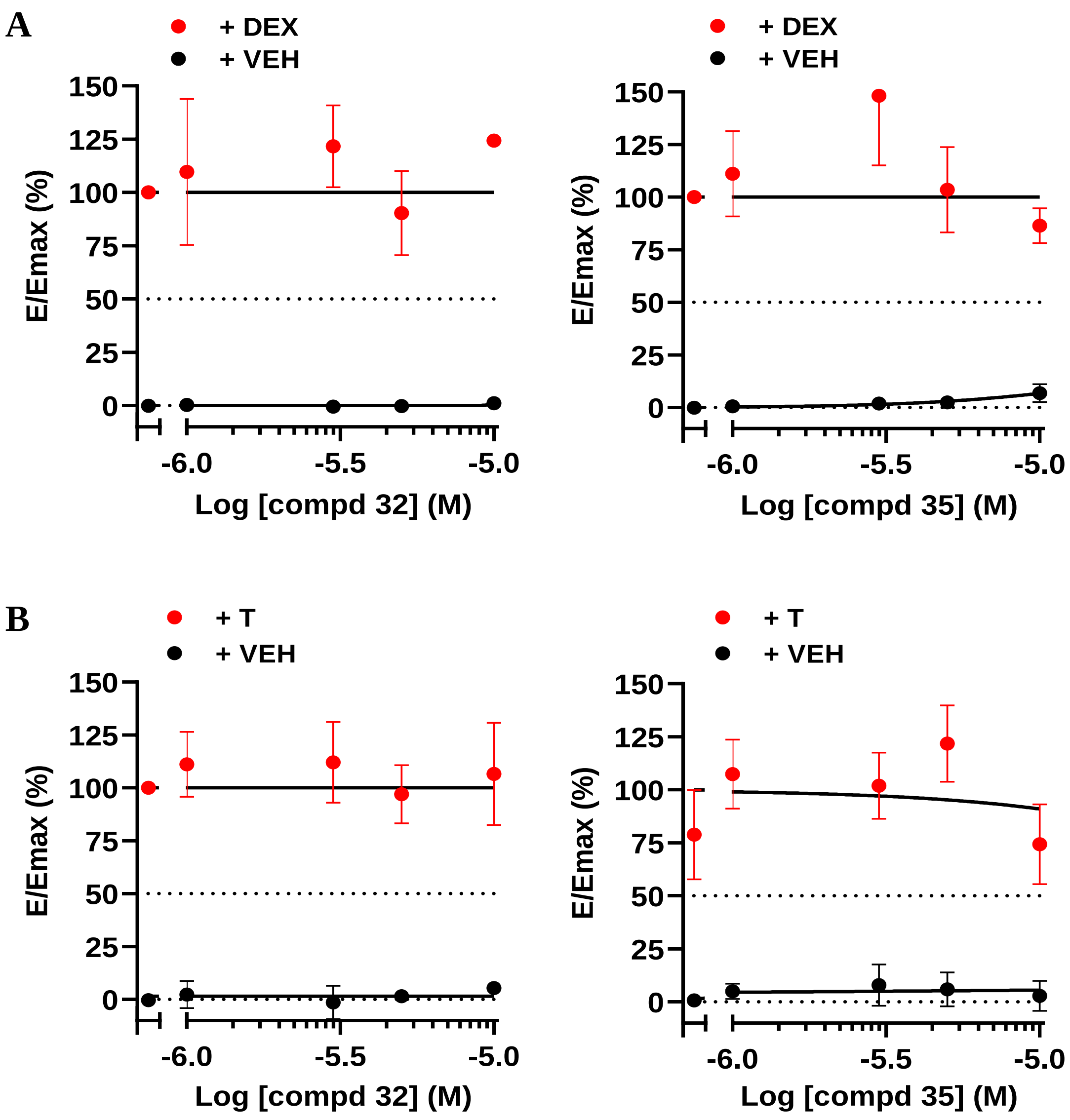

Supplementary Figure S2. Effects of 5-hydroxy-*2H*-pyrrol-2-one compounds 32 and 35 on Androgen (AR) and Glucocorticoid (GR) Receptors-mediated transcription. MDA-Kb2 cells were pretreated with increasing concentrations of compounds **32** and **35** (1 µM–10 µM) for 3 h, before the addition of vehicle (VEH; 0.05% DMSO, ●), **(A)** dexamethasone (DEX, 100 nM), ●) or **(B)** testosterone (T, 100 nM, ●) for 16-24 h. Then, Relative Luciferase Activity (RLU), was analyzed as described in Material and Methods. The maximal luciferase activity or Emax was induced by DEX (42.07 ± 14.33-fold induction) or T (6.81 ± 1.83-fold induction) and the efficacy (E) of each respective treatment, as compared with Emax, was calculated (E/Emax %). Non-linear regression analysis was applied with GraphPad Prism software 8.4.3 to calculate the IC_{50} values. Data are expressed as mean ± SEM for at least three independent experiments, where each treatment was tested in triplicate.
