# Supplementary material for: Discovery of Highly Functionalized 5-hydroxy-2H-pyrrol-2-ones That Exhibit Antiestrogenic Effects in Breast and Endometrial Cancer Cells and Potentiate the Antitumoral Effect of Tamoxifen
Source: Cancers (Basel). 2022 Oct 22;14(21):5174. doi: 10.3390/cancers14215174 (PMC9655618; doi:10.3390/cancers14215174)
Supplement: Supplementary file 1 [file cancers-14-05174-s001.zip › Figure S3.pdf]

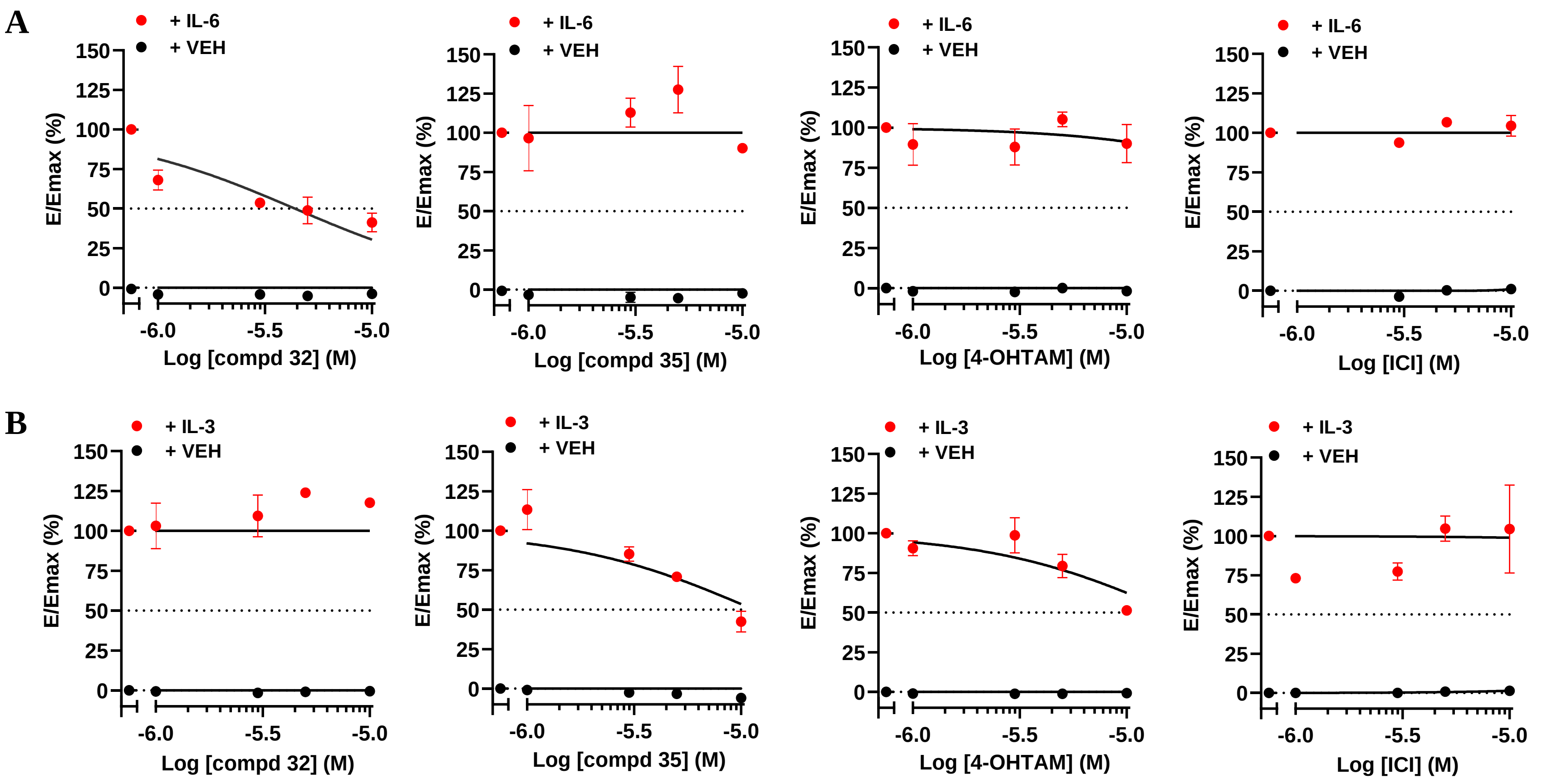


**Supplementary Figure S3. Effects of 5-hydroxy-*2H*-pyrrol-2-one compounds 32 and 35 on STAT3 and STAT5-mediated transcription.** (**A**) HEK293 cells
were pretreated with increasing concentrations of compounds 32 and 35 (1 µM–10 µM) for 4 h, before the addition of vehicle (VEH; 0.05% DMSO, ●) or hIL6 (10 ng/ml, ●) for 16 h. (**B**) Ba/F3 cells were pretreated with increasing concentrations of compounds **32** and **35** (from 1 µM to 10 µM) for 4 h, before the addition of vehicle (VEH; 0.05% DMSO, ●) or mIL3 (30 ng/ml, ●) for 16 h. Relative Luciferase Activity (RLU), was analyzed as described in Material and Methods. The maximal luciferase activity or Emax was induced by hIL-6 (10.08 ± 2.41-fold induction) or mIL-3 (9.31 ± 0.49-fold induction) and the efficacy (E) of each respective treatment, compared with Emax, was calculated (E/Emax %). Non-linear regression analysis was applied with GraphPad Prism software 8.4.3 to calculate the $IC_{50}$ values in each case. Data are expressed as mean ± SEM for at least three independent experiments, where each treatment was tested in triplicate.
