# Supplementary material for: Discovery of Highly Functionalized 5-hydroxy-2H-pyrrol-2-ones That Exhibit Antiestrogenic Effects in Breast and Endometrial Cancer Cells and Potentiate the Antitumoral Effect of Tamoxifen
Source: Cancers (Basel). 2022 Oct 22;14(21):5174. doi: 10.3390/cancers14215174 (PMC9655618; doi:10.3390/cancers14215174)
Supplement: Supplementary file 1 [file cancers-14-05174-s001.zip › Figure S4.pdf]

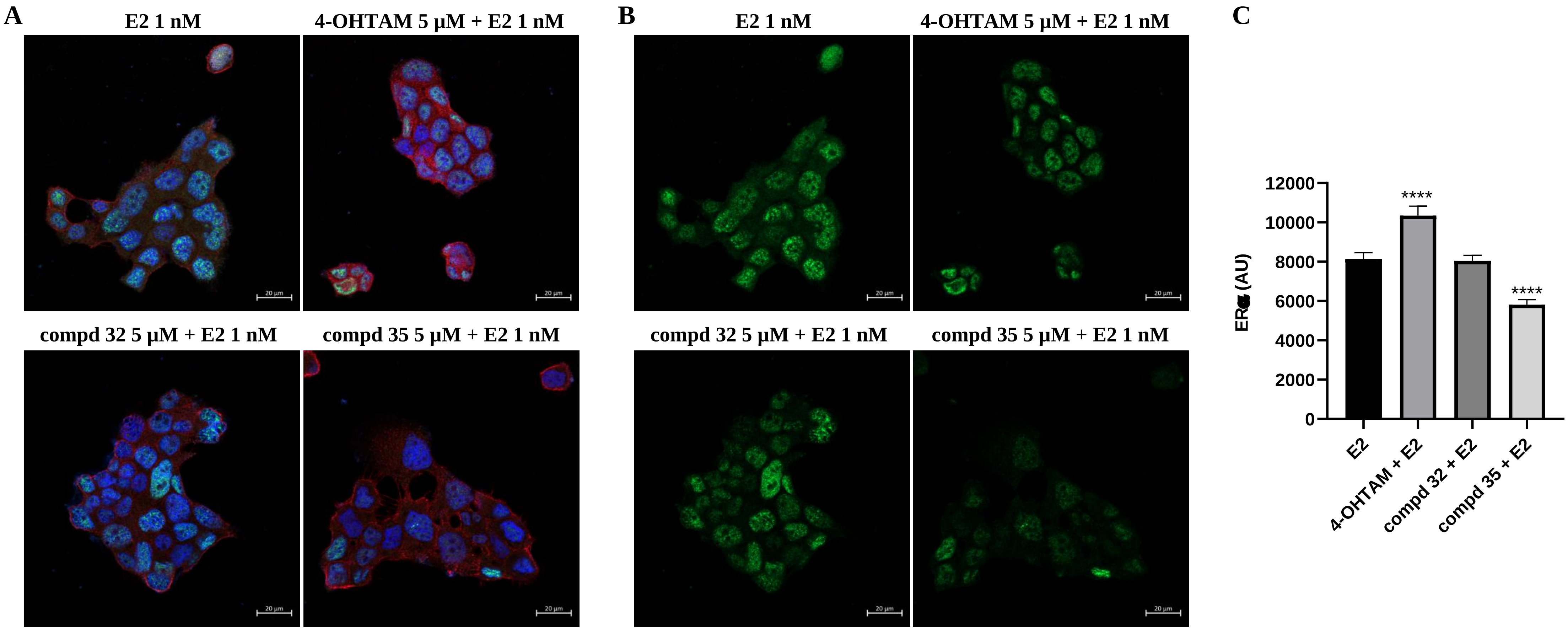

Supplementary Figure S4. Effects of 5-hydroxy-*2H*-pyrrol-2-one compounds 32 and 35 on ERα protein levels and location studied by immunofluorescence assays. E2-depleted T47D cells were pretreated with vehicle (VEH; 0.05% DMSO), 4-OHTAM (5 µM), compounds **32** or **35** (both at 5 µM) for 24 h, before addition of E2 (1 nM, ●) for 30 minutes. Then, cells were fixed, permeabilized and incubated with specific antibodies directed against ERα (ERα-F10; green staining), β-catenin (membrane marker; red staining) and DAPI (DNA marker; blue nuclear staining). (**A**) Representative images of each treatment captured with a Zeiss LSM 800+ confocal microscope with the three fluorescence channels merged, as described in Material and Methods. **(B)** The same representative images of panel A captured with the green fluorescence channel. **(C)** Quantification of the green fluorescence values (in arbitrary units, AU) obtained for the expression of nuclear ERα in each treatment condition. Statistical significance was assessed by the means comparisons of each treatment condition using a two-tailed T-Student. ****$P < 0.0001$ *vs* E2-treated cells ERα protein expression levels.
