# Supplementary material for: Discovery of Highly Functionalized 5-hydroxy-2H-pyrrol-2-ones That Exhibit Antiestrogenic Effects in Breast and Endometrial Cancer Cells and Potentiate the Antitumoral Effect of Tamoxifen
Source: Cancers (Basel). 2022 Oct 22;14(21):5174. doi: 10.3390/cancers14215174 (PMC9655618; doi:10.3390/cancers14215174)
Supplement: Supplementary file 1 [file cancers-14-05174-s001.zip › Figure S5.pdf]

Figure S5: Full uncropped immunoblot images.

Corresponding to Figure 14A (24h)

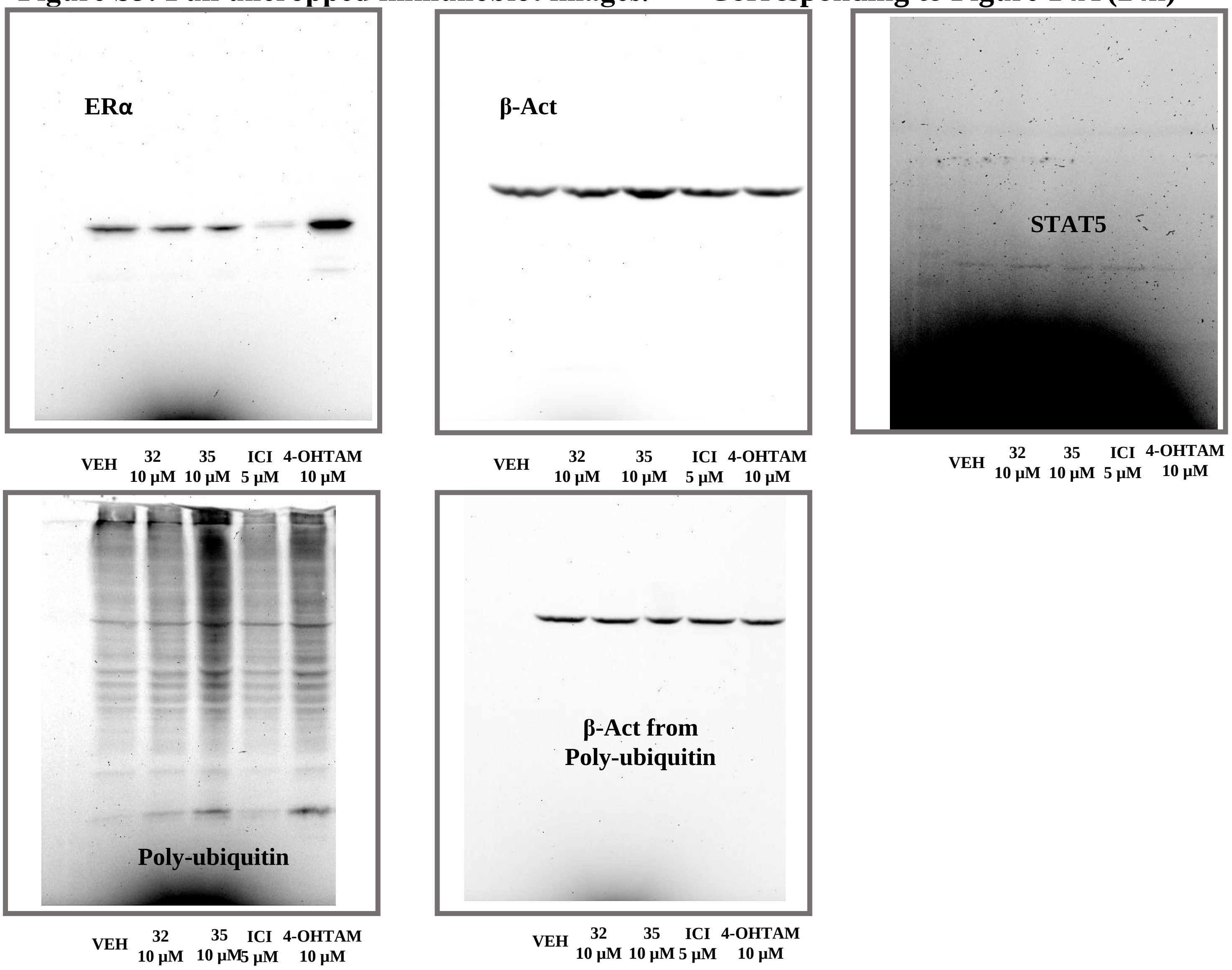

Corresponding to Figure 14A (48h)

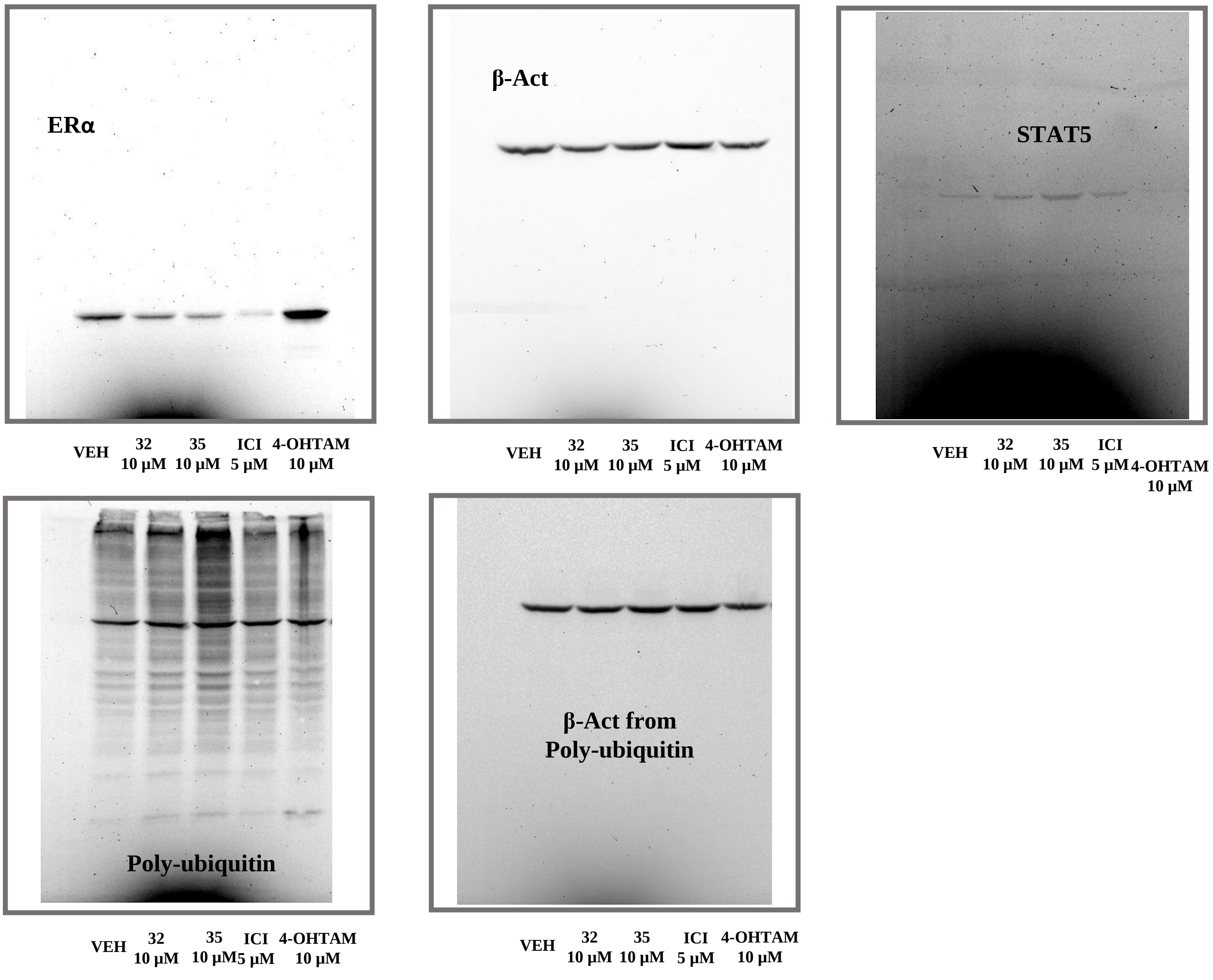

# Corresponding to Figure 14A (72h)

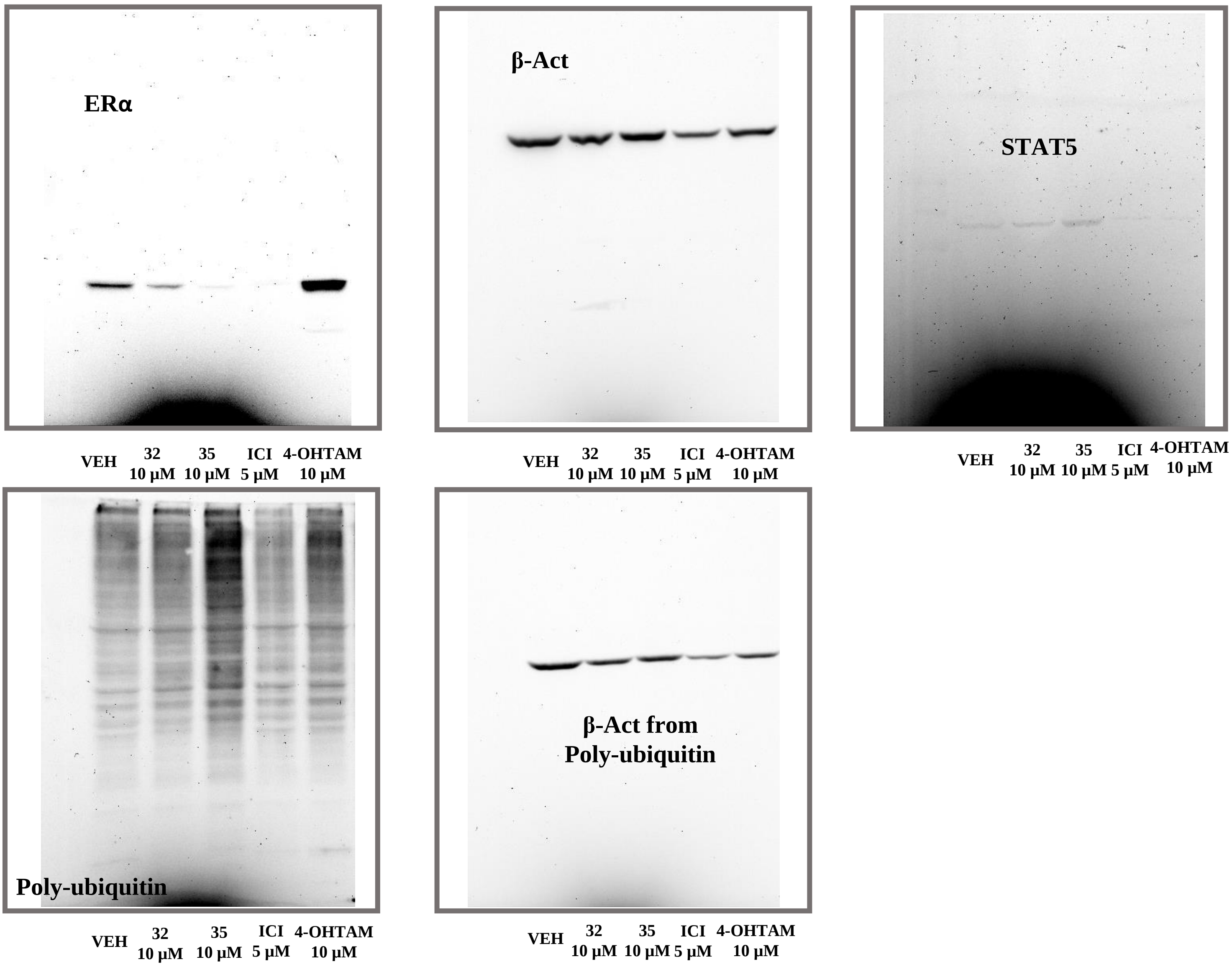

Corresponding to Figure 14B (48h)

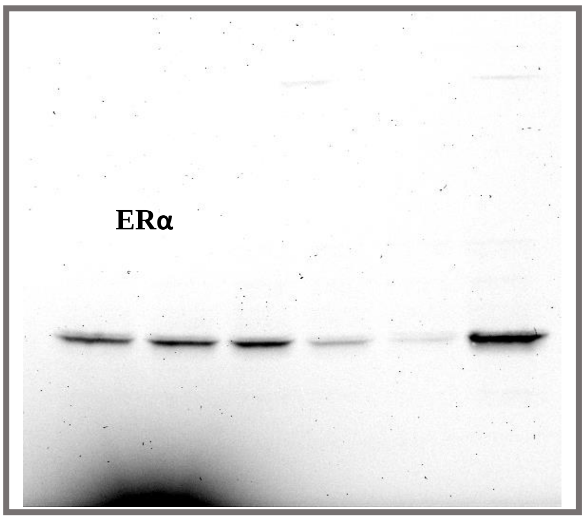

VEH | 35 3 μM | 35 5 μM | 35 10 μM | ICI 5 μM | 4-OHTAM 10 μM

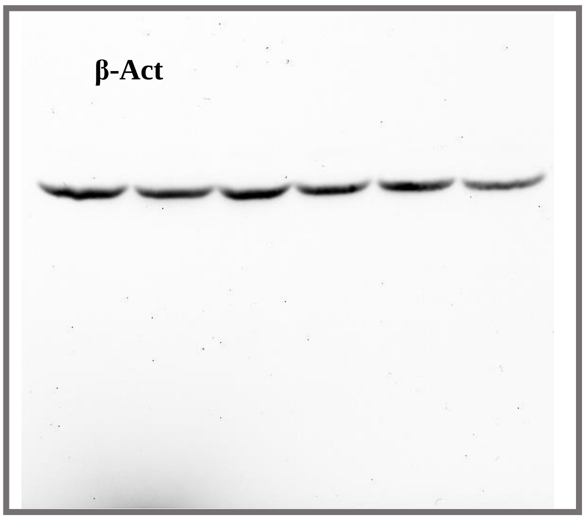

VEH | 35 3 μM | 35 5 μM | 35 10 μM | ICI 5 μM | 4-OHTAM 10 μM

Corresponding to Figure 14B (72h)

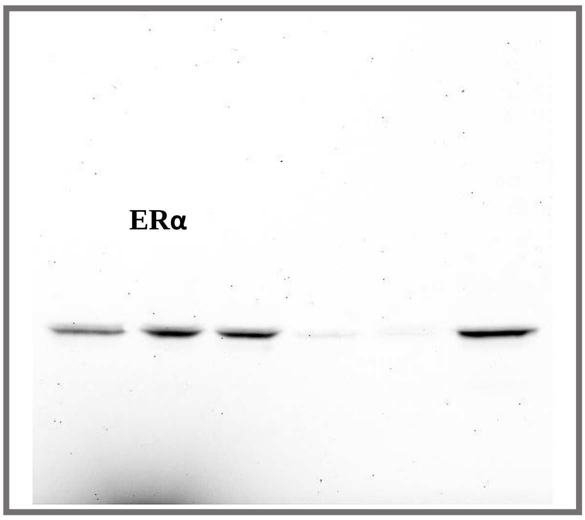

VEH | 35 3 μM | 35 5 μM | 35 10 μM | ICI 5 μM | 4-OHTAM 10 μM

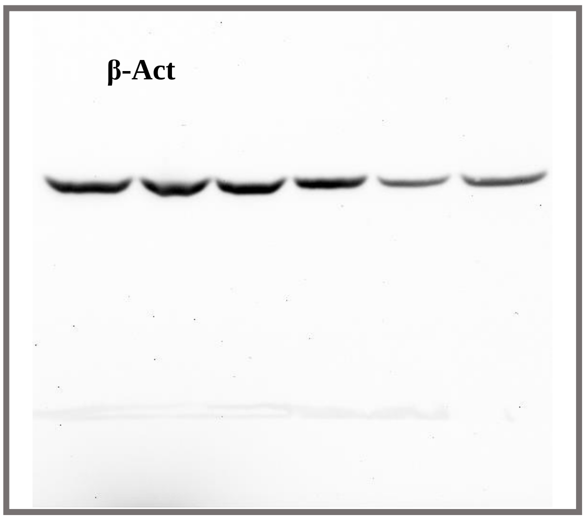

VEH | 35 3 μM | 35 5 μM | 35 10 μM | ICI 5 μM | 4-OHTAM 10 μM

Corresponding to Figure 14C (72h)

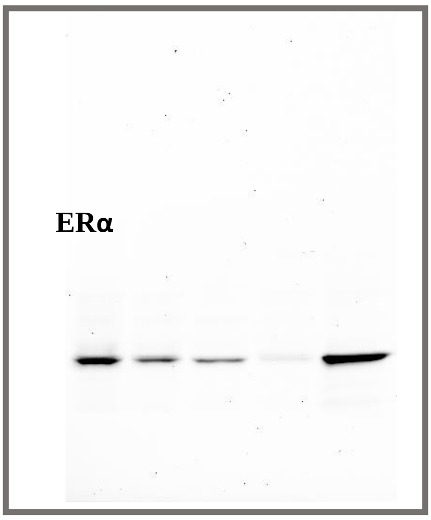

VEH | 32 5 μM | 32 10 μM | ICI 5 μM | 4-OHTAM 10 μM

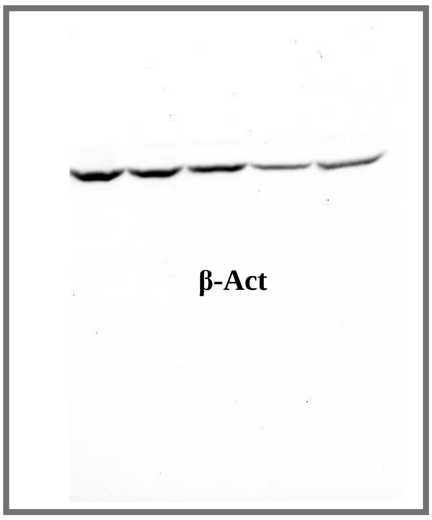

VEH | 32 5 μM | 32 10 μM | ICI 5 μM | 4-OHTAM 10 μM
