# Supplementary material for: Discovery of Highly Functionalized 5-hydroxy-2H-pyrrol-2-ones That Exhibit Antiestrogenic Effects in Breast and Endometrial Cancer Cells and Potentiate the Antitumoral Effect of Tamoxifen
Source: Cancers (Basel). 2022 Oct 22;14(21):5174. doi: 10.3390/cancers14215174 (PMC9655618; doi:10.3390/cancers14215174)
Supplement: Supplementary file 1 [file cancers-14-05174-s001.zip › Table S1.pdf]

ID Compound	Docking score	Docking score	Docking score
	PDB 1X7R	PDB 1ERR	PDB 3ERT
**E2**	**-11,2**	**–**	**–**
**RAL**	**–**	**-14,67**	**-12,88**
**4-OHTAM**	**–**	**-11,79**	**-10,94**
**4**	**-8,28**	**-10,95**	**-9,61**
**5**	**-9,27**	**-11,25**	**-9,29**
**6**	**-7,95**	**-9,21**	**-9,46**
**7**	**-7,89**	**-10,69**	**-9,13**
**8**	**-8,89**	**-10,81**	**-9,36**
**9**	**–**	**-10,15**	**-5,13**
**10**	**-9,3**	**-11,23**	**-9,48**
**11**	**-8,03**	**-11,45**	**-9,03**
**12**	**-8,17**	**-10,24**	**-5,16**
**13**	**-8,57**	**-10,92**	**-9,55**
**14**	**-9,3**	**-10,98**	**-9,25**
**15**	**-6,98**	**-9,59**	**-5,64**
**16**	**-8,32**	**-11,26**	**-7,28**
**17**	**-8,51**	**-11,35**	**-9,25**
**18**	**-3,51**	**-10,12**	**-5,47**
**19**	**-8,25**	**-10,69**	**-9,66**
**20**	**-9,29**	**-10,99**	**-9,11**
**21**	**-3,27**	**-9,73**	**–**
**22**	**-7,76**	**-10,95**	**-9,48**
**23**	**-9,21**	**-10,89**	**-9,3**
**24**	**–**	**-9,54**	**–**
**25**	**-8,02**	**-11,2**	**-6,76**
**26**	**-9,36**	**-11,13**	**-9,48**
**27**	**–**	**-10,46**	**-4,86**
**28**	**-7,7**	**-10,66**	**-9,22**
**29**	**-8,2**	**-10,92**	**-9,37**
**30**	**-3,18**	**-10,32**	**-6,64**
**31**	**-7,46**	**-11,01**	**-7,27**
**32**	**-9,3**	**-11,25**	**-9,35**
**33**	**-6,27**	**-9,77**	**-5,74**
**34**	**-8,71**	**-11,08**	**-8,51**
**35**	**-9,45**	**-11,19**	**-9,44**
**36**	**-8,11**	**-11,08**	**-5,51**
**37**	**-8,74**	**-11,58**	**-5,68**
**38**	**–**	**-11,44**	**-4,72**
**39**	**-5,26**	**-11,01**	**-5,55**
**40**	**-8,14**	**-10,87**	**-9,6**

**41**	**–**	**-9,61**	**-4,98**
**42**	**-7,07**	**-10,7**	**-5,79**
**43**	**-7,73**	**-10,56**	**-8,45**
**44**	**–**	**-9,64**	**-5,21**
**45**	**-7,69**	**-10,72**	**-4,33**
**46**	**-8,57**	**-11,83**	**-8,09**
**47**	**–**	**-11,27**	**-5,48**
**48**	**-7,8**	**-10,64**	**-4,39**
**49**	**-8,25**	**-11,12**	**-8,87**
**50**	**-3,14**	**-11,08**	**-4,43**

Supplementary Table S1. Molecular docking study of the 5-hydroxy-3,5-diaryl-1,5-dihydro-*2H*-pyrrol-2-one compounds (4-50). The docking score values (in kcal/mol) were calculated for the agonist conformational pose with genistein (G-Score PDB 1X7R) and the antagonist conformational poses with raloxifene (G-Score PDB 1ERR) and 4-OHTAM (G-Score PDB 3ERT).
