# Supplementary material for: Discovery of Highly Functionalized 5-hydroxy-2H-pyrrol-2-ones That Exhibit Antiestrogenic Effects in Breast and Endometrial Cancer Cells and Potentiate the Antitumoral Effect of Tamoxifen
Source: Cancers (Basel). 2022 Oct 22;14(21):5174. doi: 10.3390/cancers14215174 (PMC9655618; doi:10.3390/cancers14215174)
Supplement: Supplementary file 1 [file cancers-14-05174-s001.zip › Table S2.pdf]

Descriptor / Compound	#stars	CNS	QPlogBB	QPPCaco	QPPMDCK	QPlogKhsa	QPlogPo/w	QPlogKp	QPlogS	#metab	%HOA	HOA	PSA	SASA	MW	#rotor	donorHB	accptHB	volume
compd 4 R	0	1	0,035	3335,7	1819,1	0,858	4,733	-1,04	-5,925	2	100	3	42,9	613,4	333,4	1	1	3,75	1104,1
compd 4 S	0	0	-0,013	2870,4	1546,4	0,826	4,594	-1,20	-5,658	2	100	3	43,2	598,6	333,4	1	1	3,75	1090,8
compd 9 R	0	0	-0,190	2819,6	1516,9	0,744	4,759	-0,79	-6,054	3	100	3	59,7	669,8	387,4	3	1	5,25	1206,4
compd 9 S	0	0	-0,199	2786,0	1497,3	0,745	4,762	-0,79	-6,094	3	100	3	59,7	672,0	387,4	3	1	5,25	1207,4
compd 16 R	1	1	0,066	3361,6	3281,2	0,920	5,096	-1,24	-6,614	3	100	1	50,8	665,1	381,4	2	1	4,50	1199,5
compd 16 S	1	1	0,059	3313,2	3230,7	0,925	5,104	-1,25	-6,640	3	100	1	51,5	666,5	381,4	2	1	4,50	1201,5
compd 26 R	1	0	-0,071	3432,1	1876,0	0,593	4,519	-0,43	-5,309	3	100	3	63,7	627,7	385,4	3	1	5,25	1146,8
compd 26 S	0	0	-0,130	2754,0	1478,8	0,549	4,280	-0,77	-4,817	3	100	3	64,3	599,7	385,4	3	1	5,25	1125,8
compd 32 R	0	0	-0,178	2748,8	1475,8	1,040	5,337	-0,87	-6,330	5	100	1	44,2	654,0	369,5	3	1	3,75	1201,6
compd 32 S	0	0	-0,159	2803,2	1507,3	1,030	5,313	-0,87	-6,192	5	100	3	44,3	646,3	369,5	3	1	3,75	1197,4
compd 34 R	0	-2	-1,012	396,9	182,2	0,818	4,057	-2,92	-6,182	3	100	3	87,9	657,4	378,4	2	1	4,75	1180,5
compd 34 S	0	-2	-1,009	391,7	179,6	0,820	4,053	-2,94	-6,151	3	100	3	88,6	655,6	378,4	2	1	4,75	1181,0
compd 35 R	0	-2	-1,158	325,6	147,1	0,699	4,060	-2,44	-5,516	4	96	3	89,3	638,3	386,4	4	1	4,75	1165,5
compd 35 S	0	-2	-1,153	316,6	142,7	0,705	4,058	-2,47	-5,457	4	96	3	89,7	634,9	386,4	4	1	4,75	1167,0
compd 38 R	0	-2	-1,273	336,6	152,5	0,692	4,060	-2,69	-6,210	5	96	1	104,6	709,1	432,4	4	1	6,25	1280,9
compd 38 S	0	-2	-1,294	332,8	150,6	0,691	4,060	-2,69	-6,273	5	96	1	104,6	712,6	432,4	4	1	6,25	1281,2
compd 43 R	0	-2	-1,160	353,0	160,5	0,696	4,130	-0,51	-5,423	4	97	3	97,7	657,8	416,4	5	1	5,50	1234,3
compd 43 S	2	-2	-1,501	292,6	131,1	0,829	4,501	-2,13	-6,798	4	100	1	97,1	735,0	416,4	5	1	5,50	1293,3
compd 49 R	0	-2	-1,175	338,7	153,5	0,720	4,188	-2,50	-5,465	4	97	3	95,3	660,2	416,4	5	1	5,50	1243,0
compd 49 S	0	-2	-1,155	342,6	155,4	0,713	4,166	-2,51	-5,382	4	97	3	96,6	655,5	416,4	5	1	5,50	1239,8
Range/ Recomended values	0 to 5	-2 (inactive), +2 (active)	-3.0 to 1.2	<25 poor, >500 great	<25 poor, >500 great	-1.5 to 1.5	-2.0 to 6.5	-8.0 to -1.0	-6.5 to 0.5	1 to 8	>80% high <25% poor	1 (low); 2 (medium); 3 (high)	7.0 to 200.0	300.0 to 1000.0	130.0 to 725.0	0 to 15	0.0 to 6.0	2.0 to 20.0	500.0 to 2000.0

Supplementary Table S2. Computational pharmacokinetic parameters (predictive ADME) of the 5-hydroxy-3,5-diaryl-1,5-dihydro-*2H*-pyrrol-2-one enantiomers R and S of compounds 4, 9, 16, 26, 32, 34, 35, 38, 43, 49. #stars (number of property values that fall outside the 95% range of similar values for known drugs), CNS (predicted central nervous system activity on a -2 (inactive) to +2 (active) scale), QPlogBB (predicted brain/blood partition coefficient), QPPCaco (predicted human epithelial colorectal adenocarcinoma cell line permeability in nm/s), QPPMDCK (predicted Madin-Darby canine kidney permeability in nm/s), QPlogKhsa (predicted binding to human serum albumin), QPlogPo/w (predicted octanol/water partition coefficient), QPlogKp (predicted skin permeability), QPlogS (predicted aqueous solubility), #metab (number of likely metabolic reactions), %HOA (predicted human oral absorption on 0 to 100%), HOA (predicted qualitative human oral absorption), PSA (Van der Waals surface area of polar nitrogen and oxygen atoms and carbonyl atoms), SASA (total solvent accessible surface area), MW (molecular weight), #rotor (number of non-trivial, non-hindered rotable bonds), donorHB (number of hydrogen bond donors), accptHB (number of hydrogen bond acceptors).
